# Merging enzymes with chemocatalysis for amide bond synthesis

**DOI:** 10.1038/s41467-022-28005-4

**Published:** 2022-01-19

**Authors:** Luis Bering, Elliott J. Craven, Stanley A. Sowerby Thomas, Sarah A. Shepherd, Jason Micklefield

**Affiliations:** Department of Chemistry and Manchester Institute of Biotechnology, The University of Manchester, 131 Princess Street, Manchester, M1 7DN UK

**Keywords:** Biocatalysis, Sustainability

## Abstract

Amides are one of the most fundamental chemical bonds in nature. In addition to proteins and other metabolites, many valuable synthetic products comprise amide bonds. Despite this, there is a need for more sustainable amide synthesis. Herein, we report an integrated next generation multi-catalytic system, merging nitrile hydratase enzymes with a Cu-catalysed N-arylation reaction in a single reaction vessel, for the construction of ubiquitous amide bonds. This synergistic one-pot combination of chemo- and biocatalysis provides an amide bond disconnection to precursors, that are orthogonal to those in classical amide synthesis, obviating the need for protecting groups and delivering amides in a manner unachievable using existing catalytic regimes. Our integrated approach also affords broad scope, very high (molar) substrate loading, and has excellent functional group tolerance, telescoping routes to natural product derivatives, drug molecules, and challenging chiral amides under environmentally friendly conditions at scale.

## Abstract (web summary)

Proteins, other metabolites and many valuable synthetic products contain amide bonds and there is a need for more sustainable amide synthesis routes. Here the authors show an integrated next generation multi-catalytic system, merging nitrile hydratase enzymes with a Cu-catalysed N-arylation reaction in a single reaction vessel, for the construction of ubiquitous amide bonds.

## Introduction

**Fig. 1:**
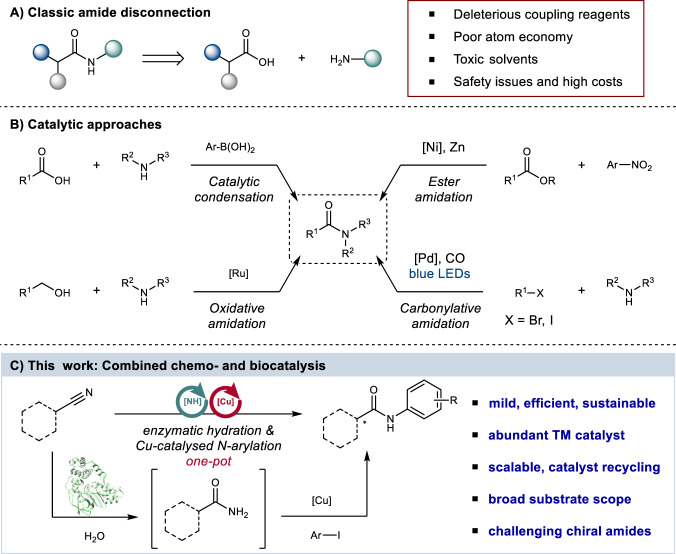
Approaches towards the construction of amide bonds. **A** Retrosynthetic analysis for classic amide disconnection. **B** Catalytic approaches for amide bond synthesis. **C** Amide bond synthesis by integrating nitrile hydratase enzymes with Cu-catalysed Ullmann-type arylation.

The formation of amide bonds from carboxylic acids and amines is of fundamental importance in nature^1^. A high proportion of essential chemicals and materials are also constructed from amide bonds, including pharmaceuticals, where amide synthesis is the most frequently conducted reaction^2^. Traditional amide coupling of acids and amines is, however, increasingly unsustainable, requiring stoichiometric amounts of costly and deleterious coupling reagents as well as undesirable organic solvents, all of which create significant problems in purification and waste processing (Fig. 1A)^3^. Atom inefficient protective-group chemistries are often necessary with multiple steps required to generate a single amide bond. Thus, atom efficient and benign catalytic methods are urgently required to produce amides^4^. The development of catalytic methods to access amide containing molecules has received increasing attention^5^, with examples including direct amidation via boron-based catalysis^6^, oxidative amination^7^, ester amidation^8^ and carbonylative amidation^9^ (Fig. 1B). However, these approaches have not been widely adopted, due to limitations in scale, substrate scopes, efficiency, toxicity, and sustainability. Enzymatic amide synthesis is promising^10^, but so far this is limited to ligase enzymes that couple a narrow range of amines with carboxylic acid substrates, requiring expensive adenosine triphosphate (ATP) as a co-factor^11–13^. Alternatively, amides have been prepared using hydrolase enzymes to couple amines with carboxylate esters, which often require organic solvents to overcome competing hydrolysis^12,13^. Given the urgent need for greener and more efficient catalytic methods to generate amides, an alternative approach to construct this fundamental bond is desirable, particularly if this affords an alternative disconnection to abundant feedstock chemicals, orthogonal to classical amide precursors, and avoids the use of protective groups.

The combination of chemocatalysis and biocatalysis in the same reaction has emerged as an efficient and environmentally benign alternative to traditional chemical methods. Whilst such integrated catalysis has yet to be applied to catalytic amide synthesis, it has the potential to improve overall synthetic efficiency through decreasing solvent consumption and waste production, whilst reducing work-up and isolation steps^14^. Integrated processes in aqueous media are particularly desirable from a green chemistry perspective^15^. Combining multiple catalysts in a cascade can also facilitate the processing of unstable intermediates and overcome unfavourable reaction equilibria^16^. However, mutual inactivation and different operating conditions of chemo- and biocatalysts present a major challenge for the development of integrated processes^17^. Despite this, the merger of transition metals (TM) and biocatalysts has led to some notable cascade-type processes, including the combination of stereoselective bioreduction with Pd or Ni catalysts^18,19^. The merger of halogenase enzymes with Pd catalysed cross-coupling has also been deployed for the construction of C−C bonds via a net C−H bond functionalisation process^20,21^. In addition, photoredox catalysis has been recently successfully implemented along with enzyme catalysis in an integrated fashion^22–24^.

In this work, we describe the development of a hybrid chemobiocatalytic process incorporating nitrile hydratase (NHase) enzymes and transition-metal catalysed C−N bond formation to deliver diverse target amides (Fig. 1C). Selective and mild enzymatic hydration of organic nitriles generates primary amide intermediates combined with an in situ N-arylation using an inexpensive, earth-abundant Cu-catalyst. Issues of catalyst compatibility were overcome by retaining NHase enzymes within cells, while very high substrate loadings were achieved by the inclusion of micellar organo-compartments, enabling clean, practical, and scalable synthesis of diverse target amides.

## Results

### Development of an integrated amide bond synthesis approach

We chose to explore NHase hydration of nitriles with concomitant TM-catalysed functionalisation of the intermediate primary amides, as NHase enzymes have a broad substrate scope, operate under mild conditions and are used at scale for industrial applications^25^. Chemocatalytic hydration of nitriles requires excess amounts of strong bases, acids or explosive peroxides, often under harsh conditions, and are poorly tolerated by other sensitive functional groups^26^. Although a number of TM-catalysts may be deployed to derivatise primary amides, Cu-catalysed Ullmann-type N-arylation was considered most practical, versatile, and suitable for aqueous media^27–29^. The combination of NHase with Ullmann-type coupling opens up disconnections to organo nitrile and aryl halide precursors which are readily available from existing inexpensive feedstocks, or can be assembled efficiently via chemo-, bio- or hybrid-catalytic routes^21,30–32^. The nitrile and aryl halide groups are also relatively inert, affording orthogonality to other common functionalities, which can negate the need for protecting groups.

We began by screening various Cu-catalysts for the coupling of benzamide (**2**) with iodobenzene (**3**) under benign aqueous conditions (Supplementary Table 1). Most of the chemocatalysts tested typically operate at molar concentrations or under neat reaction conditions and were thus ineffective at the lower substrate concentrations that are optimal for NHase activity. However, the synthesis of amide **4** was achieved with an isolated yield of 87% following systematic optimisation at 50 mM substrate concentration, using inexpensive CuBr_2_ catalyst, *trans*-*N*,*N*’-dimethylcyclohexane-1,2-diamine (**L1**) ligand, d-glucose reductant, in aqueous buffer with *i*PrOH co-solvent (Supplementary Table 2).

**Fig. 2:**
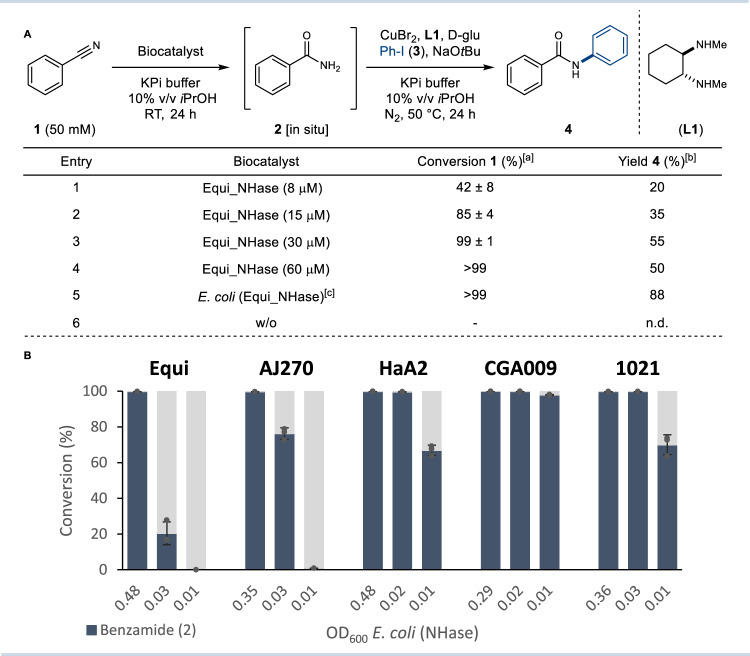
Development of the integrated chemo- and biocatalytic synthesis of amides. **A** Optimisation of the integrated reaction. Reaction conditions: 1. Nitrile (**1**) (50 mM), Equi_NHase (see table) in 0.1 M KPi buffer (pH = 7.8)/10% v/v *i*PrOH (2 mL) at RT, 24 h; 2. CuBr_2_ (10 mol%), **L1** (20 mol%), d-glu (20 mol%), **3** (150 mM) at 50 °C, 24 h under N_2_ atmosphere (headspace purge). [a] Conversion determined in triplicates by HPLC/UV using benzophenone as external standard. [b] Yield of isolated product after column chromatography. [c] *E. coli* (Equi_NHase) whole cells from ca. 20 mL cell culture (OD_600_ = ~0.5) were used. **B** Activity screening of *E. coli* (NHase) whole cells. Relative conversion was determined in triplicates by HPLC/UV analysis (*n* = 3). Blue bars represent mean values and error bars represent ± SEM of benzamide (**2**) conversion. n.d. not detected; w/o without, OD_600_ Optical density measured at 600 nm.

With reaction conditions for the Ullmann-type arylation of benzamides in aqueous buffer in hand, the integrated chemo- and biocatalytic synthesis of **4** was studied using benzonitrile (**1**) as the starting material (Fig. 2A). Initial screening was performed with the NHase enzyme from *Rhodoccocus equi* (Equi*_*NHase), which had been shown to be a useful biocatalyst with aromatic substrates^33^. The Equi*_*NHase was overproduced in *Escherichia coli* using standard conditions and found to quantitatively hydrate **1** to primary amide **2** (Fig. 2A, entry 3–5). However, subsequent integration of purified Equi*_*NHase with chemocatalytic N-arylation led to incomplete conversion of **2** with a maximum isolated yield of 55% of amide **4** (Fig. 2A, entry 1–3). Attempts to improve the integrated reaction, including increasing the concentration of the NHase, were largely unsuccessful, which could be due to the incompatibility of two catalytic systems (Fig. 2A, entry 4). To address this, the reaction was repeated using intact *E. coli* cells overproducing Equi*_*NHase, rather than purified enzyme. Under these conditions, a major improvement was observed, with **4** produced in an isolated yield of 88% (Fig. 2A, entry 5). This increased productivity is likely due to the physical separation of the chemo- and biocatalysts, provided by the *E. coli* cell membrane, preventing negative cross-talk that may lead to mutual inactivation of the catalysts. In addition to being more productive, the use of whole cells is also more practical, as it avoids laborious protein purification, which is also costly when performed at scale. Control experiments using *E. coli* cells lacking the NHase biocatalyst did not result in any formation of **4**, confirming that non-specific hydration of nitrile **1** does not occur (Fig. 2A, entry 6). Taken together, these results demonstrate that NHase and Cu-catalysts, both of which are robust, easily prepared, and inexpensive, can be merged in a single reaction vessel providing the amide product in excellent yield, without removing any reaction components or changing solvent.

To broaden the biocatalysis toolkit for integrated amide synthesis, the activity of *E. coli* cells transformed with other NHase genes from different origins were determined for the hydration of benzonitrile (**1**) to benzamide (**2**) (Fig. 2B)^34,35^. The Fe(II)-dependent NHase enzymes from *Rhodococcus equi* (Equi_NHase) and *Rhodococcus erythropolis* AJ270 (AJ270) showed lower activity compared to Co(III)-dependent NHase enzymes from *Rhodopseudomonas palustris* HaA2 (HaA2), *Rhodopseudomanas palustris* CGA009 (CGA009) and *Sinorhizobium meliloti* 1021 (1021), with CGA009 showing highest activity against **1**. In addition to these enzymes, many other NHase enzymes are available in nature, providing a flexible array of robust and efficient biocatalysts for integrated amide synthesis without the need for laborious protein engineering.

### Integrated amide bond synthesis from aromatic nitriles

**Fig. 3:**
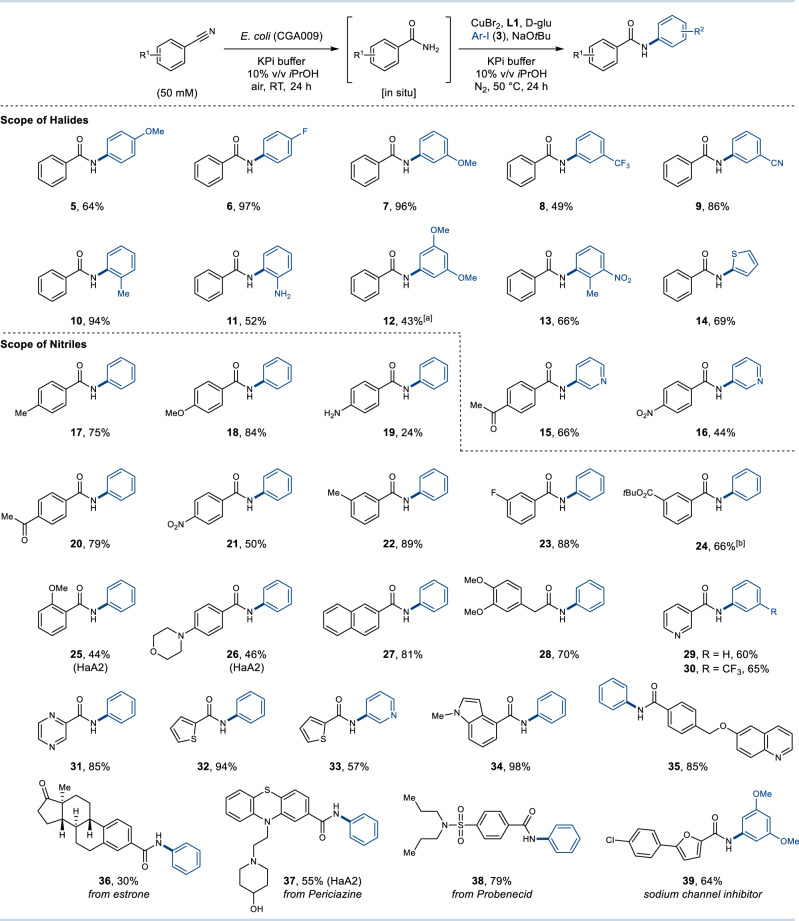
Scope for the integrated chemo- and biocatalytic synthesis of aromatic amides. Reaction conditions: 1. Nitrile (50 mM), *E. coli* (CGA009) whole cells in 0.1 M KPi buffer (pH = 7.8)/10% v/v *i*PrOH (4 mL) at RT, 24 h; 2. CuBr_2_ (10 mol%), **L1** (20 mol%), d-glu (20 mol%), iodoarene (150 mM) at 50 °C, 24 h under N_2_ atmosphere (Method A). Yields refer to isolated products after column chromatography. [a] Amide **12** was synthesised using PhCN (150 mM) and iodoarene (50 mM). [b] Amide **24** was synthesised using *E. coli* (CGA009) whole cells for 48 h.

With optimised conditions for the integrated chemo- and biocatalytic synthesis of amides in hand, the scope of the reaction was explored on a preparative scale (Method A) (Fig. 3). Initially, the compatibility of structurally and electronically diverse iodoarenes was tested, using the *E. coli* (CGA009) cells that afford high activity and preference for aromatic nitrile substrates. Electron-donating and -withdrawing groups were well tolerated on the *para*- (**5** and **6**), *meta*- (**7**–**9**) and *ortho*-position (**10** and **11**) of the iodoarenes. Notably, an additional nitrile group could be introduced in the iodoarene without further undesired hydration (**9**), potentially enabling a second amide bond to be constructed in a sequential fashion without additional manipulation. Selective N-arylation can also be achieved in the presence of an unprotected amine group (**11**). Next, polyfunctionalised iodoarenes were converted to the desired products in good yields (**12** and **13**). With **12**, the concentration of nitrile could be increased up to 150 mM enabling the iodoarene to be utilised as the limiting reagent (50 mM). Increasing the concentration of some nitrile substrates is however limited by their aqueous solubility. Finally, the utilisation of iodinated heterocycles was shown to provide amides (**14**–**16**) in good yields.

Preparative scale integrated reactions were also carried out to explore the nitrile substrate scope. Various functionalised aromatic nitriles with electron-donating or -withdrawing groups in the *para*- (**17**–**21**) or *meta*-position (**22**–**24**) were well tolerated affording amides in good to excellent yields using the CGA009 NHase (Fig. 3). The sterically demanding *tert*-butyl ester provided the desired product **24** in 66% yield. Enzymatic hydration in this example was extended to 48 h to ensure maximum conversion due to the low solubility of the nitrile precursor. Whilst *ortho*-substituted aryl nitriles were found to be poorly accepted by NHase CGA009, synthesis of *ortho*-methoxy benzamide derivative **25** could be realised using *E. coli* (HaA2) whole cells. Similarly, benzonitrile with a *para*-morpholine substituent was not accepted by CGA009 but was turned over by HaA2. The modest isolated yield of **26** resulted from an incomplete chemocatalytic arylation.

The broad scope of the integrated approach was further illustrated through the synthesis of naphthalenyl and benzyl amides (**27** and **28**) in 81% and 70% yield, respectively. Numerous heterocyclic nitriles also proved to be good substrates, providing pyridine (**29** and **30**), pyrazine (**31**), thiophene (**32** and **33**), indole (**34**) and quinoline (**35**) amides in excellent yields. The integrated method was further applied to generate amide derivatives of the natural product estrone (**36**) as well as drug molecules Periciazine (**37**) and Probenecid (**38**). Amide derivatives of **38** are inhibitors of carbonic anhydrase which have potential applications as anti-tumour agents^36^. The examples (**36**–**38**) underline the potential to utilise nitrile functional groups as efficient handles for late-stage derivatisation of more complex bioactive molecules. Whilst traditional carboxylic acid and amine synthons typically require protection, both the nitrile and halide coupling partners are relatively inert and can be tolerated through synthetic steps, before being selectively ‘unmasked’ by the NHase at a later stage following the integrated approach. Finally, the efficient synthesis of a selective inhibitor of the NaV_1.8_ sodium channel (**39**) was achieved in 64% yield to further demonstrate how the technology can be applied to other pharmaceutical targets^37^.

### Integrated amide bond synthesis from aliphatic nitriles

**Fig. 4:**
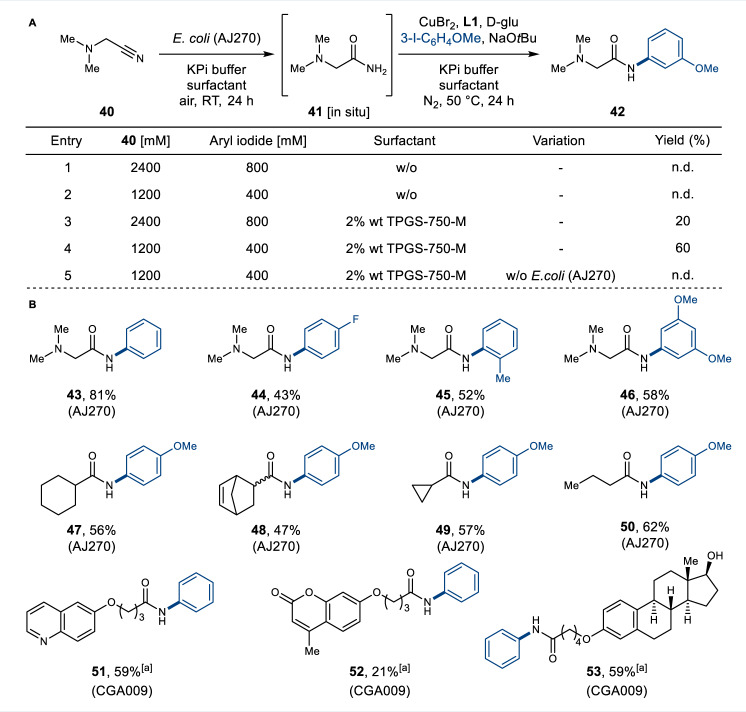
Integrated chemo- and biocatalytic synthesis of aliphatic amides. **A** Optimisation of reaction conditions. **B** Scope of reaction (Method B). Yields refer to isolated products after column chromatography. [a] Amides **51**–**53** were synthesised using nitrile (0.2 mmol) and halide (0.6 mmol). n.d. not detected, TPGS-750-M dl-α-Tocopherol methoxypolyethylene glycol succinate, w/o without.

The scope of the one-pot hydration/arylation cascade for aliphatic nitriles was tested next, with the transformation of nitrile **40** to amide **42** chosen as the model reaction (Fig. 4A). *E. coli* (AJ270) cells afford excellent conversions of **40** to the primary amide **41**, due to the preference of Fe-containing NHases for aliphatic substrates. Despite this, the aliphatic nitrile (**40**) proved unproductive in integrated reactions following the optimised conditions developed for the aromatic nitriles (Fig. 3). This is most likely due to the reduced reactivity of the aliphatic primary amide intermediate in the Cu-catalysed N-arylation reaction. Increasing substrate concentration also failed to deliver the desired amide **42** (Fig. 4A, entry 1,2). However, the addition of a surfactant (2% wt TPGS-750-M) to the cascade had a dramatic effect, resulting in the synthesis of the target product **42** in up to 60% isolated yield using the iodoarene as a limiting reagent (Fig. 4A, entry 3,4). Surfactants were recently found to enhance the rate of transition-metal catalysed reactions and biocatalytic reactions in an aqueous medium^38^. In the integrated amide synthesis it is likely that the micelles formed by the surfactant serve to accelerate the N-arylation, providing organic microcompartments solubilising and significantly increasing the effective concentration of the primary amide intermediate, iodoarene coupling partner, and the chemocatalyst. Using the whole cell biocatalysts and surfactant, very high concentrations of nitrile **40** (1200 mM) and iodoarene (400 mM) can be turned over, which can enable highly productive large-scale reactions (>100 g/L) to be performed. These conditions were successfully implemented in coupling nitrile **40** and a range of other of iodoarene possessing electron-donating and -withdrawing groups (**43**–**46**) (Method B) (Fig. 4B). Various cyclic and linear aliphatic nitriles were also converted to the corresponding amides (**47**–**50**) in good yields, using the versatile NHase AJ270.

To date, NHase has been mainly used to hydrate relatively small nitrile substrates. However, X-ray crystal structures of Fe- and Co-type NHase enzymes reveal relatively open active site architectures, with the metal centre positioned in a shallow, solvent-accessible channel^39,40^. In light of this, we reasoned those larger substrates, with a nitrile appended by a short alkyl chain, may be accepted by the NHase. This proved to be the case, using the larger and more valuable nitrile coupling partner as the limiting reagent. Desired conjugates of 4-hydroxy-quinoline (**51**), 4-methyllumbelliferone (**52**) and estradiol (**53)** were synthesised in good isolated yields.

### Enantioselectivity in the integrated amide bond synthesis

**Fig. 5:**
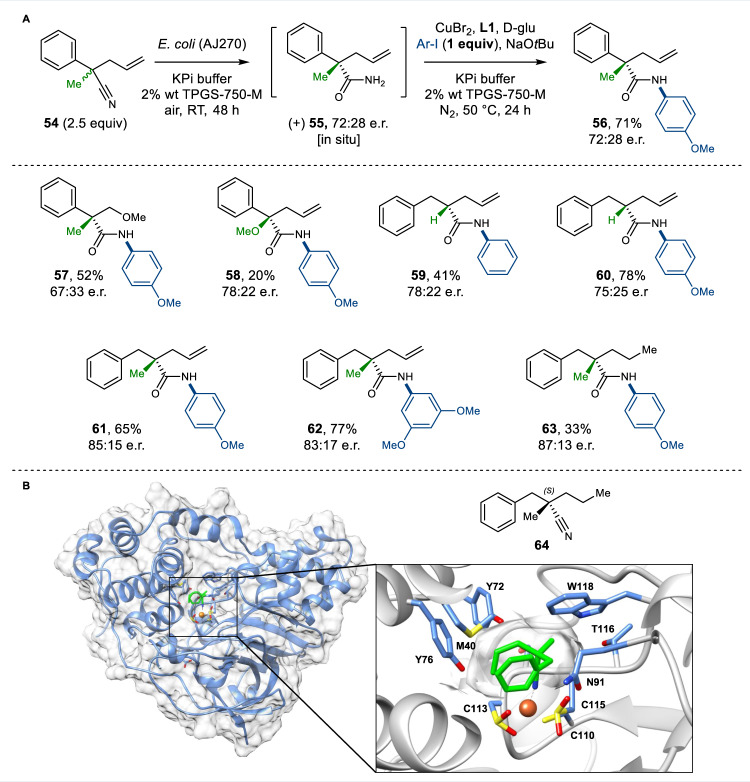
Integrated chemo- and biocatalytic synthesis of chiral amides. **A** Scope of the kinetic resolution of racemic nitriles (Method C). Yields refer to isolated products after column chromatography. TPGS-750-M dl-α-Tocopherol methoxypolyethylene glycol succinate; e.r. enantiomeric ratio. **B** Docking of nitrile **64** into the active site of AJ270 (pdb code: 2QDY) in a putative active conformation indicates a substrate-binding pose with coordination of the nitrile group to the Fe-centre, with stereoselectivity is controlled by binding of the propyl substituent to a hydrophobic pocket formed by M40, Y72, and Y76. Labelled active site residues (blue) and substrate **64** (green).

Nitrile hydratase are not reported to be highly stereoselective and thus far have had limited utility in asymmetric synthesis^34^. Nevertheless, we sought to explore if the chirality of the active site of NHases could be exploited in the kinetic resolution (KR) of racemic nitriles, with concomitant chemocatalytic derivatisation, generating valuable chiral amides in a single process. The enantioselective synthesis of these homochiral amides from racemic precursors is challenging using existing chemistries. The synthesis of the corresponding chiral carboxylic acid, required for a traditional amide coupling, can also involve multi-step synthesis using toxic and expensive transition-metals, tailored ligands or chiral auxiliaries^41,42^. Thus, a robust and practical stereoselective amide synthesis under the environmentally benign conditions would be an attractive alternative. The synthesis of enantioenriched amide **56** via KR of nitrile **54** was tested first (Method C) (Fig. 5A). KR of **54** with in situ N-arylation yielded the product **56** in good yields and reasonable 72:28 e.r., using iodoarenes as limiting reagents. Comparable results were observed in the synthesis of ether-functionalised amide product **57** and amide **58**. Further, the KR of tertiary *α*-allyl nitriles was explored, affording chiral amides **59** and **60** in good yield and 78:22 e.r. and 75:25 e.r. respectively. Resolution of challenging quaternary chiral centres was also achieved in good yields and in up to 85:15 e.r. (**61** and **62**), while the propyl substituted amide **63** was produced with a good 87:13 e.r. In addition, primary amide intermediate **55** was isolated and characterised to assign the absolute configuration of products (see Supplementary Information for the details). Although the NHase (AJ270) is not completely enantioselective in the examples shown, there is scope for improvement. Many other NHase exist in nature that could be screened for greater selectivity. Moreover, available NHase X-ray crystal structures could enable structure-guided engineering to generate variants with improved stereoselectivity. For example, through docking studies (Fig. 5B) we suggest that nitrile substrate **64** coordinates to the Fe-centre in the NHase AJ270 active site, with substituents on the α-position of **64** occupying two relatively small pockets formed by either Y72, M40, and Y76 or N91, T116, and W118 residues. The relatively small difference in size of these two pockets may explain the lower stereoselectivity observed in the KR with some of the nitrile substrates tested. Nevertheless, the model does show how the (*S*)-enantiomer of **64** can be favourably accommodated, with the flexible propyl substituent occupying the pocket formed by Y72, M40, and Y76 residues, which is consistent with the higher enantioselectivity observed for **64**.

### Further application of the integrated amide bond synthesis

**Fig. 6:**
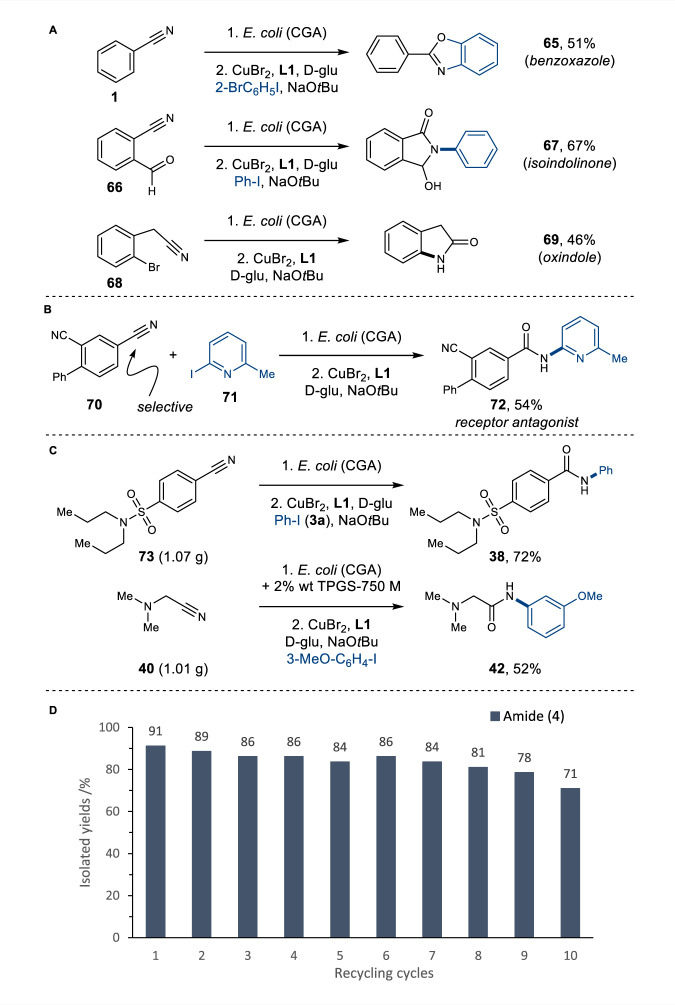
Application of the integrated chemo- and biocatalytic synthesis of amides. **A** Integrated synthesis of heterocycles (Method A). **B** Selective hydration of dinitrile **70** for the synthesis of bioactive product **72** (Method A). **C** Gram-scale reactions (Method A and B, see Supplementary Information for the details). **D** Biocatalyst recycling experiment. Experimental procedures can be found in the Supplementary Information. Yields refer to isolated products after column chromatography.

The utility of the chemo-biocatalytic methodology was further exemplified in the synthesis of valuable heterocyclic motifs (Method A) (Fig. 6A). Consecutive Cu-catalysed C–N and C–O bond formation enabled the synthesis of benzoxazole (**65**) in one-pot with 51% yield, whilst annulation employing 2-formyl benzonitrile (**66**) gave isoindolinone (**67**) in 67% isolated yield. Intramolecular ring-closure also yielded the oxindole scaffold **69** in 46% yield. In addition to stereoselectivity, the regioselectivity afforded by NHase can also be advantageous. To illustrate this, glutamate receptor antagonist **72** was produced in a single integrated process, from a dinitrile precursor **70**, exploiting the selectivity of CGA009 enzyme for the less hindered *para*-nitrile group (Method A) (Fig. 6B)^43^. To the best of our knowledge, there is no chemocatalyst available that allows for selective mono hydration of polynitriles based on steric control. Moreover, using the integrated process, the drug candidate **72** can be delivered in two steps from commercially available reagents with 49% overall yield, which is more efficient than the reported synthesis of **72**, which requires six linear steps, yielding the target amide in 24% overall yield.

To demonstrate the ease by which our integrated reactions could be scaled up, the syntheses of probenecid amide **38** and aliphatic amide **42** were carried out on a gram-scale (Fig. 6C). We conducted the scale-up by increasing the respective substrate loading, but without altering the developed reaction conditions. While amide **38** was synthesised at 50 mM nitrile concentration using 1 g of aromatic nitrile **73** (Method A; see the Supporting Information for the details), amide **42** was synthesised at 1200 mM nitrile concentration (Method B; see the Supporting Information for the details) using 1 g of aliphatic nitrile **40** in the buffer/surfactant solvent system. Both scale up reactions delivered the target amides without affecting the overall yields. With additional process development, we envisage these reactions could be scaled up further, for future biomanufacturing. Finally, the potential for biocatalyst recycling was demonstrated by repeating the synthesis of benzamide **4** ten times using the same batch of *E. coli* (NHase) cells recovered from the reaction prior to the sequential addition of the chemocatalyst (Supplementary Information for the details). This demonstrated that the NHase is extremely robust and affords excellent yields in integrated reactions across eight or nine reaction cycles. Only after being recycled 10 times, did the activity of the NHase drop to a notable degree (Fig. 6D).

## Discussion

This study describes an efficient and mild method for the synthesis of amide containing molecules using an integrated chemo- and biocatalytic approach. Combining *E. coli* (NHases) cells with Cu-catalysed Ullmann-type coupling in the same reaction vessel enables selective hydration of widely available aromatic and aliphatic nitriles to primary amides, followed by in situ N-arylation with common iodoarenes coupling partners to afford diverse functionalised amide products. Attempts to reduce the Cu-catalyst loading to less than 10 mol% were found to be inefficient. However, the Ullmann-type arylation is considered to be an environmentally friendly alternative to other transition-metal catalysed reactions due the reduced toxicity, low pricing, earth-abundance and better environmental properties of Cu-catalysts. Other known chemical methods for N-arylation of primary amides are often conducted in organic solvents while still using comparable catalyst loadings, thus they do not represent a superior alternative^27^. Nevertheless, the future availability of more efficient chemocatalysts could potentially improve the sustainability of the integrated processes described here. Our methodology also avoids the use of harmful solvents, as both the chemo- and biocatalyst work efficiently in aqueous buffer supplemented with *i*PrOH as a safe, inexpensive, nontoxic, and biodegradable co-solvent. Chlorinated solvents were also avoided for work up procedures, instead, the recommended solvent ethyl acetate was used as a more environmental benign alternative^44^. Although the Cu-catalyst and enzymes used here have been characterised previously as stand-alone catalysts, the combination of the two enables an unusual and facile amide bond disconnection. Compared with conventional amide synthesis, our integrated approach affords improvements in space–time yields, negates the requirement for protecting groups and avoids harmful reagents. Nevertheless, overcoming mutual catalyst inactivation and establishing conditions favourable for both catalytic systems remains a major challenge in this field^14^. In addition to providing an amide bond disconnection to relatively inert precursors, orthogonal to common amide chemistries, the one-pot cascade displays excellent functional group tolerance and broad substrate scope with >50 examples provided, including one-pot synthesis of bioactive natural products and drug molecules. While providing excellent chemoselectivity, the approach also affords excellent regio- and stereo-selectivity that is difficult to achieve with chemocatalysis alone. Notably, KR of racemic nitriles with concomitant N-arylation was demonstrated, which would be extremely challenging or unachievable using the available chemo- or bio-catalytic systems. Although NHase enzymes are well known, we have expanded their substrate repertoire and exploited their selectivity in integrated reactions. We also demonstrate how the integrated reactions are easily scalable and the inclusion of micellar organo-compartments drastically increases the efficiency of the reaction at very high substrate concentrations (up to 1200 mM), which would otherwise be beyond the scope of biocatalysis. Surfactants are well known to enhance the reactivity of transition-metal catalysts in aqueous media^45^ and are also likely to become a key component in the development of chemobiocatalytic reactions in the future^38,46^. The chemo- and biocatalyst, which are both readily available, robust, and inexpensive, act synergistically to enable the synthesis of desired amides under very mild and environmentally benign aqueous reaction conditions in one pot. Taken together, these findings provide an alternative approach for the integration of chemo- and biocatalysis delivering functionalised amide products, under more sustainable conditions for a raft of important future applications.

## Methods

### Method A: Integrated chemo- and biocatalytic amide bond synthesis

To a solution of nitrile **(**0.2 mmol, 1 equiv) in KPi buffer (0.1 M, pH = 7.8)/10% v/v *i*PrOH were added *E. coli* (NHase) whole cells from ca. 10 mL cell culture (final OD_600_ ~ 0.1) in KPi buffer (0.1 M, pH = 7.8) to a total volume of 4 mL and the reaction was stirred (400 rpm) for 24 h at room temperature. After that time, halide (0.6 mmol, 3 equiv), ligand (8 µL, 0.04 mmol, 0.2 equiv), d-glucose (7 mg, 0.04 mmol, 0.2 equiv), CuBr_2_ (5 mg, 0.02 mmol, 0.1 equiv), and NaO*t*Bu (39 mg, 0.4 mmol, 2 equiv) were successively added, and the reaction was vigorously stirred (1200 rpm) at 50 °C under N_2_ atmosphere (headspace purge) for 24 h. After cooling to room temperature, the reaction was diluted with ethyl acetate and filtered through a pad of Celite^©^. The solution was washed with brine (10 mL), the aqueous phase was extracted with ethyl acetate (2 × 10 mL), and the combined organic phases were dried over anhydrous MgSO_4_ and concentrated under reduced pressure. Silica gel column chromatography afforded the desired product.

### Method B: Integrated chemo- and biocatalytic amide bond synthesis mediated by surfactant TPGS-750-M

To a solution of nitrile (0.6 mmol, 3 equiv) in ~2 wt% TPGS-750-M/KPi buffer (0.1 M, pH = 7.8) were added *E. coli* (NHase) whole cells from ca. 20 mL cell culture in 2 wt% TPGS-750-M/KPi buffer (0.1 M, pH = 7.8) to a total volume of 0.5 mL and the reaction was stirred (400 rpm) for 24 h at room temperature. After that time, halide (0.2 mmol, 1 equiv), ligand (8 µL, 0.04 mmol, 0.2 equiv), d-glucose (7 mg, 0.04 mmol, 0.2 equiv), CuBr_2_ (5 mg, 0.02 mmol, 0.1 equiv), and NaO*t*Bu (39 mg, 0.4 mmol, 2 equiv) were successively added, and the reaction was vigorously stirred (1200 rpm) at 50 °C under N_2_ atmosphere (headspace purge) for 24 h. After cooling to room temperature, the reaction was diluted with ethyl acetate and filtered through a pad of Celite^©^. The solution was washed with brine (10 mL), the aqueous phase was extracted with ethyl acetate (2 × 10 mL), and the combined organic phases were dried over anhydrous MgSO_4_ and concentrated under reduced pressure. Silica gel column chromatography afforded the desired product.

### Method C: KR via integrated chemo- and biocatalytic amide bond synthesis

To a solution of nitrile (0.5 mmol, 2.5 equiv) in ~2 wt% TPGS-750-M/KPi buffer (0.1 M, pH = 7.8) were added *E. coli* (AJ270) whole cells from ca. 20 mL cell culture in 2 wt% TPGS-750-M/KPi buffer (0.1 M, pH = 7.8) to a total volume of 0.5 mL and the reaction was stirred (400 rpm) for 24 h at room temperature. After that time, halide (0.2 mmol, 1 equiv), ligand (8 µL, 0.04 mmol, 0.2 equiv), d-glucose (7 mg, 0.04 mmol, 0.2 equiv), CuBr_2_ (5 mg, 0.02 mmol, 0.1 equiv), and NaO*t*Bu (39 mg, 0.4 mmol, 2 equiv) were successively added, and the reaction was vigorously stirred (1200 rpm) at 50 °C under N_2_ atmosphere (headspace purge) for 24 h. After cooling to room temperature, the reaction was diluted with ethyl acetate and filtered through a pad of Celite^©^. The solution was washed with brine (10 mL), the aqueous phase was extracted with ethyl acetate (2 × 10 mL) and the combined organic phases were dried over anhydrous MgSO_4_ and concentrated under reduced pressure. Silica gel column chromatography afforded the desired product.

## Supplementary information

Supporting Information

## Data Availability

The original materials and data that support the findings of this study, including but not limited to molecular modelling, HPLC, NMR data, are either available within the paper or are available from the corresponding author upon request. The work is carried out in line with studies in the synthetic chemistry field. Enzymes are produced using established procedures and used in preparative scale synthetic reactions, with isolated yields reported and products characterised by ^1^H NMR, ^13^C NMR, and HRMS.
