# Supplementary material for: Merging enzymes with chemocatalysis for amide bond synthesis
Source: Nat Commun. 2022 Jan 19;13:380. doi: 10.1038/s41467-022-28005-4 (PMC8770729; doi:10.1038/s41467-022-28005-4)
Supplement: Supplementary file 1 — Supporting Information [file 41467_2022_28005_MOESM1_ESM.pdf]

Supplementary Information for

Merging Enzymes with Chemocatalysis for Amide Bond Synthesis

Luis Bering, Elliott J. Craven, Stanley A. Sowerby Thomas, Sarah A. Shepherd & Jason Micklefield*

Department of Chemistry and Manchester Institute of Biotechnology, The University of Manchester, 131 Princess Street, Manchester M1 7DN, UK.

*Corresponding author Email: Jason.micklefield@manchester.ac.uk

Table of Contents

Supplementary Notes 2

Supplementary Tables 4

Supplementary Methods 7

Nitrile Hydratase Protein Sequences 7

NHase DNA sequences 11

NHase Cloning and Expression 18

Characterisation of Amides Products from Integrated Reactions 21

Preparation of Starting Materials 48

Preparation of Racemic Standards 59

Preparation of Ligands L10 and L11 64

Determination of Configuration of Chiral Amide 55 66

Procedure for NHase Recycling Experiment 70

NMR spectra and HPLC traces 71

Supplementary References 169

Supplementary Notes

Chemicals were purchased from Sigma-Aldrich, Arcos Organics, Fluorochem, Fisher Scientific UK or Alfa Aesar and used without further purification unless otherwise stated. Solvents for chromatography were laboratory grade. Analytical thin-layer chromatography (TLC) was performed on Merck silica gel aluminium plates with F-254 indicator, visualized by irradiation with UV light. Column chromatography was performed using silica gel Merck 60 (particle size 0.040 - 0.063 mm, 60 Å pore size). Chemical yields refer to isolated substances after column chromatography.

HPLC traces were recorded on either a Shimadzu UFLC XR and analysed using Shimadzu LabSolutions Lite v5 or on an Agilent Technologies 1260 Infinity with spectra analysis processed in Agilent ChemStation Rev. B.04.03.

^{1}H and ^{13}C NMR spectra were recorded at 298K unless otherwise stated and are reported relative to residual solvent peaks. ^{1}H-NMR and ^{13}C-NMR were recorded on either a Bruker Avance I (400 MHz) or a Bruker Avance (500 MHz) spectrometer in $CDCl_3$, MeOD or DMSO-d_6. Unless otherwise stated, spectra are reported relative to residual solvent using iconNMR and TopSpin v3.5pl7. NMR data was processed using MestReNova v11 software. Data are reported in the following order: chemical shift (δ) in ppm; multiplicities are indicated s (singlet), d (doublet), t (triplet), q (quartet), m (multiplet); coupling constants (*J*) are given in Hertz (Hz). HPLC traces were recorded on a

The enantiomeric excess was determined by HPCL analysis using chiral stationary phase columns (column: *i*-amylose 4.6 x 250mm column or *i*-cellulose 4.6 x 250mm column, eluent: *n*-hexane / *i*PrOH). The chiral HPLC methods were calibrated with the corresponding racemic mixtures. HPLC traces were recorded on an Agilent Technologies 1260 Infinity with spectra analysis processed in Agilent ChemStation Rev. B.04.03.

High resolution mass spectra were recorded on Agilent Q-TOF LC-MS or ThermoFisher LCQ Orbitrap XL and all mass spec data were processed using Mass Hunter Workstation Software Qualitative Analysis B.06.00.

Cell suspension OD_{600} values were determined on an Agilent Technologies Cary 60 UV-Vis spectrometer using Cary WinUV Simple Reads Application software v5.0.0.999. Cell suspensions were diluted 1:10 with 100 mM KPi buffer (pH = 7.8) prior to measurement.

Molecular modelling was performed with *Molecular Operating Environment (MOE), 2019.01* (Chemical Computing Group ULC, 1010 Sherbooke St. West, Suite #910, Montreal, QC, Canada, H3A 2R7, 2021) using the minimise and Quick Prep functions. Prepared ligands were docked into the AJ270 crystal structure (PDB code: 2QDY) in MOE (2019.0101, Chemical Computing Group) using Alpha PMI for placement (London dG, 30 poses) and Induced Fit (GBVI/WSA dG, 5 poses) for refinement. Molecular graphics images were produced using the UCSF Chimera package from the Resource for Biocomputing, Visualization, and Informatics at the University of California, San Francisco.

Supplementary Tables

Supplementary Table 1. Initial results for the Cu-catalysed Ullmann-type arylation in aqueous buffer. [a]

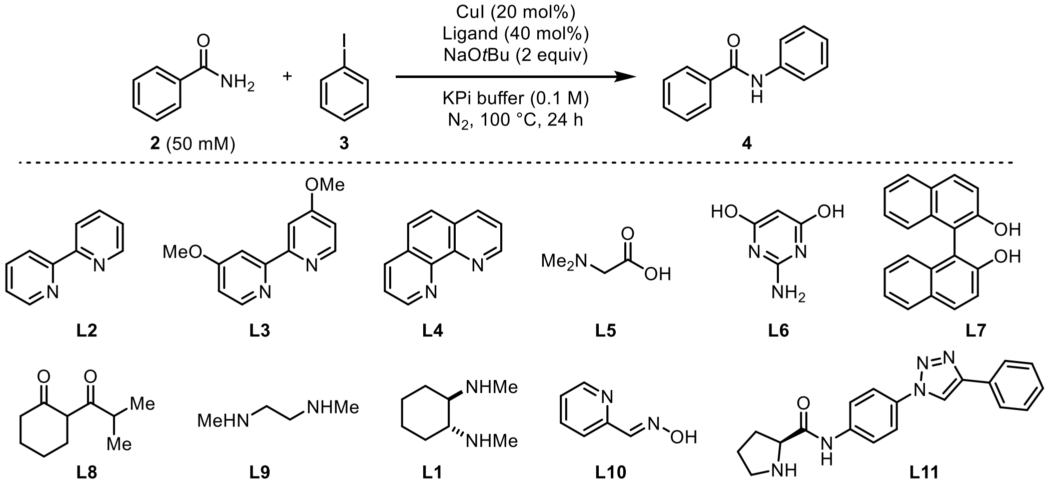

Entry	Ligand	Conversion / % [b]
1	**L2**	trace
2	**L3**	2
3	**L4**	n.d.
4	**L5**	trace
5	**L6**	trace
6	**L7**	trace
7	**L8**	n.d.
8	**L9**	10
9	**L1**	40
**10** [c]	**L1**	**45 (48)**
11	**L10**	n.d.
12	**L11**	5
13	**w/o**	n.d.

[a] Reaction conditions: **2** (12 mg, 0.1 mmol, 1 equiv), **3** (33.5 µL, 0.3 mmol, 3 equiv), CuI (4 mg, 0.02 mmol, 0.2 equiv), ligand (0.4 mmol, 0.4 equiv), NaO*t*Bu (20 mg, 0.2 mmol, 2 equiv) in 0.1 M KPi buffer (2 mL) under N_2 atmosphere. [b] Conversion was determined by HPLC/UV using benzophenone as an external standard. Data are presented as single measurements (n = 1). Yield in parentheses refers to isolated product after column chromatography. [c] Reaction conducted at 50 °C. Abbreviation: n.d. = not detected; w/o = without. **L10** and **L11** were synthesised according to literature procedures.[1, 2]

Supplementary Table 2. Systematic optimisation of the Cu-catalysed arylation of benzamide. [a]

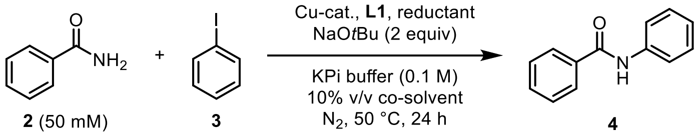

Entry	Cu-cat. / x mol%	Reductant / mol%	Co-solvent	Variation	Conversion / % [b]
1	CuI (20)	-	*i*PrOH	-	55
2	CuI (20)	-	DMSO	-	50
3	CuI (20)	-	MeOH	-	40
4	$CuCl_2$ (20)	D-glu (40)	*i*PrOH	-	75
5	$CuBr_2$ (20)	D-glu (40)	*i*PrOH	-	81 (83)
6	$Cu(OAc)_2$ (20)	D-glu (40)	*i*PrOH	-	77
7	$CuBr_2$ (5)	D-glu (10)	*i*PrOH	-	27
9	$CuBr_2$ (10)	D-glu (20)	*i*PrOH	-	81
10	$CuBr_2$ (40)	D-glu (80)	*i*PrOH	-	80
**11**	**$CuBr_2$ (10)**	**D-glu (20)**	*i*PrOH	**headspace purge**	**87 (88)**
12	$CuBr_2$ (10)	D-glu (20)	*i*PrOH	air atmosphere	85
13	$CuBr_2$ (10)	D-glu (20)	*i*PrOH	RT	47

[a] Reaction conditions: **2** (12 mg, 0.1 mmol, 1 equiv), 3a (33.5 µL, 0.3 mmol, 3 equiv), Cu-cat. (see table), **L1** (2*x mol%), D-glu (see table), NaOtBu (20 mg, 0.2 mmol, 2 equiv) in 0.1 M KPi buffer (2 mL) under N_2 atmosphere. [b] Conversion was determined by HPLC/UV using benzophenone as an external standard. Data are presented as single measurements (n = 1). Yields in parentheses refers to isolated product after column chromatography.

Supplementary Table 3. Further screening for the Cu-catalysed arylation of benzamide. [a]

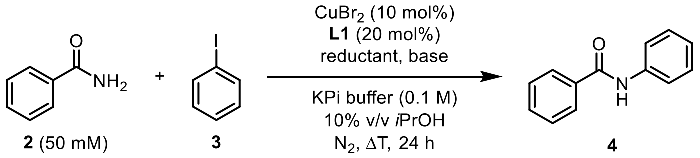

Entry	Base	Reductant /x mol%	Temperature	Halide /x equiv	Variation	Conversion / % [b]
1	KOH	D-glu (20)	50 °C	3	-	67
2	Cs_2CO_3	D-glu (20)	50 °C	3	-	42
3	K_2CO_3	D-glu (20)	50 °C	3	-	34
4	K_3PO_4	D-glu (20)	50 °C	3	-	27
5	NaO*t*Bu	D-glu (10)	50 °C	3	-	73
6	NaO*t*Bu	D-glu (50)	50 °C	3	-	53
7	NaO*t*Bu	D-glu (100)	50 °C	3	-	43
9	NaO*t*Bu	NaAsc (10)	50 °C	3	-	66 (69)
10	NaO*t*Bu	NaAsc (20)	50 °C	3	-	77
11	NaO*t*Bu	NaAsc (50)	50 °C	3	-	77
12	NaO*t*Bu	NaAsc (100)	50 °C	3	-	56
13	NaO*t*Bu	D-glu (20)	RT	3	-	47
14	NaO*t*Bu	D-glu (20)	100 °C	3	-	73
15	NaO*t*Bu	D-glu (20)	50 °C	4	-	73
16	NaO*t*Bu	D-glu (20)	50 °C	2.5	-	52
17	NaO*t*Bu	D-glu (20)	50 °C	2	-	36
18	NaO*t*Bu	D-glu (20)	50 °C	1.5	-	36
19	NaO*t*Bu	D-glu (20)	50 °C	3	+ 2 wt% TPGS-750 M	65 (70)
20	NaO*t*Bu	D-glu (20)	50 °C	4	+ 2 wt% TPGS-750 M	65
21	NaO*t*Bu	D-glu (20)	50 °C	5	+ 2 wt% TPGS-750 M	70

[a] Reaction conditions: **2** (12 mg, 0.1 mmol, 1 equiv), **3** (see table), $CuBr_2$ (2.5 mg, 0.02 mmol, 0.1 equiv), **L1** (3.2 µL, 0.04 mmol, 0.2 equiv), D-glucose (3.5 mg, 0.04 mmol, 0.2 equiv), base (2 equiv) in 0.1 M KPi buffer/ 10% v/v *i*PrOH (2 mL) under N_2 atmosphere (headspace purge). [b] Conversion was determined by HPLC/UV using benzophenone as an external standard. Data are presented as single measurements (n = 1). Yields in parentheses refers to isolated product after column chromatography.

Supplementary Methods

Nitrile Hydratase Protein Sequences

Nitrile hydratase (NHase) from *Rhodococcus hoagii* Equi_NHase

α subunit (WP_084957663.1)

MSVLIDHAKHTGVPGVPEQAPARDRAWALYEALKSKGAVPDGYVEGWKKTFEEDF
TPRKGAELVARAWTDPEFRELLLTDGTAAVAQYGWLGPQGEYIVALEDTPTLKNVI
VCSLCSCTAWPILGLPPTWYKSFEYRARVVREPRKVLEEMGTTLPADTKIRVVDTTA
ETRYLVIPVRPEGTEGWTAEQLQEIVTKDCLIGVAVPQVP

β subunit (WP_084957665.1)

MDGVHDLGGRQGFARVDHKINEYEHGQTYSDPFHADWEHLPYSLFFLGVAELGKFS
VDEVRHVVERIAPVHYLATPYYERYVIGVATLMVETGVLTREDLESFAAGPFPLALP
AASPGRPARADKQQFNVGDRVHVRDEFFAGHIRMPAYCRGRTGVIRHRTADEWPFP
DSIGHGRNDGGAEPTYHVEFTGEELFGDDTDAASILVDLFEGYLEPAA

Activator Protein (WP_084957673.1)

MTDTRLPVTVLSGFLGAGKTTLLNQILRNREGRRVAVIVNDMSEINIDSAEVEREISLS
RSQEKLVEMANGCICCTLREDLLAEIGRLAADGRFDYLIIESSGISEPLPVAETFTFIDA
DGRALADRARLDTMVTVVDGHSFLRDFRSGGAVDADEPGDQRDISDLLVDQIEFAD
VILVSKTDLISDSQLVELEAVVRALNPMARILPMTRIPLETILNTELFSLEKAAQAPGW
LQELRGDHTPETEEYGIGSTVYRERAPFHPQRLHDLLTAEWTNGKLLRAKGYFWNA
ARYTEIGSISQAGHIIRHGYIGRWWRFLPDNLWPADDHRRDGILGQWEEPVGDCRQE
LVFIGQNIDPETLRRQLDACLLTTAEIQQGPDAWSTWPDPLGPGHSDSVTNPLAQTNS
ATQPSDA

NHase from *Rhodococcus erythropolis* AJ270 (CAC08205/6)

α subunit (Q7AZY7)

MSVTIDHTTENAAPAQAPVSDRAWALFRALDGKGLVPDGYVEGWKKTFEEDFSPRR
GAELVARAWTDPEFRQLLLTDGTAAVAQYGYLGPQGEYIVAVEDTPTLKNVIVCSLCS

CTAWPILGLPPTWYKSFEYRARVVREPRKVLSEMGTEIASDIEIRVYDTTAETRYMVLP
QRPAGTEGWSQEQLQEIVTKDCLIGVAIPQVPTV

β subunit (Q7AZY6)

MDGVHDLAGVQGFGKVPHTVNADIGPTFHAEWEHLPYSLMFAGVAELGAFSVDEVR
YVVERMEPRHYMMTPYYERYVIGVATLMVEKGILTQDELESLAGGPFPLSRPSESEGR
PAPVETTTFEVGQRVRVRDEYVPGHIRMPAYCRGRVGTISHRTTEKWPFPDAIGHGRN
DAGEEPTYHVKFAAEELFGSDTDGGSVVVDLFEGYLEPAA

Activator Protein (Q9EV63)

MVDTRLPVTVLSGFLGAGKTTLLNEILRNREGRRVAVIVNDMSEINIDSAEVEREISLS
RSEEKLVEMTNGCICCTLREDLLSEISALAAAGRFDYLLIESSGISEPLPVAETFTFIDTD
GHALADVARLDTMVTVVDGNSFLRDYTAGGRVEADAPEDERDIADLLVDQIEFADVI
LVSKADLISHQHLVELTAVLRSLNATAAIVPMTLGRIPLDTILDTGLFSLEKAAQAPGW
LQELQGEHTPETEEYGISSVVYRERAPFHPQRLHDFLSSEWTNGKLLRAKGYYWNAG
RFTEIGSISQAGHLIRHGYVGRWWKFLPRDEWPADDYRRDGILDKWEEPVGDCRQEL
VFIGQAIDPSLLHRELDACLLTTAEIELGPDVWTTWSDPLGAGYTDQTV

NHase from *Rhodopseudomanas palustris* HaA2 (YP_486317/8)

α subunit (Q2IWK4)

MLRRMSEHQHGHSHDHDHDHSELSETELRVRALETILTEKGYVDPAALDILIETYETK
VGPRNGARVVAKAWTDPAYRARLLQDATAAIAELGYTGRQGEHIVAVENTPATHNMV
VCTLCSCYPWPVLGLPPVWYKSAPYRSRAVKEPRAVLADFGVTLPDDTAIRVWDSTA
EIRYLVIPMRPDGTEGFTEDQLADLVTRDSMIGTGVAQPPAENS

β subunit (Q2IWK3)

MDGAHDMGGMDGFGPVVPEANEPLFHAAWERRAFALTLAMARPGGWNLDMTRFA
RENRSPLDYLSKSYYQIWLAGLERLMAERGLVTSDEIAAARPLHPRRDVAALSADDA
APMLARGAPTERPAPAPAHFAIGDRVRARNIHPHTHTRLPRYVRGHVGVVELVHGAHI
FPDSHALGAGEQPQWLYTVTFDGRELWGDDCDPSLRISVDAWESYLEPVR

Activator Protein (WP_011441593.1)

MTADAAAVAARSVPGLPRDDDGPVFREPWEAHAFALAVTLHARGLFTWPEWAAALA
DEITRAQQRGDPDDGHTYYQHWLATLERLIADKGVASAATQARYRDAWDHAADRTP
HGRPIMLQPDDFAATP

NHase from *Rhodopseudomanas palustris* CGA009 (NP_948148/9)

α subunit (Q6N613)

MADHEHHHHHHHDHDHSELSETELRVRALETILTEKGYVDPAALDELIETYETKVGP
RNGAQVVAKAWSDPAYRERLLHDATAAIAELGYTGRQGEHIVAVENTPATHNMVVCT
LCSCYPWPVLGLPPVWYKSAPYRSRVVKEPRAVLAEFGVTLPQDTAIRVWDSTAEIRY
LVLPMRPEGSDDFSEGQLADLVTRDSMIGTGLARTPAEIPA

β subunit (Q6N612)

MNGAHDMGGMDGFGPIVIEPHEPLFHAAWERRAFALTLAMGRPGGWNIDMSRFARE
NRAPIDYLSKSYYQLWLAGLEVLMAERGLVTADEIAAGRPLHPRRDVQVLTAADVTP
MLARGAPTERDAAAPARFGVGDHVRAKDLHPRTHIRLPRYVRGRIGTIELVHGAHVF
PDSHAHGGGEQPQWLYTVAFEARELWGDDADPTSRVSVDAWDSYLERA

Activator Protein (WP_085977303.1)

MMSSQSEAAAIAAHSVPGLPRDDDGPVFREPWEAHAFAMAVTLHGRGLFTWPEWAA
ALSAEIRRAQADGDPDCGDTYYRHWLATLEQMVATKGVASLATQHRYRDAWDRAA
DRTPHGRPIELQPGDFPQSEAFSAAESPPR

NHase from *Sinorhizobium meliloti* 1021 (NP_386211/2)

α subunit (WP_003533852.1)

MSEHHHGHGDDHGHHHDNHLTDMEARVKALETVLTEKGLIDPAAIDAIVDAYETKV
GPRNGARVVAKAWSDPGFADWLKRDATAAIASLGFTGRQGEHMRAVFNTSETHNLIV
CTLCSCYPWAVLGLPPVWYKAPPYRSRAVIDPRGVLAEFGLELSAEKKVRVWDSTAE
LRYLVVPERPEGTDGFGEDALAELVTRDSMIGTGLALSPEDVR

β subunit (Q92NS2)

MNGPHDLGGQHGMGPIAPERNEPIFHAEWEKRALGITLSCGAFGAWTLDESRHARES
LAPATYLSASYYEIWTRALETLLKRHGFVTQAELDAGHMLDKGREPKRVLTADMVA
GVLAKGGPCDRPVEAPPRFAAGDSVRTKNFNPESHTRLPRYARARTGMVEAVQGSFV
FPDDNAHGKGENPQWLYMVVFDAGEIWGEGADPTLTVSIDAWESYLEHA

Activator Protein (Q92NS3)

MNTLRSTPALPRSPLLASAELPKSREGDPVFAEPWQAVAFAMTVRLHEQGVFSWSEW
AEALSAELYKPGRRADGTDYYDCWVAALSRLVTELSIASGPELEALVGSWQRAAEAT
PHGTPIALANDPLR

NHase DNA sequences

***Rhodococcus equi* TG328-2**

The Equi_NHase genes from *Rhodococcus equi* TG328-2 were provided by *Bornscheuer et al.*[3]. The DNA sequence is:

>Equi_NHAse

α subunit:

ATGTCCGTACTGATCGATCACGCCAAGCACACCGGCGTCCCGGGTGTTCCCGAAC
AGGCCCCCGCGCGCGACCGCGCGTGGGCGCTCTACGAAGCACTCAAGAGCAAGG
GCGCTGTCCCCGACGGCTACGTCGAAGGCTGGAAGAAGACCTTCGAAGAGGACT
TCACCCCGCGCAAGGGCGCCGAACTCGTTGCACGCGCCTGGACGGACCCCGAGTT
CCGTGAGCTACTCCTGACCGACGGCACCGCCGCCGTTGCCCAGTACGGGTGGCTG
GGTCCGCAGGGCGAGTACATCGTGGCGCTCGAGGACACTCCGACGCTGAAGAAC
GTCATCGTCTGCTCCCTCTGCTCGTGCACAGCATGGCCGATCCTCGGTCTGCCCCC
GACGTGGTACAAGAGCTTCGAGTACCGCGCCCGCGTCGTCCGAGAGCCGCGCAA
GGTGCTCGAAGAGATGGGCACCACCCTGCCGGCAGACACGAAGATCCGAGTTGT
CGACACCACTGCCGAGACCCGGTACCTGGTGATCCCCGTGCGCCCGGAAGGCACC
GAAGGCTGGACGGCCGAGCAGCTGCAGGAGATCGTGACCAAGGACTGCCTGATC
GGCGTTGCCGTCCCCCAGGTGCCGTAG

β subunit:

ATGGATGGAGTACACGACCTCGGAGGCAGACAGGGCTTCGCCCGGGTCGACCAC
AAGATCAACGAGTACGAGCACGGCCAGACGTACTCGGACCCGTTCCACGCAGAC
TGGGAGCACCTTCCCTACAGCCTGTTCTTCCTCGGCGTCGCCGAGCTGGGCAAGT
TCAGTGTCGACGAGGTCCGTCACGTCGTCGAACGCATCGCCCCTGTGCACTACCT
CGCCACCCCGTACTACGAACGTTACGTCATCGGTGTCGCGACGCTCATGGTCGAA
ACCGGTGTCCTGACCCGGGAAGACCTCGAGTCCTTCGCCGCAGGGCCGTTCCCGT
TGGCATTGCCGGCCGCCTCGCCGGGTCGACCGGCGCGTGCTGACAAGCAGCAGTT
CAACGTCGGCGACCGGGTGCACGTGCGTGACGAGTTCTTCGCCGGACACATTCGG
ATGCCTGCGTACTGCCGCGGTCGAACGGGCGTGATCCGTCACCGCACCGCGGACG
AGTGGCCCTTCCCGGACTCGATCGGCCACGGACGCAACGACGGAGGCGCCGAGC
CGACCTACCACGTCGAGTTCACCGGCGAGGAGCTCTTCGGCGACGACACGGATGC
AGCGAGCATCCTGGTCGACCTCTTCGAGGGATACCTCGAGCCGGCCGCCTGA

Activator Protein:

ATGACCGATACCAGGCTTCCGGTCACAGTGCTCTCAGGCTTCCTCGGGGCGGGCA
AGACCACCCTTCTCAATCAGATTCTCCGTAACCGTGAGGGCCGCCGCGTCGCTGT
CATCGTCAACGACATGAGCGAAATCAATATCGACAGTGCCGAAGTCGAACGCGA
GATATCCCTGAGCCGCTCTCAGGAGAAGCTCGTCGAGATGACCAACGGCTGCATC
TGCTGCACCTTGCGGGAAGACCTCCTCGCAGAAATCGGAAGGCTCGCCGCCGACG
GACGGTTCGACTATCTGATCATCGAGTCGTCCGGCATCTCCGAGCCCCTTCCGGTT
GCTGAGACGTTCACCTTCATCGACGCCGACGGCAGGGCACTCGCCGATCGTGCCA
GGCTGGACACAATGGTCACCGTCGTCGACGGACACAGCTTCCTCCGGGACTTCCG
GTCGGGTGGCGCGGTGGATGCCGATGAACCGGAGGACCAGCGCGACATTTCCGA
CCTATTGGTCGACCAGATCGAGTTCGCCGACGTGATCCTGGTCAGCAAAACCGAT
CTGATATCCGACAGCCAACTGGTGGAGCTTGAAGCCGTGGTCAGGGCGCTGAACC
CGATGGCCCGGATCCTGCCGATGACGAACGGAGAAGTCCCTCTCGAAACAATCCT
GAACACCGAACTGTTCAGTCTCGAGAAGGCGGCGCAGGCACCGGGGTGGTTGCA
GGAGCTTCGCGGTGATCACACGCCCGAAACGGAGGAGTACGGGATCGGCTCCAC
CGTTTACCGCGAGCGGGCACCATTCCACCCACAGCGACTGCACGATCTGCTCACC
GCCGAATGGACGAACGGAAAACTACTGCGCGCCAAAGGATACTTCTGGAACGCC
GCCCGATACACCGAGATCGGCAGTATCTCGCAGGCCGGCCACATCATCCGACACG
GGTACATCGGCCGGTGGTGGAGATTCTTGCCCGACAACCTTTGGCCGGCCGACGA
CCACCGCCGTGACGGAATTCTGGGTCAGTGGGAGGAACCCGTCGGAGACTGCCG
TCAGGAACTGGTCTTCATCGGCCAGAACATCGACCCCGAAACCCTGCGGCGGCAA
CTCGACGCATGCCTGCTCACCACCGCCGAAATTCAACAAGGTCCCGACGCGTGGA
GCACCTGGCCCGACCCCCTCGGCCCCGGCCACAGCGACTCGGTCACCAACCCACT
CGCCCAAACCAACTCGGCCACCCAACCGTCCGACGCCTGA

The HaA2 gene from *Rhodopseudomonas palustris* HaA2 was codon optimised for *Escherichia coli* (*E. coli*) and synthesised by Twist Bioscience. The optimised DNA sequence is:

>HaA2

ATGCTGCGCCGTATGTCCGAGCACCAACATGGACACTCACATGACCACGACCATG
ATCATTCGGAACTGTCAGAAACCGAGTTACGTGTACGTGCTCTGGAGACTATTTT
GACGGAAAAGGGTTACGTAGATCCCGCGGCCCTTGATATTTTAATTGAAACATAC

GAGACAAAAGTAGGCCCTCGTAATGGAGCACGTGTTGTAGCAAAAGCGTGGACT
GATCCGGCATATCGTGCTCGCTTGTTACAGGATGCAACGGCTGCAATTGCAGAGC
TGGGTTATACAGGTCGTCAAGGTGAACACATTGTTGCGGTGGAAAATACCCCGGC
TACTCATAACATGGTTGTCTGTACCTTGTGTAGCTGTTACCCATGGCCTGTACTGG
GATTACCACCCGTATGGTATAAGAGCGCACCTTACCGTAGTCGTGCCGTTAAAGA
ACCACGTGCTGTTCTCGCTGACTTTGGGGTTACGTTACCGGATGACACTGCTATTC
GCGTCTGGGACTCCACGGCCGAAATTCGTTACCTTGTTATCCCCATGCGCCCGGA
TGGAACCGAGGGATTTACGGAAGATCAGTTGGCGGACCTTGTGACGCGTGATAGT
ATGATTGGTACGGGTGTAGCTCAACCCCCTGCCGAAAACAGCTAATTAAGAAGG
AGATATACCATGGACGGAGCACATGATATGGGTGGTATGGACGGCTTTGGTCCTG
TCGTCCCAGAAGCAAATGAACCACTCTTCCATGCTGCGTGGGAACGTCGTGCATT
TGCTTTAACCCTTGCCATGGCACGCCCGGGTGGATGGAACCTGGATATGACCCGT
TTTGCTCGTGAAAACCGTTCACCGCTCGATTACCTCAGCAAATCATATTATCAAAT
TTGGTTGGCAGGACTGGAGCGCCTCATGGCCGAGCGCGGCCTTGTTACCTCTGAT
GAAATTGCGGCAGCACGCCCCTTGCACCCCCGTCGTGATGTTGCTGCGCTTTCCG
CAGATGACGCAGCACCAATGTTAGCACGTGGAGCCCCTACGGAGCGTCCTGCACC
AGCTCCAGCGCACTTTGCAATTGGGGATCGTGTGCGTGCACGTAATATTCATCCA
CATACGCACACTCGCTTACCCCGTTACGTTCGTGGTCACGTAGGTGTAGTGGAAT
TAGTTCACGGAGCACACATCTTCCCAGATAGTCATGCCCTTGGAGCGGGTGAGCA
ACCTCAGTGGTTATATACTGTTACATTTGATGGTCGTGAGCTTTGGGGAGATGACT
GTGACCCATCTTTACGCATTAGCGTGGATGCGTGGGAATCGTACTTGGAACCCGT
ACGCTAAAATAGAAGGAGATATACCATGACCGCGGATGCAGCAGCCGTTGCTGC
ACGCTCAGTTCCCGGTTTGCCCCGTGATGACGATGGTCCTGTATTTCGTGAACCGT
GGGAAGCACATGCATTTGCCCTCGCTGTTACCCTTCACGCCCGTGGTTTATTTACA
TGGCCTGAGTGGGCCGCAGCGCTGGCGGATGAAATTACCCGTGCTCAACAACGC
GGTGACCCTGATGACGGGCATACTTATTATCAACATTGGCTGGCAACCTTAGAGC
GTCTCATTGCAGATAAAGGGGTAGCGTCTGCCGCCACCCAAGCCCGTTACCGTGA
TGCATGGGACCATGCTGCAGATCGTACGCCACATGGTCGTCCTATTATGCTGCAA
CCCGATGACTTCGCTGCCACTCCCTAA

The CGA009 gene from *Rhodopseudomonas palustris* CGA009 was codon optimised for *E. coli* and synthesised by Twist Bioscience. The optimised DNA sequence is:

>CGA009

ATGGCTGATCACGAACACCACCATCATCATCATCATGACCATGATCACTCAGAGC
TCTCAGAGACGGAACTGCGCGTCCGTGCCCTGGAGACGATTCTTACTGAGAAAGG
TTACGTGGACCCGGCTGCCCTTGACGAATTAATTGAAACCTATGAAACTAAAGTG
GGTCCCCGTAATGGTGCGCAGGTAGTTGCAAAAGCGTGGTCGGACCCCGCTTATC
GTGAACGCCTCTTGCATGATGCAACTGCCGCAATTGCCGAACTTGGTTATACAGG
ACGTCAAGGTGAGCATATTGTTGCAGTGGAAAACACACCTGCTACTCATAATATG
GTGGTATGTACACTCTGTAGCTGTTATCCTTGGCCAGTATTGGGTCTTCCACCAGT
GTGGTATAAGAGTGCACCATACCGCAGTCGTGTCGTGAAAGAACCTCGTGCTGTT
CTGGCGGAGTTTGGTGTTACCCTTCCTCAAGATACCGCCATTCGCGTTTGGGACA
GTACGGCAGAAATTCGCTATCTTGTACTGCCAATGCGTCCAGAAGGATCAGATGA
CTTTAGTGAGGGCCAATTAGCGGATCTGGTGACGCGTGATAGTATGATTGGTACG
GGGCTGGCGCGTACCCCCGCCGAAATTCCTGCCTAATTAAGAAGGAGATATACCA
TGAATGGAGCCCATGATATGGGTGGTATGGATGGCTTTGGACCCATCGTAATCGA
ACCGCACGAACCTTTGTTCCACGCCGCGTGGGAACGTCGTGCGTTTGCGTTAACT
CTCGCTATGGGTCGCCCGGGCGGTTGGAATATTGATATGAGCCGTTTTGCACGTG
AAAATCGTGCACCCATTGACTACCTTTCCAAAAGTTATTACCAATTATGGCTTGCA
GGTCTGGAAGTTTTGATGGCGGAACGTGGTCTGGTGACGGCGGATGAAATTGCGG
CTGGCCGTCCTCTTCACCCACGTCGCGATGTGCAAGTATTAACAGCCGCCGATGT
CACACCTATGCTTGCCCGTGGTGCTCCCACGGAGCGTGATGCAGCAGCGCCAGCA
CGTTTTGGTGTGGGAGACCACGTGCGCGCTAAAGATCTCCACCCGCGCACACACA
TTCGTTTACCACGTTATGTTCGTGGTCGCATTGGTACAATTGAGTTGGTGCATGGT
GCTCATGTTTTCCCCGATAGTCATGCTCACGGTGGTGGGGAACAACCACAGTGGC
TGTATACCGTTGCCTTTGAGGCGCGTGAGCTGTGGGGTGATGACGCGGATCCCAC
TTCCCGCGTATCTGTAGACGCGTGGGATAGCTACTTGGAACGTGCCTAAAATAGA
AGGAGATATACCATGTCCAGTCAATCAGAAGCAGCCGCCATTGCGGCCCATTCAG
TCCCTGGTCTTCCTCGTGATGACGATGGTCCAGTCTTTCGTGAACCGTGGGAAGCT
CACGCTTTCGCAATGGCTGTCACCTTGCACGGTCGTGGTTTATTTACTTGGCCGGA
GTGGGCTGCCGCCCTGAGCGCGGAAATCCGTCGCGCTCAAGCGGATGGTGACCC
GGATTGTGGTGATACGTATTACCGTCACTGGCTGGCGACTTTGGAGCAAATGGTT
GCTACGAAAGGGGTAGCATCGCTGGCCACTCAACACCGCTATCGTGATGCTTGGG
ATCGTGCGGCGGACCGTACCCCTCATGGGCGCCCAATTGAACTTCAACCAGGCGA
CTTCCCGCAAAGCGAAGCCTTCTCAGCCGCAGAATCTCCACCCCGCTGA

The AJ270 gene from *Rhodococcus erythropolis* AJ270 was codon optimised for *E. coli* and synthesised by Twist Bioscience. The optimised DNA sequence is:

>AJ270

ATGTCGGTCACCATTGATCATACCACAGAAAATGCTGCGCCTGCTCAAGCCCCTG
TTTCTGATCGCGCCTGGGCGTTGTTTCGTGCGTTAGATGGCAAAGGCCTGGTGCC
GGATGGCTATGTGGAAGGCTGGAAGAAAACGTTTGAAGAAGATTTTAGCCCGCG
CCGTGGTGCCGAGCTGGTCGCTCGTGCGTGGACTGATCCGGAATTTCGTCAATTG
CTCCTGACAGATGGCACTGCGGCTGTGGCTCAATATGGGTATTTGGGACCGCAAG
GTGAGTATATTGTTGCTGTTGAGGATACACCCACTCTGAAGAATGTAATTGTATG
TAGTCTTTGCTCCTGTACGGCATGGCCGATTCTGGGCTTACCGCCAACGTGGTATA
AATCTTTTGAGTATCGCGCACGTGTTGTACGTGAGCCGCGCAAAGTGCTGTCGGA
AATGGGGACTGAAATTGCATCTGATATTGAAATCCGTGTTTATGATACGACGGCG
GAGACCCGTTATATGGTGCTGCCTCAACGCCCTGCGGGAACAGAGGGATGGTCGC
AAGAGCAGCTTCAAGAGATTGTAACAAAAGATTGTCTCATTGGTGTGGCTATTCC
ACAAGTCCCGACGGTATAATTAAGAAGGAGATATACCATGGACGGCGTGCATGA
CTTAGCTGGCGTTCAGGGTTTTGGTAAGGTGCCACACACGGTGAATGCGGATATT
GGTCCGACGTTCCATGCAGAGTGGGAGCATTTGCCTTATTCATTGATGTTTGCTGG
CGTGGCTGAGCTGGGCGCGTTTTCAGTAGATGAGGTACGTTATGTTGTTGAACGT
ATGGAACCACGTCATTATATGATGACGCCTTATTATGAACGTTATGTGATTGGCG
TAGCCACTTTAATGGTAGAGAAAGGTATTCTTACCCAAGATGAGCTTGAGTCGTT
AGCCGGCGGCCCATTTCCTTTGAGCCGTCCTTCAGAGTCGGAAGGTCGCCCAGCC
CCAGTTGAAACAACAACTTTTGAGGTGGGCCAACGTGTCCGTGTGCGTGATGAAT
ATGTGCCCGGTCACATCCGCATGCCGGCCTATTGTCGCGGGCGTGTAGGTACGAT
TAGTCACCGCACGACAGAAAAGTGGCCTTTCCCGGATGCTATTGGACATGGCCGT
AATGATGCAGGGGAAGAGCCTACTTATCATGTAAAATTTGCGGCGGAAGAGTTAT
TTGGCTCGGATACGGATGGCGGCTCCGTGGTGGTTGATTTGTTTGAAGGATATCT
GGAACCGGCTGCATAAAATAGAAGGAGATATACCATGGTAGATACCCGCTTACC
CGTTACCGTTTTATCTGGCTTTCTTGGAGCGGGGAAAACCACTCTCTTAAATGAAA
TTCTCCGTAACCGTGAAGGACGCCGCGTGGCCGTAATTGTGAATGATATGTCTGA
GATTAATATTGATTCCGCCGAGGTTGAACGCGAAATTAGCCTTAGCCGTAGCGAA
GAAAAGTTGGTGGAAATGACTAATGGTTGTATTTGTTGTACCTTGCGCGAAGACT
TGCTGTCTGAAATTTCGGCGTTGGCAGCGGCGGGGCGTTTTGATTATCTGTTGATT

GAGTCATCCGGTATTTCTGAGCCCTTGCCTGTTGCCGAAACCTTTACATTTATTGA
CACTGATGGTCATGCCTTGGCGGATGTTGCGCGCTTAGATACGATGGTGACGGTC
GTGGATGGTAATAGCTTTCTCCGTGATTATACCGCCGGCGGACGTGTTGAGGCGG
ACGCGCCTGAGGACGAGCGCGATATTGCCGACCTGCTGGTGGATCAAATTGAATT
CGCAGATGTAATTTTAGTTTCTAAAGCTGACCTTATTAGCCATCAACATTTGGTGG
AGCTGACAGCTGTTTTGCGTAGCCTTAATGCCACTGCCGCTATCGTGCCTATGACC
CTGGGCCGTATTCCGTTGGATACCATCCTGGATACGGGGCTGTTTAGCCTTGAAA
AGGCCGCGCAAGCGCCGGGTTGGCTTCAGGAGTTACAGGGCGAGCATACCCCAG
AGACTGAGGAATATGGTATTTCTAGCGTTGTATATCGTGAACGTGCGCCGTTTCA
TCCGCAGCGCTTGCACGATTTTCTCAGTTCTGAATGGACTAATGGCAAACTGCTG
CGCGCGAAAGGATATTATTGGAACGCGGGTCGCTTTACGGAAATTGGCAGCATCT
CGCAAGCAGGCCACCTGATCCGTCATGGTTATGTGGGTCGCTGGTGGAAATTCTT
GCCGCGCGATGAATGGCCAGCGGATGACTATCGTCGTGATGGCATTCTGGATAAA
TGGGAAGAGCCGGTTGGAGATTGTCGTCAAGAGCTTGTATTTATTGGACAGGCGA
TTGATCCTAGCCTGCTTCATCGTGAGTTGGATGCCTGCTTGTTGACTACCGCAGAA
ATTGAGTTAGGTCCGGATGTCTGGACAACGTGGAGTGATCCGTTAGGGGCGGGCT
ACACTGATCAAACGGTATAA

The 1021 gene from *Sinorhizobium meliloti* 1021 was codon optimised for *E. coli* and synthesised by Twist Bioscience. The optimised DNA sequence is:

>1021

ATGAGCGAGCACCACCACGGACACGGTGATGACCATGGGCACCATCATGATAAT
CATTTAACAGATATGGAAGCGCGCGTGAAAGCTCTGGAAACCGTTCTTACTGAGA
AAGGCCTTATTGACCCCGCTGCTATTGATGCTATTGTGGATGCCTATGAAACAAA
AGTCGGCCCTCGTAATGGTGCCCGTGTGGTGGCAAAAGCATGGTCGGACCCAGG
ATTCGCAGACTGGTTGAAGCGTGATGCTACTGCCGCTATTGCATCTTTAGGATTCA
CGGGCCGTCAAGGGGAACACATGCGCGCTGTCTTTAATACCAGCGAAACCCATA
ATCTGATTGTGTGCACATTGTGTAGCTGTTACCCGTGGGCGGTTCTTGGCTTGCCG
CCCGTGTGGTATAAAGCCCCGCCATACCGCAGTCGTGCGGTGATTGACCCGCGTG
GTGTTCTGGCGGAGTTTGGTCTGGAACTTAGCGCGGAGAAGAAGGTGCGTGTATG
GGACTCCACTGCGGAACTGCGTTATCTGGTGGTACCTGAACGTCCTGAGGGTACG
GATGGTTTTGGTGAAGATGCATTAGCTGAACTGGTCACCCGTGACTCTATGATTG

GAACGGGCCTGGCGCTCAGTCCAGAAGACGTTCGCTAATTAAGAAGGAGATATA
CCATGAACGGCCCACACGACCTGGGTGGTCAACACGGTATGGGTCCAATTGCCCC
AGAGCGTAACGAACCTATCTTTCACGCGGAATGGGAGAAACGTGCTCTTGGTATT
ACATTGTCTTGCGGGGCATTTGGCGCGTGGACCCTTGATGAGTCCCGTCATGCTC
GTGAGTCTCTCGCACCAGCCACATACCTGTCTGCCTCGTACTACGAGATTTGGAC
CCGCGCATTAGAGACATTACTGAAACGTCACGGTTTTGTAACCCAAGCTGAACTG
GACGCTGGGCACATGTTAGATAAAGGCCGTGAACCAAAGCGCGTCTTAACGGCC
GATATGGTAGCAGGGGTACTGGCGAAAGGTGGACCTTGTGATCGTCCTGTTGAGG
CACCACCACGTTTCGCGGCTGGTGATTCGGTCCGCACCAAGAATTTTAACCCTGA
ATCACATACCCGTTTACCTCGTTATGCACGTGCGCGCACCGGAATGGTAGAAGCG
GTTCAAGGGTCATTTGTTTTCCCAGATGATAATGCGCATGGTAAAGGTGAAAATC
CTCAGTGGCTTTATATGGTTGTGTTTGACGCAGGTGAAATTTGGGGCGAAGGTGC
CGATCCTACCCTGACAGTTAGCATTGACGCTTGGGAATCATACTTAGAGCATGCG
TAAAATAGAAGGAGATATACTATGAACACGCTGCGCTCTACCCCGGCGTTACCTC
GCAGCCCTTTGCTTGCGAGCGCAGAGCTCCCTAAGTCTCGTGAAGGTGATCCGGT
GTTCGCGGAACCGTGGCAAGTAGTGGCGTTTGCAATGACAGTTCGCCTGCACGAA
CAAGGGGTGTTCAGTTGGTCGGAGTGGGCAGAGGCCCTCTCAGCAGAACTGTATA
AACCGGGGCGTCGTGCTGATGGCACTGATTACTATGACTGTTGGGTCGCAGCATT
GAGTCGTTTAGTTACCGAGCTGAGCATTGCGTCGGGCCCTGAGCTCGAAGCATTG
GTGGGTTCCTGGCAACGTGCCGCAGAGGCCACACCCCATGGTACCCCTATCGCTC
TGGCAAATGACCCGTTGCGCTAA

NHase Cloning and Expression

Nitrile hydratase (NHase) cloning and expression was performed according to literature procedures.[3, 4] NHase enzymes from *Rhodopseudomonas palustris* HaA2 (HaA2), *Rhodopseudomonas palustris* CGA009 (CGA009), *Sinorhizobium meliloti* 1021 (1021) and *Rhodococcus erythropolis* AJ270 (AJ270) were codon optimised for heterologous expression in *E. coli*. Synthetic genes encoding the NHase enzymes were purchased from Twist Bioscience and supplied as pET28a (+) constructs with the genes of interest cloned between *NdeI* and *XhoI*.

Nitrile hydratase (Equi_NHase) from *Rhodococcus hoagii* (*Rhodococcus equi* TG328-2) was kindly provided by Uwe T. Bornscheuer's group (Greifswald University, Germany) with constructs containing genes encoding for the α and β subunits being supplied in pET21a (pNHAB4) and activator protein in pET28a (pNHO5).[3]

HaA2, CGA009 and 1021 are cobalt containing nitrile hydratases where as AJ270 and Equi_NHase are iron dependent nitrile hydratases. Each of the nitrile hydratase plasmids were used to transform chemically competent *E. coli* BL21(DE3) cells for protein production. Chemically competent *E.coli* BL21(DE3) cells were co-transformed with both pNHAB4 and pNHO5 plasmids for Equi_NHase protein production. Transformants were selected by growth on LB agar supplemented with kanamycin (50 µg/ml) at 37 °C overnight or kanamycin (50 µg/mL) and ampicillin (100 µg/mL) for Equi_NHase selection. Single colonies of transformant were used to inoculate LB media containing kanamycin (50 µg/ml) or kanamycin (50 µg/mL) and ampicillin (100 µg/mL) for Equi_NHase. After cultivation for overnight at 37 °C, with 180 rpm agitation, the AJ270, CGA009, HaA2 and 1021 cultures were diluted 1:100 in 2YT auto-induction media with kanamycin (50 µg/ml) and incubated at 37 °C with shaking (180 rpm). After incubation for 6 h, $CoCl_2$ (1 mM final concentration) was added to HaA2, CGA009 and 1021 and incubation continued at 18 °C overnight. For Equi_NHase expression, after overnight incubation at 37 °C with 180 rpm agitation, the culture was diluted 1:100 into 2YT media containing kanamycin (50 µg/ml) and ampicillin (100 g/mL) and incubated at 37 °C with shaking (180 rpm). Cells were grown until OD_{600} = 0.8 and the incubation temperature was lowered to 20 °C and further incubated untill OD_{600} = 1.0 was reached. Next, induction was caried out with IPTG (0.1 mM final concentration) and incubation was continued at 20 °C at 180 rpm for 22 hours.

Cells were harvested by centrifugation (2830 g, 20 min, 4 °C), washed with 100 mM KPi buffer, aliquoted (20 mL cell culture aliquots) and centrifuged (2415 g, 15 min, 4 °C) and the resultant cell pellets were then used immediately or stored at -80 °C until use.

Protein Purification Buffers

Buffers containing Tris buffer (50 mM), NaCl (500 mM), imidazole (10 mM, 30 mM or 300 mM) and butyric acid (40 mM) in distilled water were prepared, pH adjusted after addition of imidazole and butyric acid to pH 7.4 and stored at 4 °C overnight before protein purification.

Protein purification

Pellets of *E. coli* expressing NHase were re-suspended in 10 mM imidazole Tris HCl buffer (50 mM, pH 7.5 containing 500 mM NaCl and 40 mM butyric acid) (ca. 50 mL total volume for 10 cm^3 of cells), lysed by sonication (10 min, Pulse: 20 sec on 20 sec off, Amplitude 50%) and the resultant lysate clarified by centrifugation (13750 g, 60 min, 4 °C). Ni-NTA was equilibrated with 10 mM imidazole Tris buffer prior to loading with clarified lysate under gravity flow. The resin was then washed with 30 mM imidazole Tris buffer (3 CV) prior to elution with 300 mM imidazole Tris buffer (5 CV). The eluted fraction was then buffer exchanged into 50 mM Tris-HCl buffer (pH 7.5) containing 500 mM NaCl, butyric acid (40 mM) and glycerol (10% v/v final).

Protein concentration was then determined by using the A_{280} feature of a ThermoScientific 2000 Nanodrop. SDS-Page analysis showed that all proteins were obtained in high purity and reasonable yield.

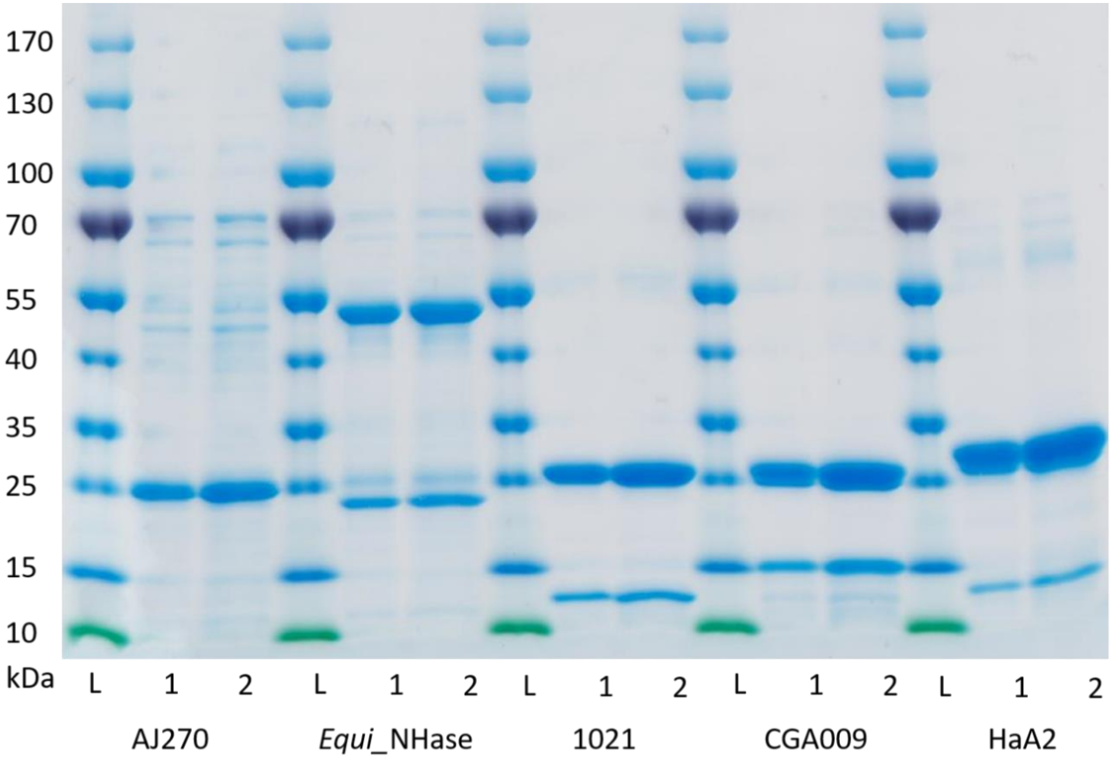

Supplementary Figure 1. SDS-PAGE analysis of the expression of NHases from different nitrile metabolism species. L = Protein ladder, 1 = 5 µL of a 1 mg/mL solution of purified protein, 2 = 10 µL of a 1 mg/mL solution of purified protein.

Characterisation of Amides Products from Integrated Reactions

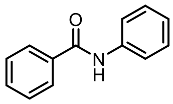

***N*-phenylbenzamide (4).** Prepared according to Method A using benzonitrile (21 µl, 0.2 mmol), iodobenzene (66 µl, 0.6 mmol), and *E. coli* (CGA009) whole cells; the product was obtained as a pale white solid (36 mg, 0.18 mmol, 91%). **^{1}H NMR** (400 MHz, $CDCl_3$) δ 7.93 (s, 1H), 7.86 (d, *J* = 7.4 Hz, 2H), 7.65 (d, *J* = 7.8 Hz, 2H), 7.54 (m, 1H), 7.47 (t, *J* = 7.4 Hz, 2H), 7.36 (t, *J* = 7.7 Hz, 2H), 7.15 ppm (t, *J* = 7.4 Hz, 1H). **^{13}C NMR** (101 MHz, $CDCl_3$) δ 165.91, 138.06, 135.13, 131.96, 129.22, 128.91, 127.16, 124.70, 120.36 ppm. **HR-MS:** calc. for $[M+H]^+$ $C_{13}H_{12}NO$ =198.09134 found 198.09218 (-4.26 ppm).

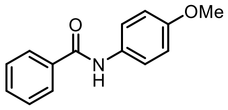

***N*-(4-methoxyphenyl)benzamide (5).** Prepared according to Method A using benzonitrile (21 µl, 0.2 mmol), 4-iodoanisole (140 mg, 0.6 mmol), and *E. coli* (CGA009) whole cells; the product was obtained as a pale white solid (29 mg, 0.13 mmol, 64%). **^{1}H NMR** (400 MHz, DMSO) δ 10.13 (s, 1H), 7.95 (dd, *J* = 8.3, 1.5 Hz, 2H), 7.74 – 7.63 (m, 2H), 7.62 – 7.45 (m, 3H), 6.93 (d, *J* = 9.0 Hz, 2H), 3.75 ppm (s, 3H). **^{13}C NMR** (101 MHz, DMSO) δ 165.09, 155.54, 135.05, 132.23, 131.35, 128.33, 127.53, 121.97, 113.72, 55.17 ppm. **HR-MS:** calc. for $[M+H]^+$ $C_{14}H_{14}NO_2$ = 228.10191 found 228.10276 (-3.76 ppm).

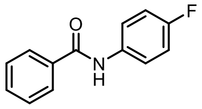

N-(4-fluorophenyl)benzamide (6). Prepared according to Method A using benzonitrile (21 µl, 0.2 mmol), 4-fluoroiodobenzene (69 µl, 0.6 mmol), and *E. coli* (CGA009) whole cells; the product was obtained as a pale white solid (42 mg, 0.194 mmol, 97%) as a white solid. **^{1}H NMR** (400 MHz, DMSO) δ 10.30 (s, 1H), 7.99 – 7.90 (m, 2H), 7.84 – 7.73 (m, 2H), 7.58 (dd, *J* = 5.1, 3.6 Hz, 1H), 7.57 – 7.48 (m, 2H), 7.25 – 7.13 ppm (m, 2H). **^{13}C NMR** (101 MHz,

DMSO) δ 165.47, 158.28 (d, J_{CF} = 240.1 Hz), 135.52 (d, J_{CF} = 2.5 Hz), 134.80, 131.60, 128.41, 127.62, 122.17 (d, J_{CF} = 7.8 Hz), 115.18 ppm (d, J = 22.1 Hz). **HR-MS:** calc. for $[M+H]^+$ $C_{13}H_{11}FNO$ = 216.08192 found 216.08265 (-3.4 ppm).

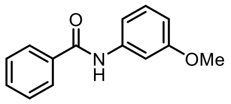

***N*-(3-methoxyphenyl)benzamide (7).** Prepared according to Method A using benzonitrile (21 µl, 0.2 mmol), 3-iodoanisole (71 µl, 0.6 mmol), and *E. coli* (CGA009) whole cells; the product was obtained as a white solid (44 mg (0.192 mmol, 96%). **^{1}H NMR** (400 MHz, $CDCl_3$) δ 7.79 (s, 1H), 7.68 (d, J = 7.3 Hz, 2H), 7.36 (t, J = 7.3 Hz, 1H), 7.32 – 7.22 (m, 3H), 7.07 (t, J = 8.0 Hz, 1H), 6.94 (m, 1H), 6.57 – 6.49 (m, 1H), 3.64 ppm (s, 3H). **^{13}C NMR** (101 MHz, $CDCl_3$) δ 165.95, 160.32, 139.31, 135.07, 131.96, 129.85, 128.88, 127.13, 112.45, 110.67, 105.95, 55.44 ppm. **HR-MS:** calc. for $[M+H]^+$ $C_{14}H_{14}NO_2$ = 228.10191 found 228.10277 (-3.81 ppm).

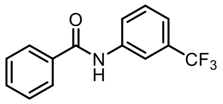

***N*-(3-(trifluoromethyl)phenyl)benzamide (8).** Prepared according to Method A using benzonitrile (21 µl, 0.2 mmol), 3-iodobenzotrifluoride (86 µl, 0.6 mmol), and *E. coli* (CGA009) whole cells; the product was obtained as a pale yellow solid (26 mg, 0.1 mmol. 49%). **^{1}H NMR** (400 MHz, DMSO) δ 10.55 (s, 1H), 8.26 (t, J = 2.0 Hz, 1H), 8.06 (dd, J = 8.2, 2.0 Hz, 1H), 8.01 – 7.94 (m, 2H), 7.65 – 7.52 (m, 4H), 7.48 – 7.42 ppm (m, 1H). **^{13}C NMR** (101 MHz, DMSO) δ 165.93, 139.96, 134.42, 131.90, 129.86, 129.50, 129.18, 128.47, 127.71, 123.75, 119.93 (d, J_{CF} = 4.0 Hz), 116.32 ppm (d, J_{CF} = 4.2 Hz). **HR-MS:** calc. for $[M+H]^+$ $C_{14}H_{11}F_3NO$ = 266.07873 found 266.07989 (-4.4 ppm).

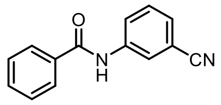

***N*-(3-cyanophenyl)benzamide (9).** Prepared according to Method A using benzonitrile (21 µl, 0.2 mmol), 3-iodobenzonitrile (137 mg, 0.6 mmol), and *E. coli* (CGA009) whole cells; the product was obtained as a pale yellow solid (38 mg, 0.17 mmol, 86%). **^{1}H NMR** (400 MHz, DMSO) δ 10.56 (s, 1H), 8.26 (s, 1H), 8.09 – 8.01 (m, 1H), 8.00 – 7.92 (m, 2H), 7.66 – 7.50 ppm (m, 5H). **^{13}C NMR** (101 MHz, DMSO) δ 165.97, 139.99, 134.31, 131.97, 130.13, 128.49, 127.73, 127.14, 124.82, 122.96, 118.73, 111.45 ppm. **HR-MS:** calc. for $[M+H]^+$ $C_{14}H_{11}N_2O$ = 223.08659 found 223.08755 (-4.33 ppm).

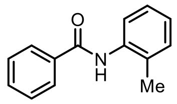

***N*-(*o*-tolyl)benzamide (10).** Prepared according to Method A using benzonitrile (21 µl, 0.2 mmol), 2-iodotoluene (76 µL, 0.6 mmol), and *E. coli* (CGA009) whole cells; the product was obtained as a white solid (40 mg, 0.19 mmol, 94%). **^{1}H NMR** (400 MHz, $CDCl_3$) δ 7.92 (d, *J* = 7.8 Hz, 1H), 7.87 (d, *J* = 7.3 Hz, 2H), 7.68 (s, 1H), 7.55 (t, *J* = 7.2 Hz, 1H), 7.49 (d, *J* = 7.5 Hz, 2H), 7.26 – 7.17 (m, 2H), 7.10 (t, *J* = 7.4 Hz, 1H), 2.32 ppm (s, 3H). **^{13}C NMR** (101 MHz, $CDCl_3$) δ 165.78, 135.91, 135.15, 131.98, 130.69, 129.36, 128.97, 127.18, 127.04, 125.49, 123.25, 17.98 ppm. **HR-MS:** calc. for $[M+H]^+$ $C_{14}H_{14}NO$ = 212.10699 found 212.10774 (-3.55 ppm).

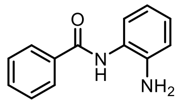

***N*-(2-aminophenyl)benzamide (11).** Prepared according to Method A using benzonitrile (21 µl, 0.2 mmol), 2-iodoaniline (131 mg, 0.6 mmol), and *E. coli* (CGA009) whole cells; the product was obtained as a white solid (22 mg, 0.1 mmol, 52%). **^{1}H NMR** (400 MHz, DMSO) δ 9.66 (s, 1H), 7.99 (d, *J* = 7.3 Hz, 2H), 7.54 (m, 3H), 7.17 (d, *J* = 7.6 Hz, 1H), 6.98 (m, 1H), 6.79 (dd, *J* = 8.0, 1.4 Hz, 1H), 6.60 (m, 1H), 4.90 ppm (s, 2H). **^{13}C NMR** (101 MHz, DMSO)

δ 165.30, 143.16, 134.62, 131.36, 128.27, 127.74, 126.70, 126.48, 123.29, 116.23, 116.10 ppm. **HR-MS:** calc. for $[M+H]^+$ $C_{13}H_{13}N_2O$ = 213.10224 found 213.10288 (-3.02 ppm).

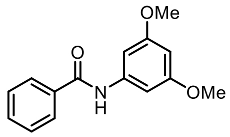

***N*-(3,5-dimethoxyphenyl)benzamide (12).** Prepared according to Method A using benzonitrile (63 µl, 0.6 mmol), 3,5-dimethoxy-iodobenzene (52 mg, 0.2 mmol), and *E. coli* (CGA009) whole cells; the product was obtained as a pale yellow solid (22 mg, 0.09 mmol, 43%). **^{1}H NMR** (400 MHz, $CDCl_3$) δ 7.85 (d, *J* = 7.3 Hz, 2H), 7.80 (s, 1H), 7.55 (t, *J* = 7.4 Hz, 1H), 7.48 (t, *J* = 7.4 Hz, 2H), 6.90 (d, *J* = 2.1 Hz, 2H), 6.28 (t, *J* = 2.1 Hz, 1H), 3.80 ppm (s, 6H). **^{13}C NMR** (101 MHz, $CDCl_3$) δ 165.86, 161.26, 139.84, 135.10, 132.05, 128.96, 127.10, 98.44, 97.21, 55.58 ppm. **HR-MS:** calc. for $[M+H]^+$ $C_{15}H_{16}NO_3$ = 258.11247 found 258.11298 (-1.98 ppm).

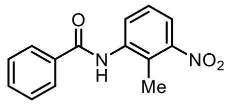

***N*-(2-methyl-3-nitrophenyl)benzamide (13).** Prepared according to Method A using benzonitrile (21 µl, 0.2 mmol), 1-iodo-2-methyl-3-nitrobenzene (157 mg, 0.6 mmol), and *E. coli* (CGA009) whole cells; the product was obtained as a white solid (34 mg, 0.13 mmol, 66%). **^{1}H NMR** (400 MHz, DMSO) δ 10.29 (s, 1H), 8.03 – 7.99 (m, 2H), 7.82 (dd, *J* = 8.1, 1.3 Hz, 1H), 7.69 (m, 1H), 7.66 – 7.60 (m, 1H), 7.59 – 7.53 (m, 2H), 7.49 (dd, *J* = 12.2, 4.3 Hz, 1H), 2.30 ppm (s, 3H). **^{13}C NMR** (101 MHz, DMSO) δ 165.68, 150.91, 138.42, 133.83, 131.94, 131.38, 128.52, 128.27, 127.76, 126.73, 121.57, 14.05 ppm. **HR-MS:** calc. for $[M+H]^+$ $C_{14}H_{13}N_2O_3$ = 257.09207 found 257.09265 (-2.27 ppm).

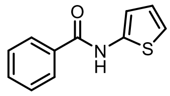

N-(thiophen-2-yl)benzamide (14). Prepared according to Method A using benzonitrile (21 µl, 0.2 mmol), 3-iodothiophene (61 µL, 0.6 mmol), and *E. coli* (CGA009) whole cells; the product was obtained as a white solid (28 mg, 0.14 mmol, 69%). ^{1}**H NMR** (400 MHz, DMSO) δ 10.68 (s, 1H), 8.00 – 7.92 (m, 2H), 7.75 (dd, *J* = 3.2, 1.3 Hz, 1H), 7.54 (m, 4H), 7.32 ppm (dd, *J* = 5.2, 1.3 Hz, 1H). ^{13}**C NMR** (101 MHz, DMSO) δ 164.45, 136.97, 134.37, 131.55, 128.42, 127.49, 124.47, 122.09, 109.50 ppm. **HR-MS:** calc. for $[M+H]^+$ $C_{11}H_{10}NOS$ = 204.04776 found 204.04792 (-0.78 ppm).

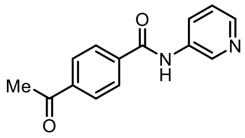

4-Acetyl-*N*-(pyridin-3-yl)benzamide (15). Prepared according to Method A using 4-acetylbenzonitrile (29 mg, 0.2 mmol), 3-iodopyridine (123 mg, 0.6 mmol), and *E. coli* (CGA) whole cells; the product was obtained as a light brown solid (32 mg, 0.13 mmol, 66%). ^{1}**H NMR** (400 MHz, Chloroform-*d*) δ 8.80 – 8.75 (m, 1H), 8.54 (s, 1H), 8.39 (d, *J* = 4.8 Hz, 1H), 8.37 – 8.32 (m, 1H), 8.02 (q, *J* = 8.5 Hz, 4H), 7.35 (dd, *J* = 8.3, 4.8 Hz, 1H), 2.65 ppm (s, 3H); ^{13}**C NMR** (100 MHz, Chloroform-*d*) δ 197.52, 165.45, 145.51, 141.56, 139.80, 138.15, 135.06, 128.84, 128.19, 127.77, 124.07, 27.01 ppm. **HRMS** calc. for $[M+H]^+$ $C_{14}H_{13}N_2O_2^+$= 241.0972 found: 241.09741 (+1.12 ppm).

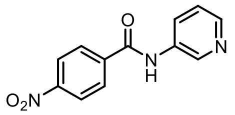

4-Nitro-*N*-(pyridin-3-yl)benzamide (16). Prepared according to Method A using 4-nitrobenzonitrile (30 mg, 0.2 mmol), 3-iodopyridine (123 mg, 0.6 mmol), and *E. coli* (CGA) whole cells; the product was obtained as a an off white solid (21.4 mg, 0.09 mmol, 44%). ^{1}**H NMR** (400 MHz, DMSO-d_6) δ 10.80 (s, 1H), 8.95 (d, *J* = 2.6 Hz, 1H), 8.43 – 8.34 (m, 3H), 8.22 (dq, *J* = 9.2, 2.4 Hz, 3H), 7.46 ppm (dd, *J* = 8.3, 4.7 Hz, 1H); ^{13}**C NMR** (100 MHz,

DMSO-d_6) δ 164.32, 149.34, 144.77, 141.74, 139.91, 135.50, 129.31, 127.83, 123.77, 123.63 ppm. HRMS calcd $[M+H]^+$ for $C_{12}H_{10}N_3O_3^+$: 244.0717 found: 244.07232 (+2.54 ppm).

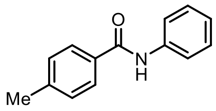

4-Methyl-*N*-phenylbenzamide (17). Prepared according to Method A using 4-acetylbenzonitrile (29 mg, 0.2 mmol), iodobenzene (66 µL, 0.6 mmol), and *E. coli* (CGA009) whole cells; the product was obtained as a white solid (32 mg, 0.15 mmol, 75%). **^{1}H NMR** (400 MHz, DMSO) δ 10.14 (s, 1H), 7.87 (d, *J* = 8.2 Hz, 2H), 7.77 (dd, *J* = 8.6, 1.2 Hz, 2H), 7.38 – 7.25 (m, 4H), 7.15 – 6.99 (m, 1H), 2.39 ppm (s, 3H). **^{13}C NMR** (101 MHz, DMSO) δ 165.33, 141.54, 139.23, 132.07, 128.89, 128.56, 127.67, 123.53, 120.34, 21.01 ppm. **HR-MS:** calc. for $[M+H]^+$ $C_{14}H_{14}NO$ = 212.10699 found 212.10781 (-3.88 ppm).

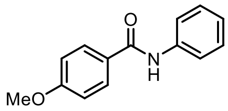

4-Methoxy-*N*-phenylbenzamide (18). Prepared according to Method A using 4-methoxybenzonitrile (27 mg, 0.2 mmol), iodobenzene (66 µL, 0.6 mmol), and *E. coli* (CGA009) whole cells; the product was obtained as a white solid (38 mg, 0.17 mmol, 84%). **^{1}H NMR** (400 MHz, DMSO) δ 10.07 (s, 1H), 7.96 (d, *J* = 8.8 Hz, 2H), 7.76 (dd, *J* = 8.8, 1.2 Hz, 2H), 7.34 (dd, *J* = 8.5, 7.3 Hz, 2H), 7.12 – 6.97 (m, 3H), 3.84 ppm (s, 3H). **^{13}C NMR** (101 MHz, DMSO) δ 164.86, 161.86, 139.33, 129.56, 128.53, 126.96, 123.39, 120.31, 113.57, 55.42 ppm. **HR-MS:** calc. for $[M+H]^+$ $C_{14}H_{14}NO_2$ = 228.10191 found 228.10243 (-2.31 ppm).

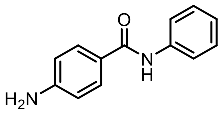

4-Amino-*N*-phenylbenzamide (19). Prepared according to Method A using 4-aminobenzonitrile (17 µL, 0.2 mmol), iodobenzene (66 µL, 0.6 mmol), and *E. coli* (CGA009) whole cells; the product was obtained as a white solid (10 mg, 0.05 mmol, 24%). **^{1}H NMR** (400 MHz, $CDCl_3$) δ 7.76 – 7.66 (m, 3H), 7.62 (dd, *J* = 8.6, 1.2 Hz, 2H), 7.35 (dd, *J* = 8.6, 7.3 Hz, 2H), 7.12 (t, *J* = 7.4 Hz, 1H), 6.70 (d, *J* = 8.6 Hz, 2H), 4.03 ppm (s, 2H). ^{13}C NMR (101 MHz, $CDCl_3$) δ 165.53, 150.07, 138.46, 129.16, 129.02, 124.43, 124.21, 120.19, 114.39 ppm. **HR-MS:** calc. for $[M+H]^+$ $C_{13}H_{13}N_2O$ = 213.10224 found 213.10267 (-2.03 ppm).

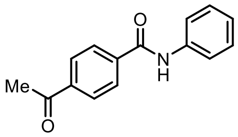

4-Acetyl-*N*-phenylbenzamide (20). Prepared according to Method A using 4-acetylbenzonitrile (29 mg, 0.2 mmol), iodobenzene (66 µL, 0.6 mmol), and *E. coli* (CGA009) whole cells; the product was obtained as a white solid (38 mg, 0.17 mmol, 79%). **^{1}H NMR** (400 MHz, DMSO) δ 10.41 (s, 1H), 8.13 – 8.03 (m, 4H), 7.82 – 7.73 (m, 2H), 7.41 – 7.31 (m, 2H), 7.17 – 7.06 (m, 1H), 2.65 ppm (s, 3H). **^{13}C NMR** (101 MHz, DMSO) δ 197.71, 164.75, 138.90, 138.83, 138.75, 128.66, 128.18, 128.01, 123.94, 120.44, 27.01 ppm. **HR-MS:** calc. for $[M+H]^+$ $C_{15}H_{14}NO_2$ = 240.10191 found 240.10273 (-3.45 ppm).

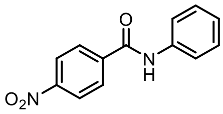

4-Nitro-*N*-phenylbenzamide (21). Prepared according to Method A using 4-nitrobenzonitrile (30 mg, 0.2 mmol), iodobenzene (66 µL, 0.6 mmol), and *E. coli* (CGA009) whole cells; the product was obtained as a white solid (24 mg, 0.1 mmol, 50%). **^{1}H NMR** (400 MHz, DMSO) δ 10.56 (s, 1H), 8.37 (d, *J* = 8.8 Hz, 2H), 8.19 (d, *J* = 8.8 Hz, 2H), 7.78 (dd, *J* = 8.5, 1.3 Hz, 2H), 7.38 (dd, *J* = 8.5, 7.3 Hz, 2H), 7.23 – 7.06 ppm (m, 1H). **^{13}C NMR** (101 MHz, DMSO) δ

163.88, 149.14, 140.62, 138.69, 129.20, 128.71, 124.17, 123.55, 120.47 ppm. **HR-MS:** calc. for $[M+H]^+$ $C_{13}H_{11}N_2O_3$ = 243.07642 found 243.0775 (-4.47 ppm).

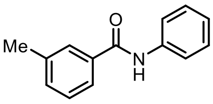

3-Methyl-*N*-phenylbenzamide (22). Prepared according to Method A using 3-methylbenzonitrile (24 µL, 0.2 mmol), iodobenzene (66 µL, 0.6 mmol), and *E. coli* (CGA009) whole cells; the product was obtained as a white solid (38 mg, 0.18 mmol, 89%). **^{1}H NMR** (400 MHz, DMSO) δ 10.19 (s, 1H), 7.83 – 7.65 (m, 4H), 7.45 – 7.38 (m, 2H), 7.35 (dd, *J* = 8.5, 7.3 Hz, 2H), 7.13 – 7.05 (m, 1H), 2.40 pp (s, 3H). **^{13}C NMR** (101 MHz, DMSO) δ 165.63, 139.20, 137.66, 134.98, 132.09, 128.57, 128.26, 128.11, 124.79, 123.57, 120.30, 20.96 ppm. **HR-MS:** calc. for $[M+H]^+$ $C_{14}H_{14}NO$ = 212.10699 found 212.1074 (-1.94 ppm).

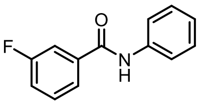

3-Fluoro-*N*-phenylbenzamide (23). Prepared according to Method A using 3-fluorobenzonitrile (21 µL, 0.2 mmol), iodobenzene (66 µL, 0.6 mmol), and *E. coli* (CGA009) whole cells; the product was obtained as a white solid (38 mg, 0.18 mmol, 88%). **^{1}H NMR** (400 MHz, DMSO) δ 10.30 (s, 1H), 7.83 – 7.69 (m, 4H), 7.59 (m, 1H), 7.49 – 7.37 (m, 1H), 7.36 (dd, *J* = 8.5, 7.4 Hz, 2H), 7.17 – 7.03 ppm (m, 1H). **^{13}C NMR** (101 MHz, DMSO) δ 164.12 (d, J_{CF} = 2.6 Hz), 163.13, 160.70, 138.87, 137.25 (d, J_{CF} = 6.8 Hz), 130.58 (d, J_{CF} = 8.2 Hz), 128.64, 124.59 – 123.17 (m), 120.44, 118.45 (d, J_{CF} = 21.1 Hz), 114.47 ppm (d, J_{CF} = 22.9 Hz). **HR-MS:** calc. for $[M+H]^+$ $C_{13}H_{11}FNO$ = 216.08192 found 216.08282 (-4.19 ppm).

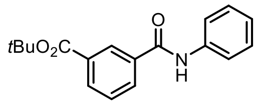

***tert*-Butyl 3-(phenylcarbamoyl)benzoate (24).** Prepared according to Method A using *tert*-butyl 3-cyanobenzoate (41 mg, 0.2 mmol), iodobenzene 66 µL, 0.6 mmol), and *E. coli* (CGA009) whole cells for 48 h; the product was obtained as a white solid (39 mg, 0.13 mmol, 66%). **^{1}H NMR** (400 MHz, $CDCl_3$) δ 8.41 (t, *J* = 1.8 Hz, 1H), 8.24 (s, 1H), 8.10 (m, 1H), 8.06 – 7.99 (m, 1H), 7.65 (dd, *J* = 8.6, 1.2 Hz, 2H), 7.47 (t, *J* = 7.8 Hz, 1H), 7.34 (dd, *J* = 8.5, 7.4 Hz, 2H), 7.18 – 7.10 (m, 1H), 1.59 ppm (s, 9H). **^{13}C NMR** (101 MHz, $CDCl_3$) δ 165.25, 165.03, 137.91, 135.25, 132.58, 132.49, 131.49, 129.14, 128.88, 127.68, 124.81, 120.53, 81.93, 28.24 ppm. **HR-MS:** calc. for $[M+H]^+$ $C_{18}H_{20}NO_3$ = 298.14377 found 298.1446 (-2.79 ppm).

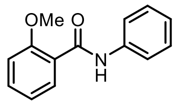

2-Methoxy-*N*-phenylbenzamide (25). Prepared according to Method A using 2-methoxybenzonitrile (27 mg, 0.2 mmol), iodobenzene (66 µL, 0.6 mmol), and *E. coli* (CGA009) whole cells; the product was obtained as a white solid (20 mg, 0.09 mmol, 44%). **^{1}H NMR** (400 MHz, $CDCl_3$) δ 9.80 (s, 1H), 8.30 (d, *J* = 9.6 Hz, 1H), 7.68 (dd, *J* = 8.5, 1.3 Hz, 2H), 7.50 (ddd, *J* = 8.5, 7.3, 1.9 Hz, 1H), 7.36 (dd, *J* = 8.5, 7.4 Hz, 2H), 7.13 (m, 2H), 7.04 (dd, *J* = 8.3, 1.0 Hz, 1H), 4.06 ppm (s, 3H). **^{13}C NMR** (101 MHz, $CDCl_3$) δ 163.35, 157.34, 138.54, 133.36, 132.70, 129.12, 124.28, 121.98, 121.85, 120.59, 111.68, 56.38 ppm. **HR-MS:** calc. for $[M+H]^+$ $C_{14}H_{14}NO_2$ = 228.10191 found 228.1029 (-4.38 ppm).

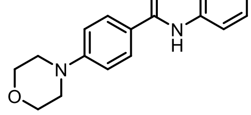

4-Morpholino-*N*-phenylbenzamide (26). Prepared according to Method A using 4-morpholinobenzonitrile (37 mg, 0.2 mmol), iodobenzene (66 µL, 0.6 mmol), and *E. coli* (CGA009) whole cells; the product was obtained as a white solid (26 mg, 0.09 mmol, 46%).

^{1}H NMR (400 MHz, DMSO) δ 9.94 (s, 1H), 7.89 (d, *J* = 8.9 Hz, 2H), 7.76 (dd, *J* = 8.5, 1.3 Hz, 2H), 7.32 (dd, *J* = 8.5, 7.3 Hz, 2H), 7.10 – 6.99 (m, 3H), 3.79 – 3.71 (m, 4H), 3.29 – 3.20 ppm (m, 4H). **^{13}C NMR** (101 MHz, DMSO) δ 164.94, 153.16, 139.51, 129.02, 128.48, 124.10, 123.15, 120.21, 113.32, 65.91, 47.27 ppm. **HR-MS:** calc. for $[M+H]^+$ $C_{17}H_{19}N_2O_2$ = 283.1441 found 283.14469 (-2.08 ppm).

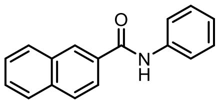

***N*-phenyl-2-naphthamide (27).** Prepared according to Method A using 2-naphthonitrile (30 mg, 0.2 mmol), iodobenzene (66 µL, 0.6 mmol), and *E. coli* (CGA009) whole cells; the product was obtained as a white solid (40 mg, 0.16 mmol, 81%). **^{1}H NMR** (400 MHz, DMSO) δ 10.43 (s, 1H), 8.58 (d, *J* = 1.5 Hz, 1H), 8.14 – 7.95 (m, 4H), 7.88 – 7.78 (m, 1H), 7.70 – 7.56 (m, 1H), 7.38 (dd, *J* = 8.5, 7.3 Hz, 1H), 7.17 – 7.05 ppm (m, 1H). **^{13}C NMR** (101 MHz, DMSO) δ 165.57, 139.23, 134.25, 132.28, 132.07, 128.93, 128.63, 127.99, 127.94, 127.80, 127.66, 126.84, 124.45, 123.66, 120.35 ppm. **HR-MS:** calc. for $[M+H]^+$ $C_{17}H_{14}NO$ = 248.10699 found 248.10779 (-3.24 ppm).

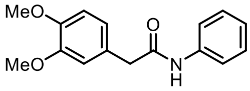

2-(3,4-Dimethoxyphenyl)-*N*-phenylacetamide (28). Prepared according to Method A using (3,4-dimethoxyphenyl)acetonitrile (36 mg, 0.2 mmol), iodobenzene (66 µL, 0.6 mmol), and *E. coli* (CGA009) whole cells; the product was obtained as a white solid (38 mg, 0.14 mmol, 70%). **^{1}H NMR** (400 MHz, DMSO) δ 10.07 (s, 1H), 7.59 (dd, *J* = 8.6, 1.2 Hz, 2H), 7.28 (dd, *J* = 8.6, 7.3 Hz, 2H), 7.07 – 6.98 (m, 1H), 7.05 – 6.99 (m, 1H), 6.95 (d, *J* = 1.9 Hz, 1H), 6.89 (d, *J* = 8.2 Hz, 1H), 6.84 (m, 1H), 3.74 (s, 3H), 3.72 (s, 3H), 3.54 ppm (s, 2H). **^{13}C NMR** (101 MHz, DMSO) δ 169.32, 148.51, 147.59, 139.25, 128.67, 128.34, 123.13, 121.04, 119.08, 113.02, 111.83, 55.54, 55.44, 42.90 ppm. **HR-MS:** calc. for $[M+H]^+$ $C_{16}H_{17}NO_3$ = 272.12812 found 272.12893 (-2.99 ppm).

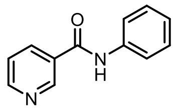

***N*-phenylnicotinamide (29).** Prepared according to Method A using 3-pyridinecarbonitrile (18 µL, 0.2 mmol), iodobenzene (66 µL, 0.6 mmol), and *E. coli* (CGA009) whole cells; the product was obtained as a white solid (24 mg, 0.12 mmol, 60%). **^{1}H NMR** (400 MHz, $CDCl_3$) δ 9.08 (d, *J* = 2.3 Hz, 1H), 8.75 (dd, *J* = 4.9, 1.7 Hz, 1H), 8.20 (m, 1H), 8.08 (s, 1H), 7.63 (t, *J* = 4.2 Hz, 2H), 7.45 – 7.34 (m, 3H), 7.18 ppm (t, *J* = 7.4 Hz, 1H). **^{13}C NMR** (101 MHz, $CDCl_3$) δ 164.03, 152.64, 147.99, 137.59, 135.48, 130.94, 129.32, 125.22, 123.83, 120.60 ppm. **HR-MS:** calc. for $[M+H]^+$ $C_{12}H_{11}N_2O$ = 199.08659 found 199.08751 (-4.65 ppm).

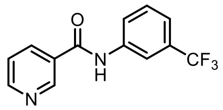

***N*-(3-(trifluoromethyl)phenyl)nicotinamide (30).** Prepared according to Method A using 3-pyridinecarbonitrile (18 µL, 0.2 mmol), 1-iodo-3-(trifluoromethyl)-benzene (86 µL, 0.6 mmol), and *E. coli* (CGA009) whole cells; the product was obtained as a white solid (35 mg, 0.13 mmol, 65%). **^{1}H NMR** (400 MHz, DMSO) δ 10.73 (s, 1H), 9.13 (dd, *J* = 2.4, 0.9 Hz, 1H), 8.79 (dd, *J* = 4.8, 1.7 Hz, 1H), 8.35 – 8.28 (m, 1H), 8.25 (t, *J* = 2.0 Hz, 1H), 8.09 – 7.98 (m, 1H), 7.68 – 7.53 (m, 2H), 7.49 ppm (m, 1H). **^{13}C NMR** (101 MHz, DMSO) δ 164.48, 152.39, 148.72, 139.63, 135.52, 130.14, 129.98, 129.40 (d, J_{CF} = 31.7 Hz), 124.11 (d, J_{CF} = 272.2 Hz), 123.80, 123.55, 120.27 (d, J_{CF} = 3.9 Hz), 116.36 ppm (d, *J* = 4.1 Hz). **HR-MS:** calc. for $[M+H]^+$ $C_{13}H_{10}F_3N_2O$ = 267.07397 found 267.0752 (-4.61 ppm).

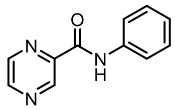

***N*-phenylpyrazine-2-carboxamide (31).** Prepared according to Method A using pyrazine-2-carbonitrile (18 µL, 0.2 mmol), iodobenzene (66 µL, 0.6 mmol), and *E. coli* (CGA009) whole cells; the product was obtained as a white solid (34 mg, 0.17 mmol, 85%). **^{1}H NMR** (400 MHz, DMSO) δ 10.72 (s, 1H), 9.30 (d, *J* = 1.4 Hz, 1H), 8.93 (d, *J* = 2.5 Hz, 1H), 8.81 (dd, *J* = 2.5, 1.4 Hz, 1H), 7.96 – 7.78 (m, 2H), 7.47 – 7.31 (m, 2H), 7.23 – 7.06 ppm (m, 1H). **^{13}C NMR** (101 MHz, DMSO) δ 161.65, 147.69, 145.07, 144.04, 143.21, 138.14, 128.68, 124.24,

120.52 ppm. **HR-MS:** calc. for $[M+H]^+$ $C_{11}H_{10}N_3O$ = 200.08184 found 200.08261 (-3.88 ppm).

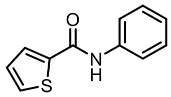

***N*-phenylthiophene-2-carboxamide (32).** Prepared according to Method A using thiophene-2-carbonitrile (18 µL, 0.2 mmol), iodobenzene (66 µL, 0.6 mmol), and *E. coli* (CGA009) whole cells; the product was obtained as a white solid (38 mg, 0.19 mmol, 94%). **^{1}H NMR** (400 MHz, DMSO) δ 10.21 (s, 1H), 8.02 (dd, *J* = 3.8, 1.1 Hz, 1H), 7.85 (dd, *J* = 5.0, 1.0 Hz, 1H), 7.77 – 7.66 (m, 2H), 7.35 (dd, *J* = 8.5, 7.3 Hz, 2H), 7.22 (dd, *J* = 5.0, 3.7 Hz, 1H), 7.14 – 7.05 ppm (m, 1H). **^{13}C NMR** (101 MHz, DMSO) δ 159.87, 140.05, 138.70, 131.84, 129.09, 128.67, 128.05, 123.75, 120.39 ppm. **HR-MS:** calc. for $[M+H]^+$ $C_{11}H_{10}NOS$ = 204.04776 found 204.04872 (-4.72 ppm).

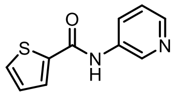

***N*-(pyridin-3-yl)thiophene-2-carboxamide (33).** Prepared according to Method A using thiophene-2-carbonitrile (18 µL, 0.2 mmol), 3-iodopyridine (123 mg, 0.6 mmol), and *E. coli* (CGA) whole cells; the product was obtained as a white solid (24 mg, 0.11 mmol, 57%). **^{1}H NMR** (400 MHz, DMSO-d_6) δ 10.38 (s, 1H), 8.88 – 8.79 (m, 1H), 8.26 (dd, *J* = 4.7, 1.5 Hz, 1H), 8.09 (m, 1H), 7.99 (dd, *J* = 3.8, 1.2 Hz, 1H), 7.84 (dd, *J* = 5.0, 1.1 Hz, 1H), 7.35 (m, 1H), 7.19 (dd, *J* = 5.0, 3.8 Hz, 1H); **^{13}C NMR** (100 MHz, DMSO-d_6) δ 160.26, 144.58, 141.86, 139.28, 135.46, 132.35, 129.64, 128.17, 127.44, 123.61 ppm. **HRMS** calc. for $[M+H]^+$ $C_{10}H_9N_2OS^+$ = 205.0430 found: 205.04240 (-2.93 ppm).

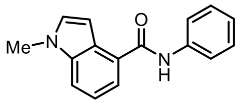

1-Methyl-*N*-phenyl-1*H*-indole-4-carboxamide (34). Prepared according to Method A using **72** (31 mg, 0.2 mmol), iodobenzene (66 µL, 0.6 mmol), and *E. coli* (CGA009) whole cells; the product was obtained as a white solid (49 mg, 0.2 mmol, 98%). **^{1}H NMR** (400 MHz, DMSO) δ 10.18 (s, 1H), 7.82 (d, *J* = 8.6 Hz, 2H), 7.66 (d, *J* = 8.2 Hz, 1H), 7.58 (d, *J* = 6.9 Hz, 1H), 7.45 (d, *J* = 3.1 Hz, 1H), 7.39 – 7.31 (m, 2H), 7.31 – 7.23 (m, 1H), 7.09 (t, *J* = 7.4 Hz, 1H), 6.82 (d, *J* = 3.5 Hz, 1H), 3.85 ppm (s, 3H). ^{13}C NMR (101 MHz, DMSO) δ 166.67, 139.56, 136.97, 131.03, 128.55, 127.05, 126.46, 123.26, 120.24, 120.06, 119.00, 112.83, 100.87, 32.64 ppm. **HR-MS:** calc. for $[M+H]^+$ $C_{16}H_{15}N_2O$ = 251.11789 found 251.11877 (-3.52 ppm).

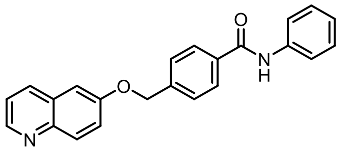

***N*-phenyl-4-((quinolin-6-yloxy)methyl)benzamide (35).** Prepared according to Method A using **73** (52 mg, 0.2 mmol), iodobenzene (66 µL, 0.6 mmol), and *E. coli* (CGA009) whole cells; the product was obtained as a white solid (60 mg, 0.17 mmol, 85%). **^{1}H NMR** (400 MHz, DMSO) δ 10.26 (s, 1H), 8.75 (dd, *J* = 4.2, 1.7 Hz, 1H), 8.24 (dd, *J* = 8.3, 1.6 Hz, 1H), 8.03 – 7.98 (m, 2H), 7.96 (d, *J* = 9.0 Hz, 1H), 7.79 (d, *J* = 8.5, 2H), 7.67 (d, *J* = 8.0 Hz, 2H), 7.54 – 7.45 (m, 3H), 7.35 (t, *J* = 7.9 Hz, 2H), 7.10 (t, *J* = 7.4 Hz, 1H), 5.36 ppm (s, 2H). **^{13}C NMR** (101 MHz, DMSO) δ 165.24, 156.01, 148.13, 143.88, 140.27, 139.13, 134.79, 134.52, 130.51, 128.92, 128.59, 127.89, 127.47, 123.66, 122.21, 121.72, 120.33, 107.21, 69.01 ppm. **HR-MS:** calc. for $[M+H]^+$ $C_{23}H_{19}N_2O_2$ = 355.1441 found 355.14454 (-1.23 ppm).

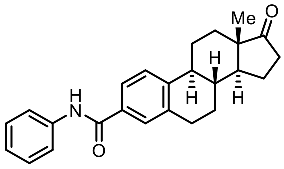

(8*R*,9*S*,13*S*,14*S*)-13-methyl-17-oxo-*N*-phenyl-7,8,9,11,12,13,14,15,16,17-decahydro-6*H*-cyclopenta[*a*]phenanthrene-3-carboxamide (36). Prepared according to Method A using **75** (56 mg, 0.2 mmol), iodobenzene (66 µL, 0.6 mmol), and *E. coli* (HaA2) whole cells; the product was obtained as a white solid (22 mg, 0.07 mmol, 30%). **^{1}H NMR** (400 MHz, Chloroform-*d*) δ 7.79 (s, 1H), 7.68 – 7.59 (m, 4H), 7.44 – 7.32 (m, 3H), 7.18 – 7.11 (m, 1H), 2.99 (m, 2H), 2.52 (dd, *J* = 18.6, 8.6 Hz, 1H), 2.49 – 2.42 (m, 1H), 2.36 (td, *J* = 11.0, 4.1 Hz, 1H), 2.18 (q, *J* = 9.8, 9.2 Hz, 1H), 2.13 – 2.03 (m, 2H), 2.03 – 1.96 (m, 1H), 1.60 (m, 6H), 0.93 ppm (s, 3H); **^{13}C NMR** (100 MHz, Chloroform-*d*) δ Carbonyl peak out of acquisition range. 165.72, 144.17, 138.17, 137.43, 132.52, 129.23, 128.03, 125.92, 124.58, 124.20, 120.22, 50.66, 48.05, 44.72, 38.04, 35.96, 31.70, 29.50, 26.44, 25.79, 21.74, 13.98 ppm. **HRMS** calc. for $[M+H]^+$ $C_{25}H_{28}NO_2^+$ = 374.2115 found: 374.21255 (-1.46 ppm).

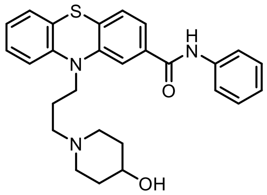

10-(3-(4-hydroxypiperidin-1-yl)propyl)-*N*-phenyl-10*H*-phenothiazine-2-carboxamide (37). Prepared according to Method A using **76** (73 mg, 0.2 mmol), iodobenzene (66 µL, 0.6 mmol), and *E. coli* (HaA2) whole cells; the product was obtained as a yellow oil (51.0 mg, 0.11 mmol, 55%). **^{1}H NMR** (400 MHz, Chloroform-d) δ 8.26 (s, 1H), 7.68 (d, *J* = 8.0 Hz, 2H), 7.42 (s, 1H), 7.38 – 7.27 (m, 3H), 7.18 – 7.08 (m, 4H), 6.96 – 6.85 (m, 2H), 3.98 (t, *J* = 6.9 Hz, 2H), 3.65 (p, *J* = 5.2 Hz, 1H), 2.72 (m, 2H), 2.48 (t, *J* = 7.1 Hz, 2H), 2.16 (s, 2H), 1.95 (p, *J* = 7.1 Hz, 2H), 1.86 (d, *J* = 13.0 Hz, 2H), 1.51 ppm (m, 2H); **^{13}C NMR** (100 MHz, Chloroform-*d*) δ 165.75, 145.84, 144.50, 138.21, 134.29, 130.02, 129.15, 127.74, 127.61, 127.24, 124.62, 124.24, 123.04, 120.48, 116.03, 114.86, 55.45, 51.08, 45.33, 34.04, 34.01, 24.31 ppm. **HRMS** calc. for $[M+H]^+$ $C_{27}H_{30}N_3O_2S^+$ = 460.2053 found: 460.20579 (-1.01 ppm).

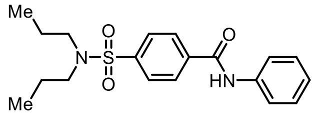

4-(*N*,*N*-dipropylsulfamoyl)-*N*-phenylbenzamide (38). Prepared according to Method A using **71** (53 mg, 0.2 mmol), iodobenzene (66 µL, 0.6 mmol), and *E. coli* (CGA) whole cells; the product was obtained as a white solid (57 mg, 0.16 mmol, 79%). **^{1}H NMR** (400 MHz, Chloroform-*d*) δ 8.63 (s, 1H), 7.93 – 7.85 (m, 2H), 7.70 (m, 4H), 7.39 – 7.31 (m, 2H), 7.18 – 7.12 (m, 1H), 3.10 – 3.03 (m, 4H), 1.59 – 1.47 (m, 4H), 0.85 ppm (t, *J* = 7.4 Hz, 6H); **^{13}C NMR** (100 MHz, Chloroform-*d*) δ 164.99, 142.55, 138.98, 138.00, 129.10, 128.15, 127.22, 124.90, 120.50, 50.08, 22.02, 11.23 ppm. **HRMS** calc. for $[M+H]^+$ $C_{19}H_{25}N_2O_3S^+$ = 361.1580 found: 361.15873 (-0.39 ppm).

Gram-scale procedure: To a solution of 4-cyano-*N*,*N*-dipropylbenzenesulfonamide (**71**) (1.07 g, 4 mmol, 1 equiv) in KPi buffer (0.1 M, pH = 7.8) / 10% v/v *i*PrOH were added *E. coli* (CGA009) whole cells from ca. 80 mL cell culture in KPi buffer (0.1 M, pH = 7.8) to a total volume of 80 mL and the reaction was stirred (400 rpm) for 24 h at room temperature. After that time, iodobenzene (1.34 mL, 12 mmol, 3 equiv), *trans*-dimethylcyclohexane-1,2-diamine (126 µL, 0.8 mmol, 0.2 equiv), D-glu (144 mg, 0.8 mmol, 0.2 equiv), $CuBr_2$ (90 mg, 0.4 mmol, 0.1 equiv), and NaO*t*Bu (768 mg, 8 mmol, 2 equiv) were successively added, and the reaction was vigorously stirred (1000 rpm) at 50 °C under N_2 atmosphere (headspace purge) for 24 h. After cooling to room temperature, the reaction was diluted with ethyl acetate and filtered through a pad of Celite©. The solution was washed with brine (100 mL), extracted with ethyl acetate (3x100 mL), dried over anhydrous $MgSO_4$ and concentrated under reduced pressure. Silica gel column chromatography (elute: *n*-hexane / ethyl acetate) afforded the desired product. The product was obtained as a white solid (1.04 g, 2.89 mmol, 72%). **^{1}H NMR** (400 MHz, $CDCl_3$) δ 8.90 (s, 1H), 7.79 (d, *J* = 8.1 Hz, 2H), 7.57 (dd, *J* = 32.4, 8.0 Hz, 4H), 7.21 (t, *J* = 7.8 Hz, 2H), 7.03 (t, *J* = 7.4 Hz, 1H), 3.07 – 2.82 (m, 4H), 1.41 (q, *J* = 7.5 Hz, 4H), 0.74 ppm (t, *J* = 7.4 Hz, 6H). **^{13}C NMR** (101 MHz, $CDCl_3$) δ 165.04, 142.23, 138.76, 137.94, 128.86, 128.12, 126.95, 124.71, 120.56, 49.94, 21.87, 11.07 ppm.

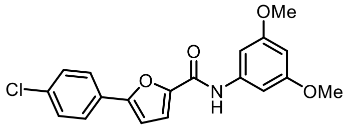

5-(4-chlorophenyl)-*N*-(3,5-dimethoxyphenyl)furan-2-carboxamide (39). Prepared according to Method A using **1aj** (40 mg, 0.2 mmol), 1,3-dimethoxy-5-iodobenzene (158 mg, 0.6 mmol), and *E. coli* (CGA009) whole cells; the product was obtained as a white pale orange solid (46 mg, 0.13 mmol, 64%). **^{1}H NMR** (400 MHz, DMSO) δ 10.11 (s, 1H), 8.06 – 7.92 (m, 2H), 7.63 – 7.50 (m, 2H), 7.40 (d, *J* = 3.6 Hz, 1H), 7.23 (d, *J* = 3.7 Hz, 1H), 7.06 (d, *J* = 2.3 Hz, 2H), 6.29 (t, *J* = 2.3 Hz, 1H), 3.75 ppm (s, 6H). **^{13}C NMR** (101 MHz, DMSO) δ 160.40, 155.94, 154.09, 146.84, 140.05, 133.25, 129.01, 128.13, 126.28, 117.16, 108.64, 98.80, 95.94, 55.16 ppm. **HR-MS:** calc. for $[M+H]^+$ $C_{19}H_{17}ClNO_4$ = 358.08406 found 358.08461 (-1.53 ppm).

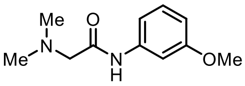

2-(Dimethylamino)-*N*-(3-methoxyphenyl)acetamide (42). Prepared according to Method B using 2-(dimethylamino)acetonitrile (59 µL, 0.6 mmol), 3-iodoanisole (24 µL, 0.2 mmol), and *E. coli* (AJ270) whole cells; the product was obtained as a white solid (25 mg, 0.12 mmol, 60%). **^{1}H NMR** (400 MHz, $CDCl_3$) δ 9.08 (s, 1H), 7.36 (s, 1H), 7.21 (t, *J* = 8.1 Hz, 1H), 7.05 (d, *J* = 8.1 Hz, 1H), 6.66 (dd, *J* = 8.1, 1.9 Hz, 1H), 3.81 (s, 3H), 3.06 (s, 2H), 2.37 ppm (s, 6H). **^{13}C NMR** (101 MHz, $CDCl_3$) δ 168.91, 160.30, 139.05, 129.76, 111.68, 110.15, 105.15, 63.83, 55.42, 46.13 ppm. **HR-MS:** calc. for $[M+H]^+$ $C_{11}H_{17}N_2O_2$ = 209.12845 found 209.12895 (-2.38 ppm).

Gram-scale procedure: To a solution of 2-(dimethylamino)acetonitrile (**40**) (1.17 mL, 12 mmol, 3 equiv) in KPi buffer (0.1 M, pH = 7.8) / 2% TPGS-750-M were added *E. coli* (AJ270) whole cells from ca. 80 mL cell culture in KPi buffer (0.1 M, pH = 7.8) / 2% TPGS-750-M to a total volume of 8 mL and the reaction was stirred (400 rpm) for 24 h at room temperature. After that time, 3-iodoanisole (936 µL, 4 mmol, 1 equiv), *trans*-*N*,*N*'-dimethylcyclohexane-1,2-diamine (126 µL, 0.8 mmol, 0.2 equiv), D-glu (144 mg, 0.8 mmol, 0.2 equiv), $CuBr_2$ (90 mg, 0.4 mmol, 0.1 equiv), and NaO*t*Bu (768 mg, 8 mmol, 2 equiv) were successively added, and the reaction was vigorously stirred (1000 rpm) at 50 °C under N_2 atmosphere (headspace purge) for 24 h. After cooling to room temperature, the reaction was

diluted with ethyl acetate and filtered through a pad of Celite©. The solution was washed with brine (100 mL), extracted with ethyl acetate (3x100 mL), dried over anhydrous $MgSO_4$ and concentrated under reduced pressure. Silica gel column chromatography (elute: *n*-hexane / ethyl acetate) afforded the desired product. The product was obtained as colorless oil (440 mg, 2.11 mmol, 52%). **^{1}H NMR** (400 MHz, $CDCl_3$) δ 9.09 (s, 1H), 7.36 (t, *J* = 2.2 Hz, 1H), 7.22 (t, *J* = 8.1 Hz, 1H), 7.05 (m, 1H), 6.66 (m, 1H), 3.81 (s, 3H), 3.07 (s, 2H), 2.38 ppm (s, 6H). **^{13}C NMR** (101 MHz, $CDCl_3$) δ 168.84, 160.32, 139.06, 129.78, 111.69, 110.18, 105.16, 63.81, 55.43, 46.11 ppm.

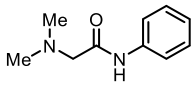

2-(Dimethylamino)-*N*-phenylacetamide (43). Prepared according to Method B using 2-(dimethylamino)acetonitrile (59 µL, 0.6 mmol), iodobenzene (22 µL, 0.2 mmol), and *E. coli* (AJ270) whole cells; the product was obtained as a white solid (29 mg, 0.16 mmol, 81%). **^{1}H NMR** (400 MHz, $CDCl_3$) δ 9.09 (s, 1H), 7.58 (d, *J* = 7.6 Hz, 2H), 7.32 (m, 2H), 7.09 (t, *J* = 7.4 Hz, 1H), 3.06 (s, 2H), 2.37 (s, 6H). **^{13}C NMR** (101 MHz, $CDCl_3$) δ 168.84, 137.79, 129.04, 124.14, 119.47, 63.75, 46.07 ppm. **HR-MS:** calc. for $[M+H]^+$ $C_{10}H_{15}N_2O$ = 179.11789 found 179.11867 (-4.38 ppm).

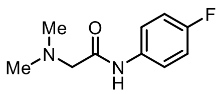

2-(Dimethylamino)-*N*-(4-fluorophenyl)acetamide (44). Prepared according to Method B using 2-(dimethylamino)acetonitrile (59 µL, 0.6 mmol), 4-fluoroiodobenzene (23 µL, 0.2 mmol), and *E. coli* (AJ270) whole cells; the product was obtained as a white solid (17 mg, 0.09 mmol, 43%). **^{1}H NMR** (400 MHz, $CDCl_3$) δ 9.08 (s, 1H), 7.59 – 7.50 (m, 2H), 7.02 (t, *J* = 8.7 Hz, 2H), 3.07 (s, 2H), 2.37 ppm (s, 6H). **^{13}C NMR** (101 MHz, $CDCl_3$) δ 168.84, 159.36 (d, J_{CF} = 242.9 Hz), 133.92 (d, J_{CF} = 2.9 Hz), 121.19 (d, J_{CF} = 7.9 Hz), 115.73 (d, J_{CF} = 22.5 Hz), 63.68, 46.17 ppm. **HR-MS:** calc. for $[M+H]^+$ $C_{10}H_{14}FN_2O$ = 197.10847 found 197.10935 (-4.5 ppm).

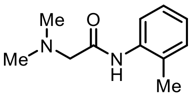

2-(Dimethylamino)-*N*-(*o*-tolyl)acetamide (45). Prepared according to Method B using 2-(dimethylamino)acetonitrile (59 µL, 0.6 mmol), 2-iodotoluene (25.5 µL, 0.2 mmol), and *E. coli* (AJ270) whole cells; the product was obtained as a white solid (20 mg, 0.1 mmol, 52%). **^{1}H NMR** (400 MHz, $CDCl_3$) δ 9.21 (s, 1H), 8.06 (dd, *J* = 8.1, 1.2 Hz, 1H), 7.25 – 7.15 (m, 2H), 7.04 (t, *J* = 7.4 Hz, 1H), 3.12 (s, 2H), 2.42 (s, 6H), 2.28 ppm (s, 3H). **^{13}C NMR** (101 MHz, $CDCl_3$) δ 168.67, 135.87, 130.44, 127.71, 126.98, 124.54, 121.53, 63.93, 46.24, 17.67 ppm. **HR-MS:** calc. for $[M+H]^+$ $C_{11}H_{17}N_2O$ = 193.13354 found 193.13426 (-3.75 ppm).

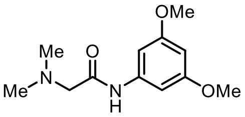

***N*-(3,5-dimethoxyphenyl)-2-(dimethylamino)acetamide (46).** Prepared according to Method B using 2-(dimethylamino)acetonitrile (59 µL, 0.6 mmol), 1,3-dimethoxy-5-iodobenzene (53 mg, 0.2 mmol), and *E. coli* (AJ270) whole cells; the product was obtained as a white solid (28 mg, 0.12 mmol, 58%). **^{1}H NMR** (400 MHz, $CDCl_3$) δ 9.06 (s, 1H), 6.83 (d, *J* = 2.3 Hz, 2H), 6.23 (t, *J* = 2.3 Hz, 1H), 3.78 (s, 6H), 3.06 (s, 2H), 2.37 (s, 6H) ppm. **^{13}C NMR** (101 MHz, $CDCl_3$) δ 169.01, 161.20, 139.53, 97.76, 96.80, 63.83, 55.52, 46.11 ppm. **HR-MS:** calc. for $[M+H]^+$ $C_{12}H_{19}N_2O_3$ = 239.13902 found 239.13982 (-3.36 ppm).

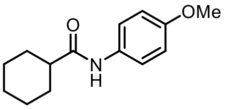

***N*-(4-methoxyphenyl)cyclohexanecarboxamide (47).** Prepared according to Method B using cyclohexanecarbonitrile (71 µL, 0.2 mmol), 4-iodoanisole (47 mg, 0.2 mmol), and *E. coli* (AJ270) whole cells; the product was obtained as a white solid (26 mg, 0.11 mmol, 56%). **^{1}H NMR** (400 MHz, $CDCl_3$) δ 7.42 (d, *J* = 8.9 Hz, 2H), 7.31 (s, 1H), 6.83 (d, *J* = 8.9 Hz, 2H), 3.77 (s, 3H), 2.20 (m, 1H), 1.93 (m, 2H), 1.86 – 1.61 (m, 3H), 1.53 (m, 2H), ppm 1.34 – 1.19 (m, 3H). **^{13}C NMR** (101 MHz, $CDCl_3$) δ 174.43, 156.33, 131.38, 121.81, 114.17, 55.59, 46.44, 29.81, 25.81 ppm. **HR-MS:** calc. for $[M+H]^+$ $C_{14}H_{20}NO_2$ = 234.14886 found 234.14994 (-4.65 ppm).

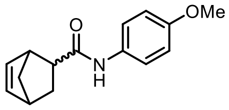

***N*-(4-methoxyphenyl)bicyclo[2.2.1]hept-5-ene-2-carboxamide (48).** Prepared according to Method B using 5-norbornene-2-carbonitrile (mixture of isomers) (72 µL, 0.6 mmol), 4-iodoanisole (47 mg, 0.2 mmol), and *E. coli* (AJ270) whole cells; the product was obtained as mixture of *exo*/*endo*-isomers.

The *exo*-product was obtained as a white solid (11 mg, 0.045 mmol, 23%). **^{1}H NMR** (400 MHz, $CDCl_3$) δ 7.42 (d, *J* = 8.9 Hz, 2H), 7.21 (s, 1H), 6.84 (d, *J* = 8.9 Hz, 2H), 6.16 (m, 2H), 3.78 (s, 3H), 3.00 (d, *J* = 33.5 Hz, 2H), 2.13 (dd, *J* = 9.6, 4.4 Hz, 1H), 2.02 (m, 1H), 1.78 (d, *J* = 8.3 Hz, 1H), 1.45 – 1.33 ppm (m, 2H). **^{13}C NMR** (101 MHz, $CDCl_3$) δ 173.89, 156.35, 138.62, 136.06, 131.48, 121.71, 114.24, 55.63, 47.49, 46.46, 45.81, 41.77, 30.78 ppm. **HR-MS:** calc. for $[M+H]^+$ $C_{15}H_{18}NO_2$ = 243.13321 found 243.1332 (-2.53 ppm).

The *endo*-product was obtained as a white solid (12 mg, 0.05 mmol, 24%). **^{1}H NMR** (400 MHz, $CDCl_3$) δ 7.37 (d, *J* = 8.9 Hz, 2H), 7.04 (s, 1H), 6.83 (d, *J* = 8.9 Hz, 2H), 6.29 (dd, *J* = 5.7, 3.1 Hz, 1H), 6.05 (dd, *J* = 5.8, 2.8 Hz, 1H), 3.78 (s, 3H), 3.22 (s, 1H), 3.06 – 2.88 (m, 2H), 2.01 (ddd, *J* = 11.9, 9.4, 3.8 Hz, 1H), 1.55 – 1.41 (m, 2H), 1.34 ppm (d, *J* = 8.3 Hz, 1H). **^{13}C NMR** (101 MHz, $CDCl_3$) δ 172.48, 156.32, 138.14, 132.27, 131.34, 121.64, 114.22, 55.63, 50.31, 46.65, 45.88, 43.00, 30.21 ppm. **HR-MS:** calc. for $[M+H]^+$ $C_{15}H_{18}NO_2$ = 243.13321 found 243.13393 (-2.98 ppm).

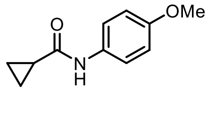

***N*-(4-methoxyphenyl)cyclopropanecarboxamide (49).** Prepared according to Method B using cyclopropanecarbonitrile (44 µL, 0.6 mmol), 4-iodoanisole (47 mg, 0.2 mmol), and *E. coli* (AJ270) whole cells; the product was obtained as a white solid (22 mg, 0.115 mmol, 57%). **^{1}H NMR** (400 MHz, $CDCl_3$) δ 7.63 (s, 1H), 7.39 (d, *J* = 8.5 Hz, 2H), 6.82 (d, *J* = 8.7 Hz, 2H), 3.77 (s, 3H), 1.54 – 1.39 (m, 1H), 1.05 (m, 2H), 0.79 ppm (m, 2H). **^{13}C NMR** (101 MHz, $CDCl_3$) δ 172.00, 156.29, 131.49, 121.81, 114.16, 55.58, 15.58, 7.83 ppm. **HR-MS:** calc. for $[M+H]^+$ $C_{11}H_{14}NO_2$ = 192.10191 found 192.10224 (-1.75ppm).

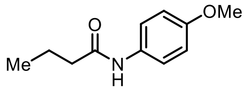

***N*-(4-methoxyphenyl)butyramide (50).** Prepared according to Method B using butyronitrile (52 µL, 0.6 mmol), 4-iodoanisole (47 mg, 0.2 mmol), and *E. coli* (AJ270) whole cells; the product was obtained as a white solid (24 mg, 0.12 mmol, 62%). **^{1}H NMR** (400 MHz, $CDCl_3$) δ 7.40 (m, 3H), 6.83 (d, *J* = 9.0 Hz, 2H), 3.77 (s, 3H), 2.29 (t, *J* = 7.4 Hz, 2H), 1.74 (p, *J* = 7.4 Hz, 2H), 0.98 ppm (t, *J* = 7.4 Hz, 3H). **^{13}C NMR** (101 MHz, $CDCl_3$) δ 171.42, 156.41, 131.24, 121.95, 114.18, 55.58, 39.55, 19.27, 13.88 ppm. **HR-MS:** calc. for $[M+H]^+$ $C_{11}H_{16}NO_2$ = 194.11756 found 194.11814 (-3.03 ppm).

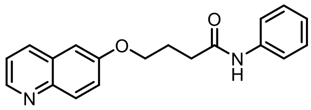

***N*-phenyl-4-(quinolin-6-yloxy)butanamide (51).** Prepared according to Method B using **78** (42 mg, 0.2 mmol), iodobenzene (66 µL, 0.6 mmol), and *E. coli* (CGA) whole cells; the product was obtained as a white solid (36 mg, 0.12 mmol, 59%). **^{1}H NMR** (400 MHz, DMSO) δ 9.96 (s, 1H), 8.73 (dd, *J* = 3.9, 1.6 Hz, 1H), 8.24 (d, *J* = 8.3 Hz, 1H), 7.91 (d, *J* = 8.8 Hz, 1H), 7.60 (d, *J* = 7.9 Hz, 2H), 7.47 (dd, *J* = 8.3, 4.2 Hz, 1H), 7.42 – 7.35 (m, 2H), 7.29 (t, *J* = 7.8 Hz, 2H), 7.02 (t, *J* = 7.4 Hz, 1H), 4.17 (t, *J* = 6.3 Hz, 2H), 2.55 (m, 2H), 2.12 ppm (m, 2H). **^{13}C NMR** (101 MHz, DMSO) δ 170.61, 156.43, 147.84, 143.67, 139.27, 134.81, 130.28, 129.05, 128.65, 122.97, 122.18, 121.62, 119.03, 106.42, 67.35, 32.77, 24.53 ppm. **HR-MS:** calc. for $[M+H]^+$ $C_{19}H_{19}N_2O_2$ = 307.1441 found 307.14469 (-1.91 ppm).

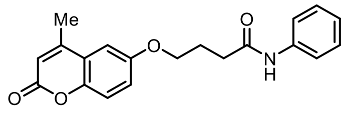

4-((4-Methyl-2-oxo-2H-chromen-6-yl)oxy)-*N*-phenylbutanamide (52). Prepared according to Method B using **59** (49 mg, 0.2 mmol), iodobenzene (66 µL, 0.6 mmol), and *E. coli* (CGA) whole cells; the product was obtained as a white solid (14 mg, 0.04 mmol, 21%). **^{1}H NMR** (400 MHz, DMSO) δ 9.94 (s, 1H), 7.67 (d, *J* = 8.6 Hz, 1H), 7.58 (t, *J* = 4.2 Hz, 2H), 7.28 (dd, *J* = 8.5, 7.3 Hz, 2H), 7.05 – 6.89 (m, 3H), 6.20 (d, *J* = 1.4 Hz, 1H), 4.13 (t, *J* = 6.3 Hz, 2H), 2.51 – 2.46 (m, 2H), 2.38 (m, 3H), 2.06 ppm (m, 2H). **^{13}C NMR** (101 MHz, DMSO) δ 170.52, 161.63, 160.14, 154.74, 153.40, 139.25, 128.64, 126.46, 122.97, 119.03, 113.08, 112.38, 111.10, 101.22, 67.75, 32.62, 24.35, 18.12 ppm. **HR-MS:** calc. for $[M+H]^+$ $C_{20}H_{20}NO_4$ = 338.13868 found 338.13897 (-0.85 ppm).

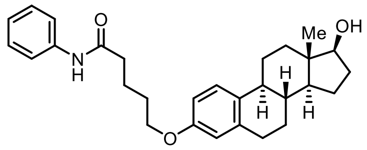

5-(((8R,9S,13S,14S,17S)-17-Hydroxy-13-methyl-7,8,9,11,12,13,14,15,16,17-decahydro-6H-cyclopenta[a]phenanthren-3-yl)oxy)-*N*-phenylpentanamide (53). Prepared according to Method B using **7l** (70 mg, 0.2 mmol), iodobenzene (66 µL, 0.6 mmol), and *E. coli* (CGA009) whole cells; the product was obtained as a pale white solid (53 mg, 0.12 mmol, 59%). **^{1}H NMR** (400 MHz, $CDCl_3$) δ 7.51 (d, *J* = 7.8 Hz, 2H), 7.45 (s, 1H), 7.31 (t, *J* = 7.9 Hz, 2H), 7.23 – 7.05 (m, 2H), 6.70 (dd, *J* = 8.6, 2.8 Hz, 1H), 6.63 (d, *J* = 2.8 Hz, 1H), 3.98 (dd, *J* = 8.1, 3.7 Hz, 2H), 3.73 (t, *J* = 8.5 Hz, 1H), 2.94 – 2.69 (m, 2H), 2.44 (dd, *J* = 8.4, 5.8 Hz, 2H), 2.31 (dd, *J* = 13.5, 3.5 Hz, 1H), 2.24 – 2.04 (m, 2H), 1.99 – 1.83 (m, 6H), 1.77 – 1.63 (m, 1H), 1.57 – 1.13 (m, 8H), 0.78 ppm (s, 3H). **^{13}C NMR** (101 MHz, $CDCl_3$) δ 171.18, 156.81, 138.17, 138.05, 132.90, 129.10, 126.50, 124.31, 119.91, 114.59, 112.10, 82.03, 67.73, 50.15, 44.08, 43.39, 38.97, 37.36, 36.84, 30.72, 29.93, 28.66, 27.37, 26.45, 23.26, 22.75, 11.20 ppm. **HR-MS:** calc. for $[M+H]^+$ $C_{29}H_{38}NO_3$ = 448.28462 found 448.28514 (-1.16 ppm).

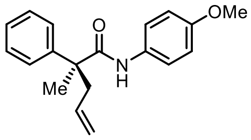

(*S*)-*N*-(4-Methoxyphenyl)-2-methyl-2-phenylpent-4-enamide (56). Prepared according to Method C using (±)-2-methyl-2-phenylpent-4-enenitrile (85 mg, 0.5 mmol), 4-iodoanisole (47 mg, 0.2 mmol), and *E. coli* (AJ270) whole cells; the product was obtained as a white solid (42 mg, 0.14 mmol, 71%). 72:28 e.r. $[\alpha]_D^{25}$ = +14.1 (*c* = 0.1 M, $CHCl_3$). **^{1}H NMR** (400 MHz, $CDCl_3$) δ 7.36 – 7.21 (m, 5H), 7.18 (d, *J* = 9.5 Hz, 2H), 6.73 (d, *J* = 8.9 Hz, 2H), 6.70 (s, 1H), 5.60 – 5.44 (m, 1H), 5.02 (d, *J* = 17.0 Hz, 1H), 4.97 (d, *J* = 11.2 Hz, 1H), 3.69 (s, 3H), 2.84 – 2.71 (m, 2H), 1.55 ppm (s, 3H). **^{13}C NMR** (101 MHz, $CDCl_3$) δ 174.89, 156.57, 143.18, 134.12, 130.99, 129.04, 127.51, 127.16, 121.94, 118.72, 114.17, 55.61, 51.11, 43.75, 23.95 ppm. **HR-MS:** calc. for $[M+H]^+$ $C_{20}H_{23}NO_2$ = 296.1651 found 296.16498 (-1.61 ppm); Chiral HPLC conditions: *i*-Cellulose 4.6 x 250mm column, *n*-hexane / iPrOH = 90:10, flow rate = 1 mL min^{-1}, minor enantiomer: t_R = 20.81 min; major enantiomer: t_R = 17.25 min.

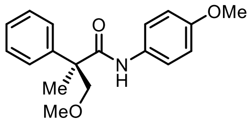

(-)-3-Methoxy-*N*-(4-methoxyphenyl)-2-methyl-2-phenylpropanamide (57). Prepared according to Method C using (±)-3-methoxy-2-methyl-2-phenylpropanenitrile (87 mg, 0.5 mmol), 4-iodoanisole (47 mg, 0.2 mmol), and *E. coli* (AJ270) whole cells; the product was obtained as a white solid (31 mg, 0.1 mmol, 52%). 67:33 e.r. $[\alpha]_D^{25}$ = -16.1 (*c* = 0.2 M, $CHCl_3$). **^{1}H NMR** (400 MHz, $CDCl_3$) δ 8.31 (s, 1H), 7.43 – 7.28 (m, 5H), 7.31 – 7.18 (m, 1H), 6.88 – 6.75 (m, 2H), 3.95 (d, *J* = 9.9 Hz, 1H), 3.75 (s, 3H), 3.71 (d, *J* = 9.9 Hz, 1H), 3.43 ppm (s, 3H). **^{13}C NMR** (101 MHz, $CDCl_3$) δ 173.09, 156.27, 142.30, 131.61, 128.72, 127.20, 126.79, 121.58, 114.16, 77.66, 59.45, 55.61, 52.06, 23.69 ppm. **HR-MS:** calc. for $[M+H]^+$ $C_{18}H_{21}NO_3$ = 300.1594 found 300.1596 (-0.6 ppm). Chiral HPLC conditions: *i*-Cellulose 4.6 x 250mm column, *n*-hexane / iPrOH = 90:10, flow rate = 1 mL min^{-1}, minor enantiomer: t_R = 29.30 min; major enantiomer: t_R = 26.17 min.

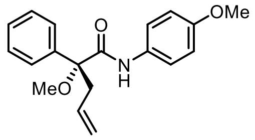

(-)-2-Methoxy-N-(4-methoxyphenyl)-2-phenylpent-4-enamide (58). Prepared according to Method C using (±)-(R)-2-methoxy-2-phenylpent-4-enenitrile (93 mg, 0.5 mmol), 4-iodoanisole (47 mg, 0.2 mmol), and *E. coli* (AJ270) whole cells; the product was obtained as a white solid (12 mg, 0.04 mmol, 20%). 78:22 e.r. $[\alpha]_D^{25}$ = -9.1 (c = 0.1 M, $CHCl_3$). **^{1}H NMR** (400 MHz, $CDCl_3$) δ 8.63 (s, 1H), 7.51 – 7.44 (m, 4H), 7.37 (t, J = 7.5 Hz, 2H), 7.33 – 7.28 (m, 1H), 6.83 (d, J = 9.0 Hz, 2H), 5.78 (m, 1H), 5.23 (dd, J = 17.1, 1.7 Hz, 1H), 5.13 (d, J = 9.3 Hz, 1H), 3.77 (s, 3H), 3.46 – 3.36 (m, 1H), 3.27 (s, 3H), 2.91 ppm (dd, J = 14.8, 7.3 Hz, 1H). **^{13}C NMR** (101 MHz, $CDCl_3$) δ 170.19, 156.50, 139.53, 132.25, 130.89, 128.69, 128.22, 126.57, 121.38, 118.90, 114.23, 84.28, 55.64, 51.16, 36.52 ppm. **HR-MS:** calc. for $[M+Na]^+$ $C_{19}H_{20}NO_3Na$ = 334.1414 found 334.1419 (-1.61 ppm). Chiral HPLC conditions: *i*-Cellulose 4.6 x 250mm column, *n*-hexane / iPrOH = 90:10, flow rate = 1 mL min^{-1}, minor enantiomer: t_R = 16.35 min; major enantiomer: t_R = 21.52 min.

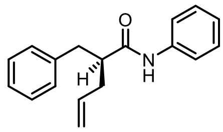

(*S*)-2-Benzyl-*N*-phenylpent-4-enamide (59). Prepared according to Method C using (±)-2-benzylpent-4-enenitrile (86 mg, 0.5 mmol), iodobenzene (22 µL, 0.2 mmol), and *E. coli* (AJ270) whole cells; the product was obtained as a white solid (22 mg, 0.09 mmol, 41%). 78:22 e.r. $[\alpha]_D^{25}$ = +8.9 (c = 0.1 M, $CHCl_3$). **^{1}H NMR** (400 MHz, $CDCl_3$) δ 7.37 – 7.20 (m, 9H), 7.10 (t, J = 7.3 Hz, 1H), 7.03 (s, 1H), 5.99 – 5.66 (m, 1H), 5.22 – 5.00 (m, 2H), 3.04 (dd, J = 13.5, 8.8 Hz, 1H), 2.88 (dd, J = 13.5, 4.9 Hz, 1H), 2.64 – 2.49 (m, 2H), 2.37 ppm (m, 1H). **^{13}C NMR** (101 MHz, $CDCl_3$) δ 172.76, 139.64, 137.56, 135.54, 129.05, 128.93, 128.69, 126.59, 124.45, 120.38, 117.53, 50.86, 38.86, 36.90 ppm. **HR-MS:** calc. for $[M+H]^+$ $C_{18}H_{20}NO$ = 266.15469 found 266.15357 (-1.01 ppm); Chiral HPLC conditions: *i*-Amylose 4.6 x 250mm column, *n*-hexane / iPrOH = 93:7, flow rate = 0.5 mL min^{-1}, minor enantiomer: t_R = 25.59 min; major enantiomer: t_R = 26.79 min.

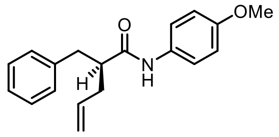

(*S*)-2-Benzyl-*N*-(4-methoxyphenyl)pent-4-enamide (60). Prepared according to Method C using (±)-2-benzylpent-4-enenitrile (86 mg, 0.5 mmol), 4-iodoanisole (47 mg, 0.2 mmol), and *E. coli* (AJ270) whole cells; the product was obtained as a white solid (44 mg, 0.15 mmol, 78%). 75:25 e.r. $[\alpha]_D^{25}$ = +24.2 (*c* = 0.1 M, $CHCl_3$). **^{1}H NMR** (400 MHz, $CDCl_3$) δ 7.27 (dd, *J* = 25.4, 7.0 Hz, 7H), 6.83 (m, 3H), 5.88 (m, 1H), 5.18 (d, *J* = 15.4 Hz, 1H), 5.12 (d, *J* = 9.3 Hz, 1H), 3.80 (s, 3H), 3.06 – 2.83 (m, 2H), 2.68 – 2.46 (m, 2H), 2.44 – 2.29 ppm (m, 1H). **^{13}C NMR** (101 MHz, $CDCl_3$) δ 172.55, 156.63, 139.78, 135.67, 130.61, 129.10, 128.70, 126.60, 122.36, 117.47, 114.13, 55.59, 50.85, 38.94, 36.95 ppm. **HR-MS:** calc. for $[M+H]^+$ $C_{19}H_{21}NO_2$ = 296.16451 found 296.16515 (-2.18 ppm); Chiral HPLC conditions: *i*-Cellulose 4.6 x 250mm column, *n*-hexane / iPrOH = 90:10, flow rate = 1 mL min^{-1}, minor enantiomer: t_R = 17.65 min; major enantiomer: t_R = 16.37 min.

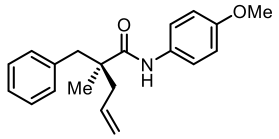

(*S*)-2-Benzyl-N-(4-methoxyphenyl)-2-methylpent-4-enamide (61). Prepared according to Method C using (±)-2-benzyl-2-methylpent-4-enenitrile (93 mg, 0.5 mmol), 4-iodoanisole (47 mg, 0.2 mmol), and *E. coli* (AJ270) whole cells; the product was obtained as a white solid (40 mg, 0.13 mmol, 65%). 85:15 e.r. $[\alpha]_D^{25}$ = +28.2 (*c* = 0.1 M, $CHCl_3$). **^{1}H NMR** (400 MHz, $CDCl_3$) δ 7.33 – 7.27 (m, 5H), 7.21 (d, *J* = 7.5 Hz, 2H), 6.98 (s, 1H), 6.89 (d, *J* = 9.0 Hz, 2H), 5.96 – 5.84 (m, 1H), 5.21 (d, *J* = 9.9 Hz, 1H), 5.18 (d, *J* = 3.3 Hz, 1H), 3.84 (s, 3H), 3.23 (d, *J* = 13.2 Hz, 1H), 2.76 (dd, *J* = 13.5, 6.4 Hz, 2H), 2.30 – 2.22 (m, 1H), 1.26 ppm (s, 3H). **^{13}C NMR** (101 MHz, $CDCl_3$) δ 174.10, 156.73, 137.71, 134.21, 130.67, 130.45, 128.25, 126.72, 122.60, 118.77, 114.19, 55.62, 47.61, 46.20, 44.46, 20.81 ppm. **HR-MS:** calc. for $[M+H]^+$ $C_{20}H_{23}NO_2$ = 310.18016 found 310.18096 (-2.6 ppm); Chiral HPLC conditions: *i*-Cellulose 4.6 x 250mm column, *n*-hexane / iPrOH = 90:10, flow rate = 1 mL min^{-1}, minor enantiomer: t_R = 17.75 min; major enantiomer: t_R = 16.54 min.

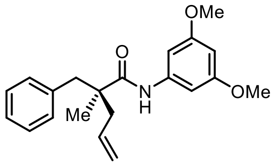

(*S*)-2-Benzyl-N-(3,5-dimethoxyphenyl)-2-methylpent-4-enamide (62). Prepared according to Method C using (±)-2-benzyl-2-methylpent-4-enenitrile (93 mg, 0.5 mmol), 1,3-dimethoxy-5-iodobenzene (53 mg, 0.2 mmol), and *E. coli* (AJ270) whole cells; the product was obtained as a white solid (52 mg, 0.15 mmol, 77%). 83:17 e.r. $[\alpha]_D^{25}$ = +14.1 (c = 0.3 M, $CHCl_3$). **^{1}H NMR** (400 MHz, Chloroform-*d*) δ 7.33 – 7.26 (m, 3H), 7.23 – 7.18 (m, 2H), 7.03 (s, 1H), 6.69 (d, J = 2.3 Hz, 2H), 6.28 (t, J = 2.2 Hz, 1H), 5.96 – 5.82 (m, 1H), 5.26 – 5.15 (m, 2H), 3.82 (s, 6H), 3.20 (d, J = 13.3 Hz, 1H), 2.81 – 2.70 (m, 2H), 2.26 (dd, J = 13.8, 8.0 Hz, 1H), 1.26 (s, 3H) ppm. **^{13}C NMR** (101 MHz, $CDCl_3$) δ 174.31, 161.09, 139.42, 137.51, 134.06, 130.43, 128.33, 126.79, 118.95, 98.54, 97.08, 55.53, 47.86, 46.13, 44.30, 20.97 ppm. **HR-MS:** calc. for $[M+H]^+$ $C_{20}H_{24}NO_3$ = 340.19174 found 340.19019 (-1.56 ppm). Chiral HPLC conditions: *i*-Cellulose 4.6 x 250mm column, *n*-hexane / iPrOH = 90:10, flow rate = 1 mL min^{-1}, minor enantiomer: t_R = 18.63 min; major enantiomer: t_R = 17.25 min.

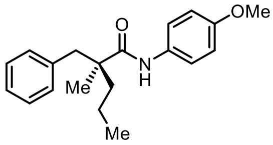

(*S*)-2-Benzyl-*N*-(4-methoxyphenyl)-2-methylpentanamide (63). Prepared according to Method C using (±)-2-benzyl-2-methylpentanenitrile (94 mg, 0.5 mmol), 4-iodoanisole (47 mg, 0.2 mmol), and *E. coli* (AJ270) whole cells; the product was obtained as a white solid (21 mg, 0.07 mmol, 33%). 87:13 e.r. $[\alpha]_D^{25}$ = +48.0 (c = 0.1 M, $CHCl_3$). **^{1}H NMR** (400 MHz, Chloroform-*d*) δ 7.26 – 7.16 (m, 5H), 7.13 (dd, J = 7.9, 1.7 Hz, 2H), 6.86 (s, 1H), 6.84 – 6.79 (m, 2H), 3.77 (s, 3H), 3.14 (d, J = 13.2 Hz, 1H), 2.64 (d, J = 13.2 Hz, 1H), 1.93 – 1.82 (m, 1H), 1.46 – 1.28 (m, 3H), 1.18 (s, 3H), 0.92 (t, J = 7.0 Hz, 3H) ppm. **^{13}C NMR** (101 MHz, $CDCl_3$) δ 174.61, 156.65, 137.91, 130.79, 130.45, 128.19, 126.62, 122.53, 114.18, 55.63, 47.88, 46.68, 42.77, 20.70, 18.12, 14.78 ppm. **HR-MS:** calc. for $[M+H]^+$ $C_{20}H_{26}NO_2$ = 312.19672 found 312.19537 (-1.39 ppm). Chiral HPLC conditions: *i*-Cellulose 4.6 x 250mm column, *n*-hexane / iPrOH = 90:10, flow rate = 1 mL min^{-1}, minor enantiomer: t_R = 15.47 min; major enantiomer: t_R = 13.64 min.

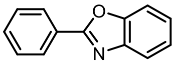

2-Phenylbenzo[*d*]oxazole (65). Prepared according to Method A using benzonitrile (21 µL, 0.2 mmol), 2-bromoiodobenzene (48 µL, 0.6 mmol), and *E. coli* (CGA009) whole cells; the product was obtained as a pale white solid (28 mg, 0.1 mmol, 51%). ^{1}**H NMR** (400 MHz, $CDCl_3$) δ 8.31 – 8.22 (m, 2H), 7.82 – 7.75 (m, 1H), 7.59 (dd, *J* = 6.0, 3.2 Hz, 1H), 7.56 – 7.50 (m, 3H), 7.36 ppm (dd, *J* = 6.0, 3.2 Hz, 2H). ^{13}**C NMR** (101 MHz, $CDCl_3$) δ 163.19, 150.91, 142.26, 131.67, 129.06, 127.77, 127.33, 125.25, 124.72, 120.17, 110.74 ppm. **HR-MS:** calc. for $[M+H]^+$ $C_{13}H_{10}NO$ = 196.07569 found 196.07609 (-2.05 ppm).

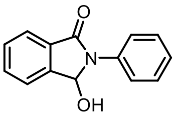

3-Hydroxy-2-phenylisoindolin-1-one (67). Prepared according to Method A using 2-cyanobenzaldehyde (26 mg, 0.2 mmol), iodobenzene (66 µL, 0.6 mmol), and *E. coli* (CGA009) whole cells; the product was obtained as a white solid (30 mg, 0.13 mmol, 67%). ^{1}**H NMR** (400 MHz, DMSO) δ 7.77 (dd, *J* = 8.6, 1.0 Hz, 3H), 7.74 – 7.65 (m, 2H), 7.61 (m, 1H), 7.44 (dd, *J* = 8.5, 7.4 Hz, 2H), 7.22 (t, *J* = 7.4 Hz, 1H), 6.84 (d, *J* = 9.9 Hz, 1H), 6.53 ppm (d, *J* = 9.9 Hz, 1H). ^{13}**C NMR** (101 MHz, DMSO) δ 165.43, 144.37, 137.45, 132.75, 131.28, 129.67, 128.66, 124.65, 123.65, 122.83, 122.30, 81.89. **HR-MS:** calc. for $[M+H]^+$ $C_{14}H_{12}NO_2$ = 226.08626 found 226.08683 (-2.55 ppm). The product matched literature characterisation.[5]

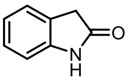

Indolin-2-one (69). Prepared according to Method A without halide, using 2-bromophenylacetonitrile (26 µL, 0.2 mmol) and *E. coli* (CGA009) whole cells; the product was obtained as a pale white solid (12 mg, 0.09 mmol, 46%). ^{1}**H NMR** (400 MHz, $CDCl_3$) δ 8.27 (s, 1H), 7.22 (t, *J* = 7.7 Hz, 2H), 7.02 (t, *J* = 7.4 Hz, 1H), 6.88 (d, *J* = 7.7 Hz, 1H), 3.55 ppm (s, 2H). ^{13}**C NMR** (101 MHz, $CDCl_3$) δ 177.46, 142.49, 128.07, 125.40, 124.82, 122.50,

109.74, 36.29 ppm. **HR-MS:** calc. for $[M+H]^+$ C_8H_8NO = 134.06004 found 134.0606 (-4.21 ppm).

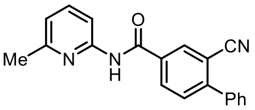

2-Cyano-N-(6-methylpyridin-2-yl)-[1,1'-biphenyl]-4-carboxamide (72). Prepared according to Method A using [1,1'-biphenyl]-2,4-dicarbonitrile (41 mg, 0.2 mmol), 2-iodo-6-methylpyridine (131 mg, 0.6 mmol), and *E. coli* (CGA) whole cells; the product was obtained as a white solid (34 mg, 0.11 mmol, 54%). ^{1}**H NMR** (400 MHz, $CDCl_3$) δ 8.68 (s, 1H), 8.35 (d, J = 1.9 Hz, 1H), 8.17 (dd, J = 8.2, 1.9 Hz, 2H), 7.71 – 7.63 (m, 2H), 7.60 (dd, J = 8.0, 1.7 Hz, 2H), 7.56 – 7.48 (m, 3H), 6.98 (d, J = 7.5 Hz, 1H), 2.49 ppm (s, 3H). ^{13}**C NMR** (101 MHz, $CDCl_3$) δ 163.37, 157.24, 150.49, 148.80, 139.11, 137.14, 133.87, 133.02, 131.31, 130.74, 129.59, 129.08, 128.86, 120.12, 117.87, 112.19, 111.31, 24.07 ppm. **HR-MS:** calc. for $[M+H]^+$ $C_{20}H_{16}N_3O$ = 314.1288 found 314.1292 (-1.31 ppm).

Connectivity was assigned via HSQC (Heteronuclear Single Quantum Coherence) NMR spectroscopy:

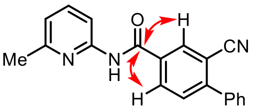

2D HMBC spectrum of **72**.

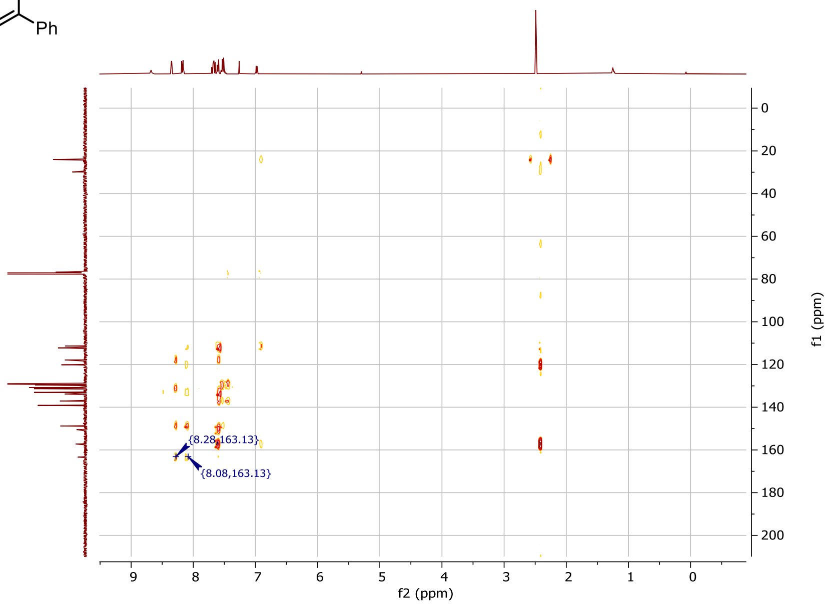

Preparation of Starting Materials

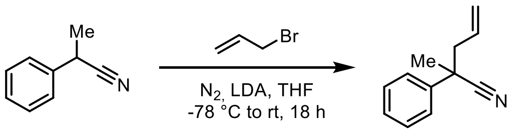

2-Methyl-2-phenylpent-4-enenitrile (54). Prepared according to a literature procedure.[8] To solution of α-methylphenylacetonitrile (266 µL, 2 mmol, 1 equiv) in dry THF (2.5 mL) was slowly added lithium diisopropylamide solution (2.4 mL, 1.0 M in THF, 1.2 equiv) under N_2 atmosphere at -78 °C. After stirring for 30 min, allyl bromide (242 µL, 2.8 mmol, 1.4 equiv) was slowly added. The resulting mixture was warmed to room temperature and stirred overnight. The reaction mixture was poured into a mixture of *n*-hexane (10 mL) and brine (10 mL) and the resulting biphasic mixture was stirred vigorously. The aqueous layer was extracted with *n*-hexane (2x10 mL) and the combined organic layers dried over anhydrous $MgSO_4$ and concentrated under reduced pressure. Silica gel column chromatography (elute: *n*-hexane / ethyl acetate) afforded the desired product as a colorless oil (149 mg, 0.87 mmol, 43%). **^{1}H NMR** (400 MHz, $CDCl_3$) δ 7.47 – 7.43 (m, 2H), 7.39 (t, *J* = 7.6 Hz, 2H), 7.32 (t, *J* = 7.2 Hz, 1H), 5.82 – 5.56 (m, 1H), 5.18 (s, 1H), 5.15 (d, *J* = 7.3 Hz, 1H), 2.65 (qd, *J* = 13.9, 7.3 Hz, 2H), 1.72 ppm (s, 3H). **^{13}C NMR** (101 MHz, $CDCl_3$) δ 139.96, 132.02, 129.01, 127.97, 125.73, 123.25, 120.30, 46.45, 42.30, 26.69 ppm.

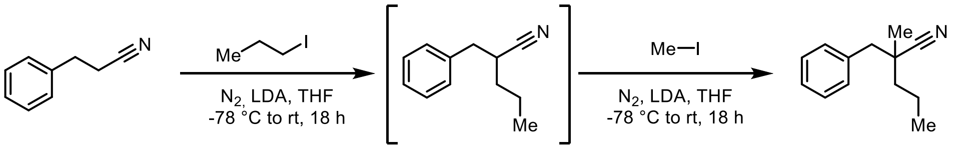

2-Benzyl-2-methylpentanenitrile (64). Prepared according to a literature procedure.[8] 1) To solution of 2-phenylacetonitrile (1.18 g, 10 mmol, 1 equiv) in dry THF (10 mL) was slowly added lithium diisopropylamide solution (10 mL, 1.0 M in THF, 1 equiv) under N_2 atmosphere at -78 °C. After stirring for 30 min, *n*-iodopropane (1.07 mL, 11 mmol, 1.1 equiv) was slowly added. The resulting mixture was warmed to room temperature and stirred overnight. The reaction mixture was poured into a mixture of *n*-hexane (50 mL) and brine (40 mL) and the resulting biphasic mixture was stirred vigorously. The aqueous layer was extracted with *n*-hexane (2x50 mL) and the combined organic layers dried over anhydrous $MgSO_4$ and concentrated under reduced pressure. The crude product was subjected to the

second alkylation step without further purification. 2) To solution of crude 2-benzylpentanenitrilein in dry THF (10 mL) was slowly added lithium diisopropylamide solution (12 mL, 1.0 M in THF, 1.2 equiv) under N_2 atmosphere at -78 °C. After stirring for 30 min, iodomethane (870 µL, 14 mmol, 1.4 equiv) was slowly added. The resulting mixture was warmed to room temperature and stirred overnight. The reaction mixture was poured into a mixture of *n*-hexane (50 mL) and brine (40 mL) and the resulting biphasic mixture was stirred vigorously. The aqueous layer was extracted with *n*-hexane (2 x 50 mL) and the combined organic layers dried over anhydrous $MgSO_4$ and concentrated under reduced pressure. Silica gel column chromatography (elute: *n*-hexane / ethyl acetate) afforded the desired product as a colorless oil (573 mg, 3.05 mmol, 31%). **^{1}H NMR** (500 MHz, Chloroform-*d*) δ 7.32 – 7.21 (m, 5H), 2.88 (d, *J* = 13.5 Hz, 1H), 2.69 (d, *J* = 13.5 Hz, 1H), 1.60 – 1.49 (m, 3H), 1.46 – 1.38 (m, 1H), 1.23 (s, 3H), 0.93 ppm (t, *J* = 7.0 Hz, 3H). **^{13}C NMR** (126 MHz, $CDCl_3$) δ 135.95, 130.72, 128.74, 127.65, 124.49, 45.77, 41.89, 38.27, 24.20, 18.72, 14.49 ppm.

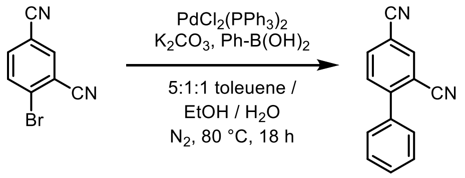

[1,1'-Biphenyl]-2,4-dicarbonitrile (70). Prepared according to a literature procedure.[9] To a suspension of 4-bromoisophthalonitrile (100 mg, 0.48 mmol), phenylboronic acid (124 mg, 0.97 mmol, 2 equiv), and K_2CO_3 (200 mg, 1.45 mmol) in a degassed mixture of toluene, EtOH and water (5:1:1, 2.5 mL total volume), was added $PdCl_2(PPh_3)_2$ (33 mg, 0.05 mmol. 0.1 equiv). The mixture was stirred at 80 °C for 18 h under inert gas atmosphere. After cooling to room temperature, the reaction was quenched with water (15 mL) and extracted with dichloromethane (2x20 mL). The combined organic layers were washed with brine, dried over $MgSO_4$, and concentrated under reduced pressure. Silica gel column chromatography (elute: *n*-hexane / ethyl acetate) afforded the desired product as a white solid (90 mg, 0.44 mmol, 90%). **^{1}H NMR** (400 MHz, $CDCl_3$) δ 8.06 (d, *J* = 1.8 Hz, 1H), 7.91 (dd, *J* = 8.2, 1.8 Hz, 1H), 7.67 (d, *J* = 8.2 Hz, 1H), 7.60 – 7.50 ppm (m, 5H). **^{13}C NMR** (101 MHz, $CDCl_3$) δ 149.69, 137.26, 136.47, 135.88, 131.27, 130.13, 129.27, 128.77, 116.87, 116.77, 113.00, 112.33 ppm.

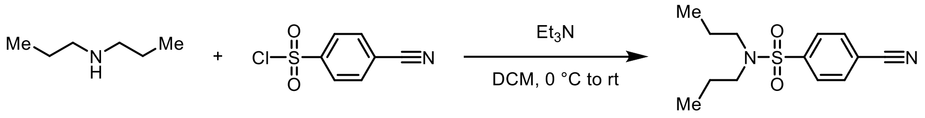

4-Cyano-*N*,*N*-dipropylbenzenesulfonamide (73). Prepared according to a literature procedure.[10] To a solution of 4-cyanobenzenesulfonyl chloride (5.00 g, 25.0 mmol, 1 equiv) and dipropylamine (5.13 mL, 37.5 mmol, 1.5 equiv) in dichloromethane (65 mL) was added Et_3N (6.98 mL, 50 mmol, 2 equiv) at 0 °C. After addition, the reaction was stirred at room temperature overnight. The reaction was concentrated under reduced pressure and silica gel column chromatography (elute: *n*-hexane / ethyl acetate) afforded the desired product as an off white solid. (6.2 g, 23.3 mmol, 93%). **^{1}H NMR** (400 MHz, Chloroform-d) δ 7.87 – 7.83 (m, 2H), 7.75 – 7.71 (m, 2H), 3.08 – 3.00 (m, 4H), 1.56 – 1.42 (m, 4H), 0.80 ppm (t, *J* = 7.4 Hz, 6H); **^{13}C NMR** (100 MHz, Chloroform-*d*) δ 144.80, 1332.098, 127.73, 117.54, 116.10, 50.061, 22.108, 11.27 ppm.

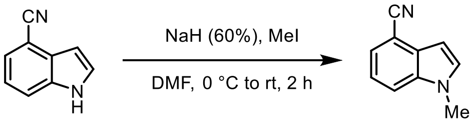

1-Methyl-1*H*-indole-4-carbonitrile (74). Prepared according to a literature procedure.[11] To a solution of 1*H*-indole-4-carbonitrile (284 mg, 2 mmol, 1 equiv) in anhydrous DMF (5 mL) was added sodium hydride (60% in mineral oil, 92 mg, 2.3 mmol, 1.15 equiv) at 0 °C and the reaction was stirred for 30 min. After that time, iodomethane (187 µL, 3 mmol, 1.5 equiv) was added dropwise and the reaction mixture was allowed to warm to room temperature and stirring was continued for 2 h. Next, the reaction was slowly quenched with water (25 mL) and the aqueous layer was extracted with ethyl acetate (3 x 25 mL), the combined organic layers were washed with brine, dried over $MgSO_4$ and concentrated under reduced pressure. Silica gel column chromatography (elute: *n*-hexane / ethyl acetate) afforded the desired product as a white solid (204 mg, 1.31 mmol, 65%). **^{1}H NMR** (400 MHz, DMSO-d_6) δ 7.83 (d, *J* = 8.3 Hz, 1H), 7.63 (d, *J* = 3.2 Hz, 1H), 7.55 (d, *J* = 7.3 Hz, 1H), 7.35 – 7.23 (m, 1H), 6.58 (d, *J* = 3.0 Hz, 1H), 3.87 ppm (s, 3H). **^{13}C NMR** (101 MHz, DMSO) δ 136.04, 132.97, 128.74, 124.56, 120.91, 118.62, 115.32, 101.29, 98.57, 32.80 ppm.

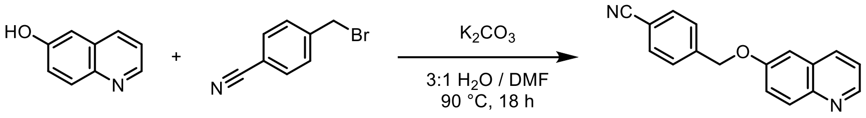

4-((Quinolin-6-yloxy)methyl)benzonitrile (75). Prepared according to a literature procedure.[12] To a solution of 6-hydroxyquinoline (290 mg, 2 mmol, 1 equiv) and 4-cyanobenzyl bromide (431 mg, 2.2 mmol, 1.1 equiv) in 3:1 water / DMF (4 mL) was added K_2CO_3 (553 mg, 4 mmol, 2 equiv) and the reaction was stirred at 90 °C for 18 h. After cooling to room temperature, the reaction was quenched with water (25 mL) and the aqueous layer was extracted with ethyl acetate (3 x 25 mL), the combined organic layers were washed with brine, dried over $MgSO_4$ and concentrated under reduced pressure. Silica gel column chromatography (elute: *n*-hexane / ethyl acetate) afforded the desired product as a white solid (430 mg, 1.65 mmol, 83%). **^{1}H NMR** (400 MHz, DMSO) δ 8.75 (dd, *J* = 4.2, 1.6 Hz, 1H), 8.23 (dd, *J* = 8.3, 1.6 Hz, 1H), 7.96 (d, *J* = 9.1 Hz, 1H), 7.89 (d, *J* = 8.3 Hz, 2H), 7.71 (d, *J* = 8.3 Hz, 2H), 7.55 – 7.42 (m, 3H), 5.36 ppm (s, 2H). **^{13}C NMR** (101 MHz, DMSO) δ 155.82, 148.19, 143.91, 142.46, 134.80, 132.45, 130.56, 128.87, 128.17, 122.08, 121.74, 118.73, 110.60, 107.17, 68.60 ppm.

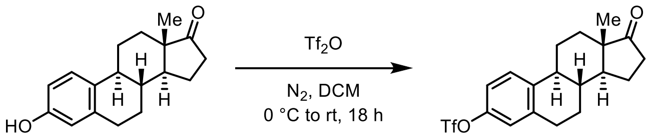

(8*R*,9*S*,13*S*,14*S*)-13-methyl-17-oxo-7,8,9,11,12,13,14,15,16,17-decahydro-6*H*-cyclopenta[*a*]phenanthren-3-yl trifluoromethanesulfonate (76). Prepared according to a literature procedure.[13] To a solution of estrone (676 mg, 2.5 mmol, 1.0 equiv) in dichloromethane (10 mL) was added pyridine (404 μL, 5.0 mmol, 2.0 equiv) under N_2 atmosphere and the reaction mixture was cooled to 0 °C. Trifluoromethanesulfonic anhydride (505 μL, 3.0 mmol, 1.2 equiv) was added dropwise and the reaction was left stirring for 18 h. The reaction mixture was washed with 2 M HCl (15 mL) prior to extraction of the aqueous layer with dichloromethane (2x15 mL). The combined organic layers were washed with brine, dried over $MgSO_4$ and concentrated under reduced pressure. Silica gel column chromatography (elute: *n*-hexane / ethyl acetate) afforded the desired product as a white solid (877 mg, 2.18 mmol, 87%). **^{1}H NMR** (400 MHz, Chloroform-*d*) δ 7.34 (d, *J* = 8.6 Hz, 1H), 7.03 (dd, *J* = 8.6, 2.7 Hz, 1H), 6.99 (d, *J* = 2.6 Hz, 1H), 2.94 (dd, *J* = 9.1, 4.3 Hz, 2H), 2.52 (dd, *J* = 18.9, 8.7 Hz, 1H), 2.40 (m, 1H), 2.30 (m, 1H), 2.21 – 2.13 (m, 1H), 2.11 – 2.01 (m,

2H), 2.01 – 1.94 (m, 1H), 1.63 (m, 2H), 1.57 (s, 1H), 1.55 – 1.42 (m, 3H), 0.92 ppm (s, 3H); **^{13}C NMR** (100 MHz, Chloroform-*d*) δ Carbonyl peak out of acquisition range. 147.72, 140.41, 139.43, 127.33, 121.38, 118.45, 117.29 (d, J_{CF} = 321 Hz), 50.53, 48.00, 44.24, 37.90, 35.95, 31.63, 29.54, 26.24, 25.83, 21.72, 13.95 ppm.

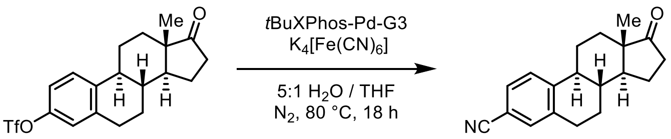

(8*R*,9*S*,13*S*,14*S*)-13-methyl-17-oxo-7,8,9,11,12,13,14,15,16,17-decahydro-6*H*-cyclopenta[*a*]phenanthrene-3-carbonitrile (77). Prepared according to a literature procedure.[14] (8*R*,9*S*,13*S*,14*S*)-13-Methyl-17-oxo-7,8,9,11,12,13,14,15,16,17-decahydro-6*H*-cyclopenta[*a*]phenanthren-3-yl trifluoromethanesulfonate (201 mg, 0.5 mmol, 1.0 equiv), $K_4[Fe(CN)_6]$ (106 mg, 0.25 mmol, 0.5 equiv) and *t*BuXPhos-Pd-G3 (40 mg, 0.05 mmol, 0.1 equiv) were added to a 4 mL screwcap vial fitted with a septum cap prior to evacuation and backfill with N_2. Degassed water (2.5 mL) and degassed THF (0.5 mL) were added prior to heating at 80 °C with vigorous stirring for 18 h. After cooling to room temperature, the reaction mixture was diluted with water (10 mL) and extracted with 1:1 ethyl acetate / dichloromethane (3x10 mL), the combined organic layers were washed with brine, dried over $MgSO_4$ and concentrated under reduced pressure. Silica gel column chromatography (elute: *n*-hexane / ethyl acetate) afforded the desired product as a white solid (126 mg, 0.45 mmol, 90%). **^{1}H NMR** (400 MHz, Chloroform-*d*) δ 7.42 (d, *J* = 8.4 Hz, 1H), 7.37 (d, *J* = 7.6 Hz, 2H), 2.93 (m, 2H), 2.52 (dd, *J* = 18.8, 8.7 Hz, 1H), 2.45 – 2.38 (m, 1H), 2.33 (m, 1H), 2.17 (q, *J* = 9.7, 9.3 Hz, 1H), 2.13 – 2.01 (m, 2H), 2.01 – 1.95 (m, 1H), 1.75 – 1.41 (m, 6H), ppm 0.92 (s, 3H). **^{13}C NMR** (100 MHz, Chloroform-*d*) δ Carbonyl peak out of acquisition range. 145.48, 138.02, 132.67, 129.47, 126.33, 119.29, 109.76, 50.60, 47.96, 44.70, 37.73, 35.91, 31.60, 29.11, 26.12, 25.58, 21.70, 13.93 ppm.

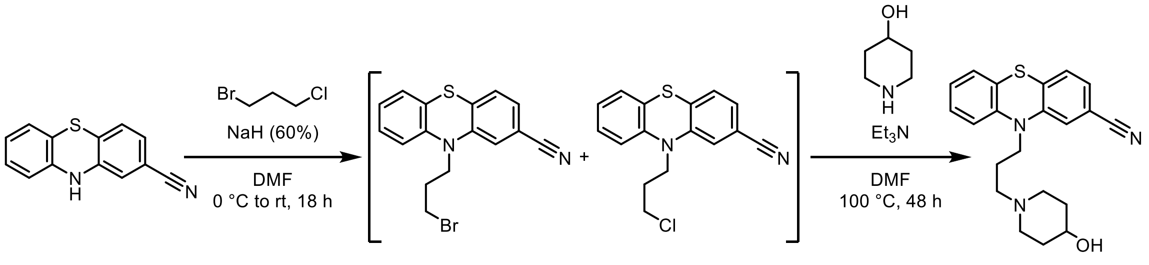

10-(3-(4-hydroxypiperidin-1-yl)propyl)-10*H*-phenothiazine-2-carbonitrile (Periciazine) (78). Prepared according to a literature procedure.[15] To a solution of 10*H*-Phenothiazine-2-carbonitrile (897 mg, 4 mmol, 1 equiv) in DMF (10 mL) was slowly added sodium hydride (60 % in mineral oil, 480 mg, 12.0 mmol, 3 equiv) under inert gas atmosphere. The reaction mixture was cooled to 0°C and 1-bromo-3-chloropropane (789 µL, 8 mmol, 2 equiv) was added dropwise and the reaction mixture was stirred at room temperature for 18 h. The reaction was quenched by pouring over ice bath cooled brine (100 mL) prior to extraction with diethyl ether (3x75 mL). The combined organic layers were washed with brine and dried over $MgSO_4$ and concentration under reduced pressure. The crude intermediate was recrystallized from *n*-hexane / ethyl acetate to yield a regioisomeric mixture of 10-(2-bromoethyl)-10*H*-phenothiazine-2-carbonitrile / 10-(2-chloroethyl)-10*H*-phenothiazine-2-carbonitrile as yellow crystals (664 mg, 2.23 mmol, 58%). The crude mixture was used without further purification. To a solution of the crude intermediate was added 4-piperidinol (468 mg, 4.36 mmol, 2.0 equiv) and Et_3N (801 µL, 5.75 mmol, 2.5 equiv) in DMF (5 mL) and the reaction was stirred at 100°C for 48 h. After that time. the reaction was cooled to room temperature, mixed with water (20 mL) and extracted with ethyl acetate (3x20 mL), the combined organics were washed with brine (20 mL) and dried over $MgSO_4$ and concentrated under reduced pressure. Silica gel column chromatography (elute: dichloromethane / MeOH) afforded the desired product as a yellow solid (712 mg, 1.95 mmol, 49%). **^{1}H NMR** (400 MHz, Chloroform-*d*) δ 7.16 (d, *J* = 11.3 Hz, 3H), 7.11 – 7.07 (m, 1H), 7.06 (s, 1H), 6.96 (d, *J* = 7.5 Hz, 1H), 6.93 – 6.89 (m, 1H), 3.90 (t, *J* = 6.9 Hz, 2H), 3.67 (tt, *J* = 8.9, 4.2 Hz, 1H), 2.72 (m, 2H), 2.45 (t, *J* = 6.9 Hz, 2H), 2.13 (t, *J* = 10.7 Hz, 2H), 1.93 (q, *J* = 7.0 Hz, 2H), 1.86 (m, 2H), 1.54 ppm (m, 2H); **^{13}C NMR** (100 MHz, Chloroform-*d*) δ 145.88, 143.98, 132.20, 128.02, 127.83, 127.66, 126.00, 123.49, 123.46, 119.20, 117.93, 116.11, 110.82, 68.09, 55.38, 51.43, 45.68, 34.66, 24.57 ppm.

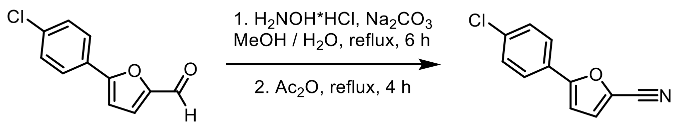

5-(4-Chlorophenyl)furan-2-carbonitrile (79). Prepared according to a literature procedure.[16] To a stirred solution of 5-(4-chlorophenyl)furfural (413 mg, 2 mmol, 1 equiv) in methanol (10 mL) was slowly added an aqueous solution (8 mL) of hydroxylamine hydrochloride (280 mg, 4 mmol, 2 equiv) and sodium carbonate (424 mg, 4 mmol, 2 equiv). Afterwards, the reaction mixture was refluxed for 6 h. After cooling to room temperature, excess of solvent was removed under reduced pressure, the precipitate was partitioned between water (50 mL) and ethyl acetate (150 mL), and the organic layer was dried over $MgSO_4$ and then concentrated under reduced pressure. Next, the crude oxime was refluxed in acetic anhydride (8 mL) for 4 h. After cooling to room temperature, the reaction mixture was poured slowly onto ice-water (50 mL) and the precipitate was filtered, washed with water and dried under reduced pressure. The product was obtained as orange solid (327 mg, 1.61 mmol, 80%). **^{1}H NMR** (400 MHz, DMSO) δ 7.85 (d, *J* = 8.5 Hz, 2H), 7.72 (d, *J* = 3.8 Hz, 1H), 7.56 (d, *J* = 8.5 Hz, 2H), 7.31 ppm (d, *J* = 3.8 Hz, 1H). **^{13}C NMR** (101 MHz, DMSO) δ 156.93, 134.18, 129.25, 127.08, 126.49, 125.73, 124.31, 111.96, 108.19 ppm.

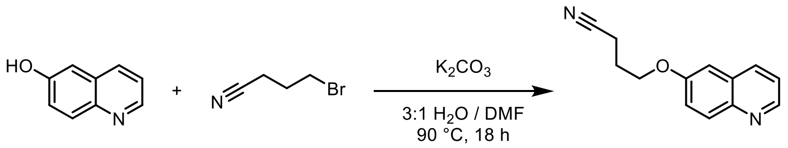

4-(Quinolin-6-yloxy)butanenitrile (80). Prepared according to a literature procedure.[12] To a solution of 6-hydroxyquinoline (290 mg, 2 mmol, 1 equiv) and 4-bromobutyronitrile (219 µL, 2.2 mmol, 1.1 equiv) in 3:1 water / DMF (4 mL) was added K_2CO_3 (553 mg, 4 mmol, 2 equiv) and the reaction was stirred at 90 °C for 18 h. After cooling to room temperature, the reaction was quenched with water (25 mL) and the aqueous layer was extracted with ethyl acetate (3 x 25 mL), the combined organic layers were washed with brine, dried over $MgSO_4$ and concentrated under reduced pressure. Silica gel column chromatography (elute: *n*-hexane / ethyl acetate) afforded the desired product as a white solid (270 mg, 1.27 mmol, 64%). **^{1}H NMR** (400 MHz, DMSO) δ 8.74 (dd, *J* = 4.2, 1.7 Hz, 1H), 8.24 (dd, *J* = 8.5, 1.7 Hz, 1H), 7.93 (d, *J* = 8.9 Hz, 1H), 7.50 – 7.41 (m, 1H), 7.42 – 7.31 (m, 2H), 4.18 (t, *J* = 6.1 Hz, 2H), 2.71 (t, *J* = 7.1 Hz, 2H), 2.18 – 2.04 ppm (m, 2H). **^{13}C NMR** (101 MHz, DMSO) δ 156.12,

148.02, 143.84, 134.78, 130.42, 128.97, 122.05, 121.65, 120.24, 106.53, 66.15, 24.60, 13.45 ppm.

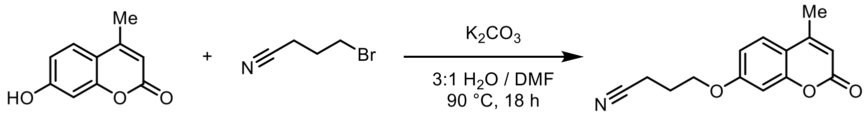

4-((4-Mmethyl-2-oxo-2*H*-chromen-7-yl)oxy)butanenitrile (81). Prepared according to a literature procedure.[12] To a solution of 4-methylumbelliferone (352 mg, 2 mmol, 1 equiv) and 4-bromobutyronitrile (219 µL, 2.2 mmol, 1.1 equiv) in 3:1 water / DMF (4 mL) was added K_2CO_3 (553 mg, 4 mmol, 2 equiv) and the reaction was stirred at 90 °C for 18 h. After cooling to room temperature, the reaction was quenched with water (25 mL) and the aqueous layer was extracted with ethyl acetate (3 x 25 mL), the combined organic layers were washed with brine, dried over $MgSO_4$ and concentrated under reduced pressure. Silica gel column chromatography (elute: *n*-hexane / ethyl acetate) afforded the desired product as a white solid (190 mg, 0.78 mmol, 39). **^{1}H NMR** (400 MHz, $CDCl_3$) δ 7.50 (d, *J* = 8.8 Hz, 1H), 6.85 (dd, *J* = 8.8, 2.5 Hz, 1H), 6.79 (d, *J* = 2.5 Hz, 1H), 6.13 (d, *J* = 1.1 Hz, 1H), 4.14 (t, *J* = 5.7 Hz, 2H), 2.61 (t, *J* = 7.1 Hz, 2H), 2.39 (d, *J* = 1.1 Hz, 3H), 2.26 – 2.11 ppm (m, 2H). **^{13}C NMR** (101 MHz, $CDCl_3$) δ 161.36, 161.21, 155.28, 152.55, 125.82, 119.01, 114.13, 112.38 (2xC), 101.67, 65.94, 25.32, 18.78, 14.31 ppm.

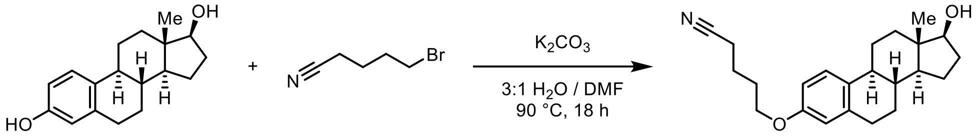

5-(((**8*R*,9*S*,13*S*,14*S*,17*S*)-17-Hydroxy-13-methyl-7,8,9,11,12,13,14,15,16,17-decahydro-6*H*-cyclopenta[*a*]phenanthren-3-yl)oxy)pentanenitrile (82).** Prepared according to a literature procedure.[12] To a solution of estradiol (544 mg, 2 mmol, 1 equiv) and 5-bromovaleronitrile (303 µL, 2.6 mmol, 1.3 equiv) in 3:1 water / DMF (4 mL) was added K_2CO_3 (553 mg, 4 mmol, 2 equiv) and the reaction was stirred at 90 °C for 18 h. After cooling to room temperature, the reaction was quenched with water (25 mL) and the aqueous layer was extracted with ethyl acetate (3 x 25 mL), the combined organic layers were washed with brine, dried over $MgSO_4$ and concentrated under reduced pressure. Silica gel column chromatography (elute: *n*-hexane / ethyl acetate) afforded the desired product as a white solid

(374 mg, 1.06 mmol, 53%). **^{1}H NMR** (400 MHz, $CDCl_3$) δ 7.20 (dd, *J* = 8.6, 1.0 Hz, 1H), 6.69 (dd, *J* = 8.6, 2.8 Hz, 1H), 6.62 (d, *J* = 2.8 Hz, 1H), 3.98 (t, *J* = 5.5 Hz, 2H), 3.73 (dd, *J* = 8.9, 8.0 Hz, 1H), 2.92 – 2.77 (m, 2H), 2.44 (t, *J* = 6.8 Hz, 2H), 2.36 – 2.25 (m, 1H), 2.24 – 2.05 (m, 2H), 1.91 (m, 6H), 1.76 – 1.64 (m, 1H), 1.57 – 1.13 (m, 8H), 0.78 ppm (s, 3H). **^{13}C NMR** (101 MHz, $CDCl_3$) δ 156.66, 138.19, 133.05, 126.50, 119.67, 114.54, 112.02, 82.00, 66.66, 50.14, 44.06, 43.37, 38.95, 36.82, 30.70, 29.91, 28.33, 27.35, 26.44, 23.25, 22.61, 17.10, 11.18 ppm.

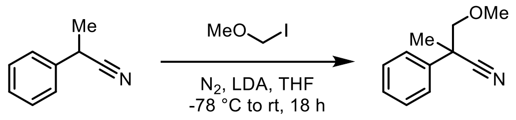

3-Methoxy-2-methyl-2-phenylpropanenitrile (83). Prepared according to a literature procedure.[8] To solution of *α*-methylphenylacetonitrile (331 µL, 2.5 mmol, 1 equiv) in dry THF (4 mL) was slowly added lithium diisopropylamide solution (2.5 mL, 1.0 M in THF, 1.0 equiv) under N_2 atmosphere at -78 °C. After stirring for 30 min, iodomethyl methyl ether (233 µL, 2.75 mmol, 1.1 equiv) was slowly added. The resulting mixture was warmed to room temperature and stirred overnight. The reaction mixture was poured into a mixture of *n*-hexane (15 mL) and brine (15 mL) and the resulting biphasic mixture was stirred vigorously. The aqueous layer was extracted with *n*-hexane (2x15 mL) and the combined organic layers dried over anhydrous $MgSO_4$ and concentrated under reduced pressure. Silica gel column chromatography (elute: *n*-hexane / ethyl acetate) afforded the desired product as a colorless oil (251 mg, 1.43 mmol, 57%). **^{1}H NMR** (400 MHz, $CDCl_3$) δ 7.48 (dd, *J* = 7.4, 2.0 Hz, 2H), 7.40 (t, *J* = 7.4 Hz, 2H), 7.37 – 7.31 (m, 1H), 3.67 – 3.54 (m, 2H), 3.41 (s, 3H), 1.75 ppm (s, 3H). **^{13}C NMR** (101 MHz, $CDCl_3$) δ 137.93, 129.05, 128.33, 126.11, 122.74, 79.10, 59.78, 43.39, 23.49 ppm.

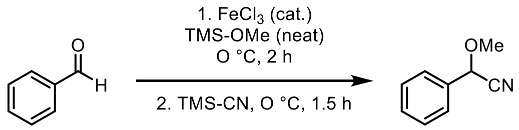

2-Methoxy-2-phenylacetonitrile (84). Prepared according to a literature procedure.[17] To a solution of methoxytrimethylsilane (1.65 mL, 12 mmol, 2.4 equiv) and $FeCl_3$ (16 mg, 0.1 mmol, 0.02 equiv) was added benzaldehyde (0.51 mL, 5 mmol, 1 equiv) and the mixture

was stirred at 0 °C for 2 h. After that time, trimethylsilyl cyanide (0.94 mL, 7.5 mmol, 1.5 equiv) was added to the mixture and the resulting mixture was stirred at 0 °C for 1.5 h. Saturated $NaHCO_3$ solution (25 mL) was added to the mixture and the product was extracted with dichloromethane (3x25 mL). The combined organic layers were dried over $MgSO_4$ and concentrated under reduced pressure. Silica gel column chromatography yield the desired product as colorless oil (676 mg, 4.6 mmol, 92%). **^{1}H NMR** (400 MHz, $CDCl_3$) δ 7.53 – 7.41 (m, 5H), 5.21 (s, 1H), 3.54 ppm (s, 3H). **^{13}C NMR** (101 MHz, $CDCl_3$) δ 133.39, 129.99, 129.17, 127.44, 117.08, 72.44, 57.35 ppm.

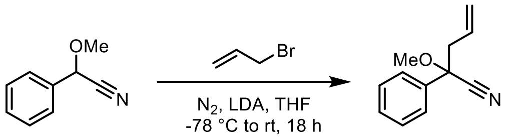

2-Methyl-2-phenylpent-4-enenitrile (85). Prepared according to a literature procedure.[8] To solution of 2-methoxy-2-phenylacetonitrile (588 mg, 4 mmol, 1 equiv) in dry THF (8 mL) was slowly added lithium diisopropylamide solution (4.4 mL, 1.0 M in THF, 1.1 equiv) under N_2 atmosphere at -78 °C. After stirring for 30 min, allyl bromide (415 µL, 4.8 mmol, 1.2 equiv) was slowly added. The resulting mixture was warmed to room temperature and stirred overnight. The reaction mixture was poured into a mixture of *n*-hexane (25 mL) and brine (20 mL) and the resulting biphasic mixture was stirred vigorously. The aqueous layer was extracted with *n*-hexane (2x25 mL) and the combined organic layers dried over anhydrous $MgSO_4$ and concentrated under reduced pressure. Silica gel column chromatography (elute: *n*-hexane / ethyl acetate) afforded the desired product as a colorless oil (476 mg, 2.54 mmol, 64%). **^{1}H NMR** (400 MHz, $CDCl_3$) δ 7.55 – 7.36 (m, 5H), 5.71 (m, 1H), 5.24 – 5.10 (m, 2H), 3.29 (s, 3H), 2.89 – 2.63 ppm (m, 2H). **^{13}C NMR** (101 MHz, $CDCl_3$) δ 136.57, 130.65, 129.39, 129.02, 126.10, 120.73, 118.04, 82.27, 54.32, 47.65 ppm.

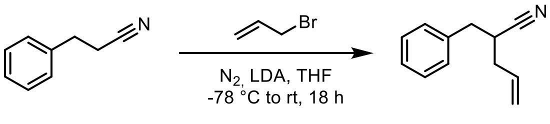

2-Benzylpent-4-enenitrile (86). Prepared according to a literature procedure.[8] To solution of 2-phenylacetonitrile (2.35 g, 20 mmol, 1 equiv) in dry THF (25 mL) was slowly added lithium diisopropylamide solution (20 mL, 1.0 M in THF, 1 equiv) under N_2 atmosphere at -

78 °C. After stirring for 30 min, allyl bromide (1.9 mL, 22 mmol, 1.1 equiv) was slowly added. The resulting mixture was warmed to room temperature and stirred overnight. The reaction mixture was poured into a mixture of *n*-hexane (50 mL) and brine (40 mL) and the resulting biphasic mixture was stirred vigorously. The aqueous layer was extracted with *n*-hexane (2x50 mL) and the combined organic layers dried over anhydrous $MgSO_4$ and concentrated under reduced pressure. Silica gel column chromatography (elute: *n*-hexane / ethyl acetate) afforded the desired product as a colorless oil (1.65 g, 5.84 mmol, 29%). **^{1}H NMR** (400 MHz, $CDCl_3$) δ 7.26 – 7.12 (m, 5H), 5.84 – 5.68 (m, 1H), 5.14 (d, *J* = 1.5 Hz, 1H), 5.13 – 5.08 (m, 1H), 2.89 – 2.70 (m, 3H), 2.25 ppm (m, 2H). **^{13}C NMR** (101 MHz, $CDCl_3$) δ 136.92, 133.05, 129.17, 128.90, 127.42, 121.37, 119.39, 37.85, 35.82, 33.68 ppm.

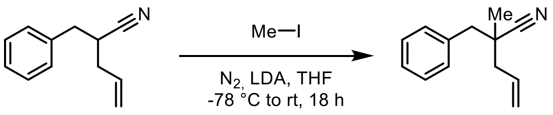

2-Benzyl-2-methylpent-4-enenitrile (87). Prepared according to a literature procedure.[8] To solution of 2-benzylpent-4-enenitrile (342 mg, 2 mmol, 1 equiv) in dry THF (2.5 mL) was slowly added lithium diisopropylamide solution (2.4 mL, 1.0 M in THF, 1.2 equiv) under N_2 atmosphere at -78 °C. After stirring for 30 min, iodomethane (174 µL, 2.8 mmol, 1.4 equiv) was slowly added. The resulting mixture was warmed to room temperature and stirred overnight. The reaction mixture was poured into a mixture of *n*-hexane (10 mL) and brine (10 mL) and the resulting biphasic mixture was stirred vigorously. The aqueous layer was extracted with *n*-hexane (2x10 mL) and the combined organic layers dried over anhydrous $MgSO_4$ and concentrated under reduced pressure. Silica gel column chromatography (elute: *n*-hexane / ethyl acetate) afforded the desired product as a colorless oil (165 mg, 0.89 mmol, 44%). **^{1}H NMR** (400 MHz, $CDCl_3$) δ 7.44 – 7.14 (m, 5H), 6.06 – 5.79 (m, 1H), 5.27 (d, *J* = 10.1 Hz, 1H), 5.22 (d, *J* = 17.0 Hz, 1H), 2.93 (d, *J* = 13.5 Hz, 1H), 2.74 (d, *J* = 13.5 Hz, 1H), 2.41 (dd, *J* = 13.8, 7.0 Hz, 1H), 2.25 (dd, *J* = 13.7, 7.6 Hz, 1H), 1.27 ppm (s, 3H). ^{13}C NMR (101 MHz, $CDCl_3$) δ 135.50, 132.18, 130.47, 128.55, 127.50, 123.70, 120.37, 45.07, 43.65, 37.81, 23.72 ppm.

Preparation of Racemic Standards

Method D: Synthesis of racemic standards

Conducted according to a literature procedure.[18, 19] Step 1) To a solution of carboxylic acid (2 mmol, 1 equiv) in dry THF (2.5 mL) was slowly added lithium diisopropylamide solution (4.4 mL, 1.0 M in THF, 2.2 equiv) under N_2 atmosphere at 0 °C. After stirring for 30 min, the alkyl halide (2.2 mmol, 1.1 equiv) was slowly added. The resulting mixture was warmed to room temperature and stirred for 18 h. After that time, the solvent was removed under reduced pressure. The resulting residue was diluted with water (10 mL) and extracted with ether (3 x 10 mL). The pH of the separated aqueous layer was adjusted to 2 using 6 M HCl and then extracted with ether (3 x 10 mL). The combined organic layers were washed with brine, dried over $MgSO_4$ and the solvent was removed under reduced pressure. The crude product was used in the next step without further purification. Step 2) To a solution of crude alkylated carboxylic acid in dry dichloromethane (3.5 mL) was successively added aniline (2.2 mmol, 1.1 equiv), *N*-(3-dimethylaminopropyl)-*N′*-ethylcarbodiimide hydrochloride (462 mg, 2.6 mmol, 1.3 equiv), and *N,N*-Dimethylaminopyridine (49 mg, 0.4 mmol, 0.2 equiv) and the reaction was stirred at room temperature for 18 h. The solution was diluted with dichloromethane (20 mL), washed with 2 M HCl (20 mL), sat. $NaHCO_3$ (20 mL) and brine (20 mL), dried over $MgSO_4$ and concentrated under reduced pressure. Silica gel column chromatography (elute: *n*-hexane / ethyl acetate) afforded the desired products.

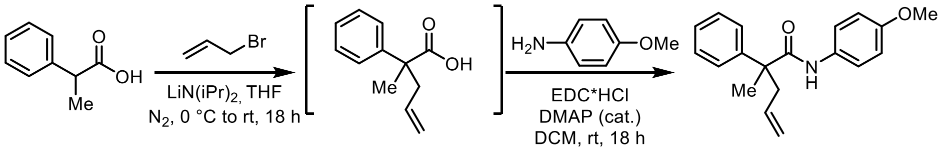

N-(4-methoxyphenyl)-2-methyl-2-phenylpent-4-enamide (rac-56). Prepared according to Method D using 2-phenylpropionic acid (150 mg, 1 mmol); the product was obtained as a white solid (236 mg, 0.78 mmol, 79%). **^{1}H NMR** (400 MHz, $CDCl_3$) δ 7.36 – 7.21 (m, 5H), 7.18 (d, *J* = 9.5 Hz, 2H), 6.73 (d, *J* = 8.9 Hz, 2H), 6.70 (s, 1H), 5.60 – 5.44 (m, 1H), 5.02 (d, *J* = 17.0 Hz, 1H), 4.97 (d, *J* = 11.2 Hz, 1H), 3.69 (s, 3H), 2.84 – 2.71 (m, 2H), 1.55 ppm (s, 3H). **^{13}C NMR** (101 MHz, $CDCl_3$) δ 174.89, 156.57, 143.18, 134.12, 130.99, 129.04, 127.51, 127.16, 121.94, 118.72, 114.17, 55.61, 51.11, 43.75, 23.95 ppm.

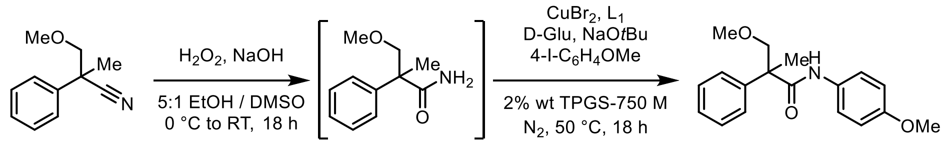

3-Methoxy-*N*-(4-methoxyphenyl)-2-methyl-2-phenylpropanamide (rac-57). Conduced according to a literature procedure.[6] Step 1) To a stirred solution of 3-methoxy-2-methyl-2-phenylpropanenitrile (150 mg, 0.86 mmol, 1 equiv) in 5:1 EtOH (4 mL) and DMSO (0.8 mL), H_2O_2 (0.88 mL, 8.67 mmol, 10 equiv) was added dropwise at 0 °C and the mixture was stirred for 30 minutes. Then, 1 M NaOH aqueous solution (1.73 mL, 1.73 mmol, 2 equiv) was added and the mixture was stirred for 18 h at room temperature. After that time, the resulting residue was diluted with water (10 mL) and extracted with ethyl acetate (3x20 mL). The combined organic layers were dried over $MgSO_4$ and concentrated under reduced pressure. Step 2) The crude primary amide was dissolved in TPGS-750 M (2 mL) and halide (608 mg, 2.4 mmol, 3 equiv), ligand (28 µL, 0.17 mmol, 20 mol%), D-Glu (31 mg, 0.17 mmol, 20 mol%), and $CuBr_2$ (19 mg, 0.09 mmol, 10 mol%) were added successively. Excess of oxygen was removed via 10s headspace purge. NaO*t*Bu (166 mg, 1.73 mmol, 2 equiv) was added to the reaction mixture and the reaction was vigorously stirred (1200 rpm) at 50 °C for 24 h. After cooling to room temperature, the reaction was diluted with ethyl acetate and filtered through a pad of Celite©. The solution was washed with brine (20 mL), the aqueous phase was extracted with ethyl acetate (2x25 mL), and the combined organic phases were dried over anhydrous $MgSO_4$ and concentrated under reduced pressure. Silica gel column chromatography (elute: *n*-hexane / ethyl acetate) afforded the desired product as a colorless oil (207 mg, 0.69 mmol, 80%). **^{1}H NMR** (400 MHz, $CDCl_3$) δ 8.33 (s, 1H), 7.43 – 7.32 (m, 6H), 7.28 (d, J = 7.0 Hz, 1H), 6.86 – 6.81 (m, 2H), 3.97 (d, J = 9.9 Hz, 1H), 3.78 (s, 3H), 3.74 (d, J = 9.9 Hz, 1H), 3.46 (s, 3H), 1.53 ppm (s, 3H). **^{13}C NMR** (101 MHz, $CDCl_3$) δ 173.09, 156.27, 142.30, 131.62, 128.72, 127.20, 126.79, 121.58, 114.16, 77.65, 59.45, 55.61, 52.06, 23.70 ppm.

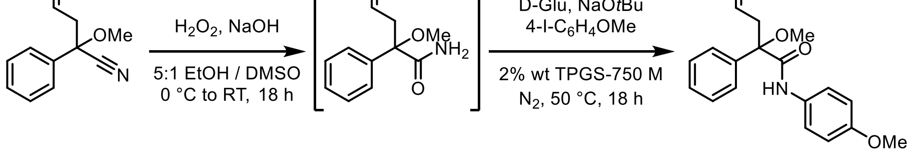

2-Methoxy-*N*-(4-methoxyphenyl)-2-phenylpent-4-enamide (rac-58). Conduced according to a literature procedure.[6] Step 1) To a stirred solution of 2-methoxy-2-phenylpent-4-

enenitrile (187 mg, 1 mmol, 1 equiv) in 5:1 EtOH (5 mL) and DMSO (1 mL), H_2O_2 (1.02 mL, 10 mmol, 10 equiv) was added dropwise at 0 °C and the mixture was stirred for 30 minutes. Then, 1 M NaOH aqueous solution (2 mL, 2 mmol, 2 equiv) was added and the mixture was stirred for 18 h at room temperature. After that time, the resulting residue was diluted with water (10 mL) and extracted with ethyl acetate (3x20 mL). The combined organic layers were dried over $MgSO_4$ and concentrated under reduced pressure. Step 2) The crude primary amide was dissolved in TPGS-750 M (2.5 mL) and halide (702 mg, 3 mmol, 3 equiv), ligand (31 µL, 0.2 mmol, 20 mol%), D-Glu (36 mg, 0.2 mmol, 20 mol%), and $CuBr_2$ (22 mg, 0.1 mmol, 10 mol%) were added successively. Excess of oxygen was removed via 10s headspace purge. NaO*t*Bu (192 mg, 2 mmol, 2 equiv) was added to the reaction mixture and the reaction was vigorously stirred (1200 rpm) at 50 °C for 24 h. After cooling to room temperature, the reaction was diluted with ethyl acetate and filtered through a pad of Celite©. The solution was washed with brine (20 mL), the aqueous phase was extracted with ethyl acetate (2x25 mL), and the combined organic phases were dried over anhydrous $MgSO_4$ and concentrated under reduced pressure. Silica gel column chromatography (elute: *n*-hexane / ethyl acetate) afforded the desired product as a white solid (238 mg, 0.76 mmol, 76%). **^{1}H NMR** (400 MHz, $CDCl_3$) δ 8.64 (s, 1H), 7.52 – 7.45 (m, 4H), 7.41 – 7.34 (m, 2H), 7.30 (t, *J* = 7.3 Hz, 1H), 6.87 – 6.81 (m, 2H), 5.79 (m, 1H), 5.24 (dd, *J* = 17.1, 1.7 Hz, 1H), 5.14 (d, *J* = 11.2 Hz, 1H), 3.77 (s, 3H), 3.42 (dd, *J* = 14.7, 6.6 Hz, 1H), 3.27 (s, 3H), 2.92 ppm (dd, *J* = 14.8, 7.3 Hz, 1H). **^{13}C NMR** (101 MHz, $CDCl_3$) δ 170.16, 156.46, 139.51, 132.22, 130.86, 128.66, 128.19, 126.54, 121.35, 118.86, 114.19, 84.24, 55.60, 51.13, 36.49 ppm.

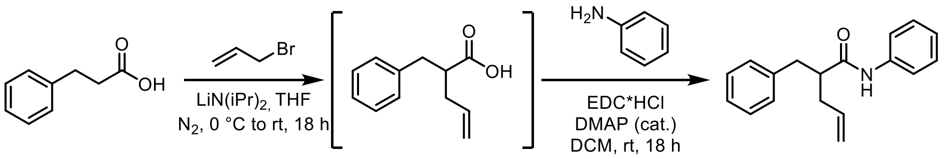

2-Benzyl-*N*-phenylpent-4-enamide (rac-59). Prepared according to Method D using 3-phenylpropanoic acid (300 mg, 2 mmol); the product was obtained as a white solid (225 mg, 0.85 mmol, 42%). **^{1}H NMR** (400 MHz, $CDCl_3$) δ 7.37 – 7.20 (m, 9H), 7.10 (t, *J* = 7.3 Hz, 1H), 7.03 (s, 1H), 5.99 – 5.66 (m, 1H), 5.22 – 5.00 (m, 2H), 3.04 (dd, *J* = 13.5, 8.8 Hz, 1H), 2.88 (dd, *J* = 13.5, 4.9 Hz, 1H), 2.64 – 2.49 (m, 2H), 2.37 ppm (m, 1H). **^{13}C NMR** (101 MHz, $CDCl_3$) δ 172.76, 139.64, 137.56, 135.54, 129.05, 128.93, 128.69, 126.59, 124.45, 120.38, 117.53, 50.86, 38.86, 36.90 ppm.

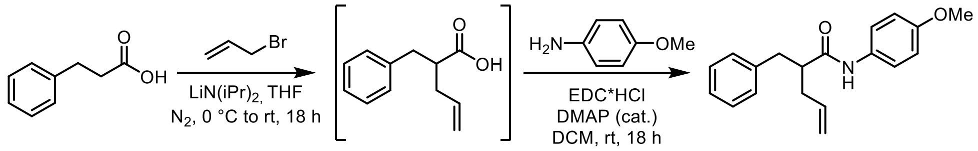

2-Benzyl-N-(4-methoxyphenyl)pent-4-enamide (rac-60). Prepared according to Method D using 3-phenylpropanoic acid (300 mg, 2 mmol); the product was obtained as a white solid (135 mg, 0.45 mmol, 45%). **^{1}H NMR** (400 MHz, $CDCl_3$) δ 7.38 – 7.26 (m, 7H), 7.05 (s, 1H), 6.86 (d, *J* = 8.7 Hz, 2H), 5.93 (m, 1H), 5.22 (d, *J* = 17.1 Hz, 1H), 5.16 (d, *J* = 10.3 Hz, 1H), 3.84 (s, 3H), 3.13 – 2.87 (m, 2H), 2.62 (m, 2H), 2.44 – 2.38 ppm (m, 1H). **^{13}C NMR** (101 MHz, $CDCl_3$) δ 172.65, 156.59, 139.75, 135.65, 130.63, 129.08, 128.66, 126.55, 122.40, 117.41, 114.09, 55.56, 50.66, 38.88, 36.91 ppm.

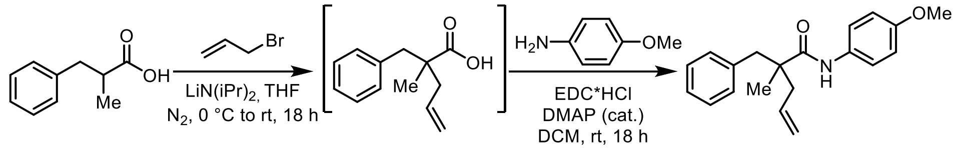

2-Benzyl-N-(4-methoxyphenyl)-2-methylpent-4-enamide (rac-61). Prepared according to Method D using 2-methyl-3-phenylpropanoic acid (164 mg, 1 mmol); the product was obtained as a white solid (75 mg, 0.24 mmol, 24%). **^{1}H NMR** (400 MHz, $CDCl_3$) δ 7.34 – 7.20 (m, 7H), 7.00 (s, 1H), 6.89 (d, *J* = 8.9 Hz, 2H), 5.90 (m, 1H), 5.21 (d, *J* = 9.9 Hz, 1H), 5.18 (d, *J* = 3.2 Hz, 1H), 3.84 (s, 3H), 3.25 (s, 1H), 2.76 (dd, *J* = 13.4, 6.0 Hz, 2H), 2.30 – 2.21 (m, 1H), 1.26 ppm (s, 3H). **^{13}C NMR** (101 MHz, $CDCl_3$) δ 174.07, 156.70, 137.70, 134.20, 130.69, 130.44, 128.24, 126.70, 122.59, 118.74, 114.16, 55.60, 47.59, 46.17, 44.44, 20.80 ppm.

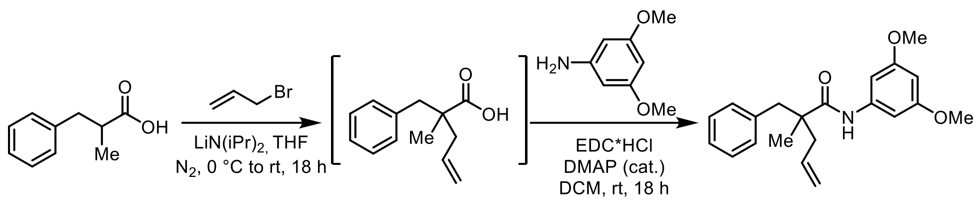

2-Benzyl-N-(3,5-dimethoxyphenyl)-2-methylpent-4-enamide (rac-62). Prepared according to Method D using 2-methyl-3-phenylpropanoic acid (164 mg, 1 mmol); the product was

obtained as a white solid (67 mg, 0.19 mmol, 19%). **^{1}H NMR** (400 MHz, Chloroform-*d*) δ 7.28 – 7.21 (m, 3H), 7.15 (dd, *J* = 7.9, 1.7 Hz, 2H), 6.98 (s, 1H), 6.64 (d, *J* = 2.3 Hz, 2H), 6.23 (d, *J* = 2.2 Hz, 1H), 5.91 – 5.77 (m, 1H), 5.20 – 5.11 (m, 2H), 3.77 (s, 6H), 3.15 (d, *J* = 13.3 Hz, 1H), 2.76 – 2.64 (m, 2H), 2.21 (dd, *J* = 13.8, 8.0 Hz, 1H), 1.21 ppm (s, 3H). **^{13}C NMR** (101 MHz, $CDCl_3$) δ 174.31, 161.08, 139.41, 137.50, 134.05, 130.43, 128.33, 126.78, 118.95, 98.54, 97.08, 55.52, 47.85, 46.12, 44.30, 20.96 ppm.

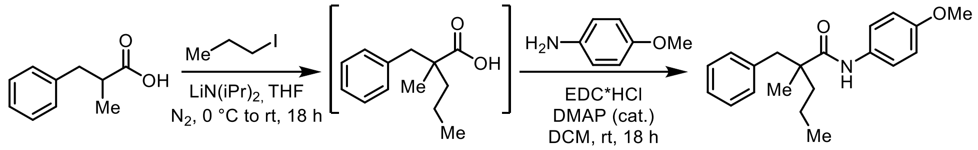

2-Benzyl-N-(4-methoxyphenyl)-2-methylpentanamide (rac-63). Prepared according to Method D using 2-methyl-3-phenylpropanoic acid (164 mg, 1 mmol); the product was obtained as a white solid (99 mg, 0.32 mmol, 32%). **^{1}H NMR** (400 MHz, $CDCl_3$) δ 7.35 – 7.26 (m, 5H), 7.23 – 7.18 (m, 2H), 6.96 (s, 1H), 6.91 – 6.86 (m, 2H), 3.84 (s, 3H), 3.22 (d, *J* = 13.2 Hz, 1H), 2.71 (d, *J* = 13.1 Hz, 1H), 2.00 – 1.90 (m, 1H), 1.52 – 1.36 (m, 3H), 1.26 (s, 3H), 1.00 ppm (t, *J* = 7.0 Hz, 3H). **^{13}C NMR** (101 MHz, $CDCl_3$) δ 174.61, 156.64, 137.90, 130.78, 130.44, 128.18, 126.60, 122.54, 114.16, 55.61, 47.86, 46.66, 42.75, 20.68, 18.10, 14.76 ppm.

Preparation of Ligands L10 and L11

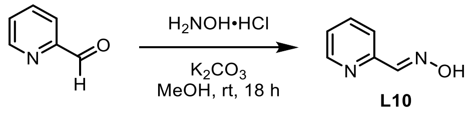

(*E*)-picolinaldehyde oxime (L10). Prepared according to a literature procedure.[1] To a solution of 2-pyridinecarboxaldehyde (0.95 mL, 10 mmol, 1 equiv) and K_2CO_3 (1.52 g, 11.0 mmol, 1.1 equiv) in MeOH (50 mL) was added hydroxylammonium chloride (0.76 g, 11.0 mmol, 1.1 equiv) and the reaction was stirred at room temperature for 18 h. After completion, the solvent was removed under reduced pressure, the residue the reaction mixture was diluted with water (50 mL) and extracted with ethyl acetate (3×50 mL). The combined organic layers were washed with brine (2×50 mL) and dried over $MgSO_4$. The solvent was removed under reduced pressure to yield the product without further purification; the product was obtained as a white solid (1.2 g, 9.83 mmol, 98%). **^{1}H NMR** (400 MHz, DMSO) δ 11.68 (s, 1H), 8.57 (dt, J = 4.9, 1.4 Hz, 1H), 8.08 (s, 1H), 7.85 – 7.75 (m, 2H), 7.37 ppm (m, 1H). **^{13}C NMR** (101 MHz, DMSO) δ 152.11, 149.43, 148.97, 136.78, 123.94, 119.78 ppm.

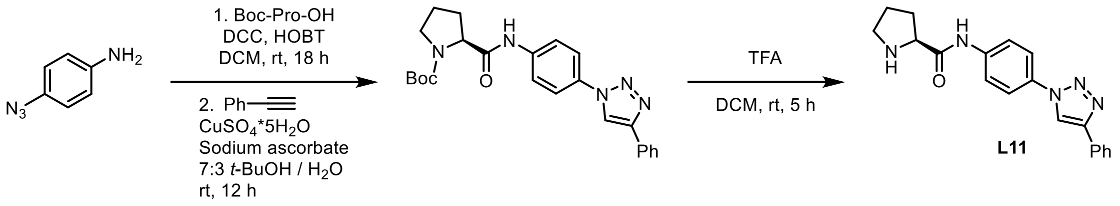

***tert*-Butyl (*S*)-2-((4-(4-phenyl-1H-1,2,3-triazol-1-yl)phenyl)carbamoyl)pyrrolidine-1-carboxylate.** 1) *N*-Boc proline (1 g, 4.65 mmol, 1 equiv), DCC (1.06 g, 5.1 mmol, 1.1 equiv) and HOBT (691 mg, 5.1 mmol, 1.1 equiv) were suspended in dry DCM (25 mL), and the mixture was allowed to stir for 45 min at 0 °C. Then, 4-azidoaniline (624 mg, 4.65 mmol, 1.0 equiv) in dry DCM (25 mL) was added dropwise to the reaction mixture and stirred for 12 h at room temperature. After completion, the reaction mixture was filtered through a pad of Celite©, and rinsed with ethyl acetate (100 mL). The organic layer was washed with 1 M HCl (30 mL), sat. NaHCO3 solution (30 mL) and brine (30 mL). The crude product was used in the next step without further purification. 2) Under N_2 atmosphere, phenylacetylene (510 µL, 4.65 mmol, 1 equiv), sodium ascorbate (92 mg, 0.47 mmol, 0.1 equiv), $CuSO_4*5H_2O$ (58 mg, 0.23 mmol, 0.05 equiv) were dissolved in 7:3 *t*-BuOH-H_2O (5 mL). Then, the azido prolinamide was added and the reaction was stirred at room temperature for 12 h. After completion, the reaction mixture was concentrated and silica gel column chromatography

yielded the desired product as a white solid (1.4 g, 3.23 mmol, 69%). **^{1}H NMR** (400 MHz, DMSO, major rotamer) δ 10.30 (s, 1H), 9.23 (d, *J* = 4.2 Hz, 1H), 7.97 – 7.83 (m, 6H), 7.49 (t, *J* = 7.7 Hz, 2H), 7.41 – 7.34 (m, 1H), 4.26 (m, 1H), 3.49 – 3.29 (m, 2H), 2.23 (m, 1H), 1.96 – 1.75 (m, 3H), 1.28 ppm (s, 9H). **^{13}C NMR** (101 MHz, DMSO, major rotamer) δ 171.90, 153.12, 147.20, 139.41, 131.85, 130.34, 128.99, 128.19, 125.32, 120.76, 120.01, 119.48, 78.57, 60.47, 46.59, 30.99, 27.96, 23.43 ppm. Spectral data in agreement with literature data.[2]

(*S*)-*N*-(4-(4-phenyl-1H-1,2,3-triazol-1-yl)phenyl)pyrrolidine-2-carboxamide (L11). To a solution of Boc-protected ligand (1.3 g, 3 mmol, 1 equiv) in dry DCM (30 mL), was added TFA (0.69 mL, 9 mmol, 3 equiv) at 0 °C and the reaction mixture was stirred for 18 h at room temperature. Next, the reaction mixture was slowly quenched with sat. $NaHCO_3$ solution (50 mL) at 0 °C. The reaction was extracted with DCM (3x30 mL), the combined organic layers were dried over $MgSO_4$ and the solvent was removed under reduced pressure. Silica gel column chromatography yielded the desired product as a yellow powder (921 mg, 2,76 mmol, 92%). **^{1}H NMR** (400 MHz, DMSO) δ 10.23 (s, 1H), 9.24 (s, 1H), 7.98 – 7.84 (m, 6H), 7.50 (t, *J* = 7.7 Hz, 2H), 7.43 – 7.34 (m, 1H), 3.74 (dd, *J* = 8.8, 5.6 Hz, 1H), 3.39 (s, 1H), 2.91 (t, *J* = 6.6 Hz, 2H), 2.14 – 2.00 (m, 1H), 1.80 (m, 1H), 1.74 – 1.60 ppm (m, 2H). **^{13}C NMR** (101 MHz, DMSO) δ 173.79, 147.19, 138.87, 131.89, 130.33, 128.99, 128.19, 125.31, 120.52, 120.08, 119.43, 60.85, 46.77, 30.46, 25.87 ppm. Spectral data in agreement with literature data.[2]

Determination of Configuration of Chiral Amide 55

Synthesis and characterisation of the racemic standard rac-**55**:

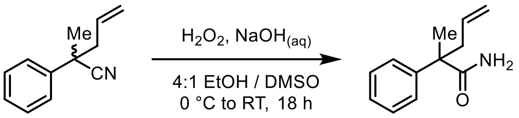

(±) **55**

Conduced according to a literature procedure.[6] To a stirred solution of the 2-methyl-2-phenylpent-4-enenitrile (85 mg, 0.5 mmol, 1 equiv) in 4:1 EtOH / DMSO (3 mL) was dropwise added H_2O_2 (30%, 0.51 mL, 5 mmol, 10 equiv) at 0 C and the mixture was stirred for 30 minutes. Then, an aqueous solution of NaOH (1 M, 1 mL, 2 equiv) was added and the mixture was stirred for overnight at room temperature. The reaction was quenched by addition of aqueous 10% $Na_2S_2O_3$ solution (20 mL), the aqueous phase was extracted with ethyl acetate (3x25 mL), and the combined organic layers were combined over anhydrous $MgSO_4$. The solvent was removed under reduced pressure and silica gel column chromatography afforded the desired product as a white solid (80 mg, 0.42 mmol, 85%). **^{1}H NMR** (400 MHz, $CDCl_3$) δ 7.34 – 7.22 (m, 4H), 7.19 (m, 1H), 6.22 (s, 1H), 5.48 (m, 1H), 5.25 (s, 1H), 5.03 – 4.90 (m, 2H), 2.73 – 2.60 (m, 2H), 1.46 ppm (s, 3H). **^{13}C NMR** (101 MHz, $CDCl_3$) δ 179.54, 143.42, 134.12, 128.67, 127.11, 126.79, 118.39, 49.98, 43.54, 23.54 ppm.

Chiral HPLC trace for rac-55:

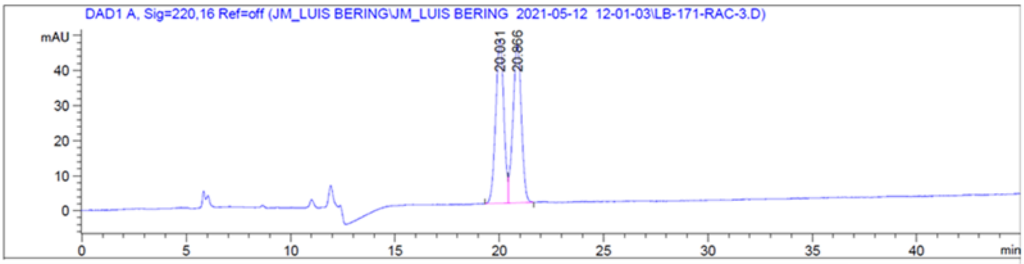

Signal 1: DAD1 A, Sig=220,16 Ref=off

Peak #	RetTime [min]	Type	Width [min]	Area [mAU*s]	Height [mAU]	Area %
1	20.031	BV	0.4094	1267.60242	46.88260	50.0114
2	20.866	VB	0.4323	1267.02612	45.02044	49.9886

^{1}H NMR spectrum of **rac-55** from standard synthesis.

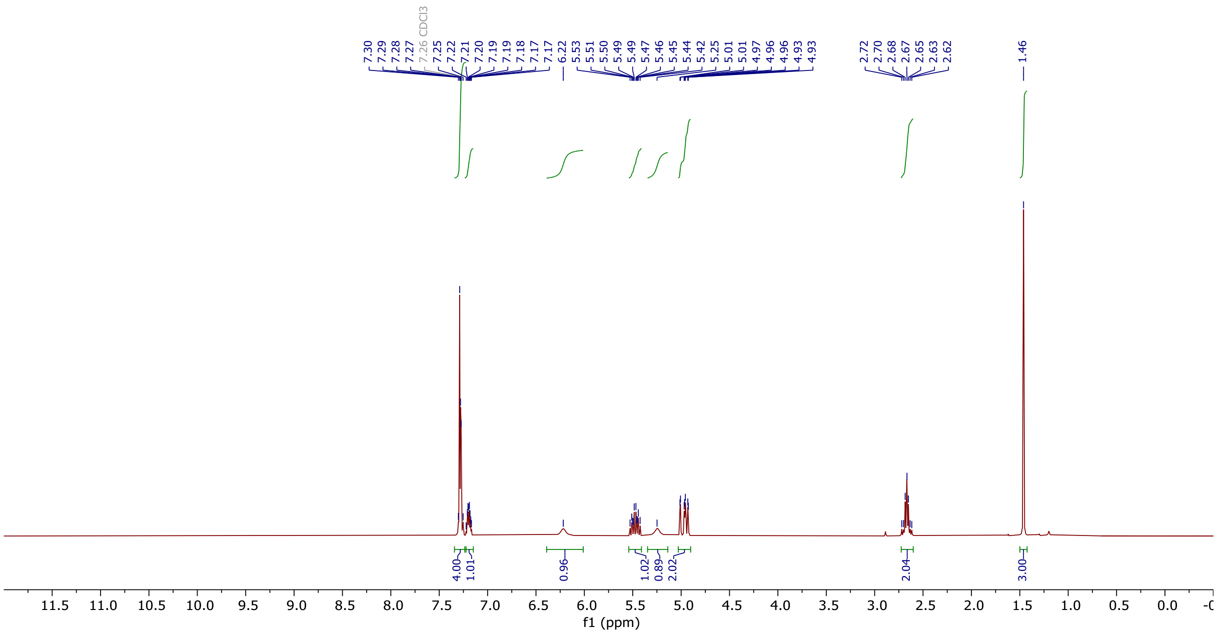

^{13}C NMR spectrum of **rac-55** from standard synthesis.

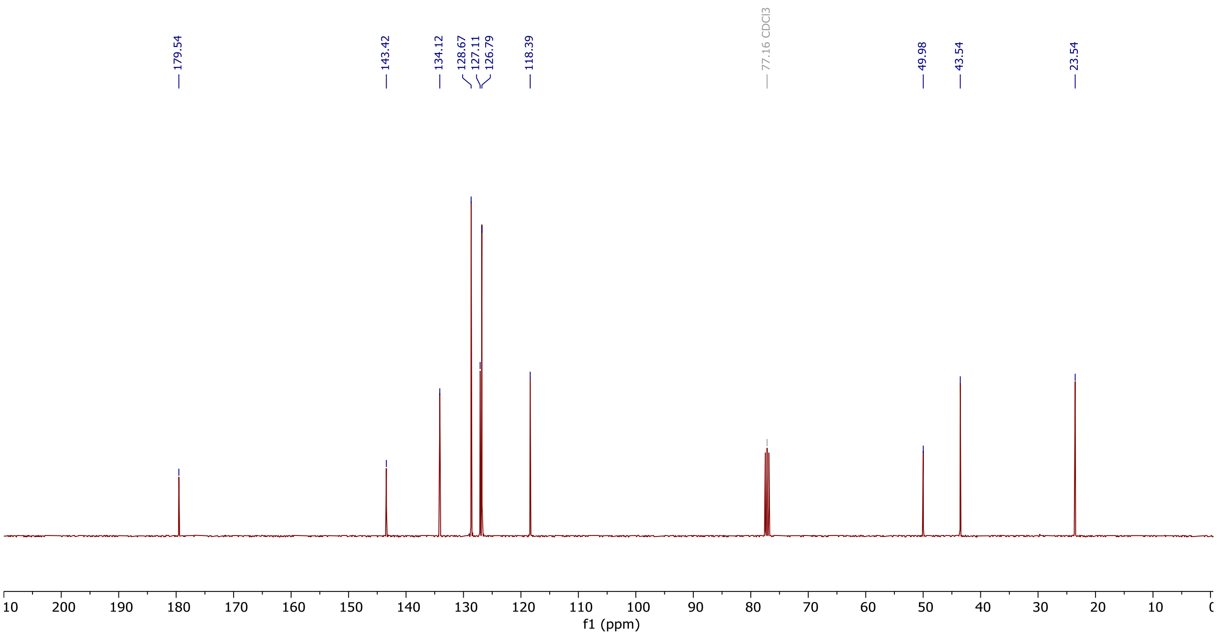

Synthesis and characterisation of chiral amide **55**:

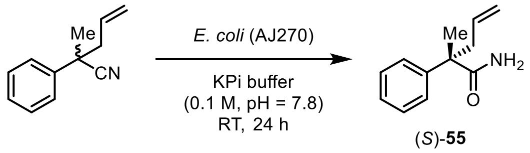

(*S*)-**55**

To a solution of 2-methyl-2-phenylpent-4-enenitrile (171 mg, 1 mmol, 1 equiv) in ~2 wt% TPGS-750 M / KPi buffer (0.1 M, pH = 7.8) were added *E. coli* (NHase) whole cells from ca. 40 mL cell culture in 2 wt% TPGS-750 M / KPi buffer (0.1 M, pH = 7.8) to a total volume of 1 mL and the reaction was stirred (400 rpm) for 24 h at room temperature. After that time, the reaction was diluted with ethyl acetate and filtered through a pad of Celite©. The solution was washed with brine (20 mL), the aqueous phase was extracted with ethyl acetate (2x25 ml), and the combined organic phases were dried over anhydrous $MgSO_4$ and concentrated under reduced pressure. Silica gel column chromatography afforded the desired product as a white solid (41 mg, 0.22 mmol, 22%). e.r. = 73:27. $[\alpha]_D^{25}$ = +13.4 (*c* = 0.1 M, $CHCl_3$).[7] **^{1}H NMR** (400 MHz, $CDCl_3$) δ 7.33 – 7.22 (m, 4H), 7.22 – 7.15 (m, 1H), 5.82 (s, 1H), 5.47 (ddt, *J* = 17.2, 10.1, 7.2 Hz, 1H), 5.18 (s, 1H), 5.03 – 4.90 (m, 2H), 2.73 – 2.61 (m, 2H), 1.46 ppm (s, 3H). **^{13}C NMR** (101 MHz, $CDCl_3$) δ 179.40, 143.42, 134.13, 128.77, 127.22, 126.87, 118.51, 50.10, 43.60, 23.64 ppm. Chiral HPLC conditions: *i*-Cellulose 4.6 x 250mm column, *n*-hexane / iPrOH = 80:20, flow rate = 0.5 mL min^{-1}, minor enantiomer: t_R = 20.93 min; major enantiomer: t_R = 20.08 min. The absolute configuration was assigned by comparison of the optical rotation with that of the authentic (*R*)-2-methyl-2-phenylpent-4-enamide $[\alpha]_D^{25}$ = −57.5 (*c* = 0.6, $CHCl_3$).[7]

Chiral HPLC trace for 55:

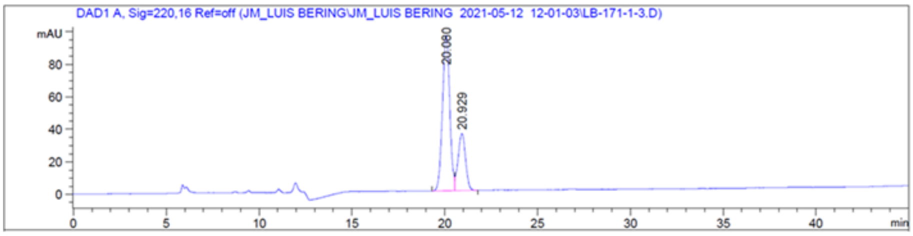

===

Signal 1: DAD1 A, Sig=220,16 Ref=off

Peak #	RetTime [min]	Type	Width [min]	Area [mAU*s]	Height [mAU]	Area %
1	20.080	BV	0.4195	2594.84619	95.35711	72.5626
2	20.929	VB	0.4145	981.16589	35.06025	27.4374

^{1}H NMR spectrum of **55** from the integrated synthesis.

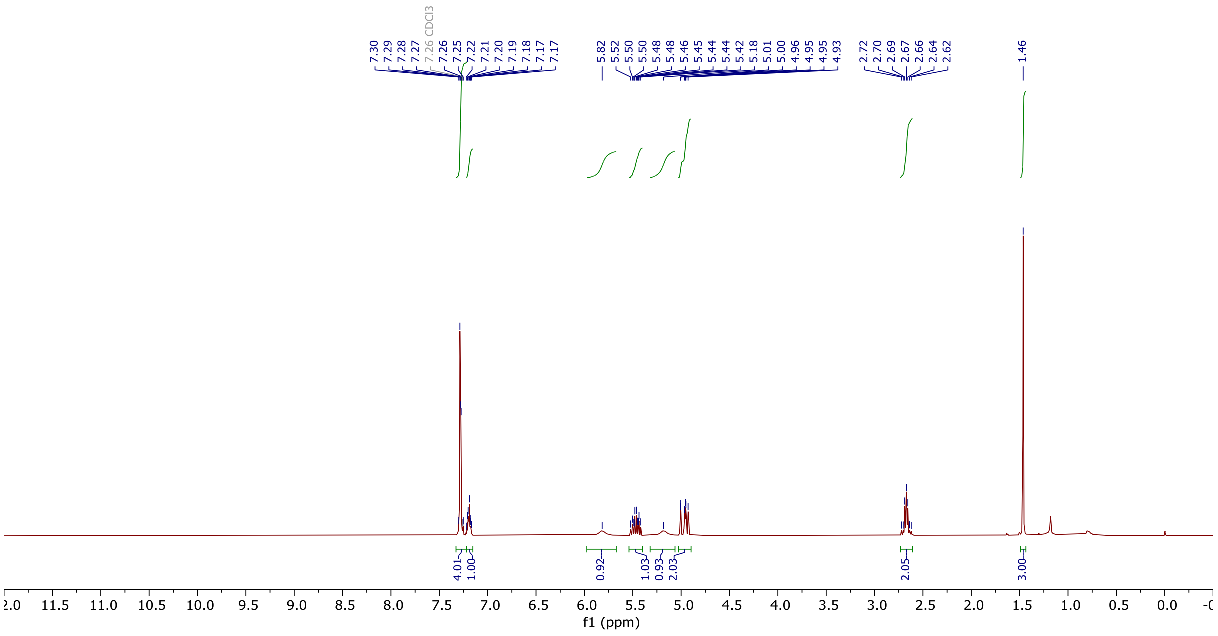

^{13}C NMR spectrum of **55** from the integrated synthesis.

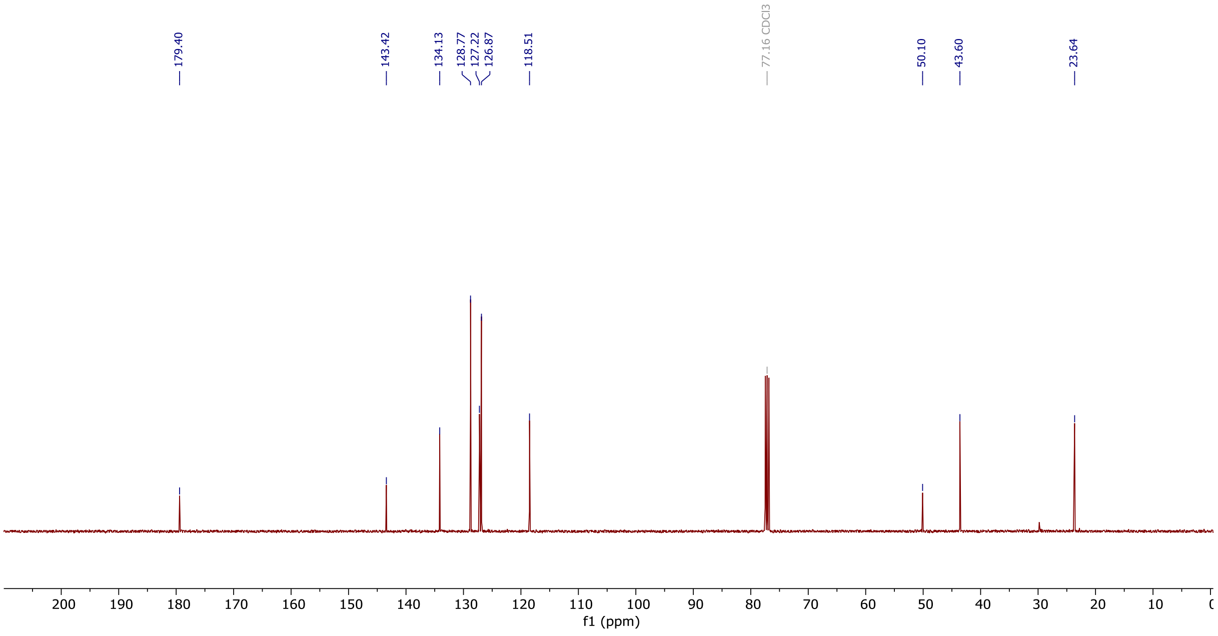

Procedure for NHase Recycling Experiment

To a solution of benzonitrile (**1**) (21 µL, 0.2 mmol, 1 equiv) in KPi buffer (0.1 M, pH = 7.8) / 10% v/v *i*PrOH were added *E.coli* (NHase) whole cells from ca. 10 mL cell culture (final OD_{600} ~ 0.1) in KPi buffer (0.1 M, pH = 7.8) to a total volume of 4 mL and the reaction was stirred (400 rpm) for 24 h at room temperature. After that time, the solution was centrifuged (2415 g, 10 min, 4 °C) and the supernatant, containing the crude primary amide **2**, was transferred into a 4 mL screwcap vial. Subsequently, iodobenzene (66 µL, 0.6 mmol, 3 equiv), **L1** (6 µL, 0.04 mmol, 0.2 equiv), D-glu (7.5 mg, 0.04 mmol, 0.2 equiv), $CuBr_2$ (4.5 mg, 0.02 mmol, 0.1 equiv), and NaO*t*Bu (39 mg, 0.4 mmol, 2 equiv) were successively added and the reaction was vigorously stirred (1200 rpm) at 50 °C under N_2 atmosphere (headspace purge) for 24 h. After cooling to room temperature, the reaction was diluted with ethyl acetate and filtered through a pad of Celite©. The solution was washed with brine (10 mL), dried over anhydrous $MgSO_4$ and concentrated under reduced pressure. Silica gel column chromatography (elute: *n*-hexane / ethyl acetate) afforded the desired amide product **4**. Meanwhile, the recycled cells were re-suspended in KPi buffer (0.1 M, pH = 7.8) and added to a fresh solution of benzonitrile (**1**) (21 µL, 0.2 mmol, 1 equiv) in KPi buffer (0.1 M, pH = 7.8) / 10% v/v *i*PrOH to a final volume of 4 mL in order to perform the subsequent hydration/arylation cascade. This process was repeated for a total of 10 NHase hydration/arylation cycles.

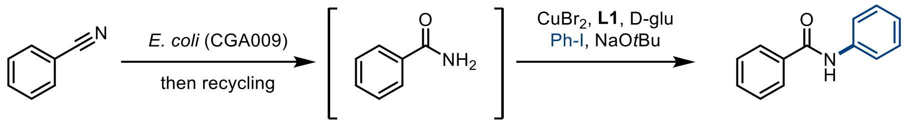

1 **2** [in situ] **4**

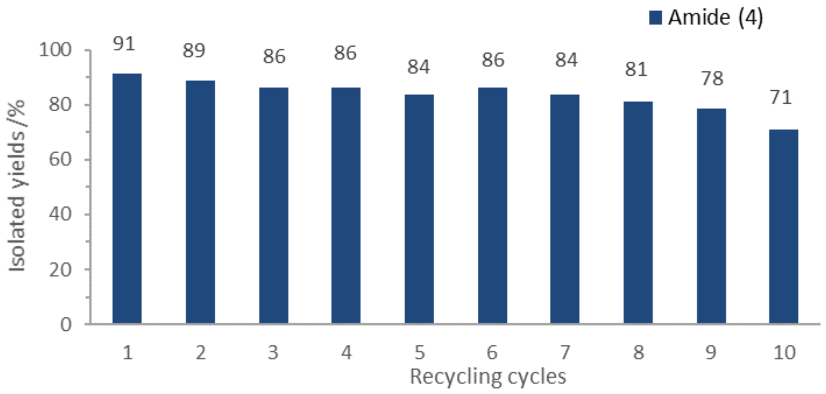

Supplementary Figure 2. Results of the NHase recycling experiment for the synthesis of amide **4**.

NMR spectra and HPLC traces

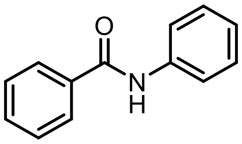

^{1}H NMR spectrum of **4**.

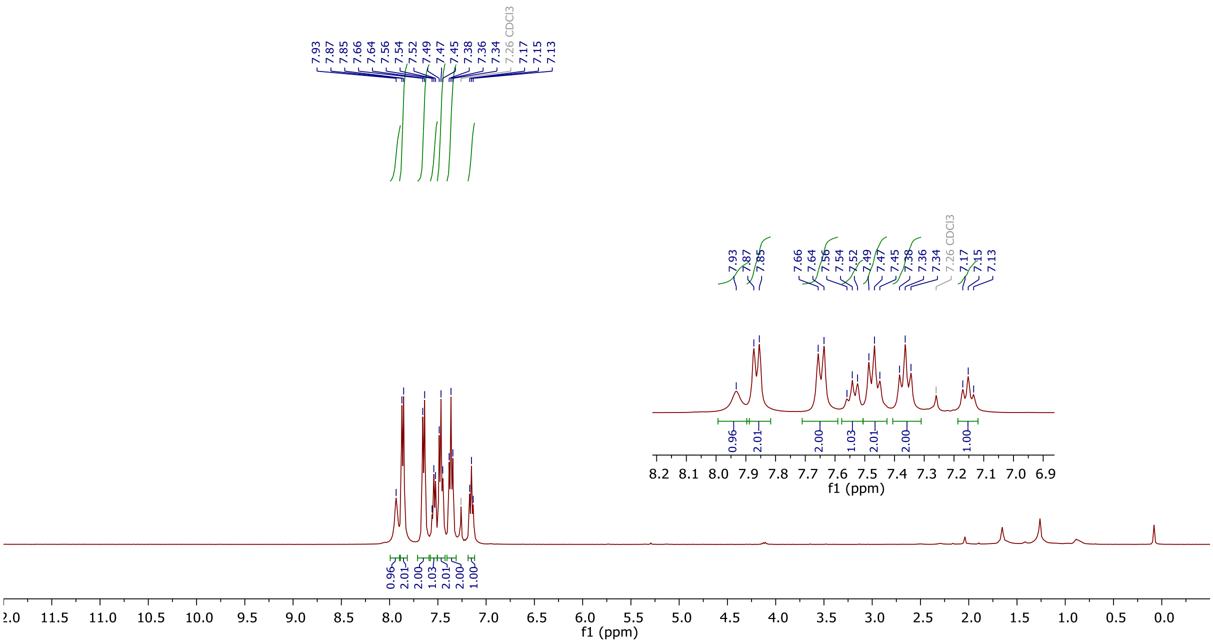

^{13}C NMR spectrum of **4**.

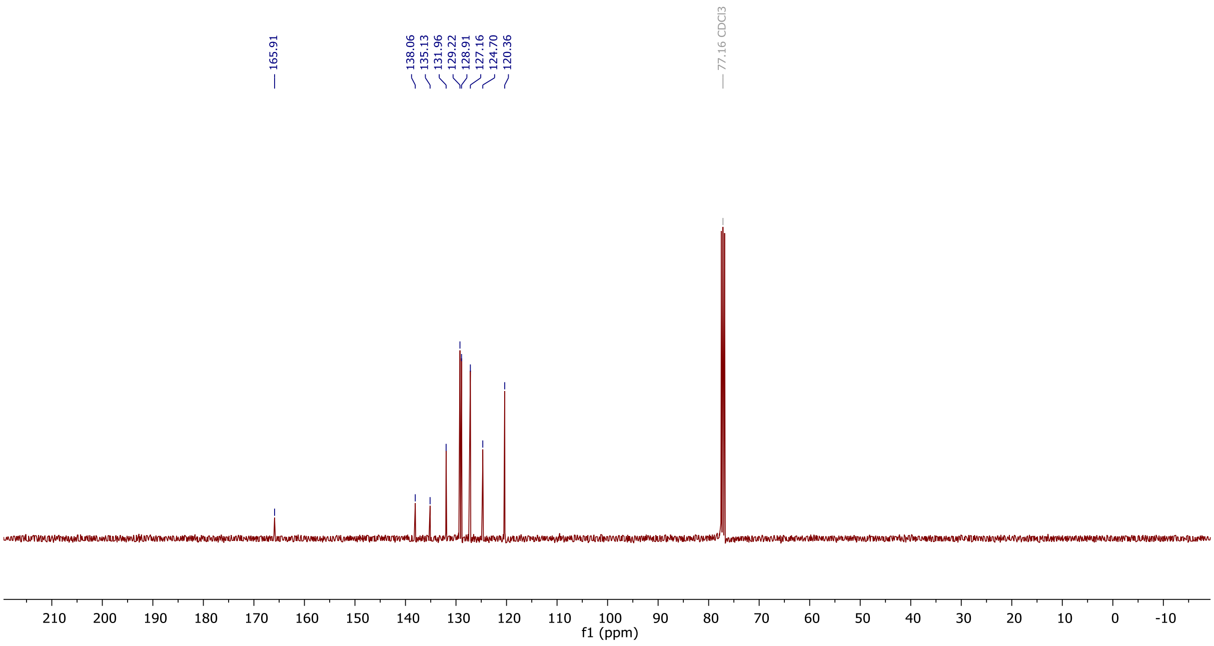

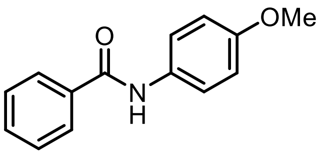

^{1}H NMR spectrum of **5**.

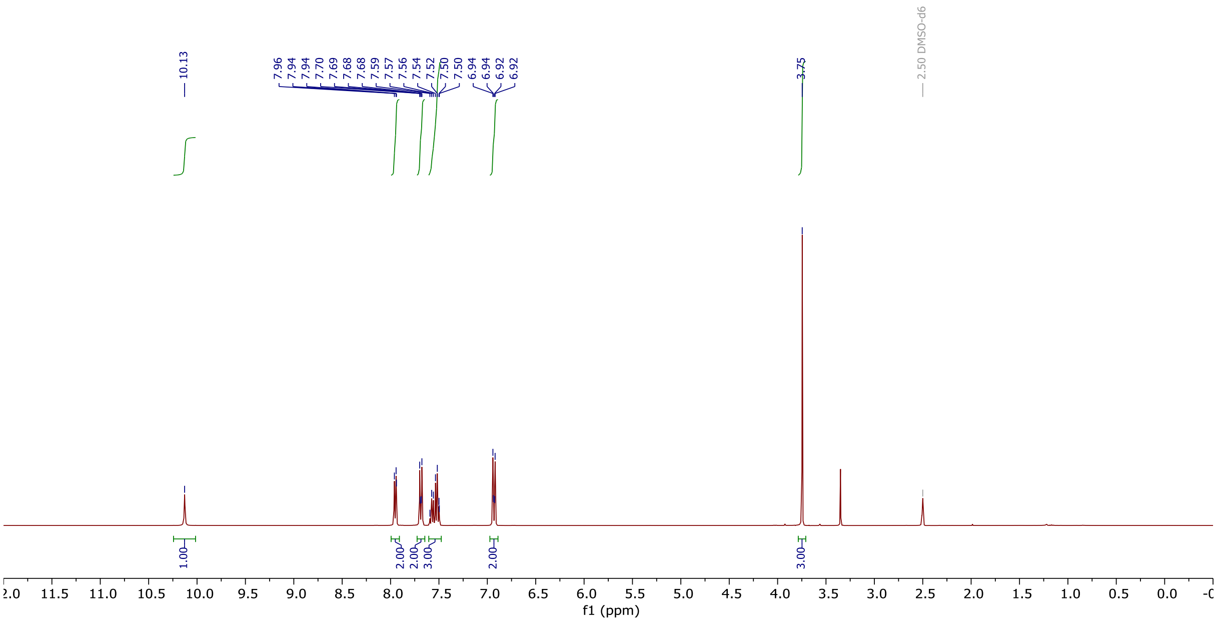

^{13}C NMR spectrum of **5**.

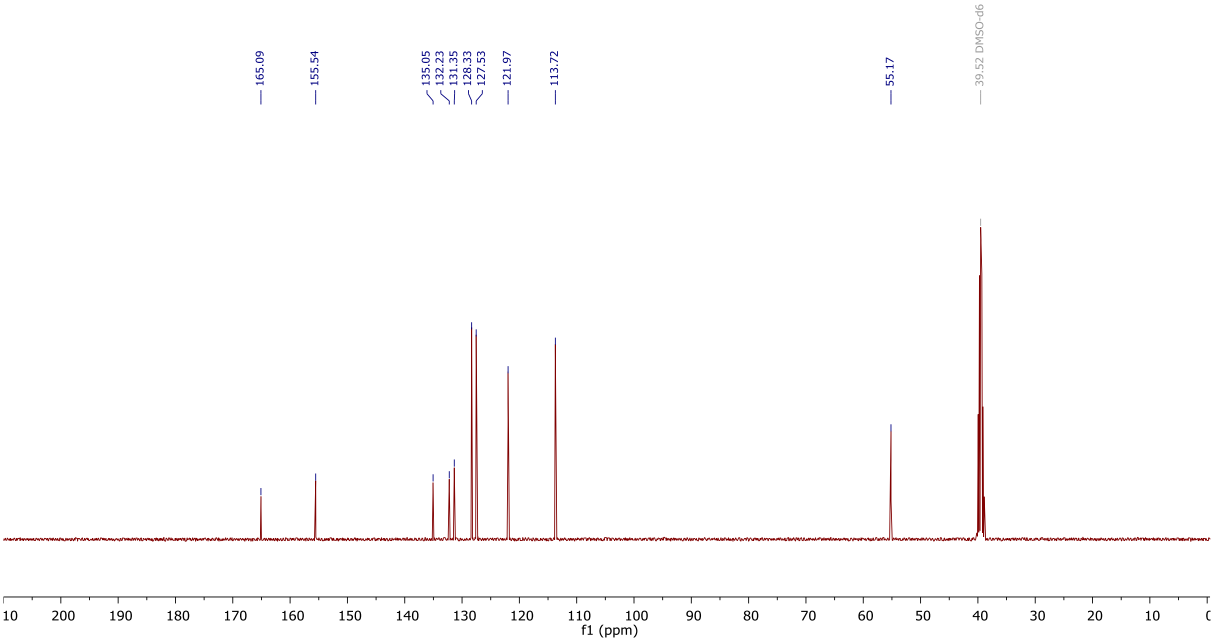

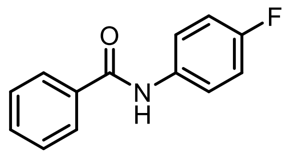

^{1}H NMR spectrum of **6**.

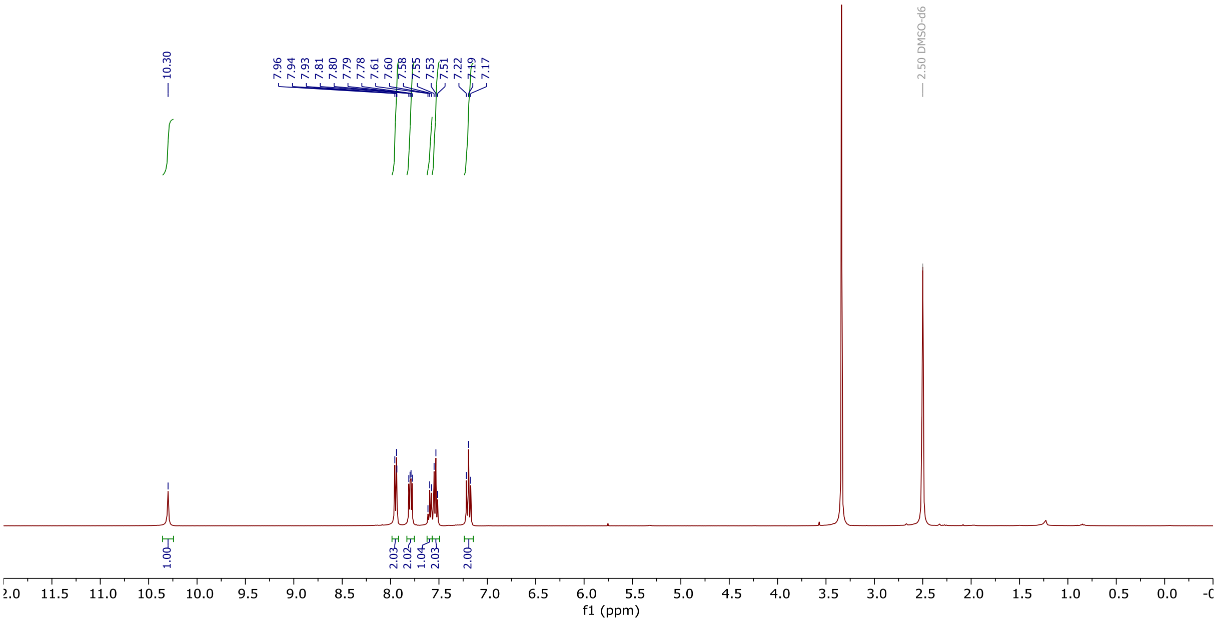

^{13}C NMR spectrum of **6**.

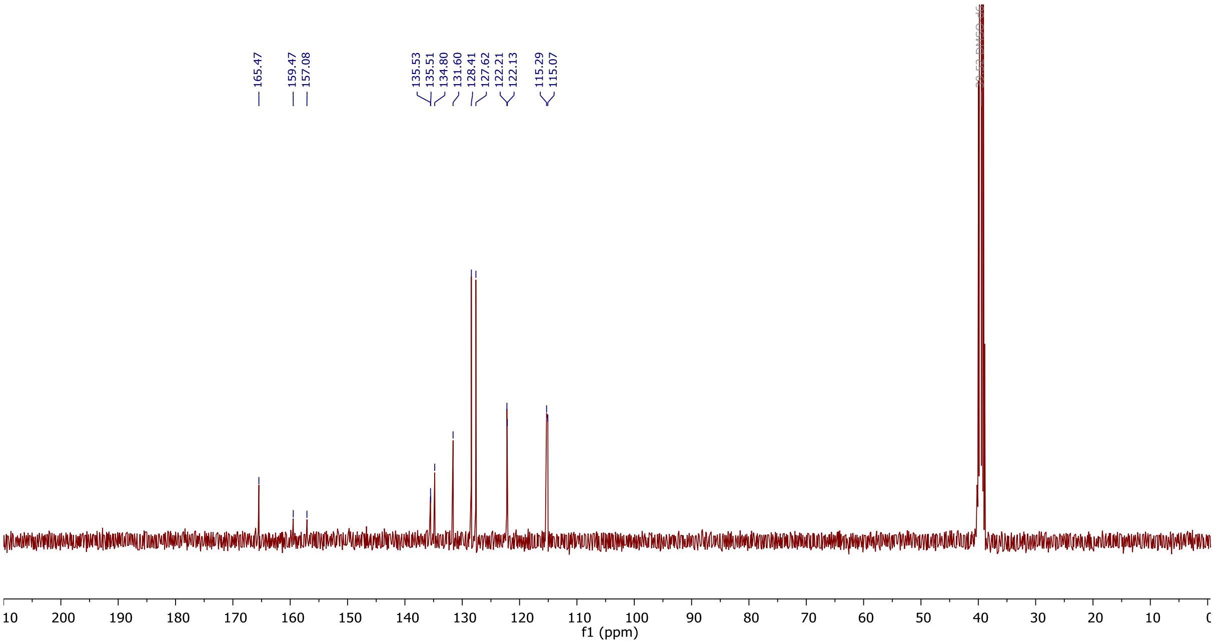

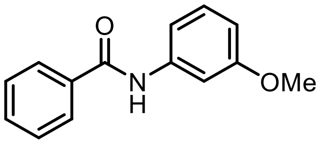

^{1}H NMR spectrum of **7**.

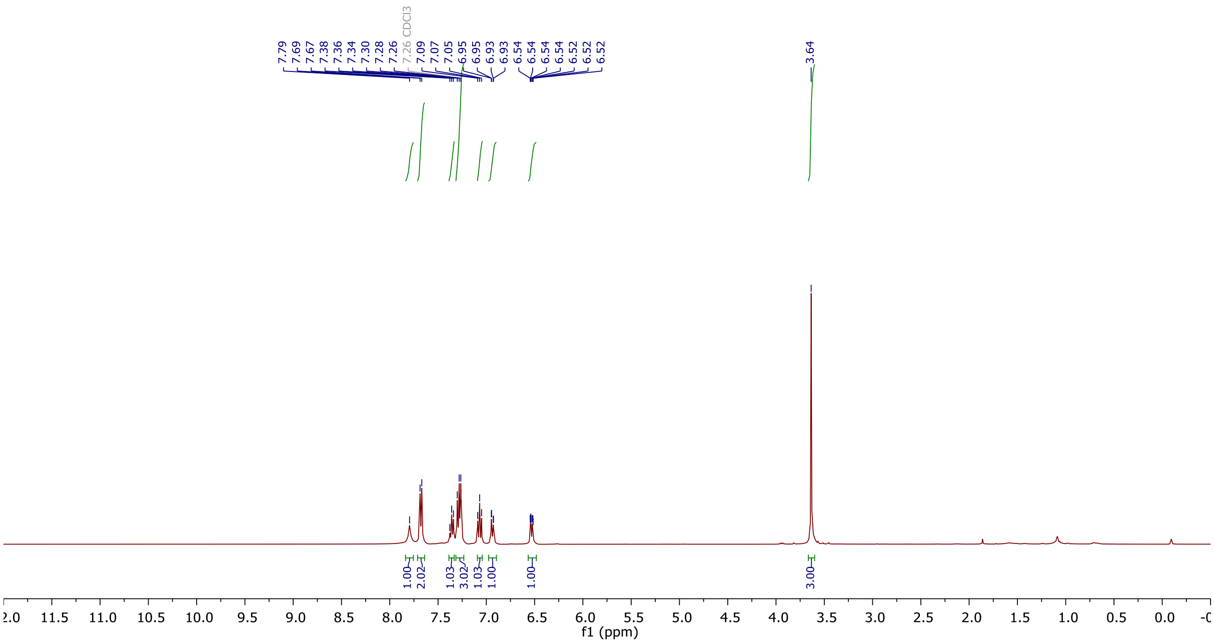

^{13}C NMR spectrum of **7**.

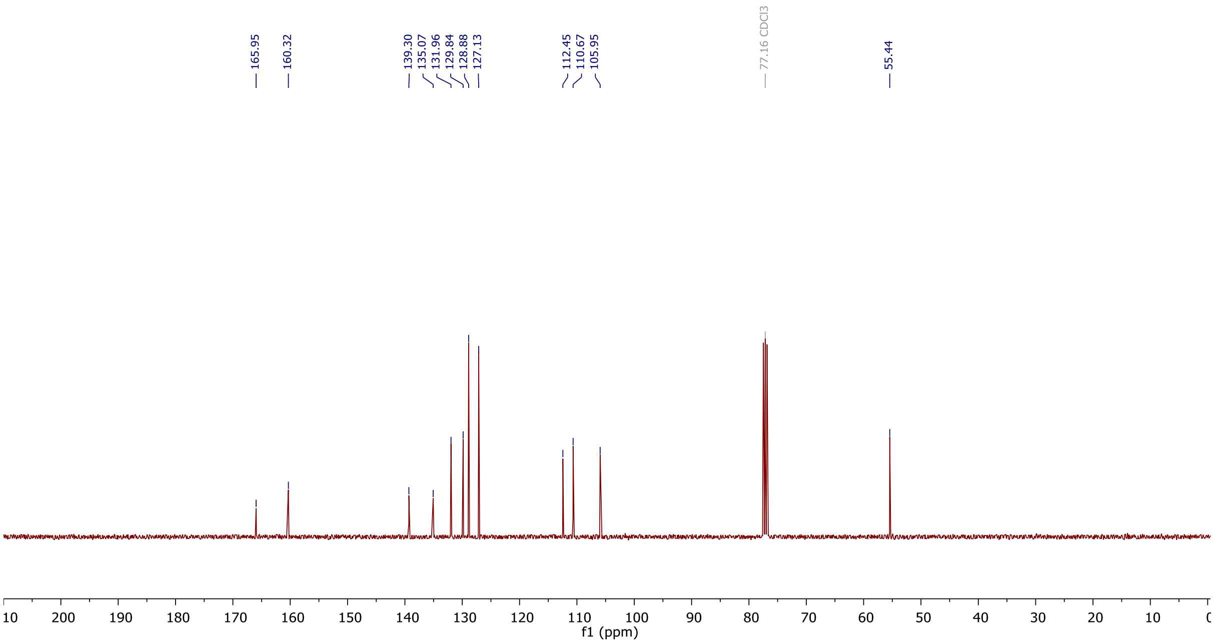

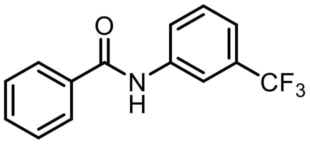

^{1}H NMR spectrum of **8**.

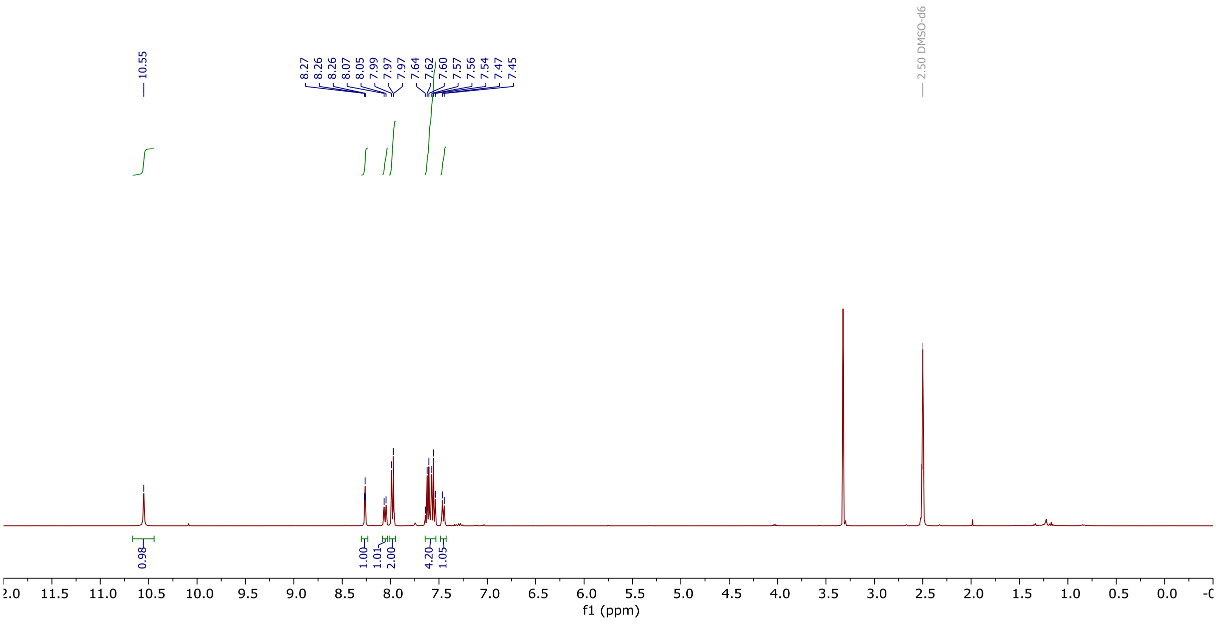

^{13}C NMR spectrum of **8**.

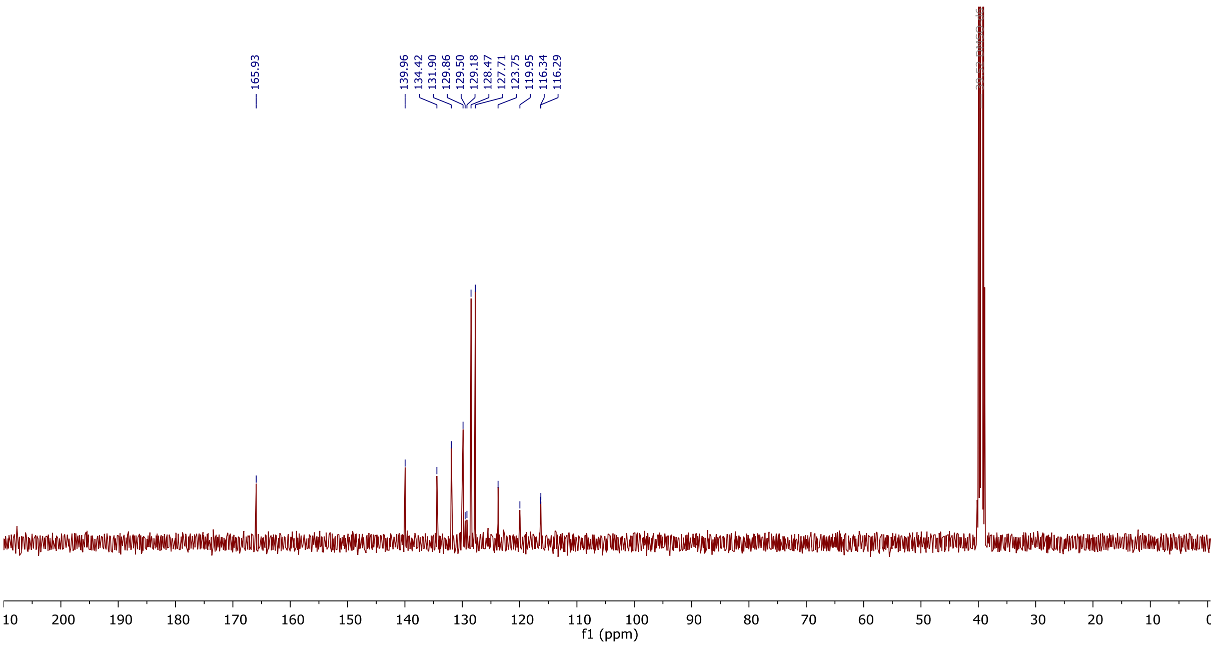

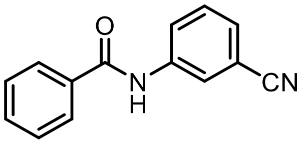

^{1}H NMR spectrum of **9**.

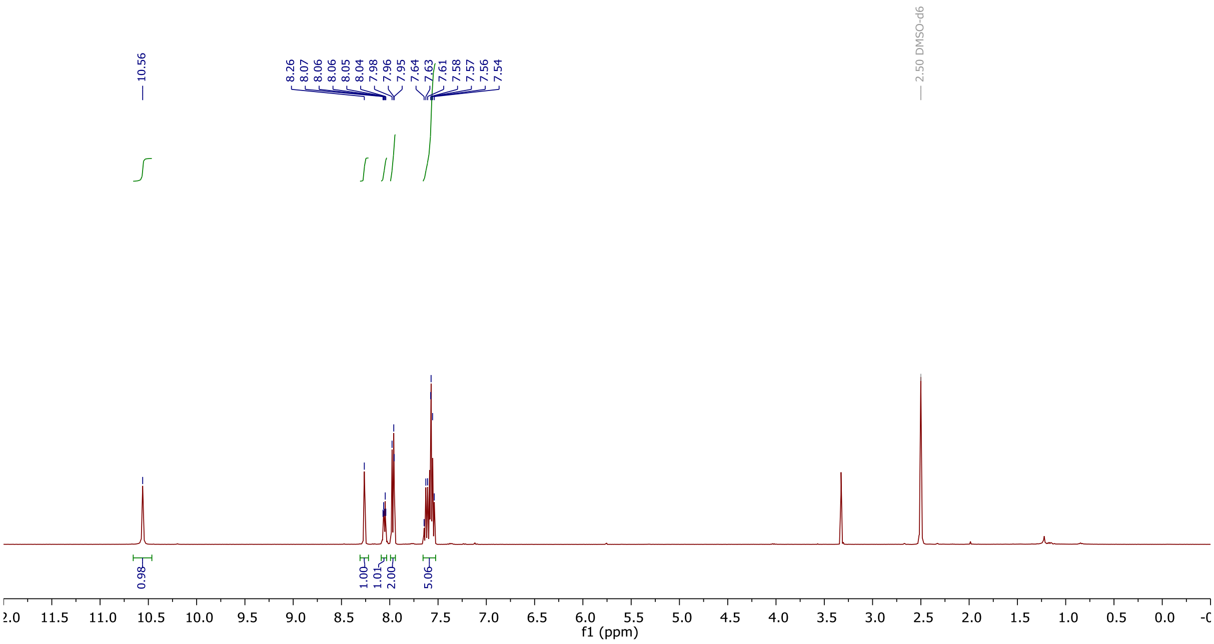

^{13}C NMR spectrum of **9**.

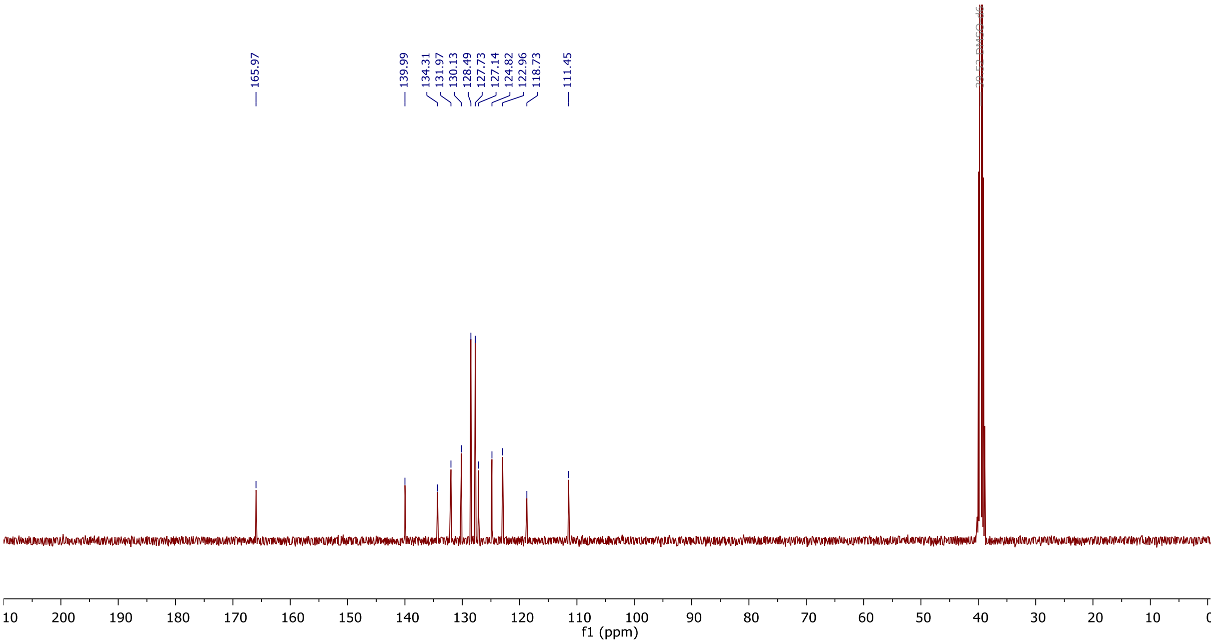

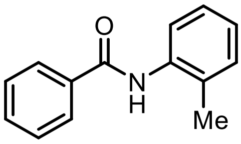

^{1}H NMR spectrum of **10**.

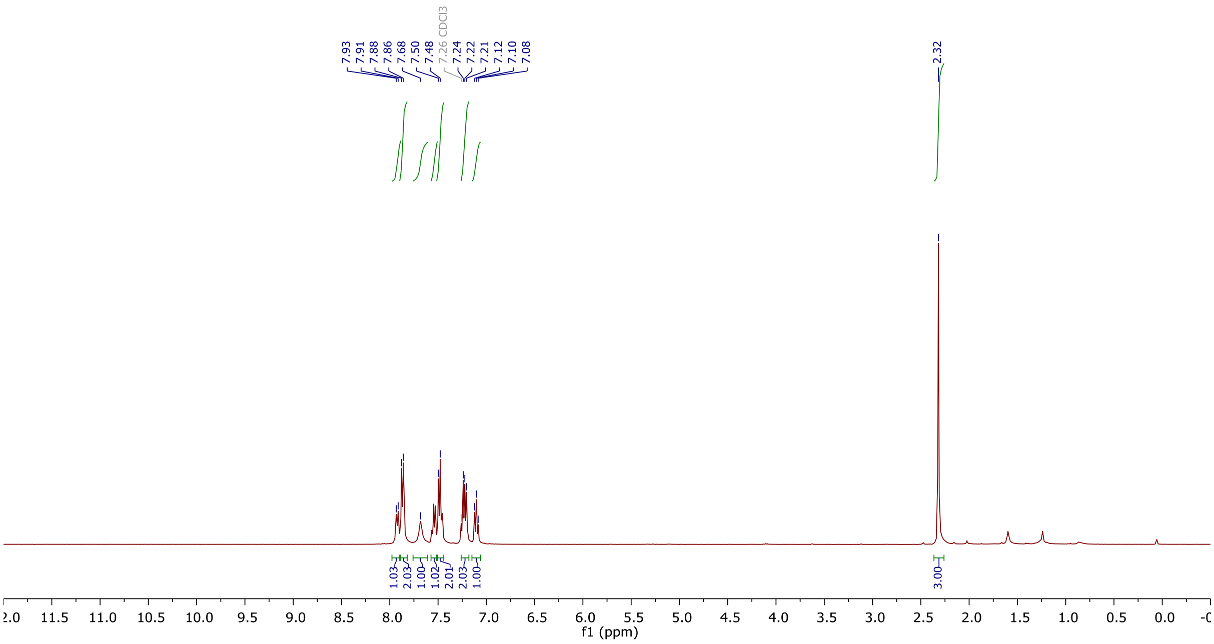

^{13}C NMR spectrum of **10**.

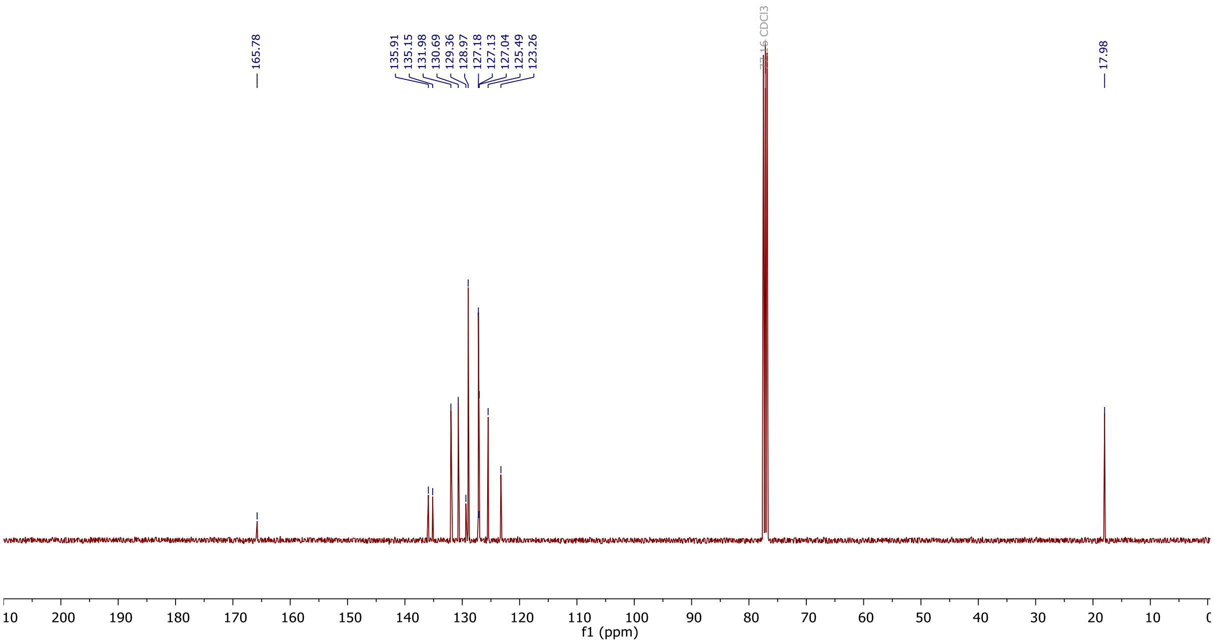

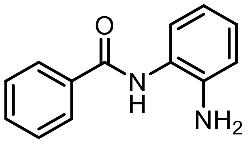

^{1}H NMR spectrum of **11**.

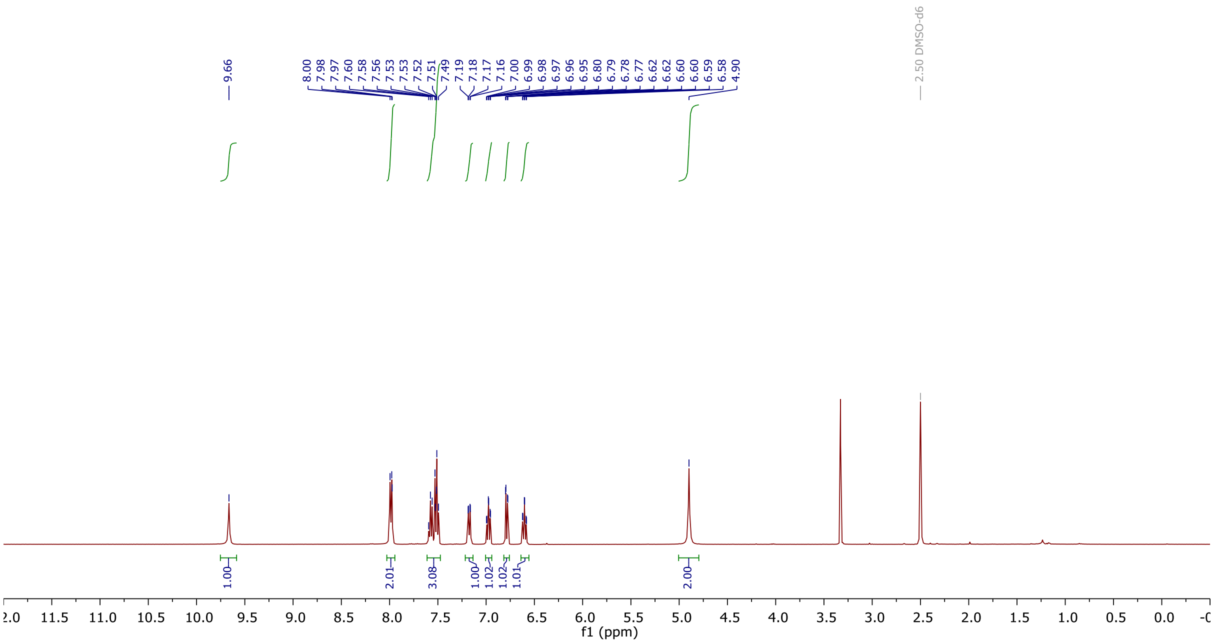

^{13}C NMR spectrum of **11**.

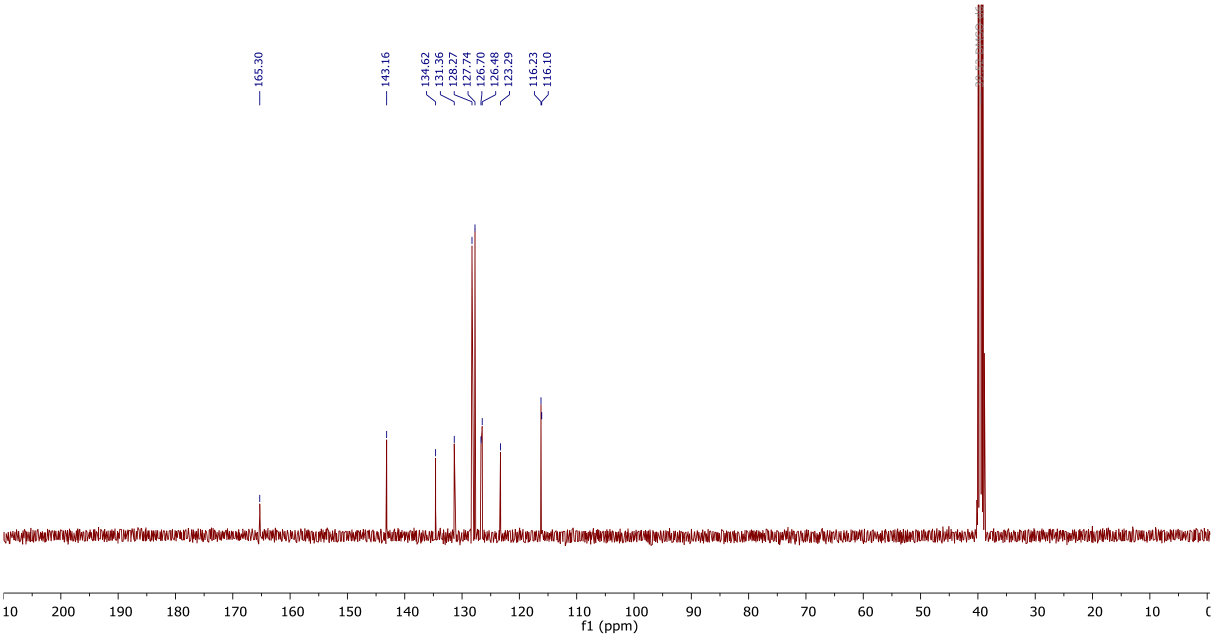

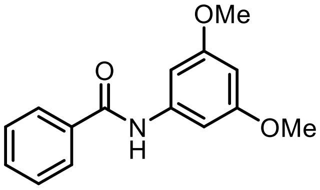

^{1}H NMR spectrum of **12**.

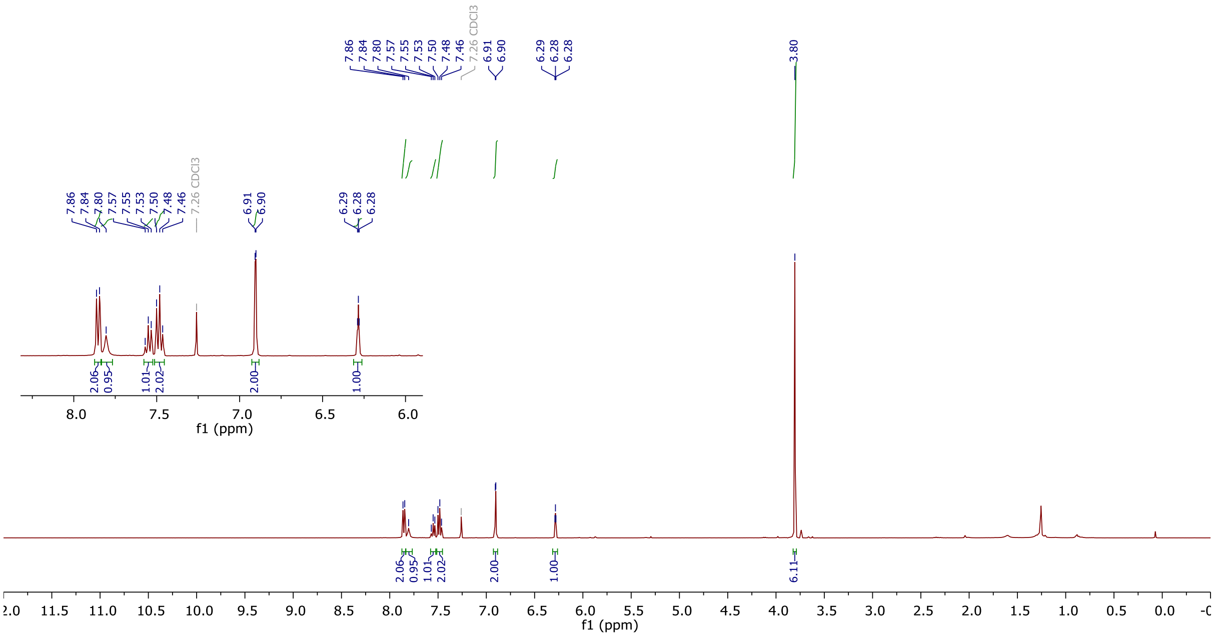

^{13}C NMR spectrum of **12**.

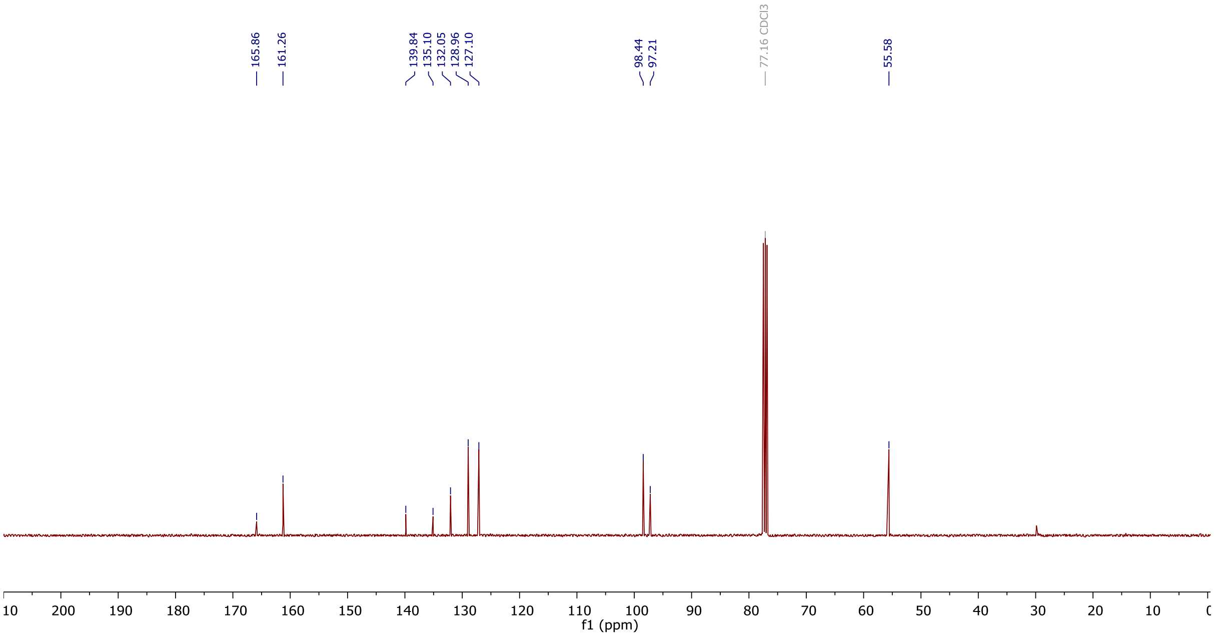

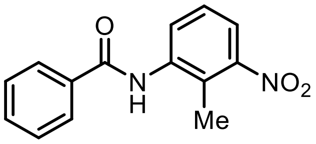

^{1}H NMR spectrum of **13**.

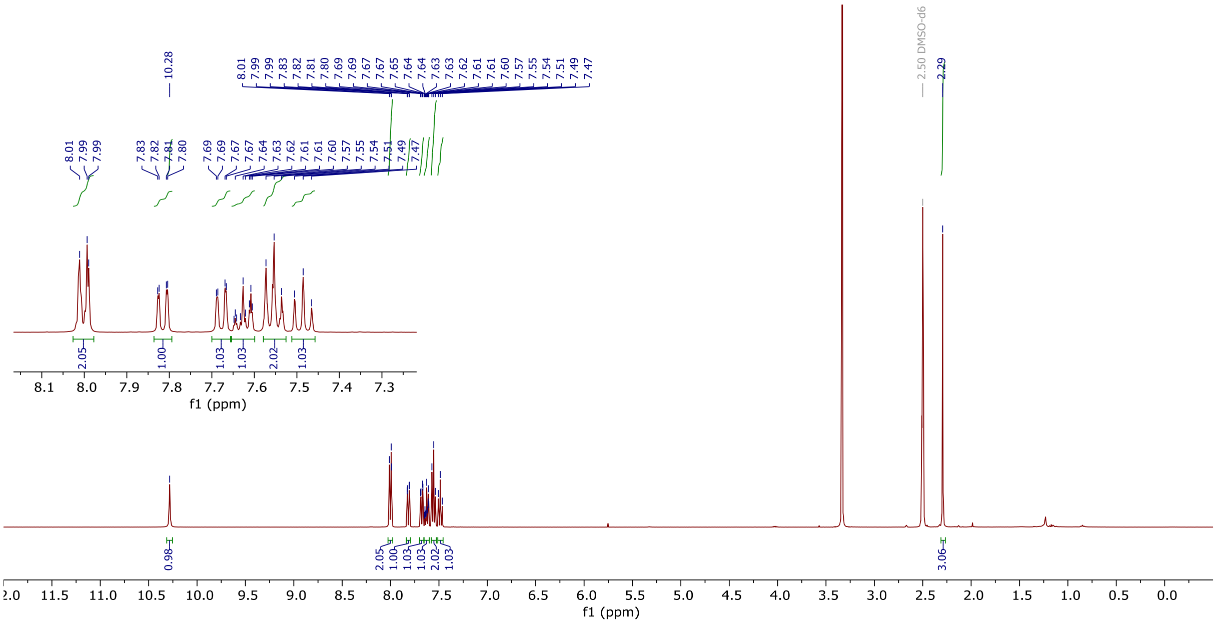

^{13}C NMR spectrum of **13**.

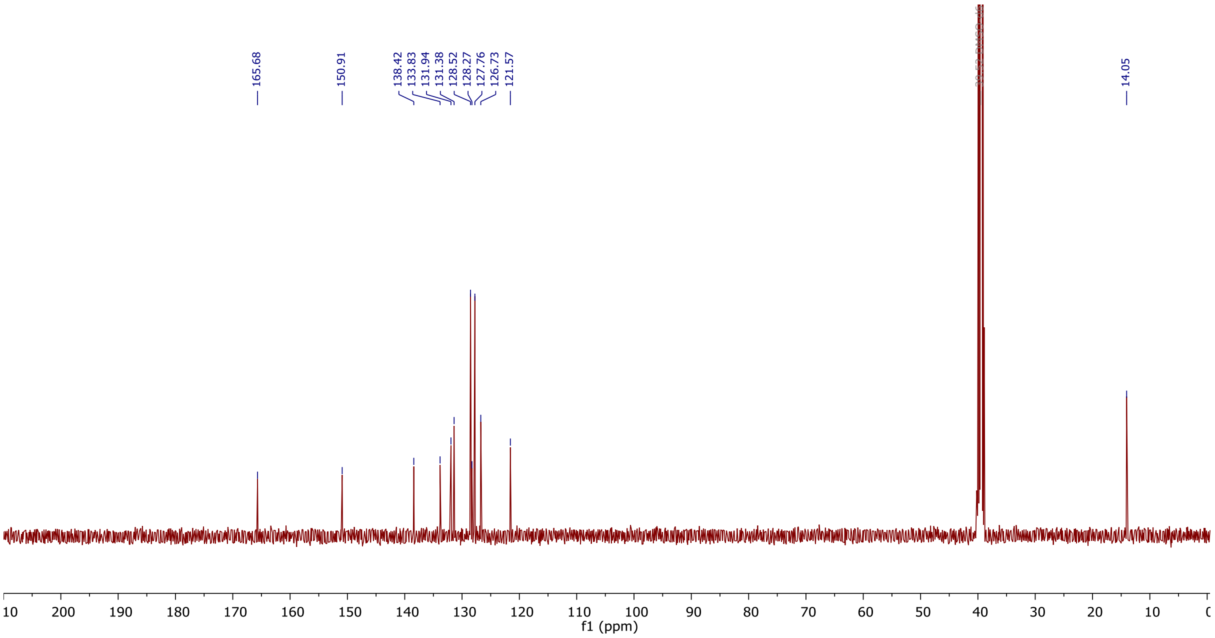

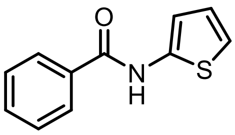

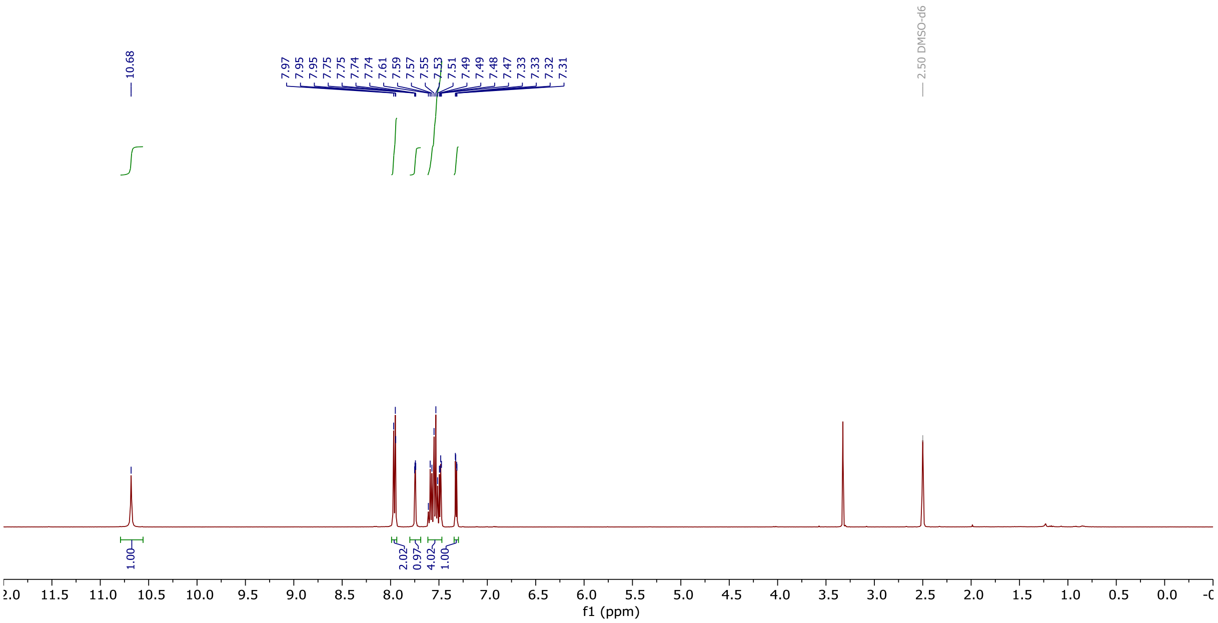

^{1}H NMR spectrum of **14**.

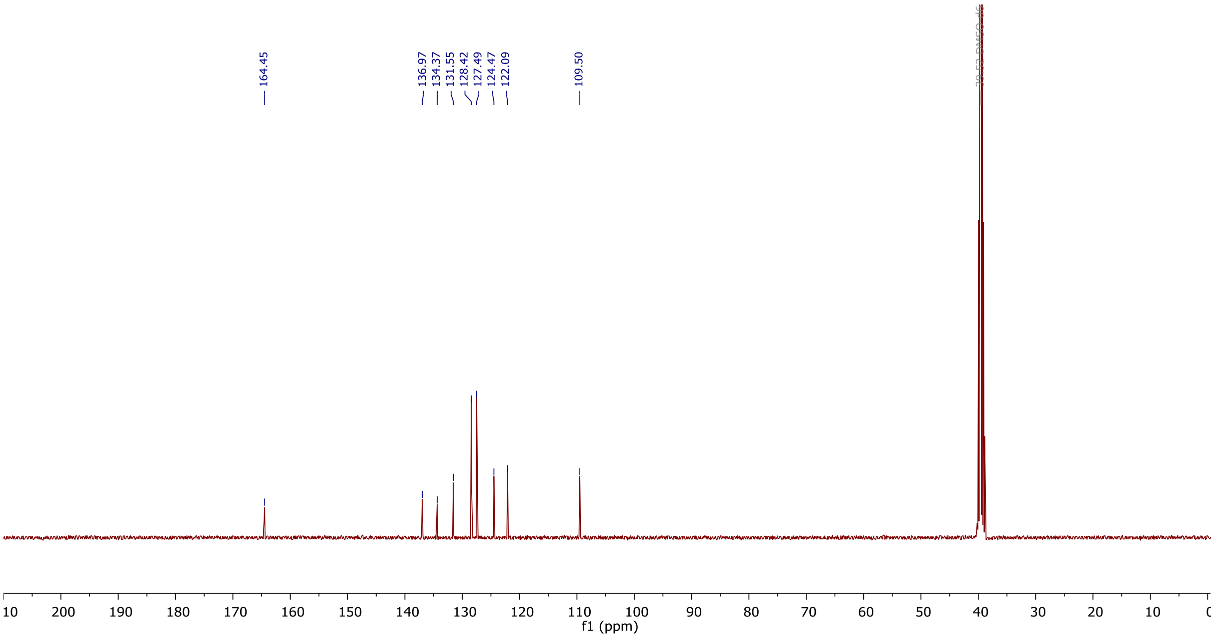

^{13}C NMR spectrum of **14**.

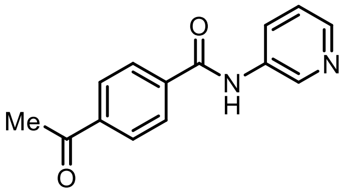

^{1}H NMR spectrum of **15**.

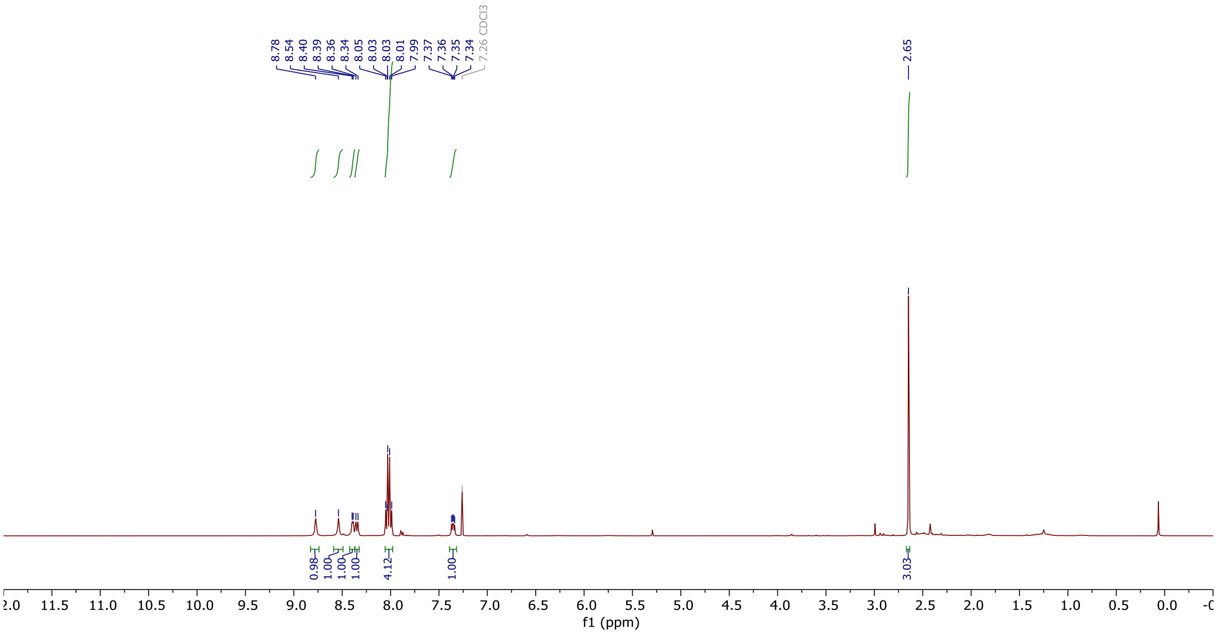

^{13}C NMR spectrum of **15**.

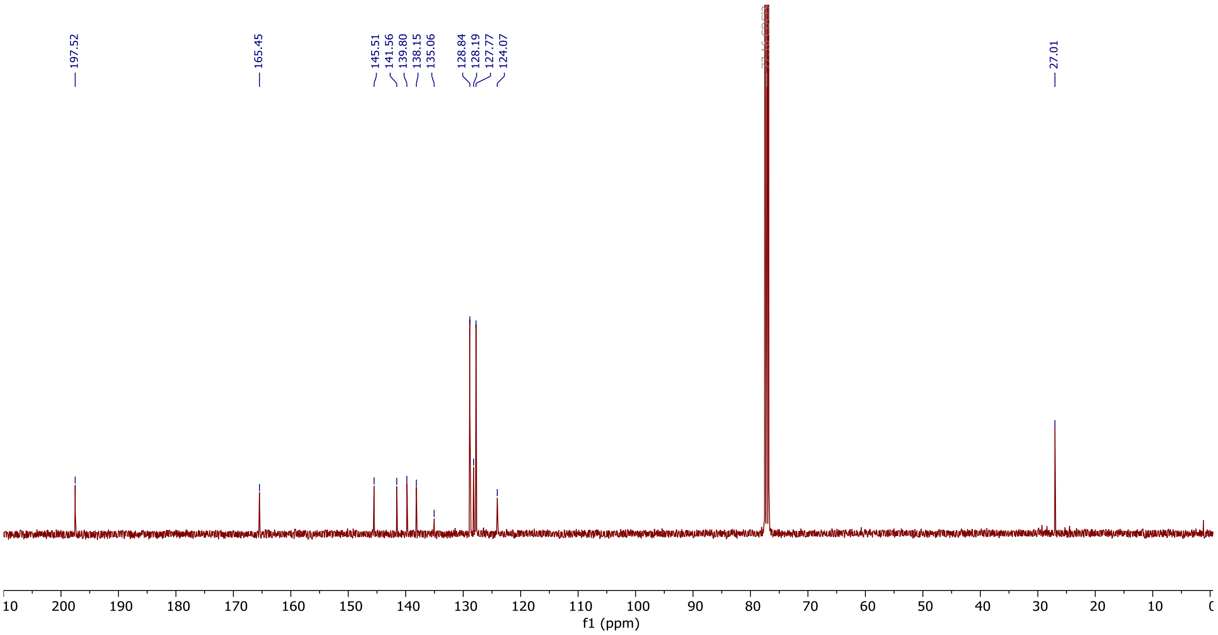

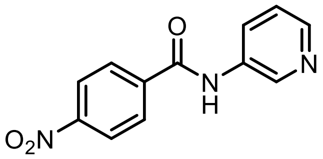

^{1}H NMR spectrum of **16**.

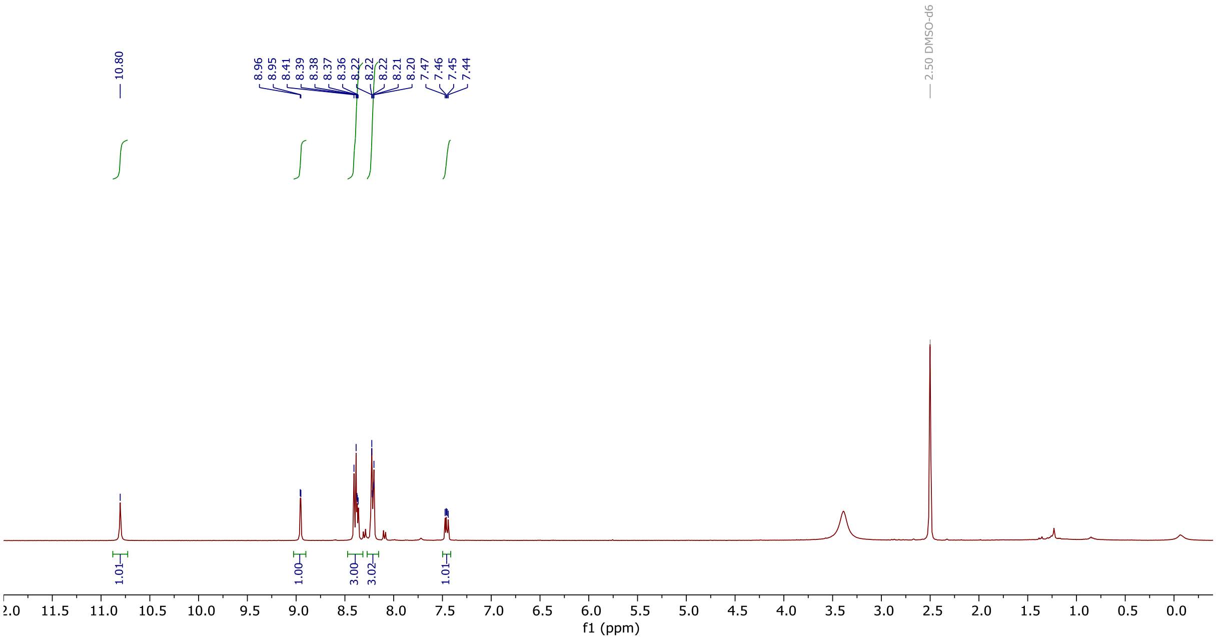

^{13}C NMR spectrum of **16**.

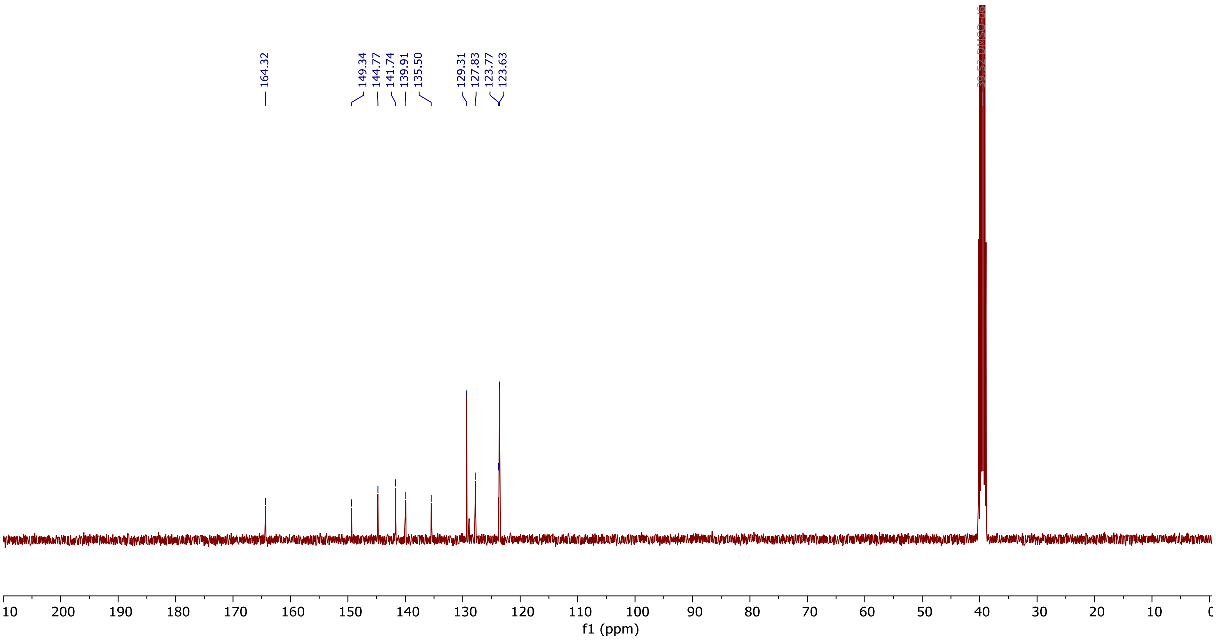

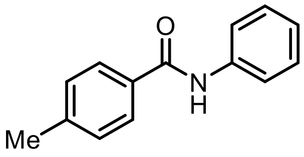

^{1}H NMR spectrum of **17**.

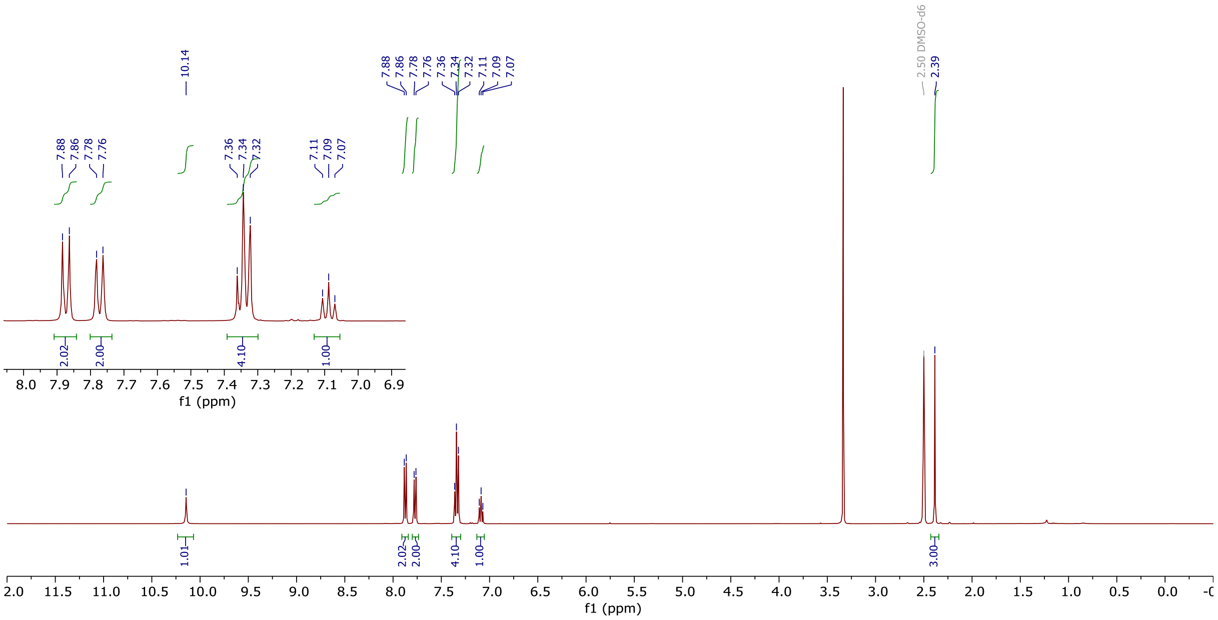

^{13}C NMR spectrum of **17**.

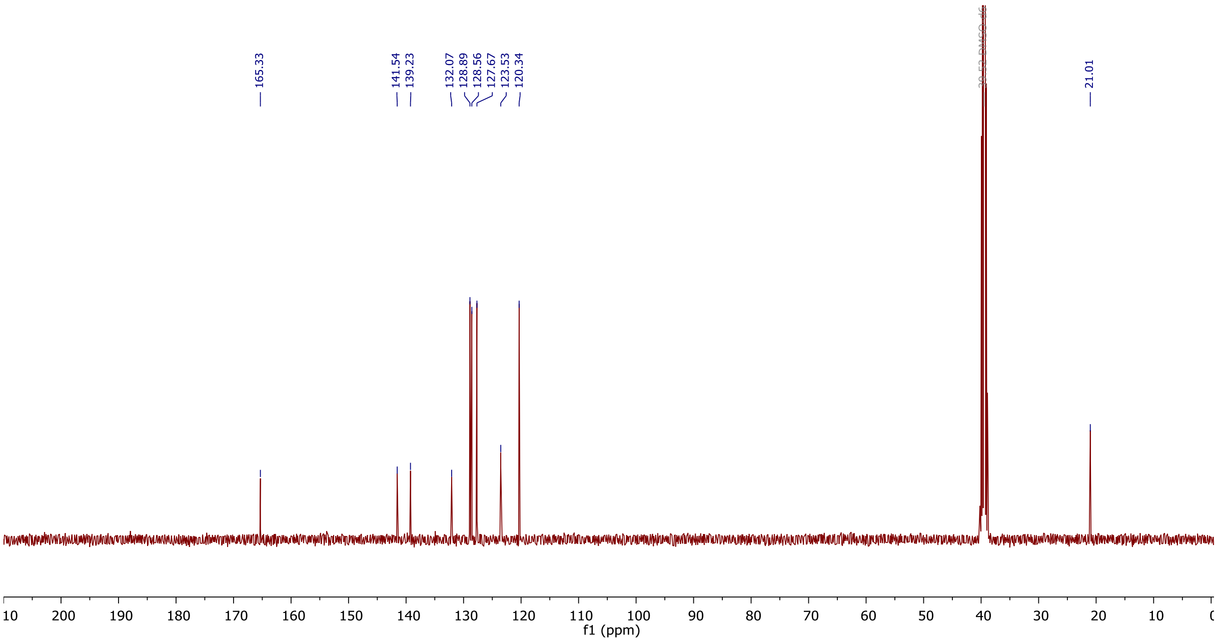

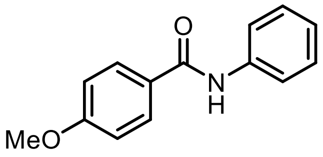

^{1}H NMR spectrum of **18**.

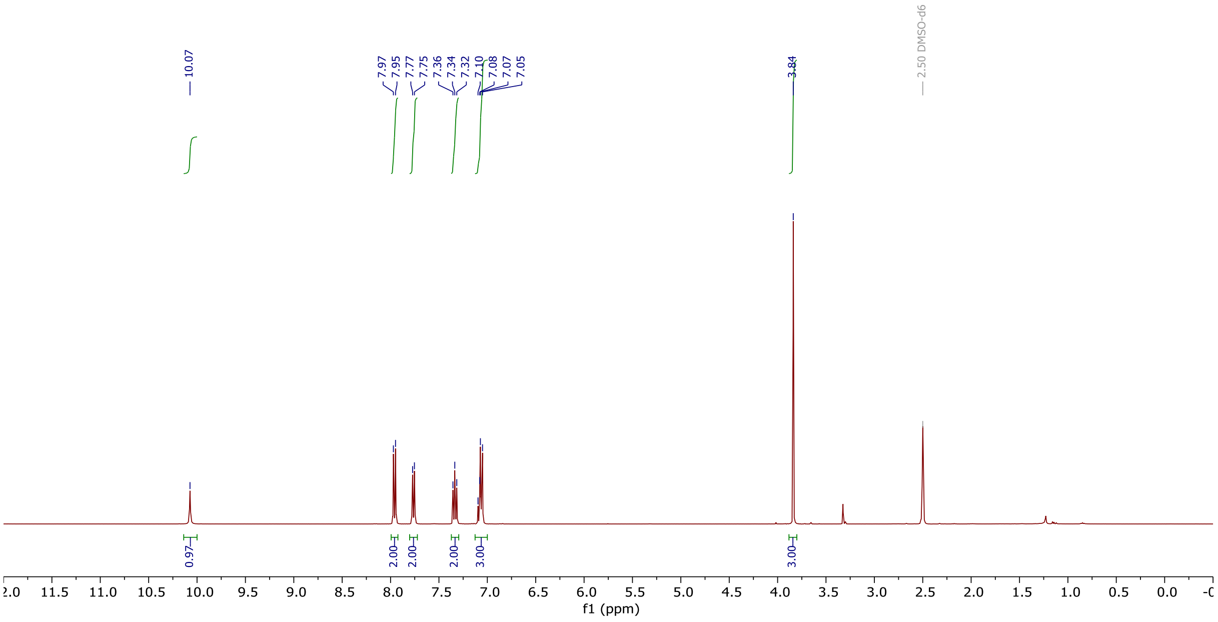

^{13}C NMR spectrum of **18**.

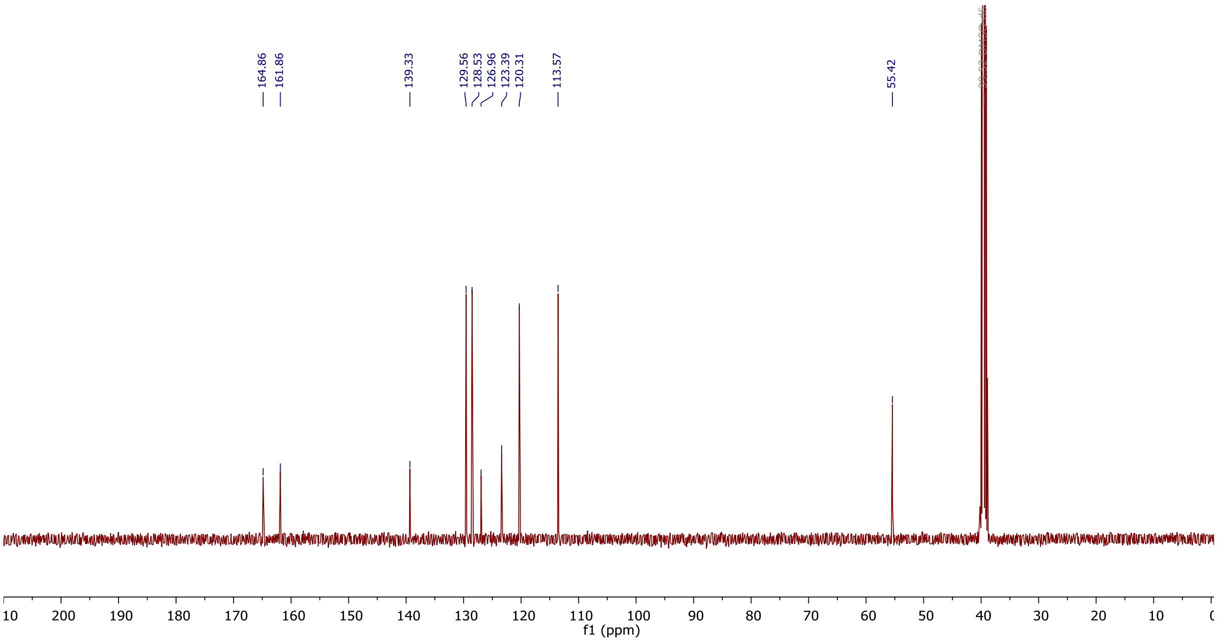

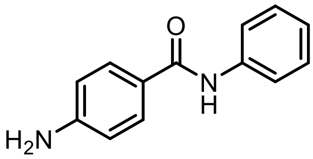

^{1}H NMR spectrum of **19**.

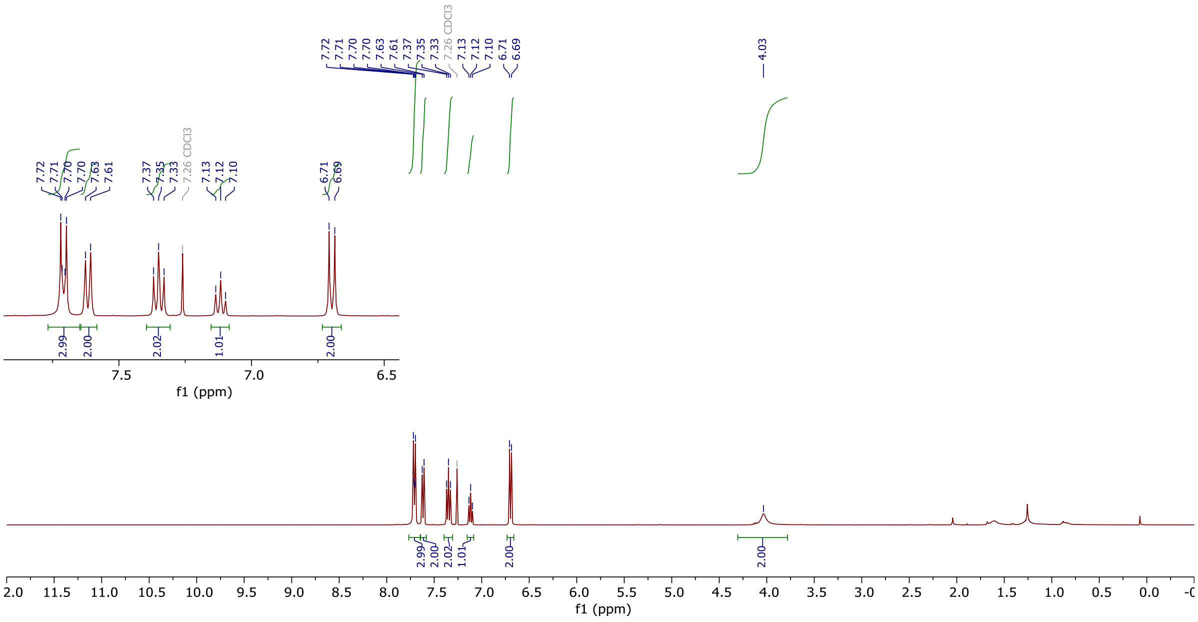

^{13}C NMR spectrum of **19**.

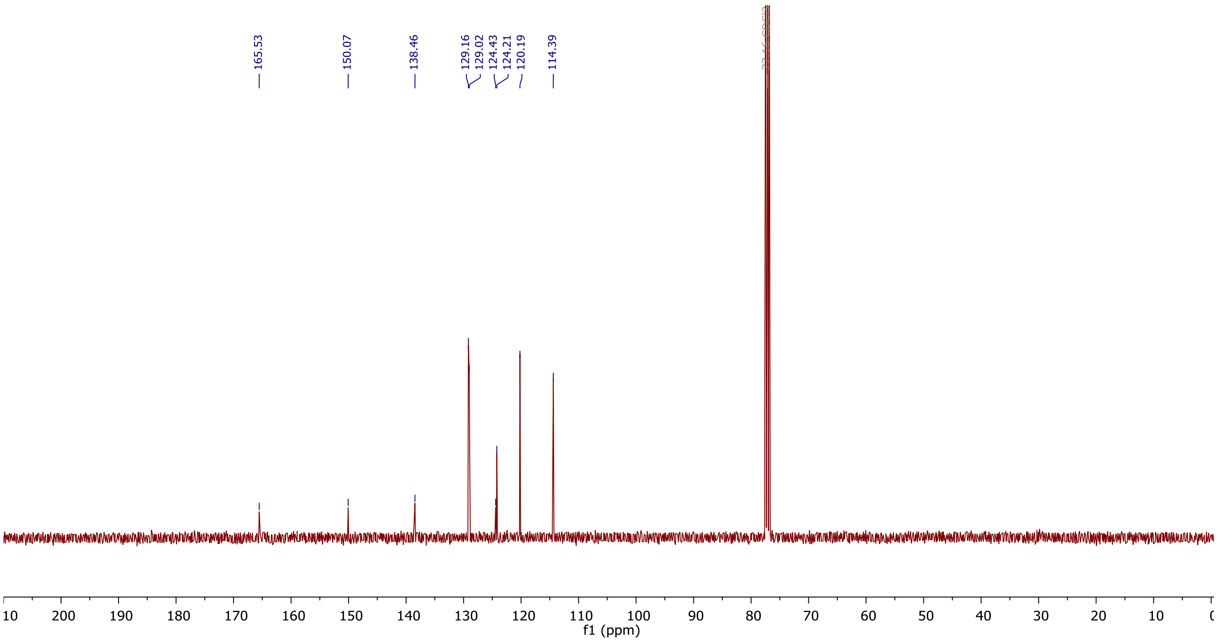

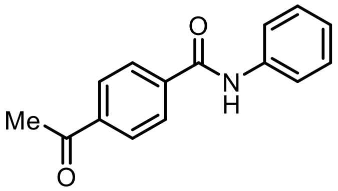

^{1}H NMR spectrum of **20**.

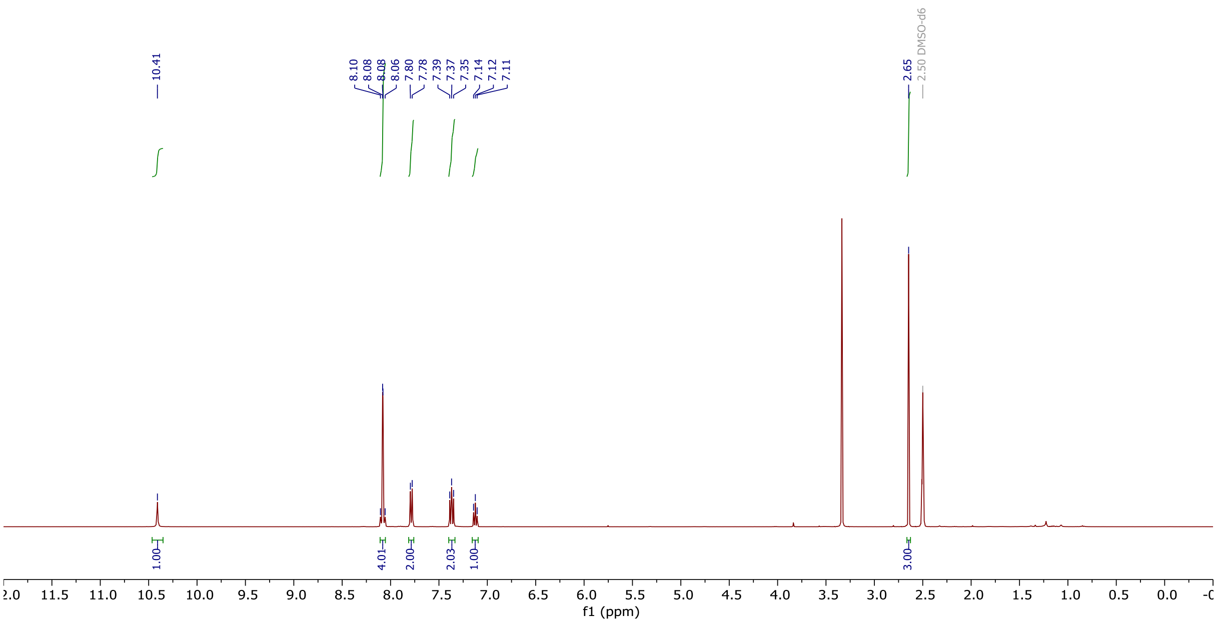

^{13}C NMR spectrum of **20**.

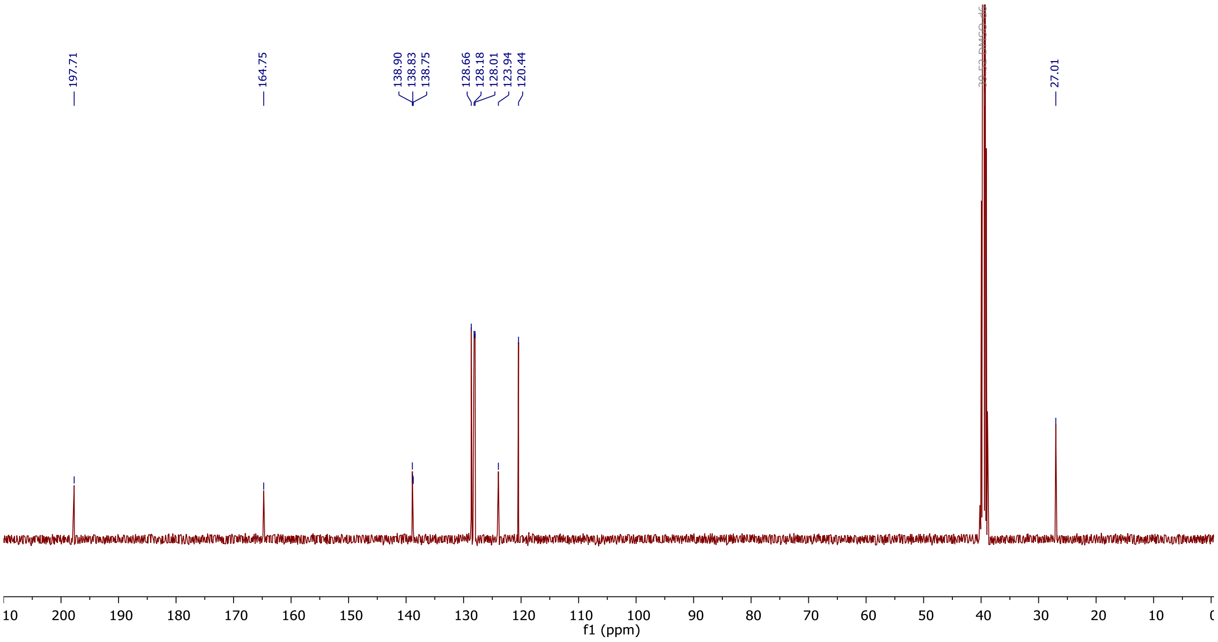

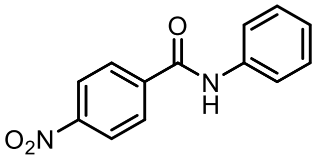

^{1}H NMR spectrum of **21**.

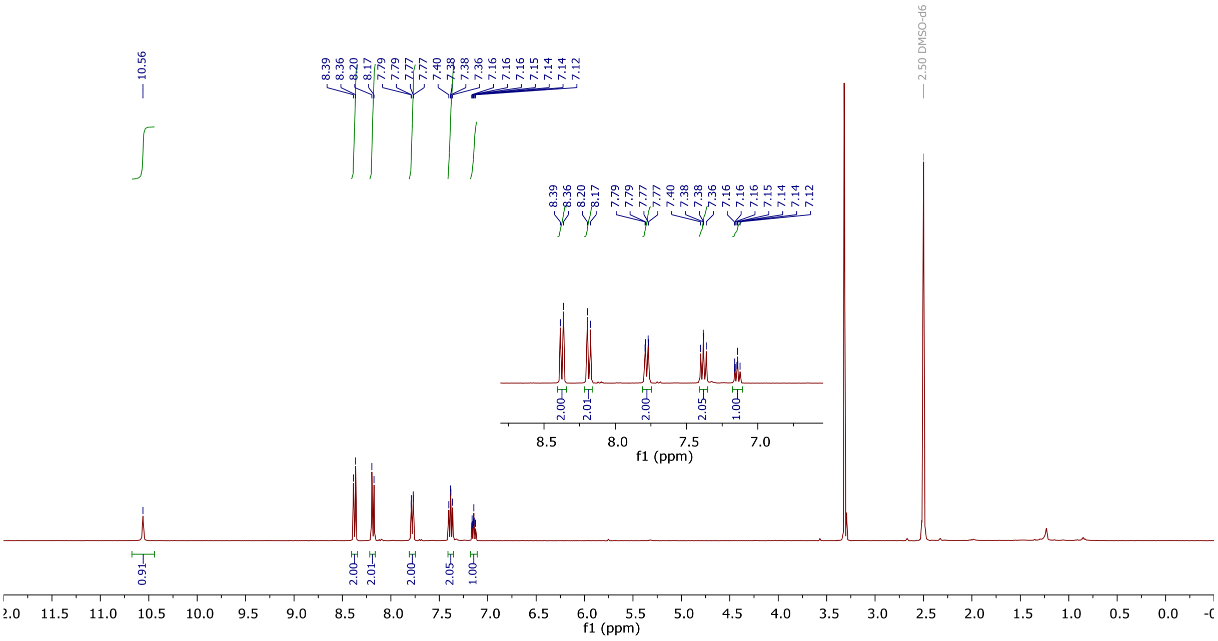

^{13}C NMR spectrum of **21**.

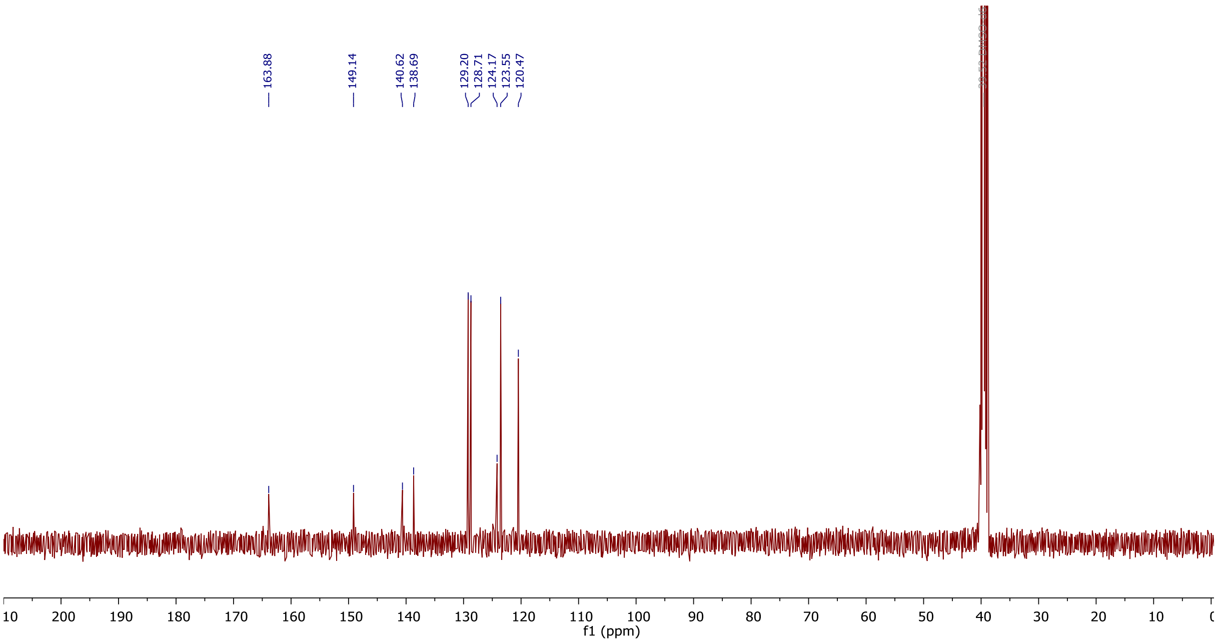

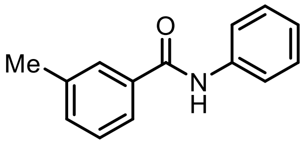

^{1}H NMR spectrum of **22**.

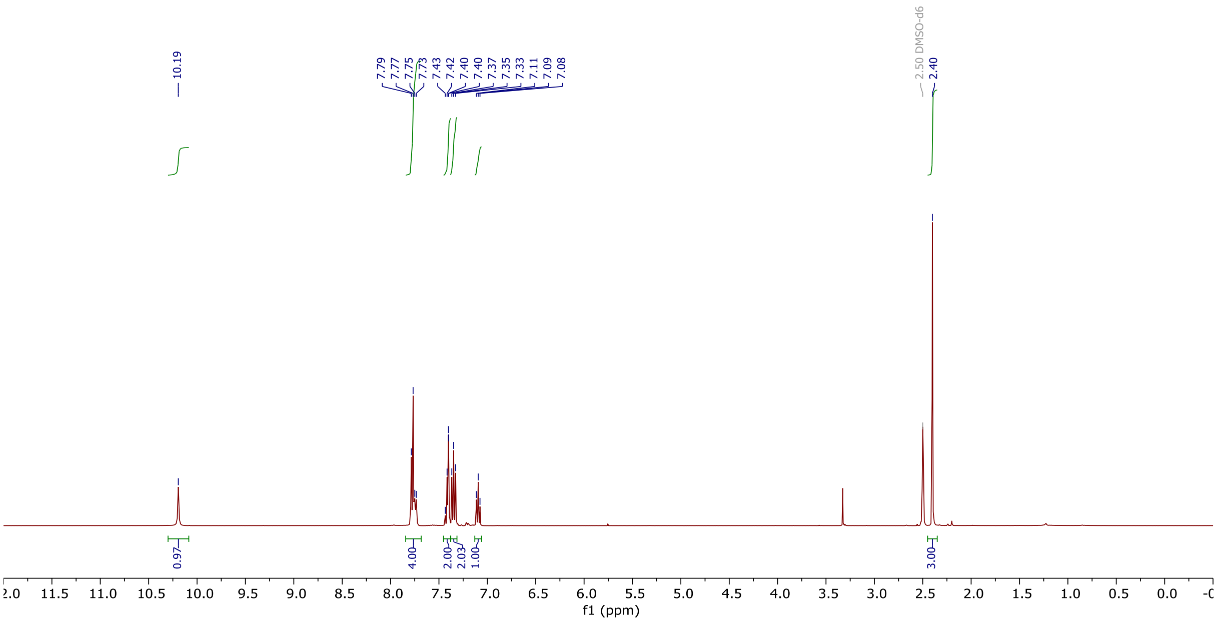

^{13}C NMR spectrum of **22**.

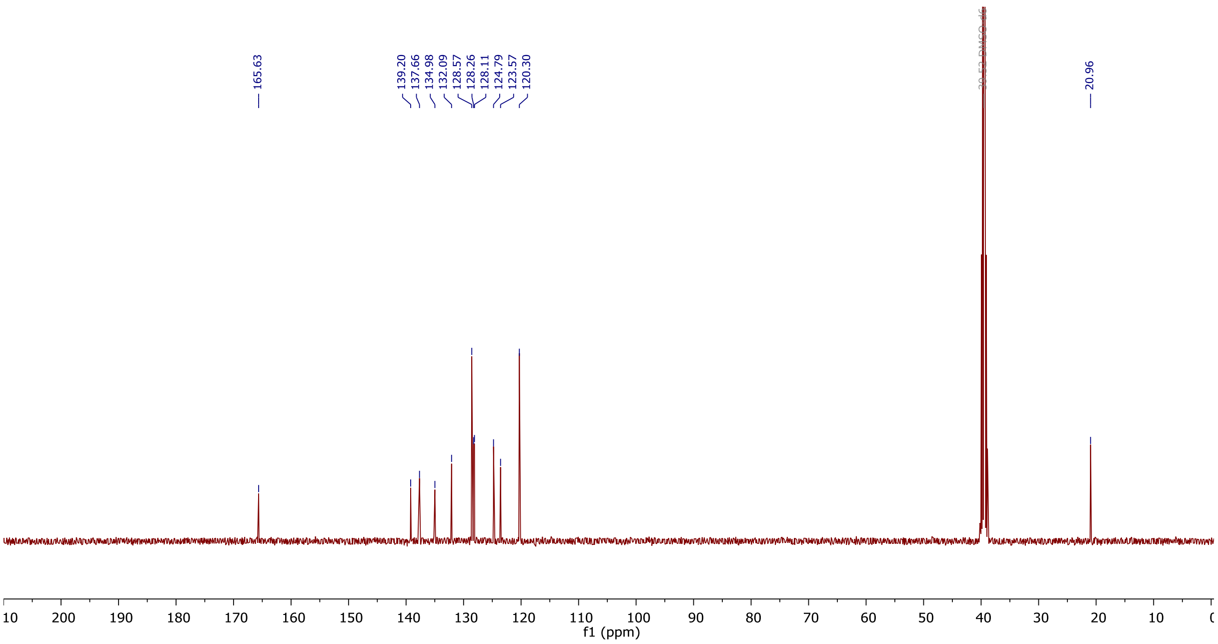

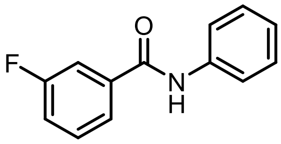

^{1}H NMR spectrum of **23**.

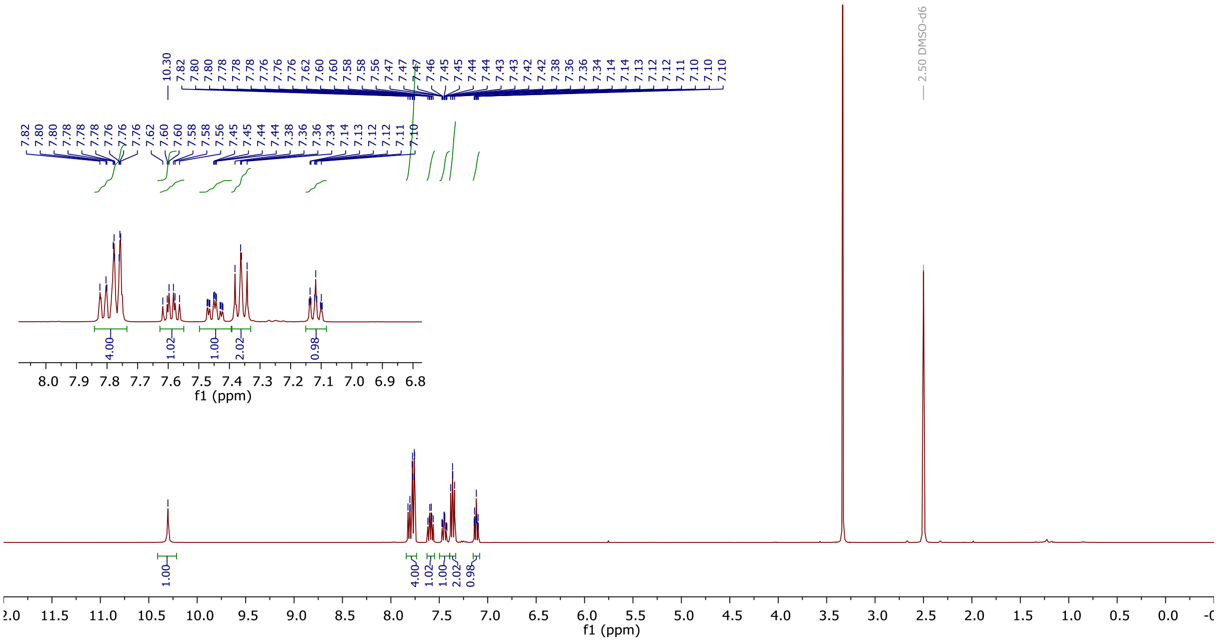

^{13}C NMR spectrum of **23**.

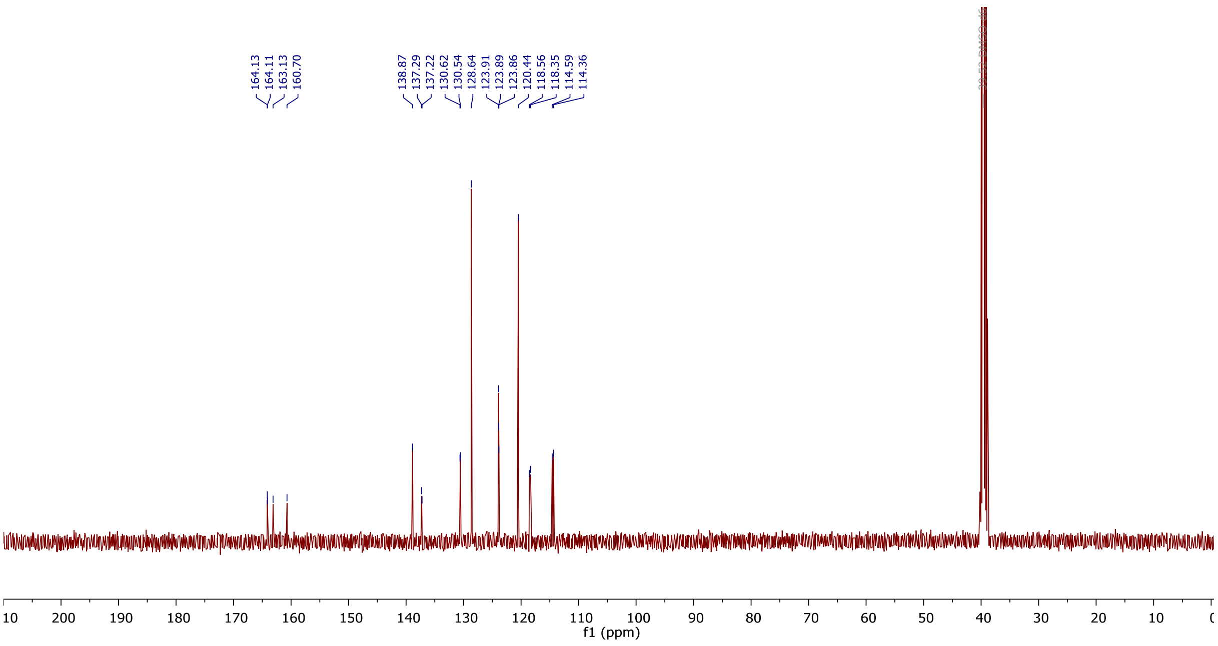

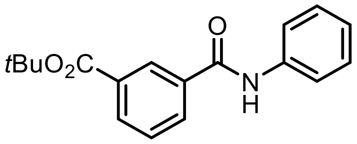

^{1}H NMR spectrum of **24**.

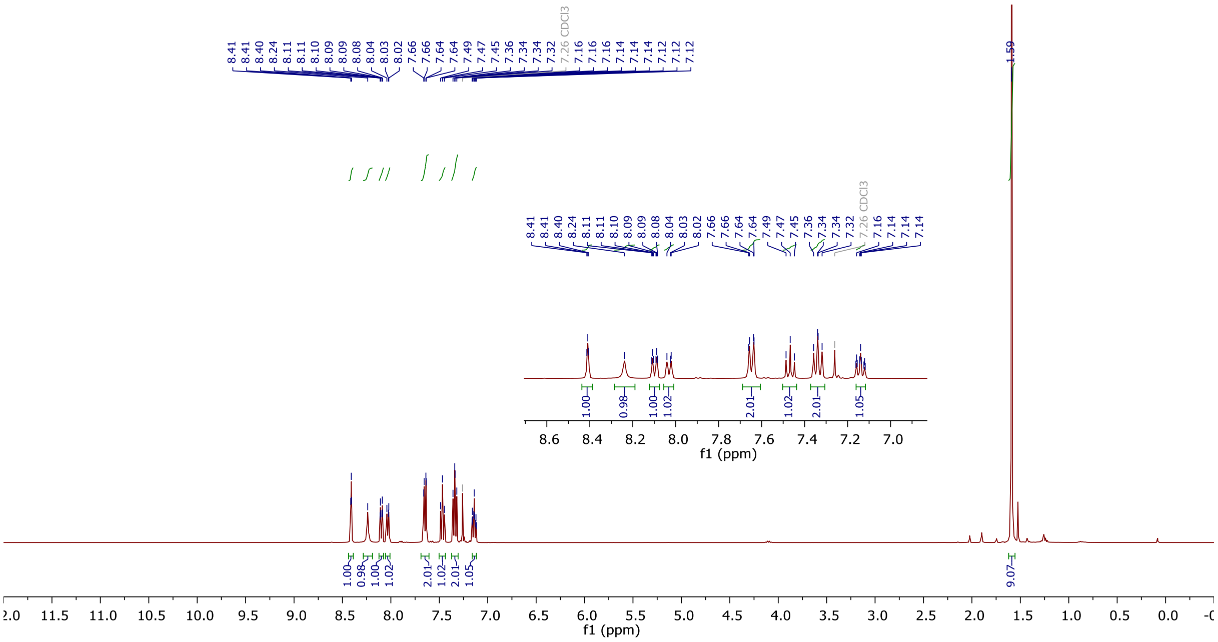

^{13}C NMR spectrum of **24**.

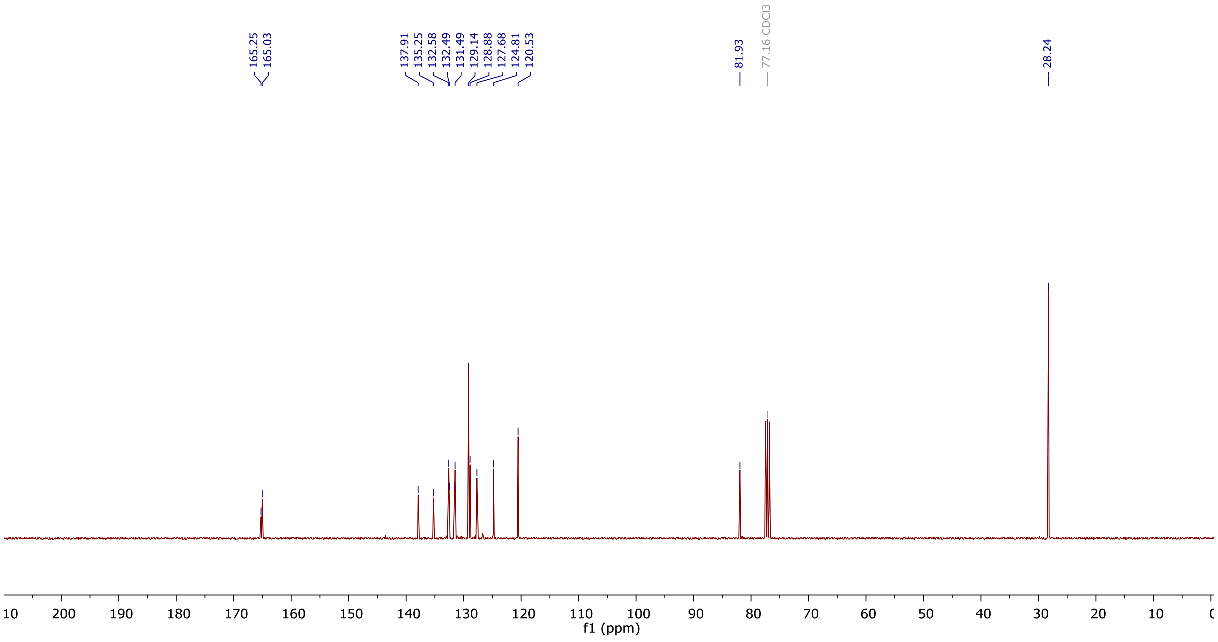

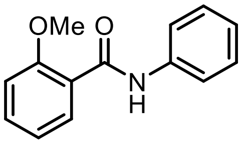

^{1}H NMR spectrum of **25**.

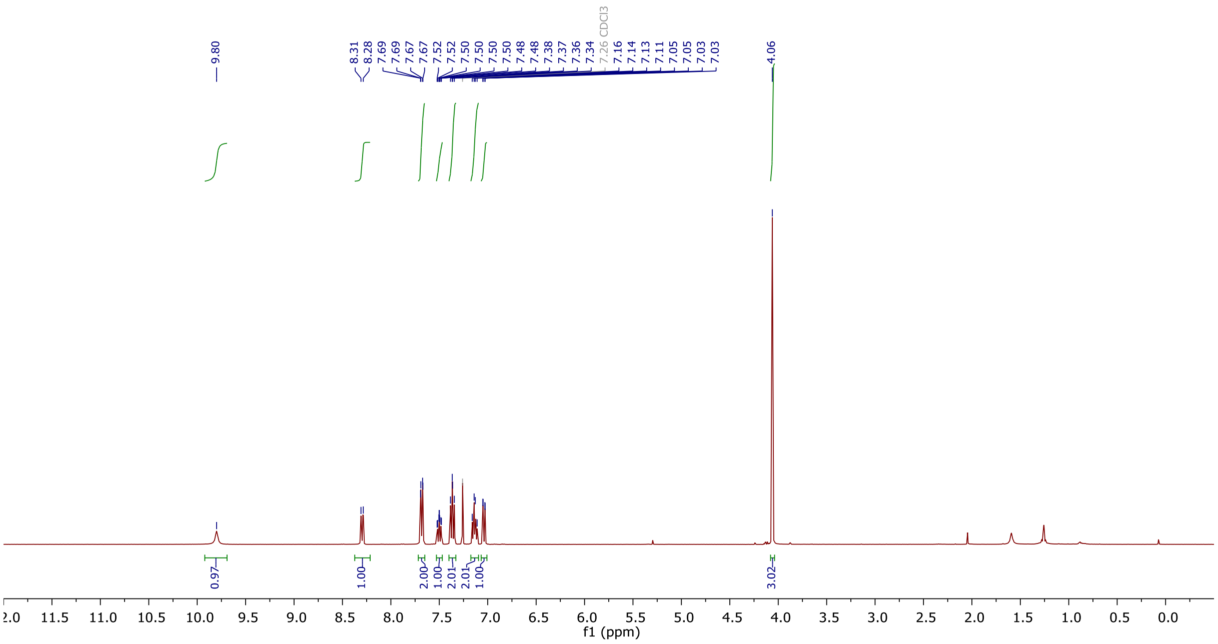

^{13}C NMR spectrum of **25**.

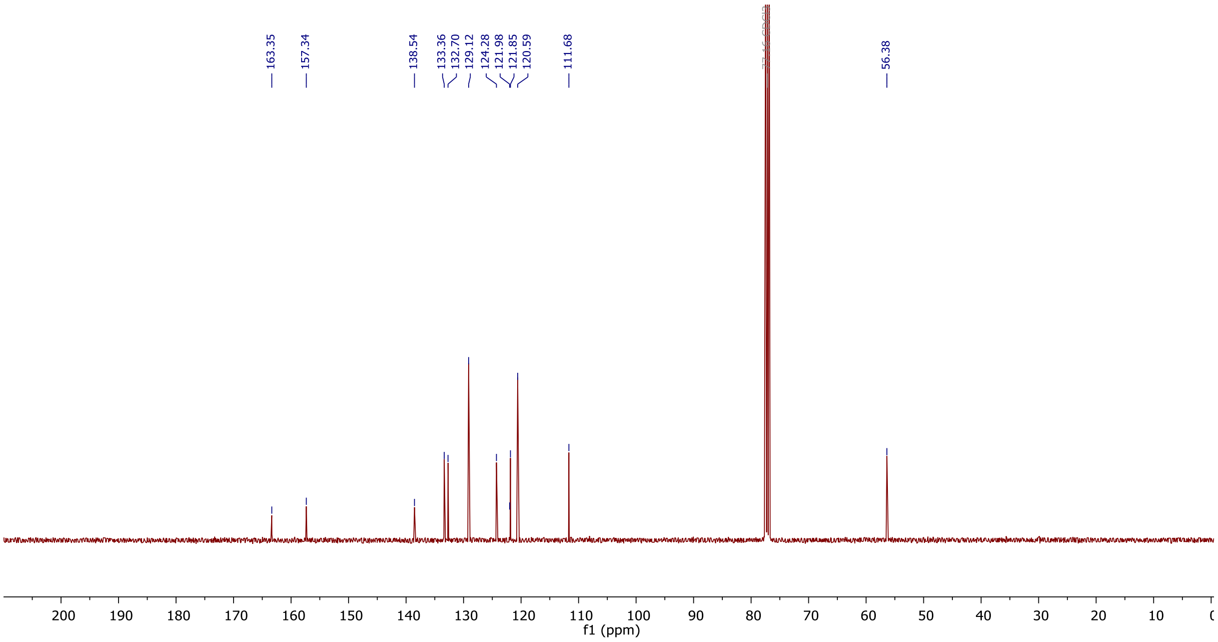

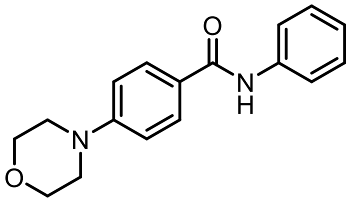

^{1}H NMR spectrum of **26**.

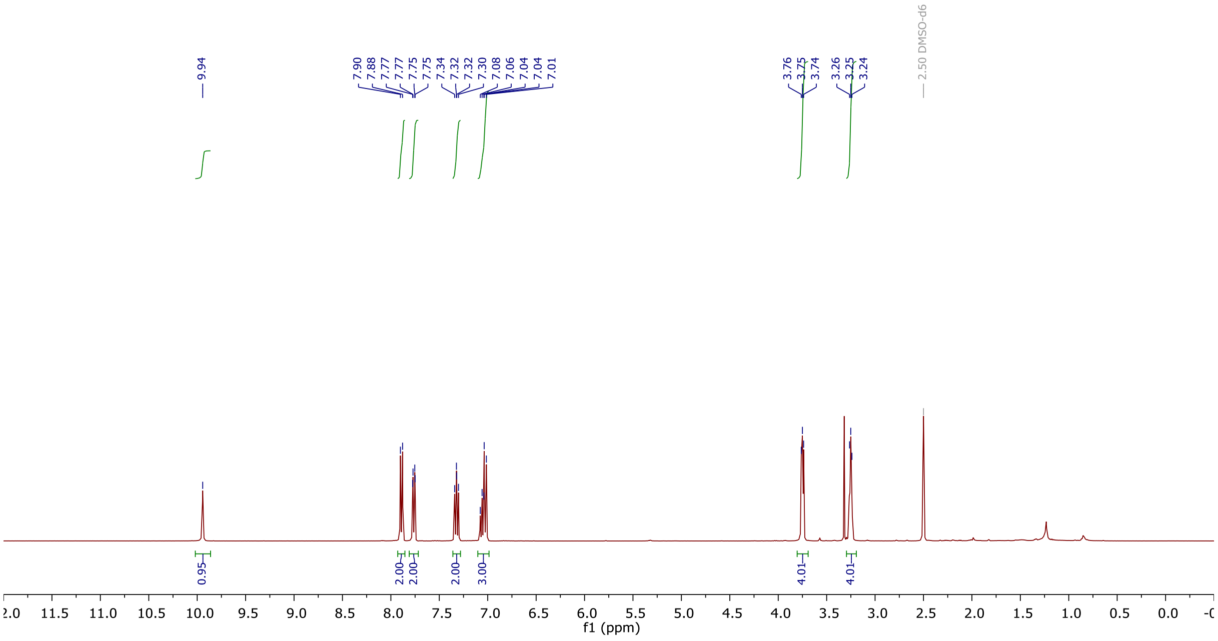

^{13}C NMR spectrum of **26**.

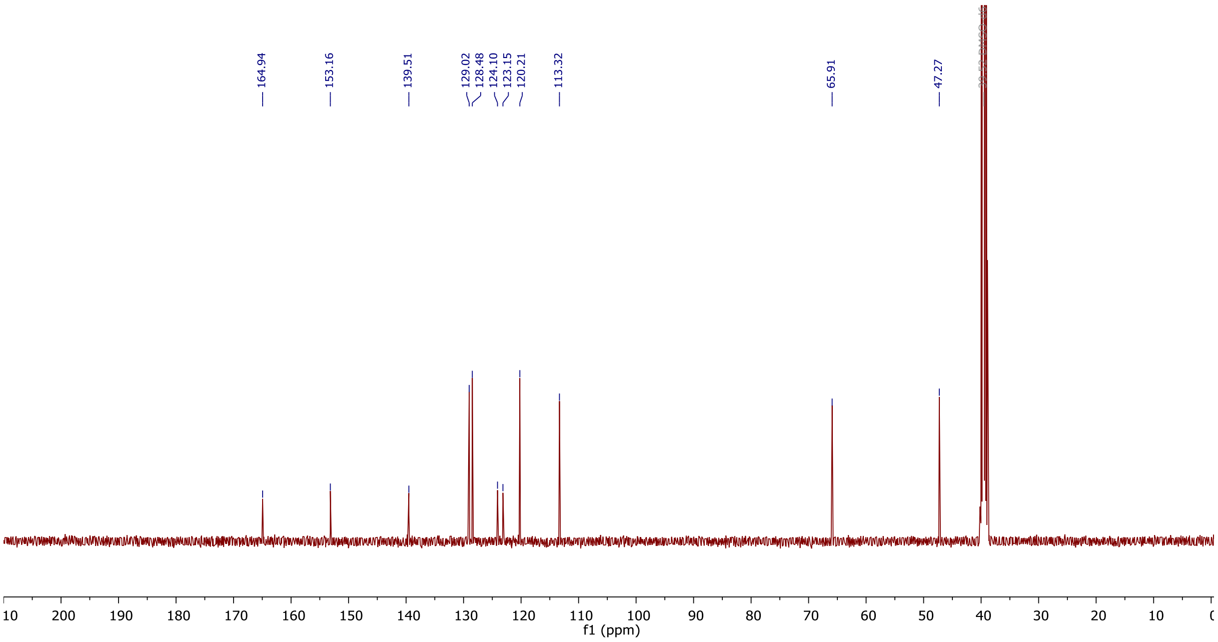

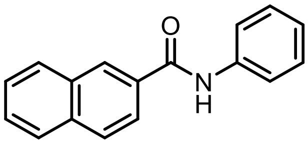

^{1}H NMR spectrum of **27**.

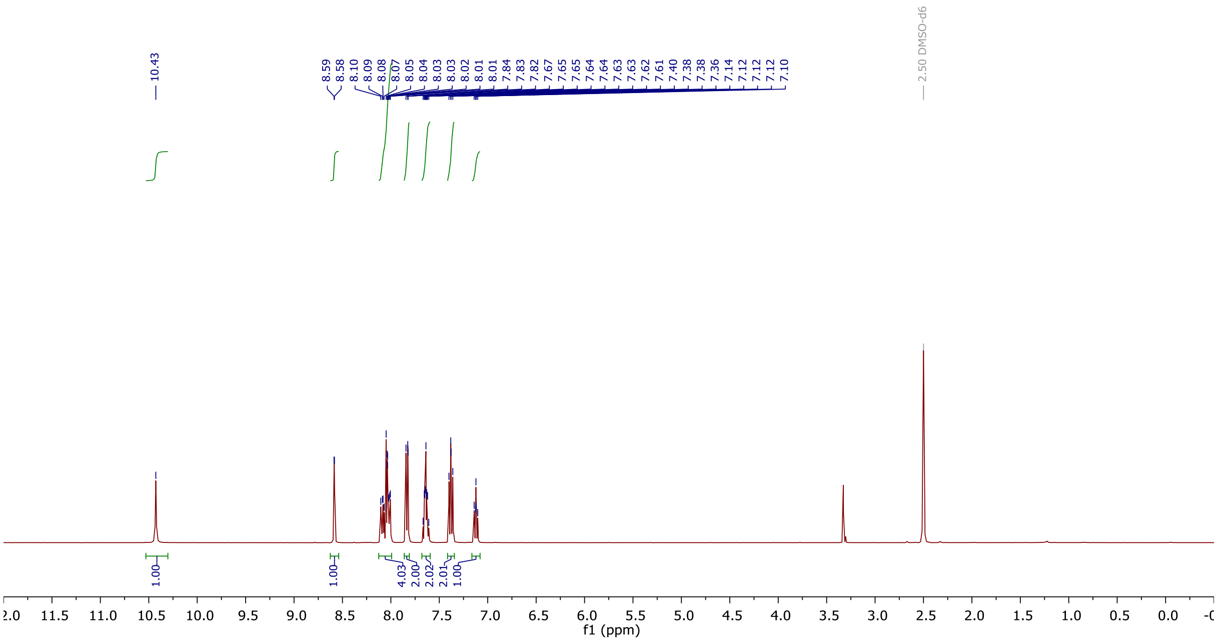

^{13}C NMR spectrum of **27**.

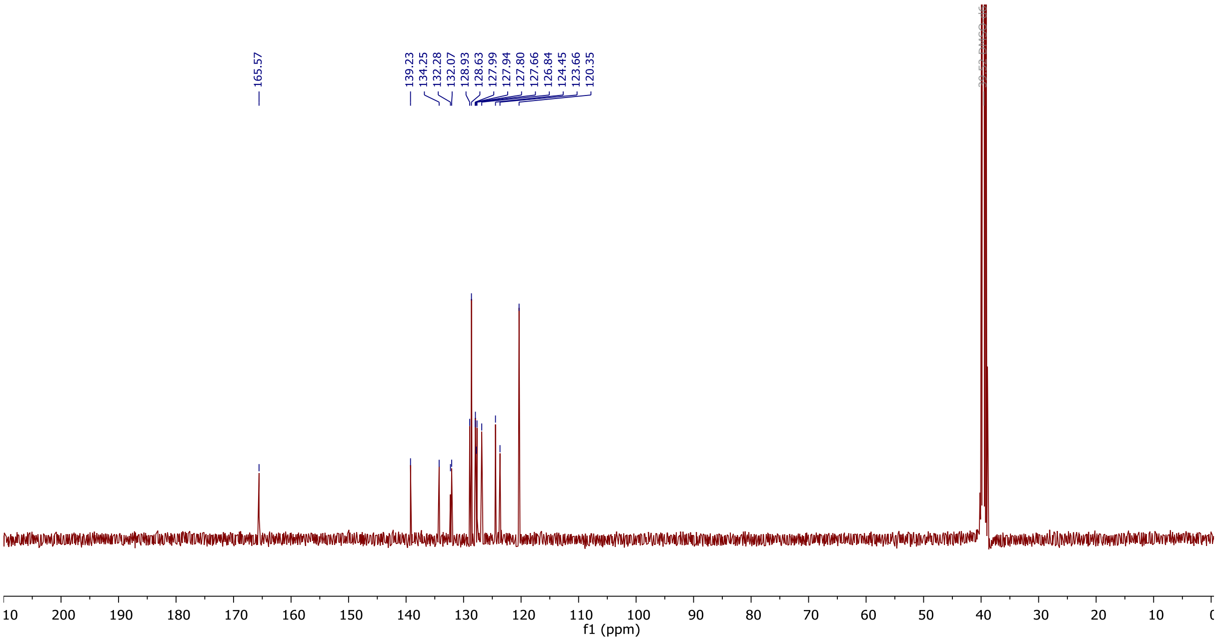

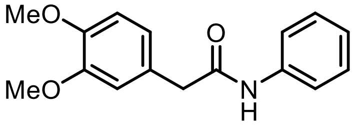

^{1}H NMR spectrum of **28**.

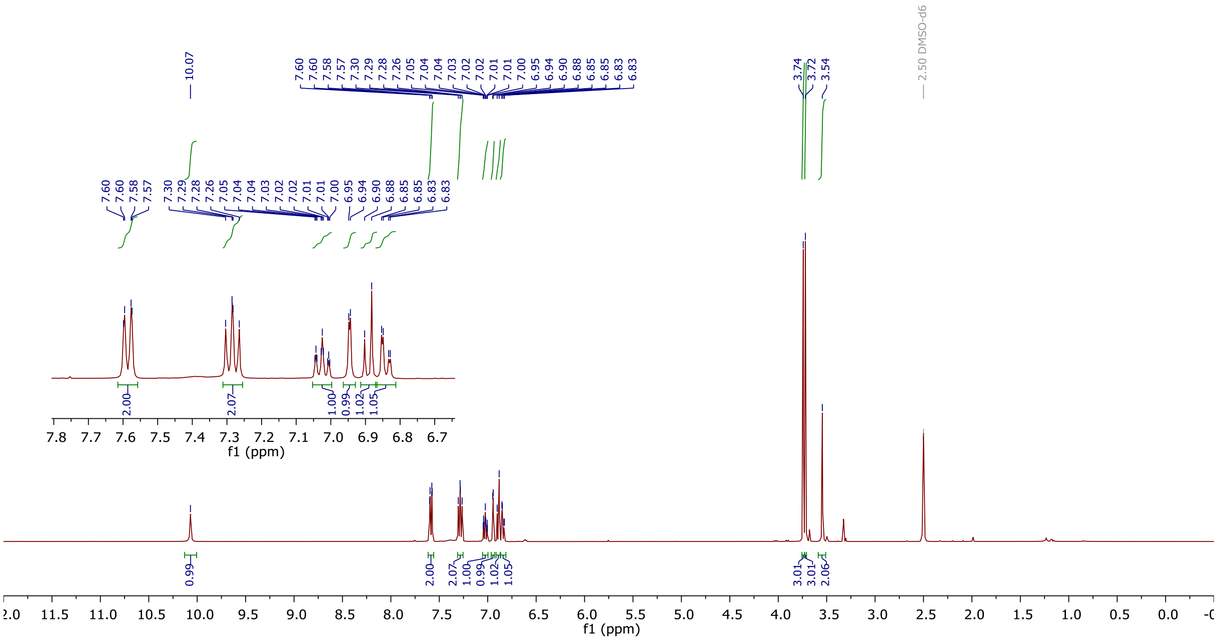

^{13}C NMR spectrum of **28**.

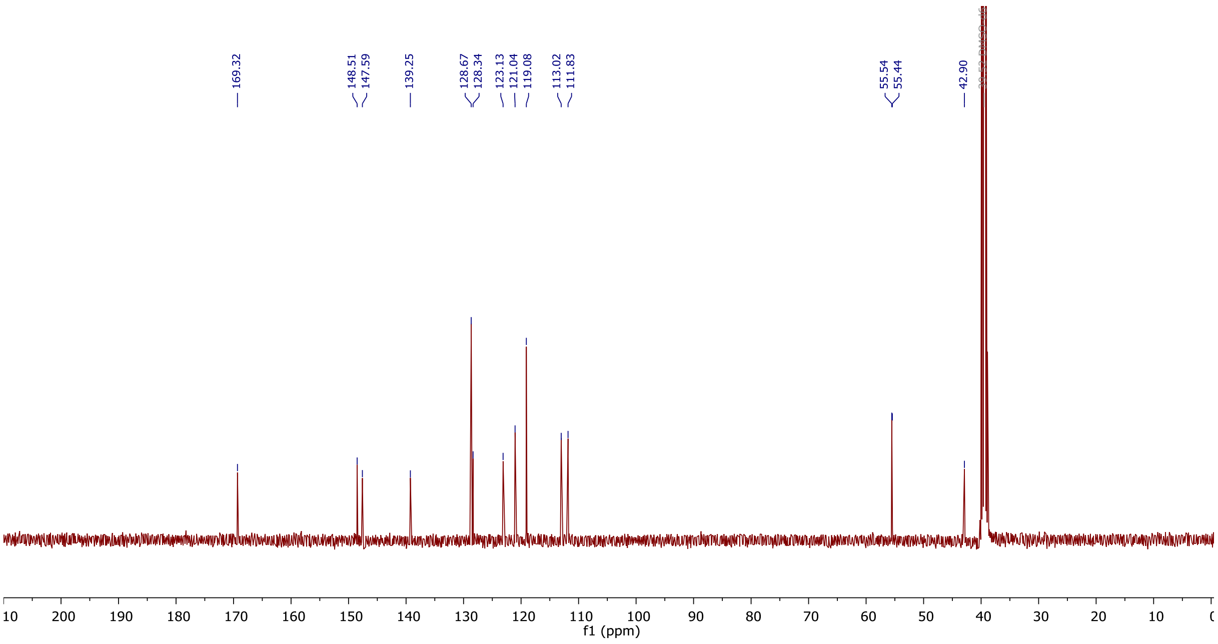

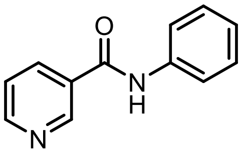

^{1}H NMR spectrum of **29**.

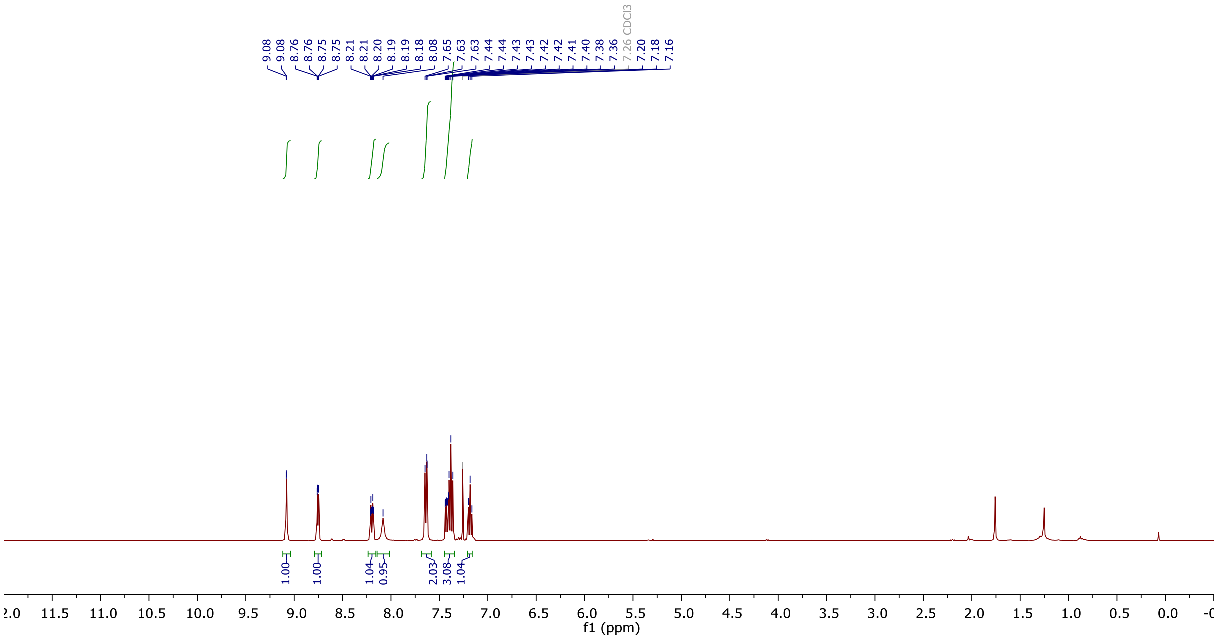

^{13}C NMR spectrum of **29**.

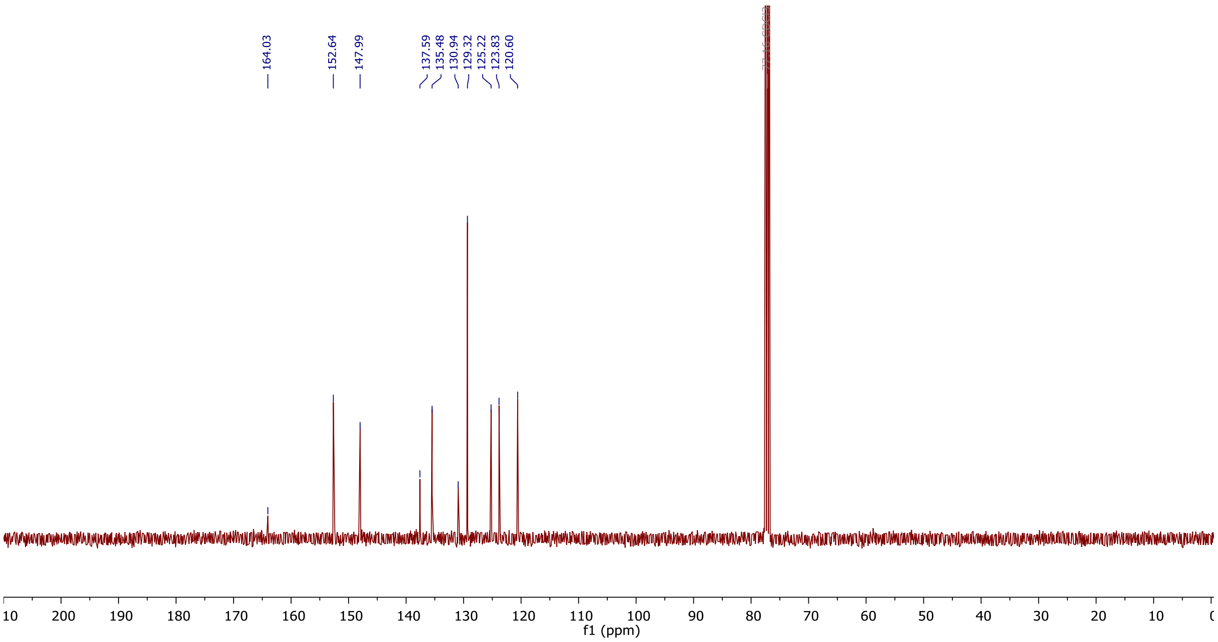

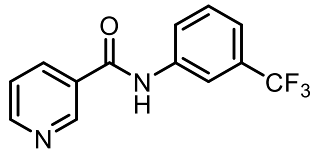

^{1}H NMR spectrum of **30**.

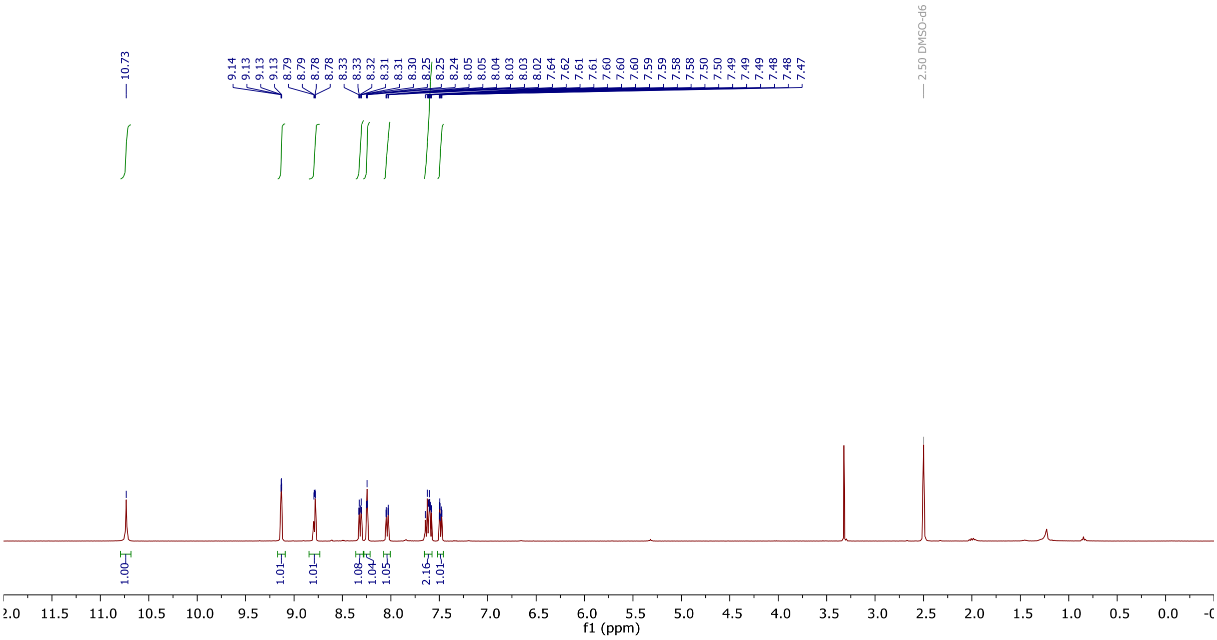

^{13}C NMR spectrum of **30**.

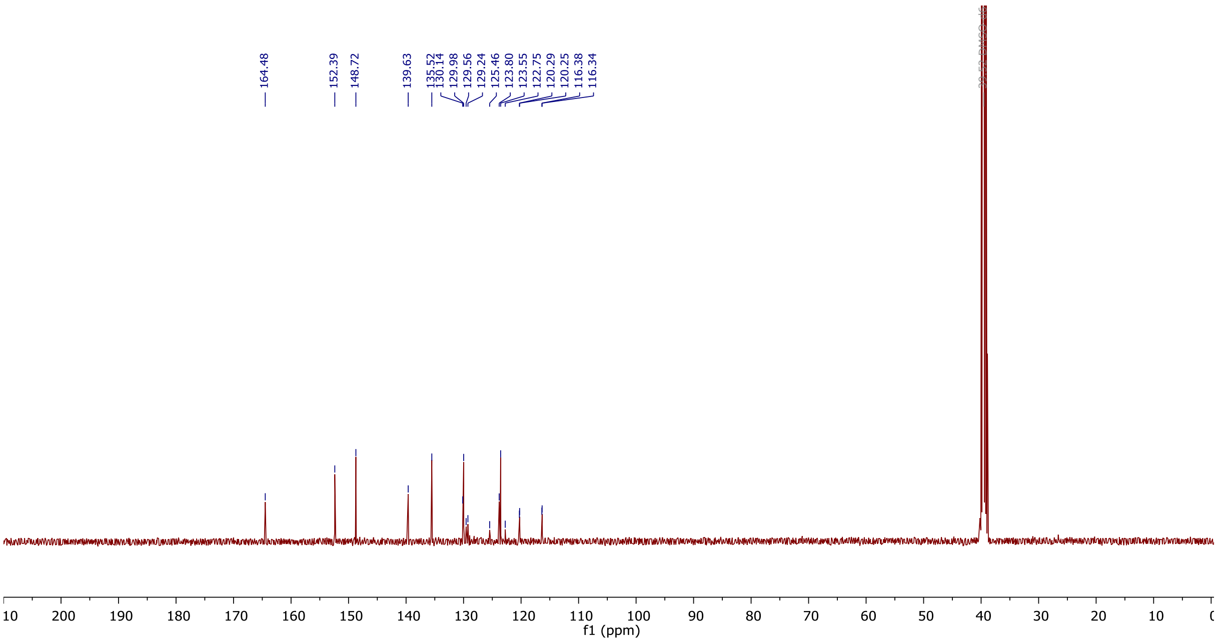

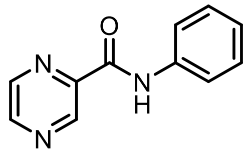

^{1}H NMR spectrum of **31**.

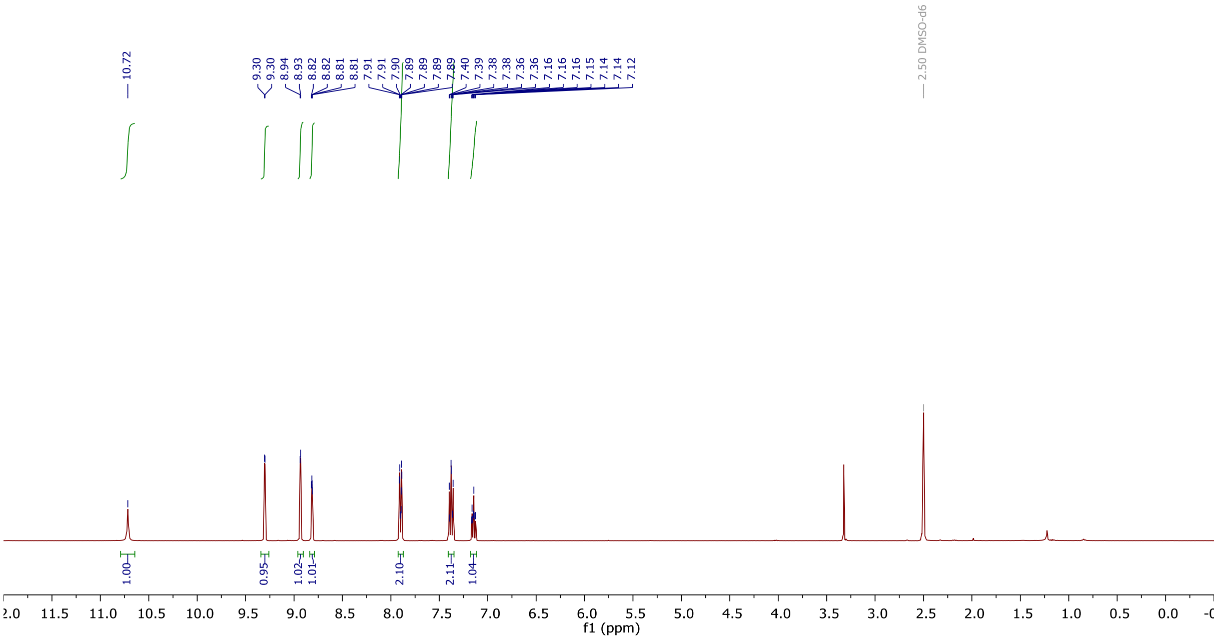

^{13}C NMR spectrum of **31**.

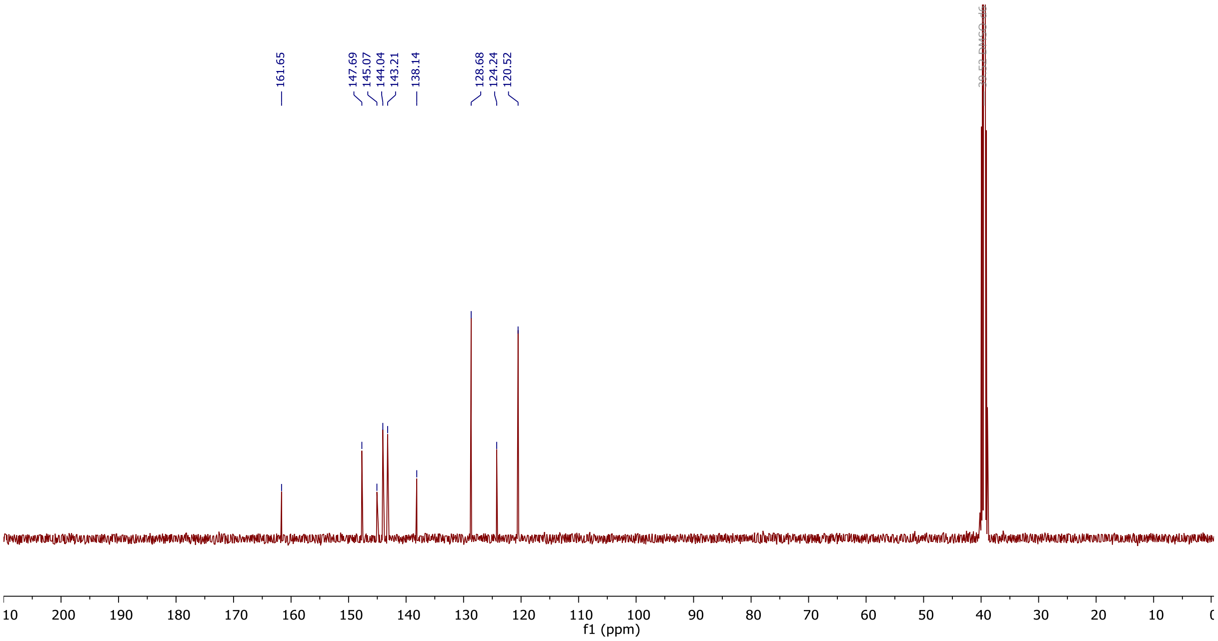

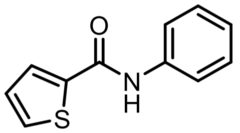

^{1}H NMR spectrum of **32**.

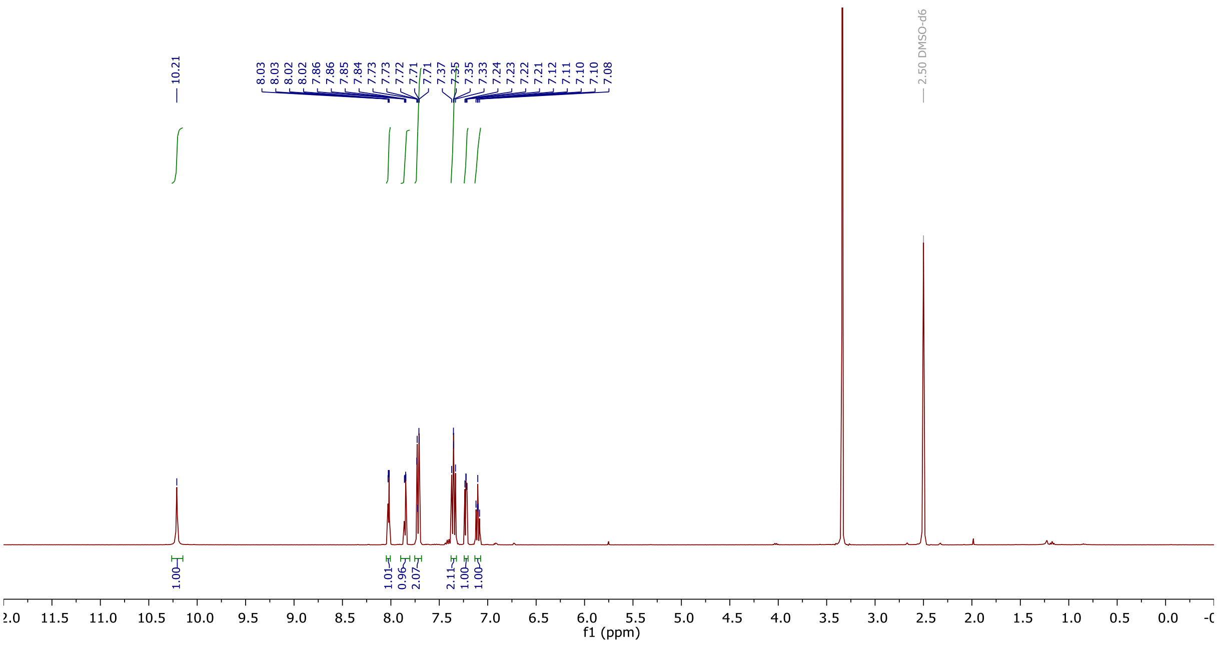

^{13}C NMR spectrum of **32**.

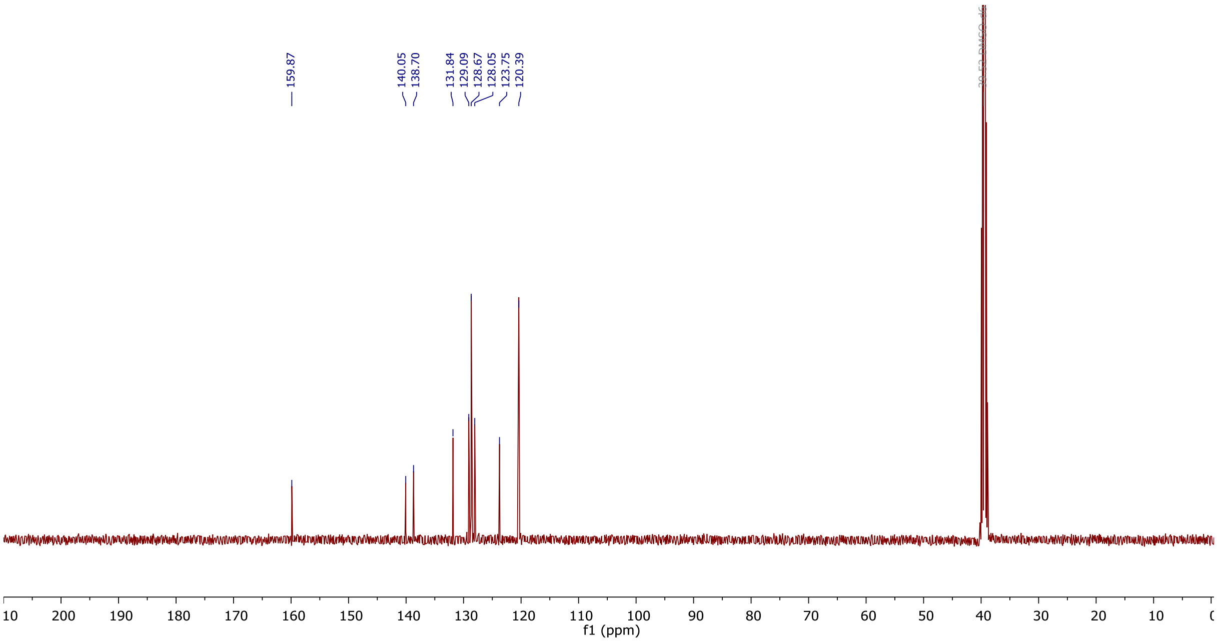

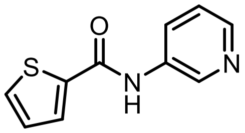

^{1}H NMR spectrum of **33**.

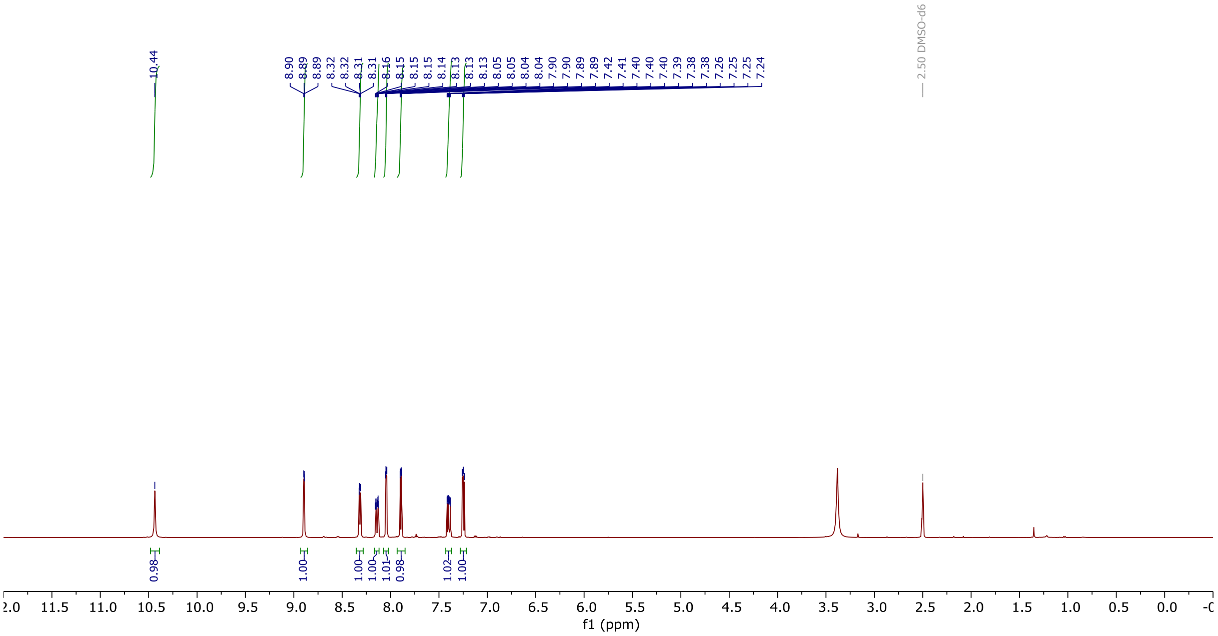

^{13}C NMR spectrum of **33**.

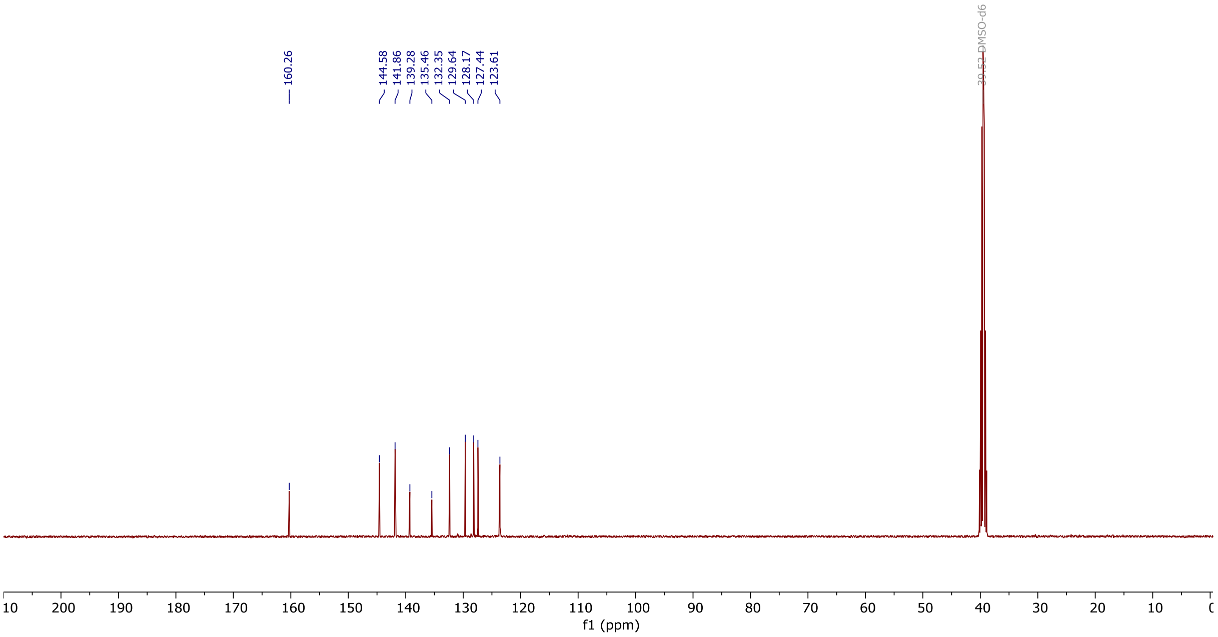

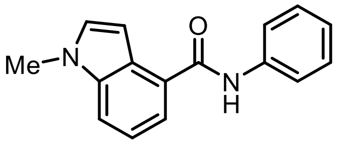

^{1}H NMR spectrum of **34**.

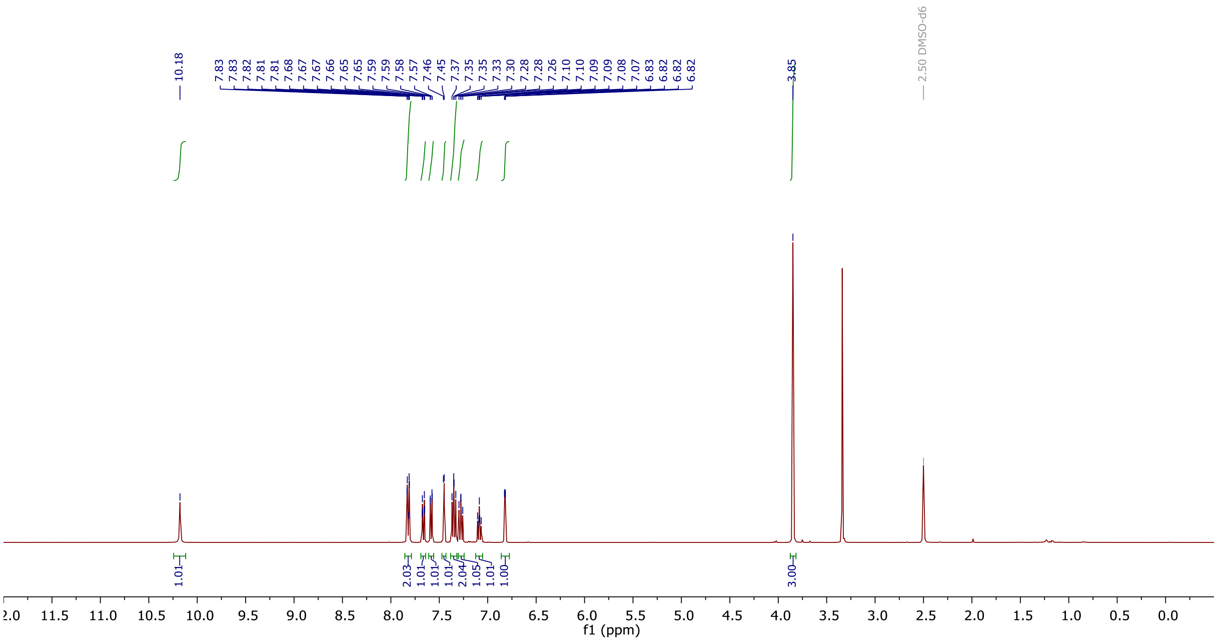

^{13}C NMR spectrum of **34**.

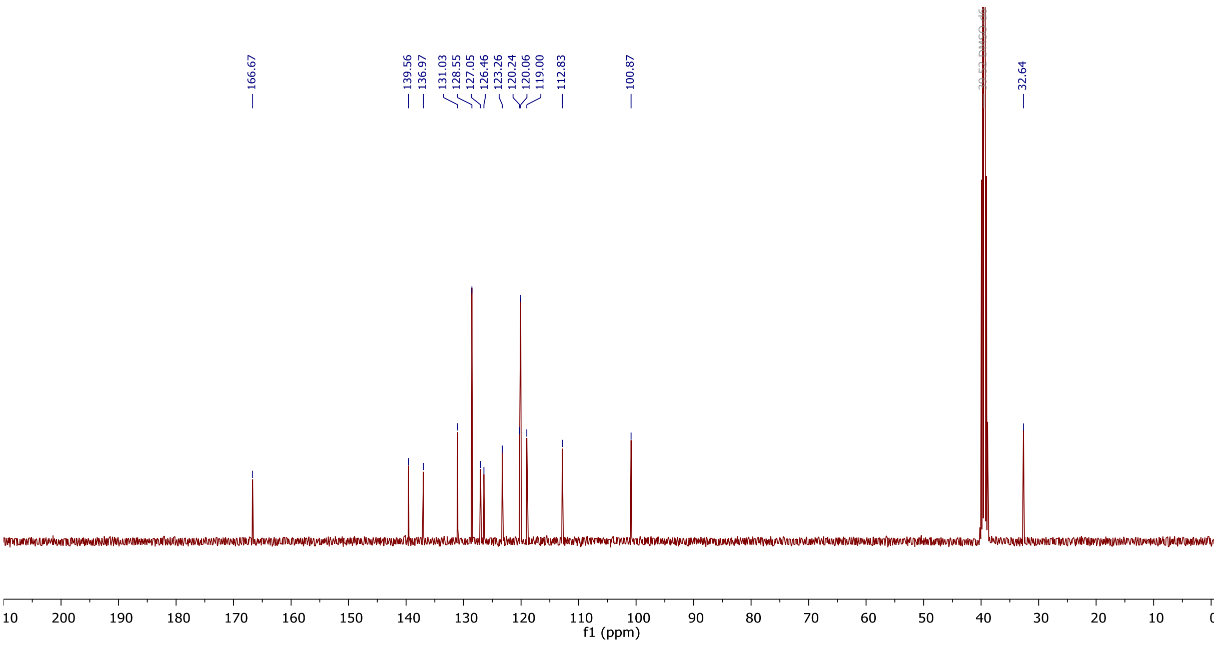

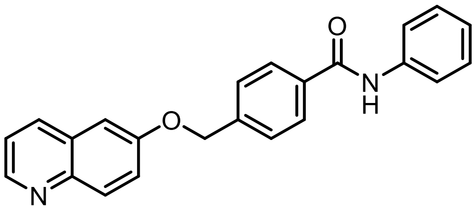

^{1}H NMR spectrum of **35**.

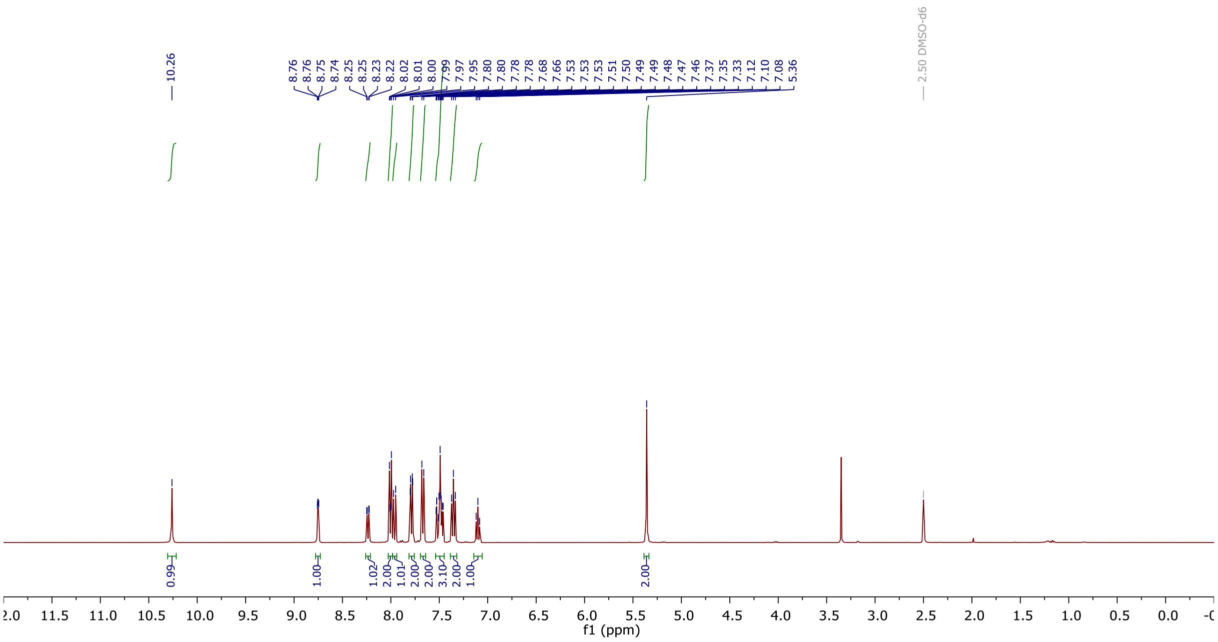

^{13}C NMR spectrum of **35**.

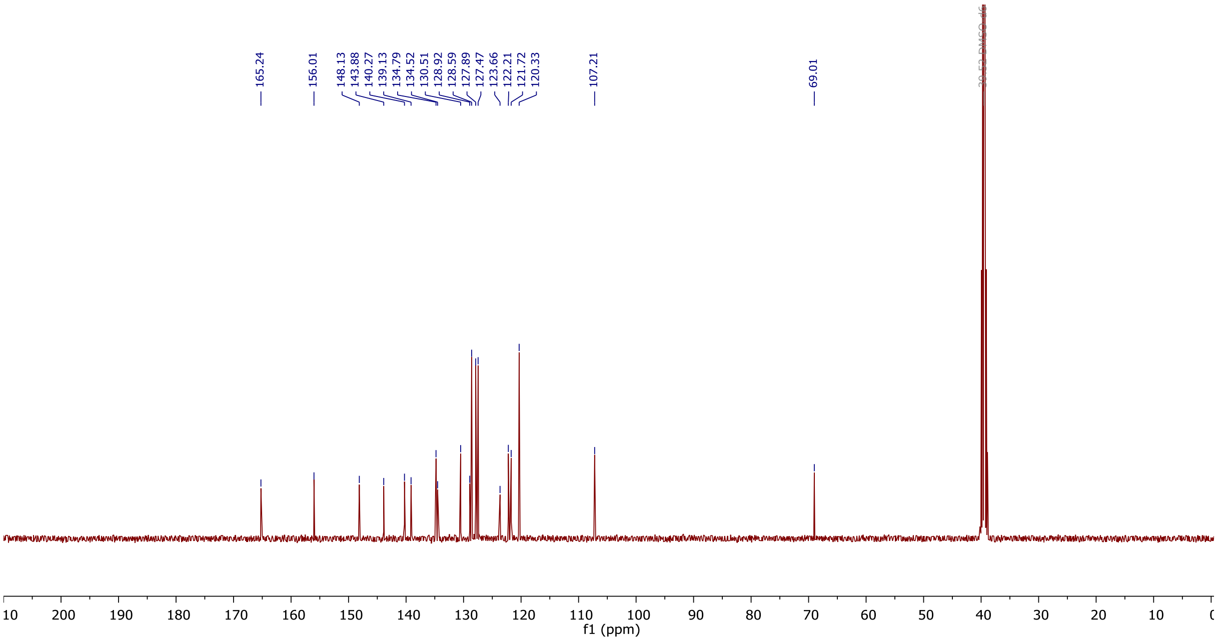

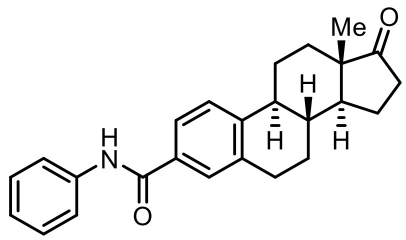

^{1}H NMR spectrum of **36**.

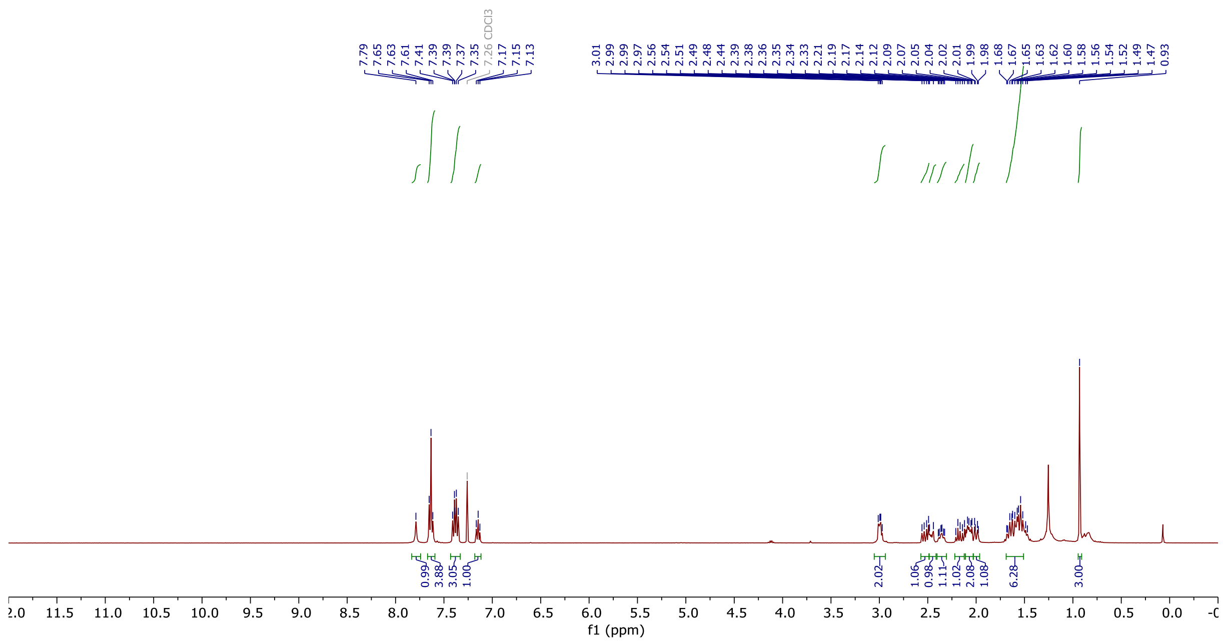

^{13}C NMR spectrum of **36**.

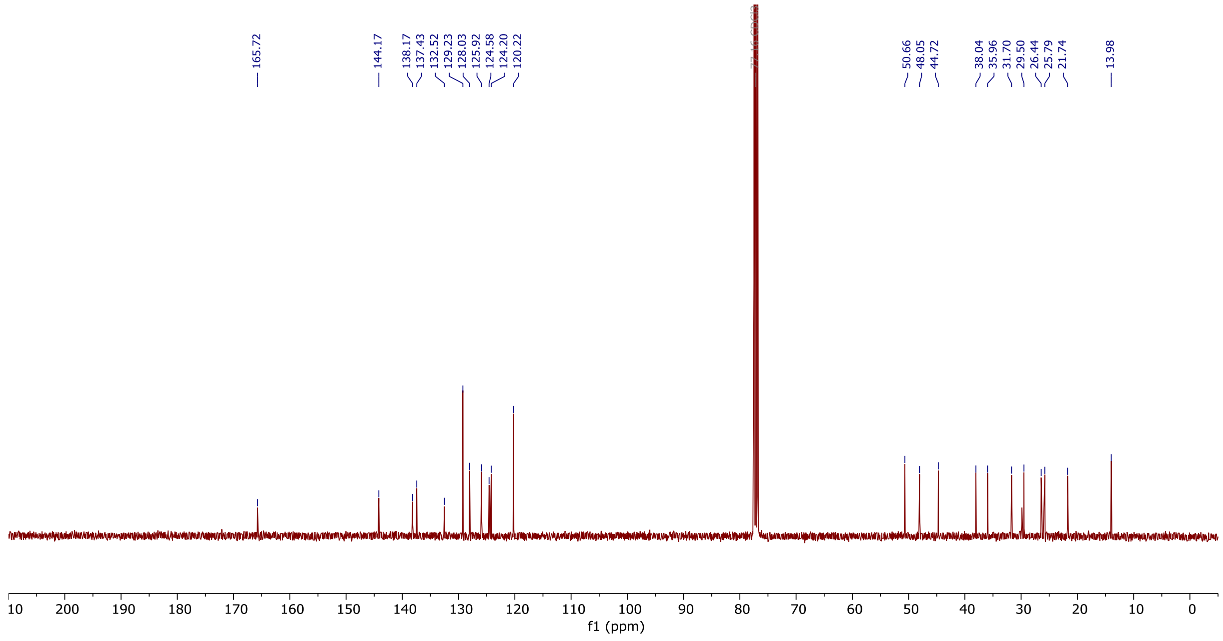

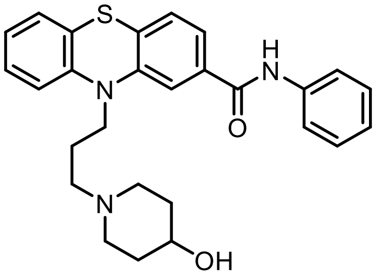

^{1}H NMR spectrum of **37**.

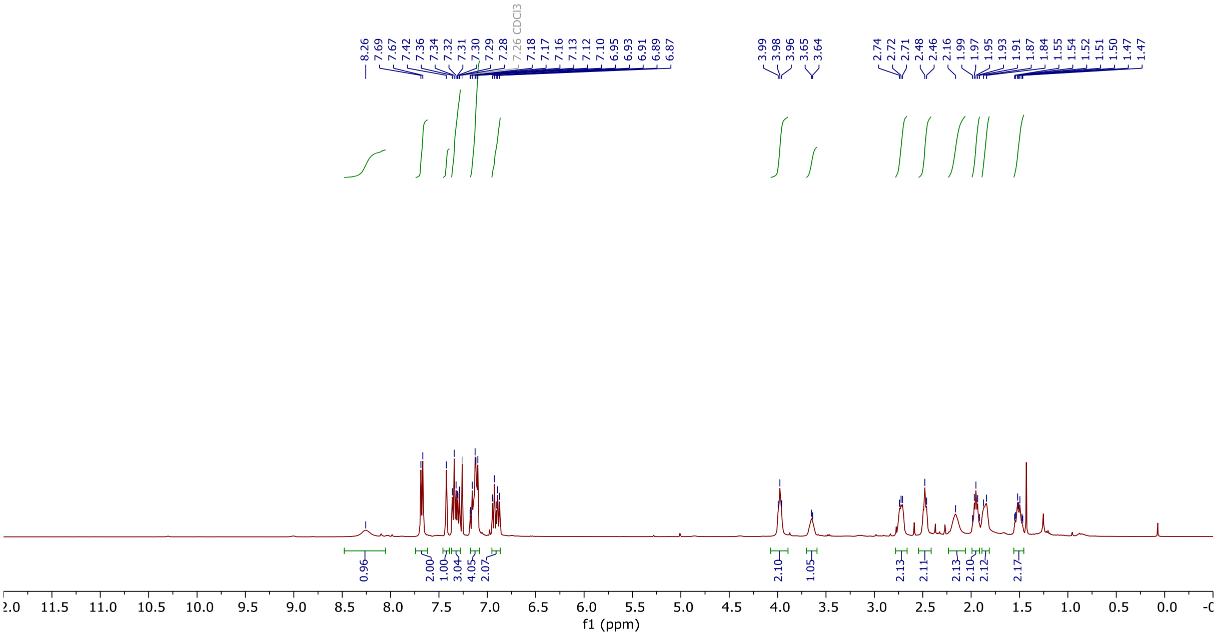

^{13}C NMR spectrum of **37**.

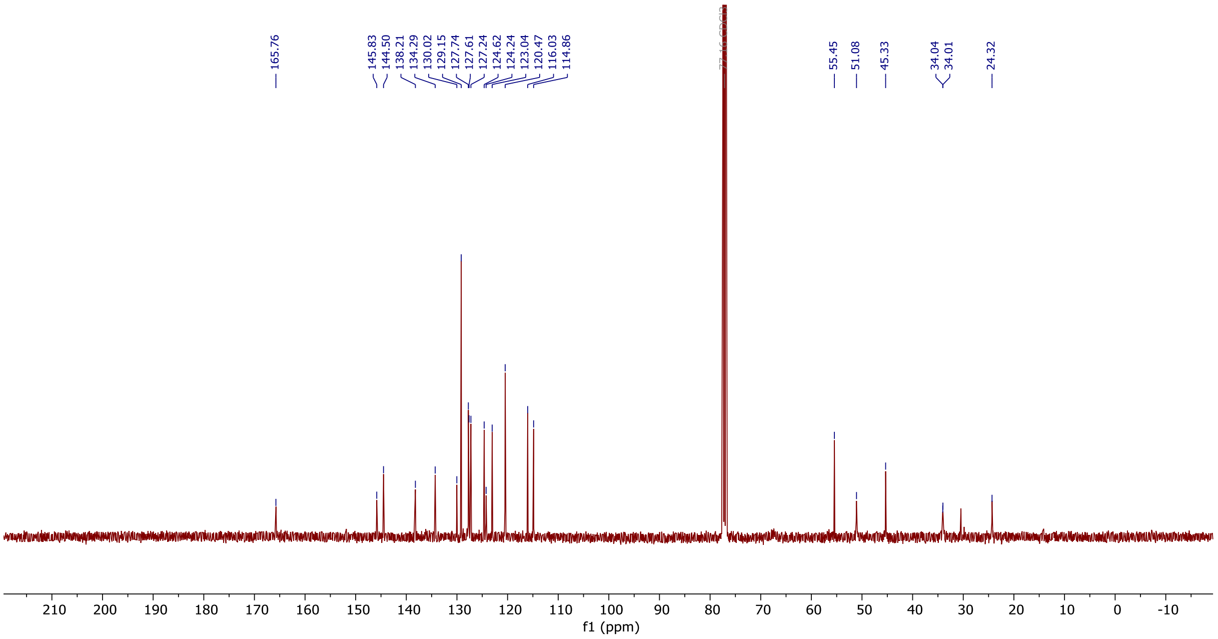

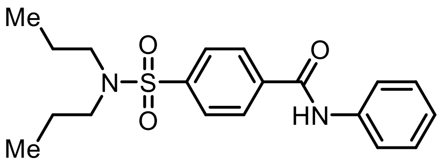

^{1}H NMR spectrum of **38**.

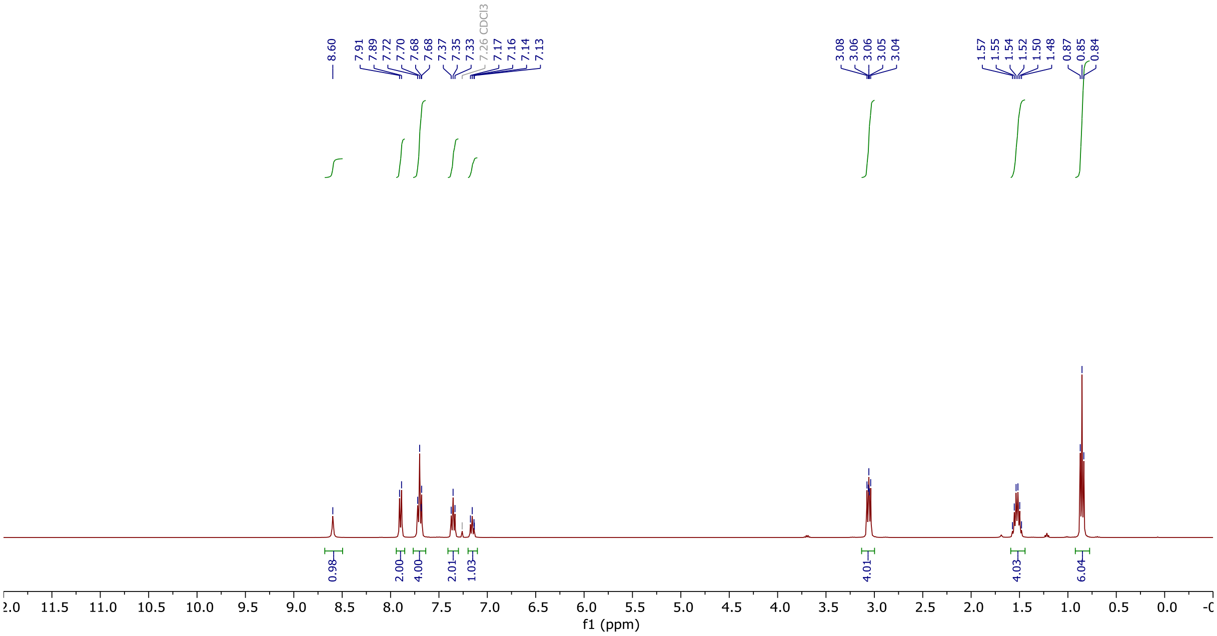

^{13}C NMR spectrum of **38**.

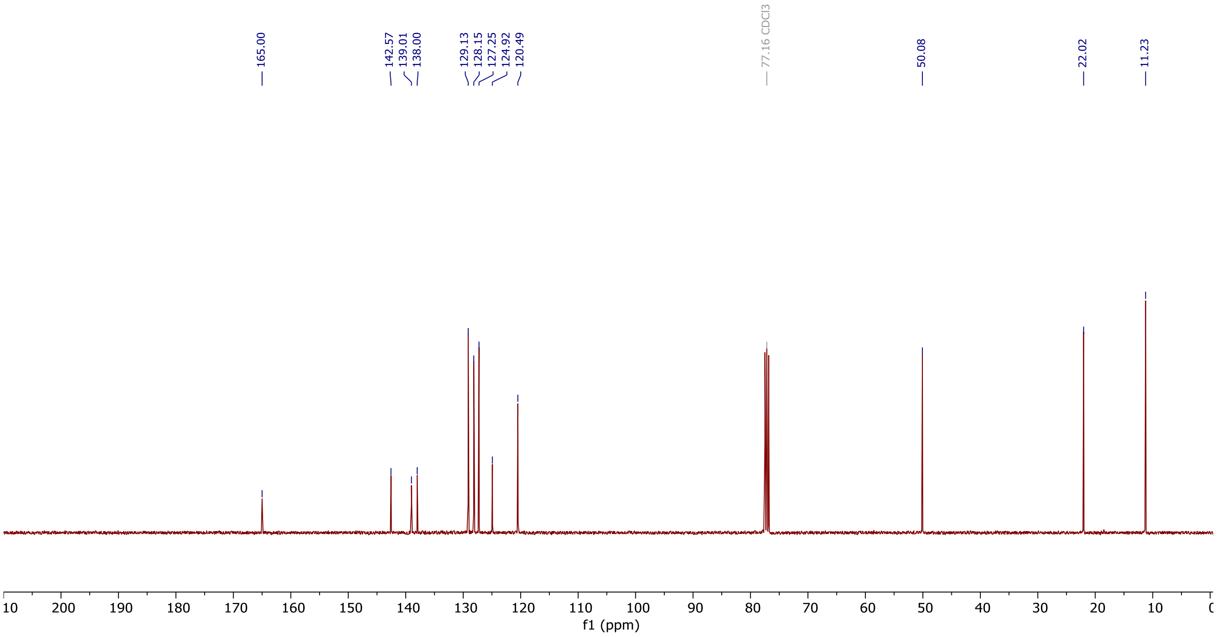

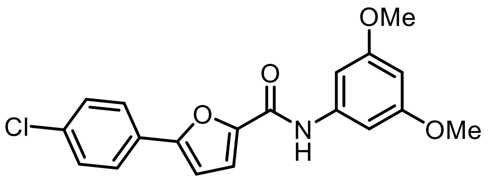

^{1}H NMR spectrum of **39**.

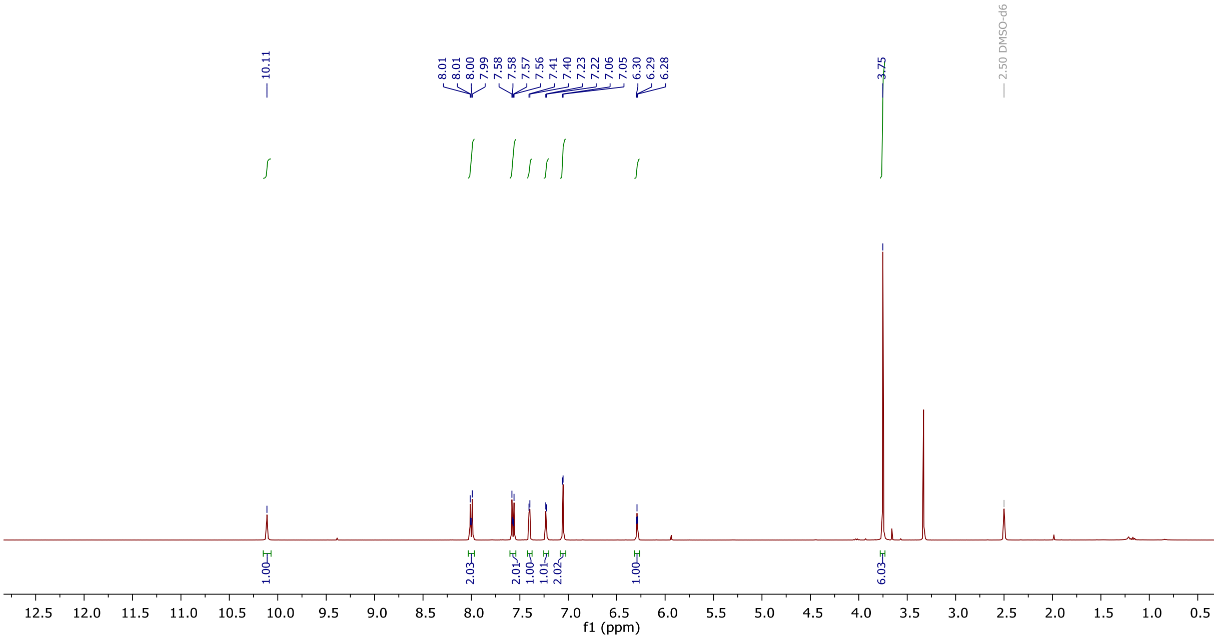

^{13}C NMR spectrum of **39**.

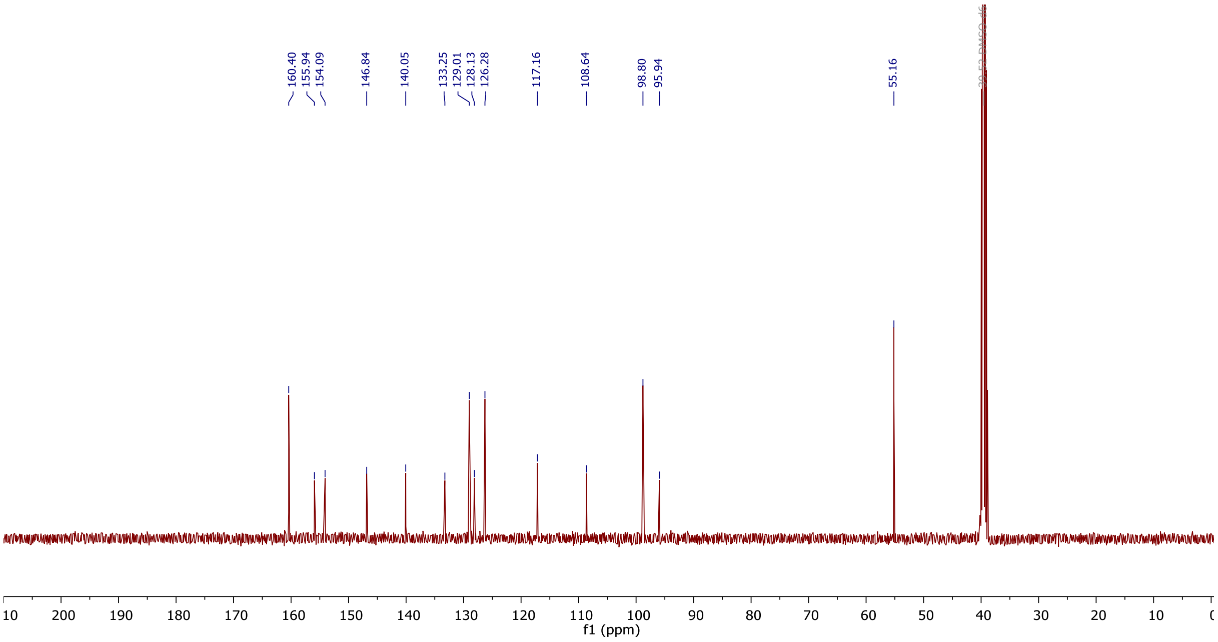

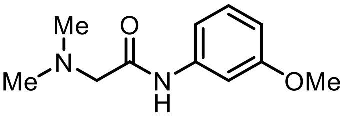

^{1}H NMR spectrum of **42**.

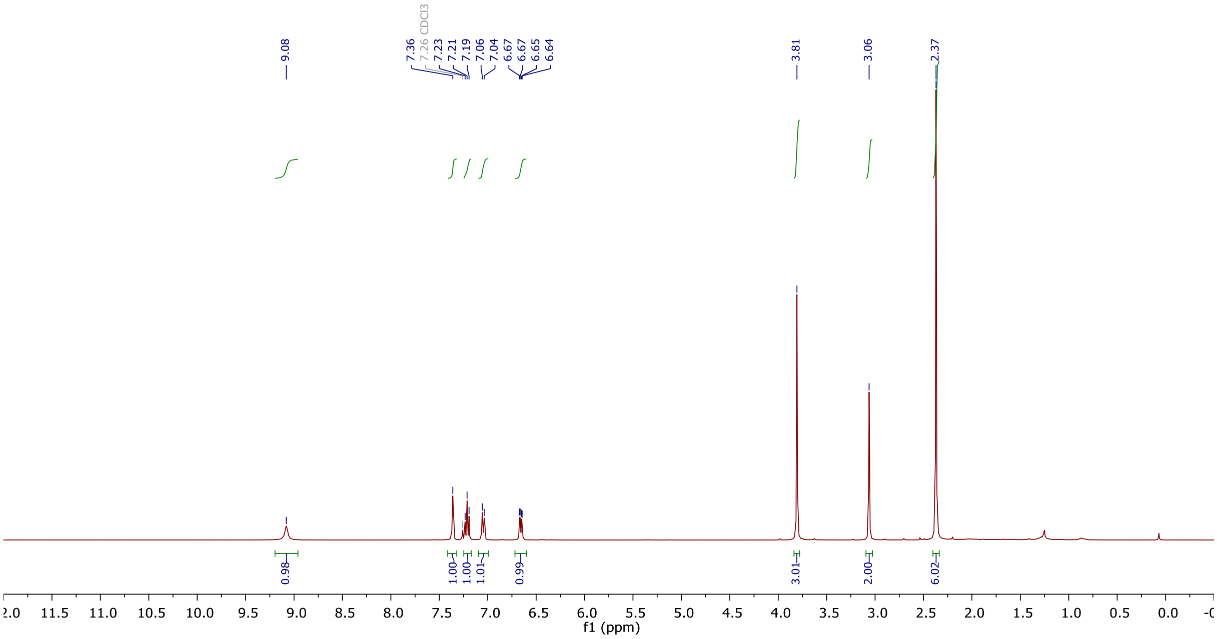

^{13}C NMR spectrum of **42**.

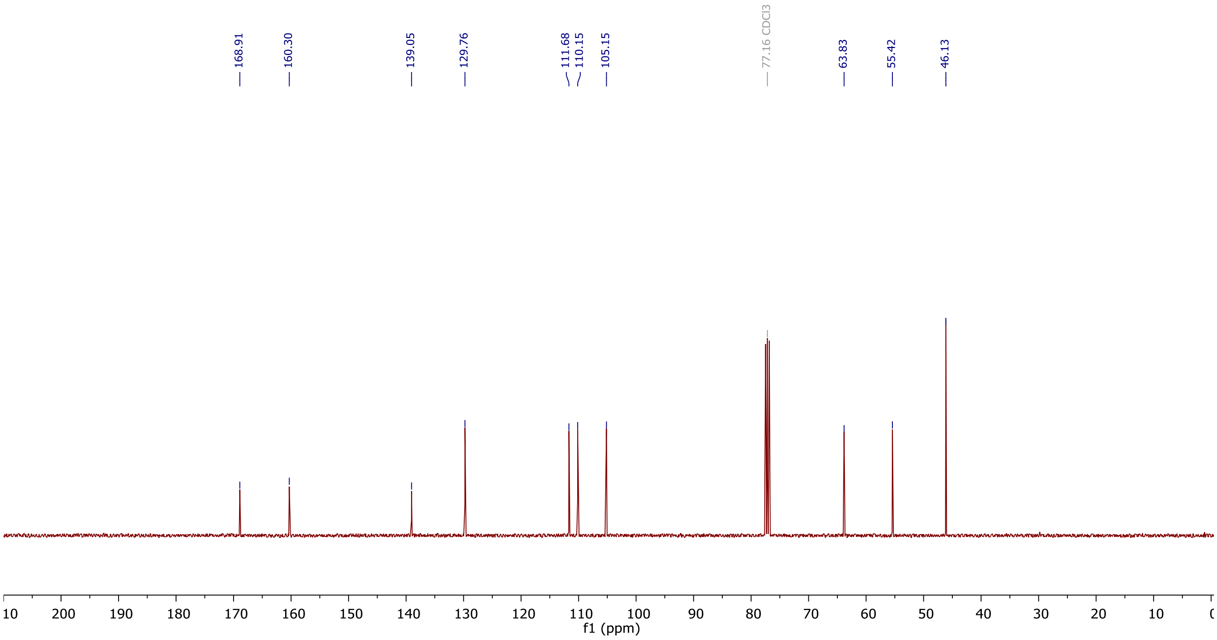

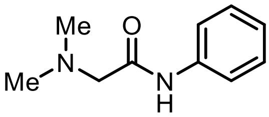

^{1}H NMR spectrum of **43**.

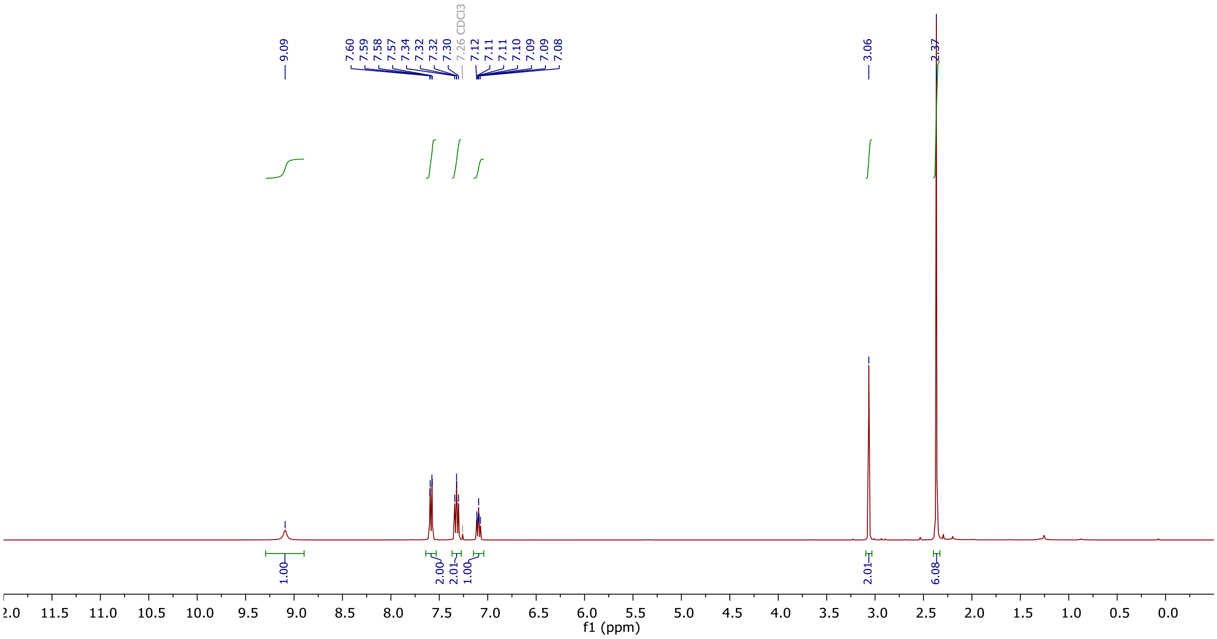

^{13}C NMR spectrum of **43**.

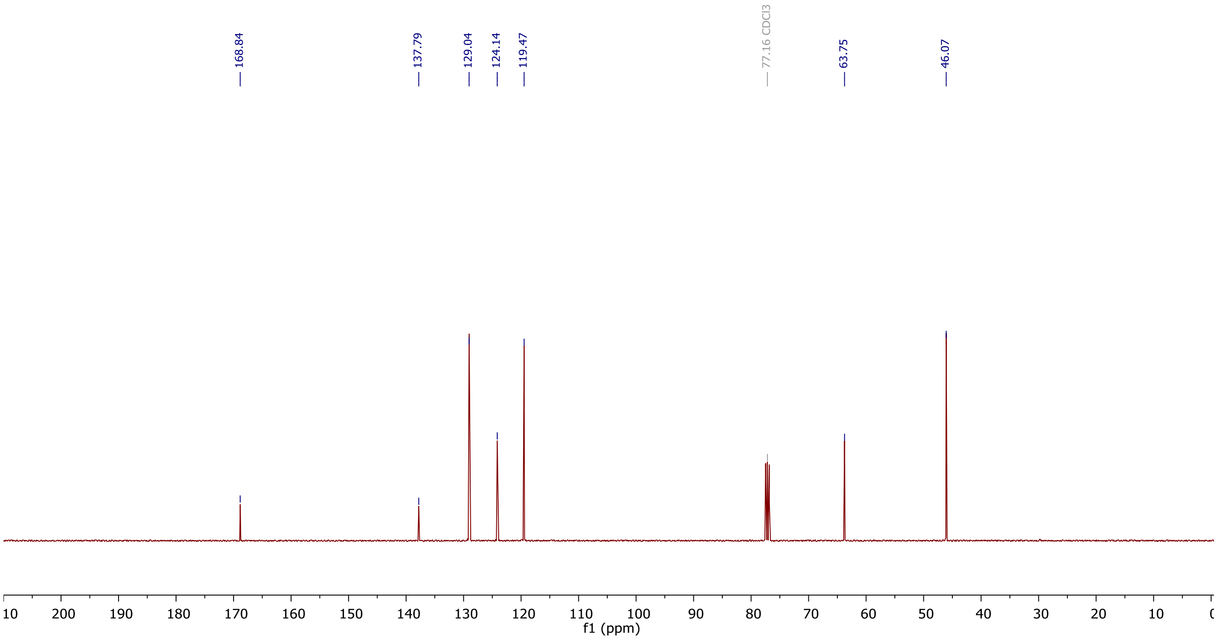

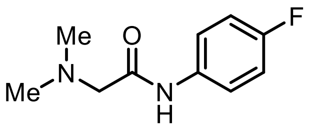

^{1}H NMR spectrum of **44**.

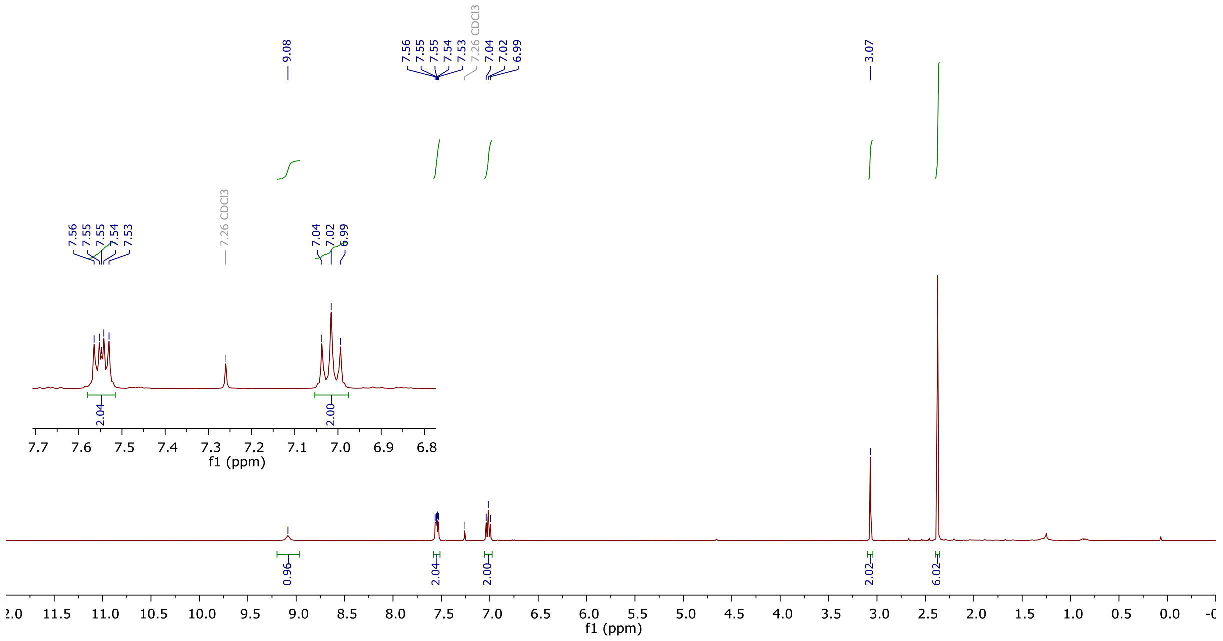

^{13}C NMR spectrum of **44**.

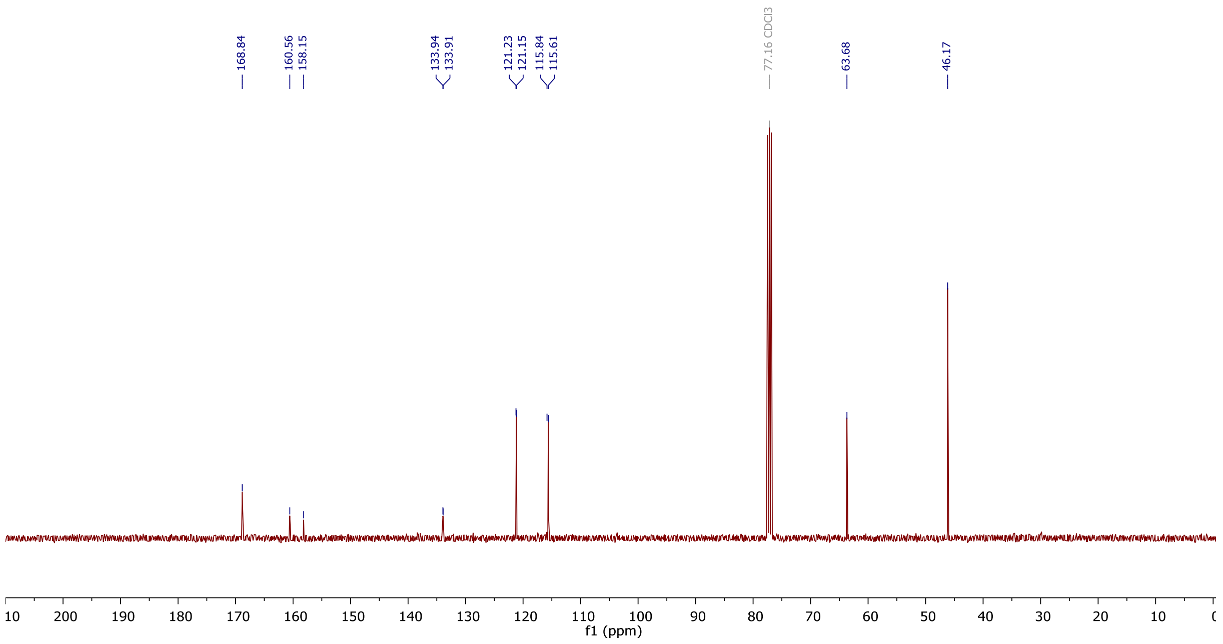

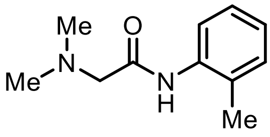

^{1}H NMR spectrum of **45**.

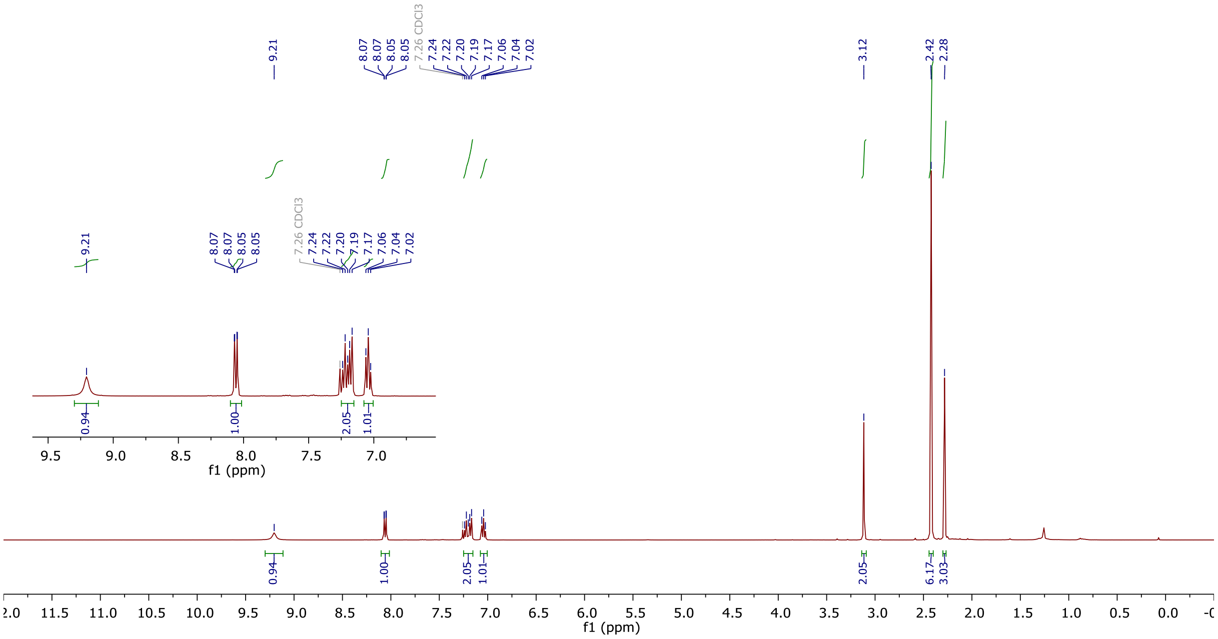

^{13}C NMR spectrum of **45**.

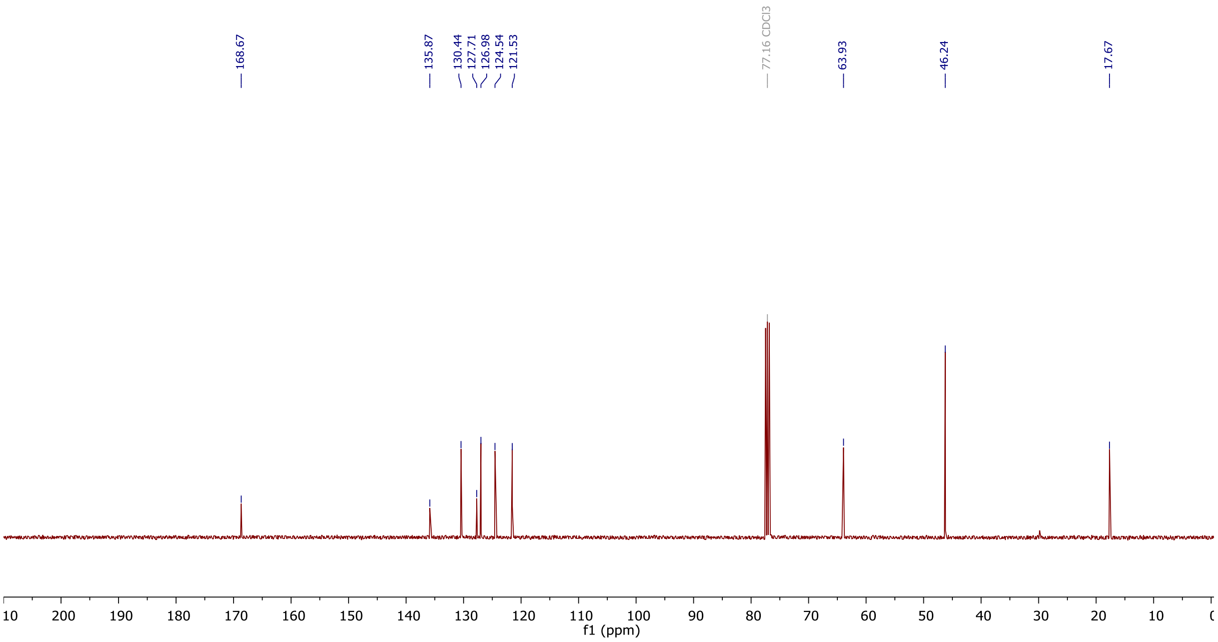

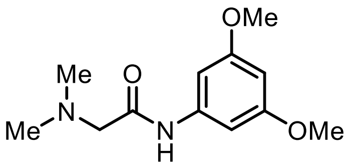

^{1}H NMR spectrum of **46**.

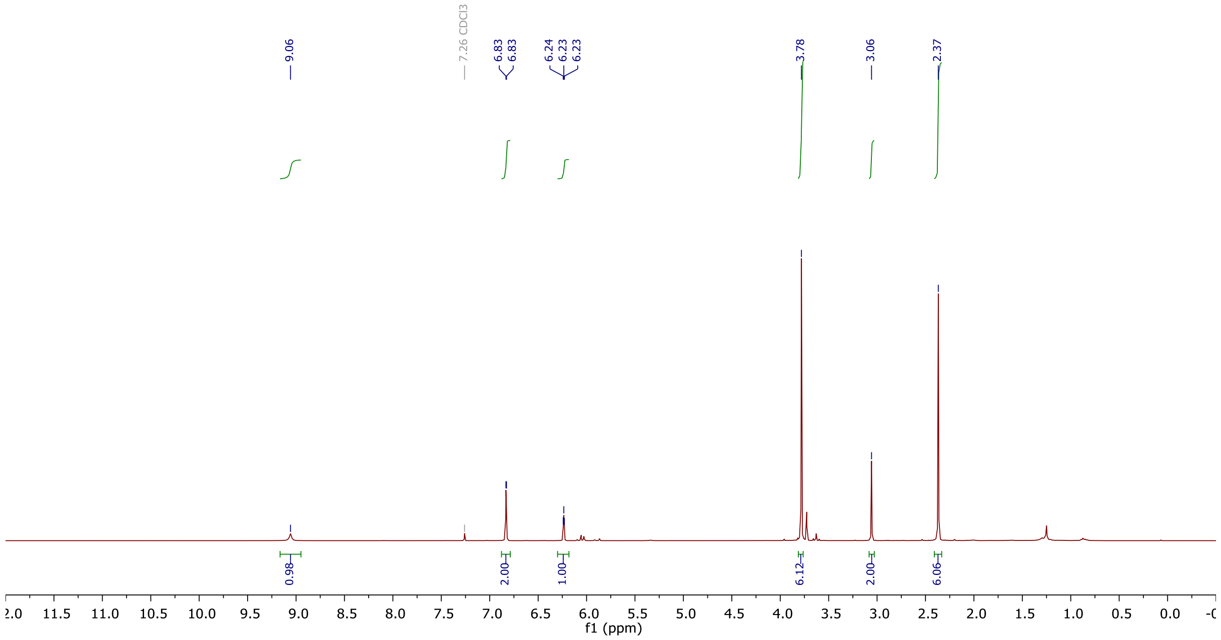

^{13}C NMR spectrum of **46**.

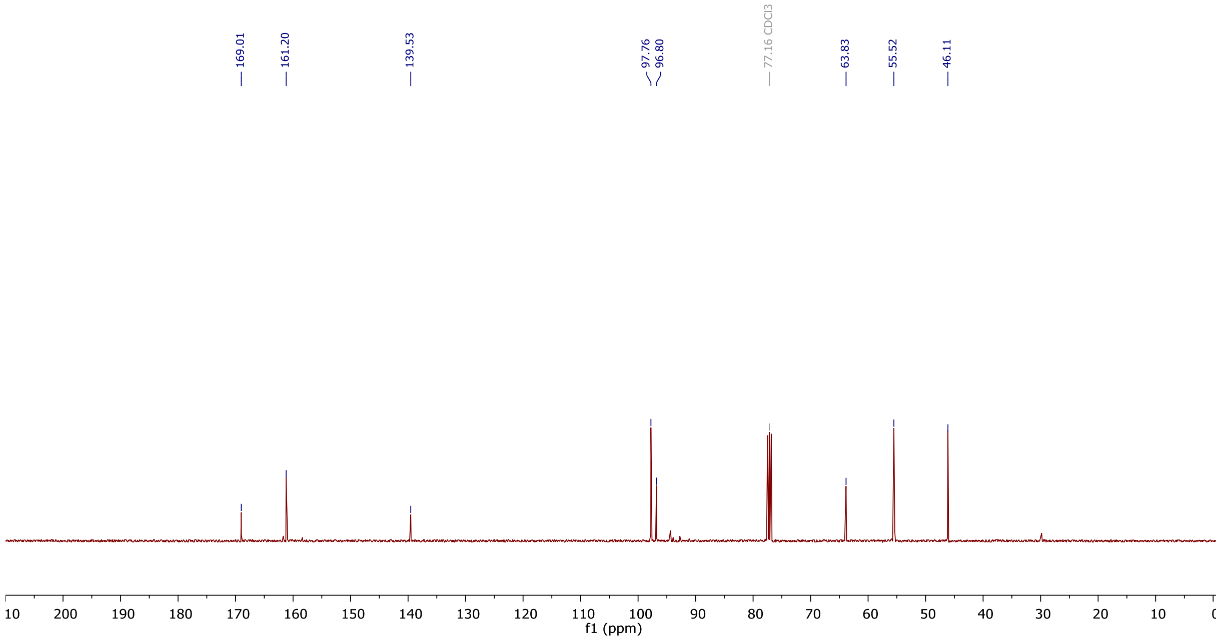

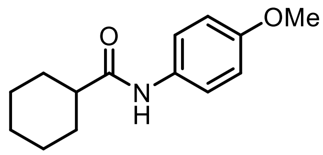

^{1}H NMR spectrum of **47**.

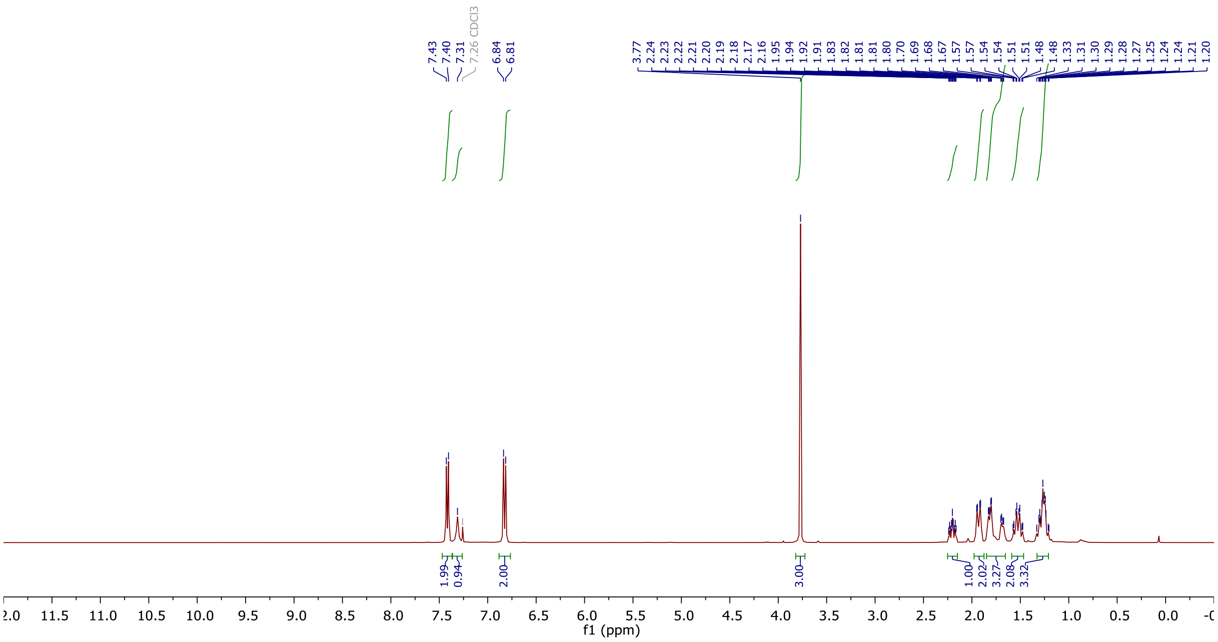

^{13}C NMR spectrum of **47**.

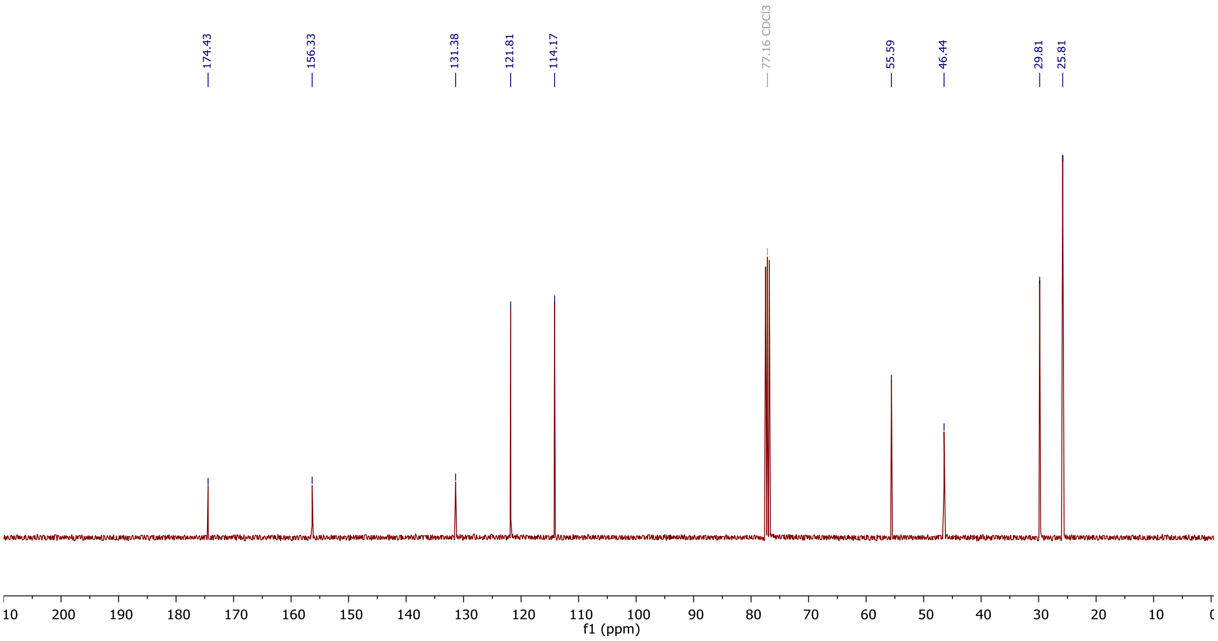

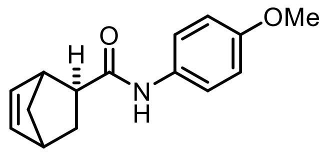

^{1}H NMR spectrum of *exo*-**48**.

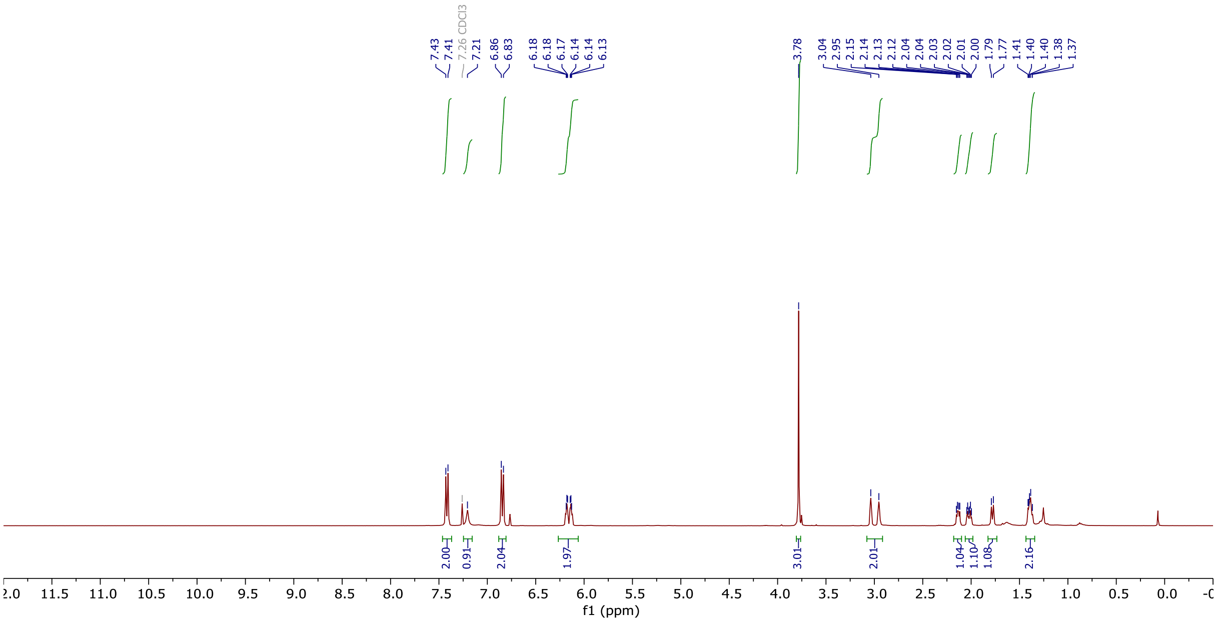

^{13}C NMR spectrum of *exo*-**48**.

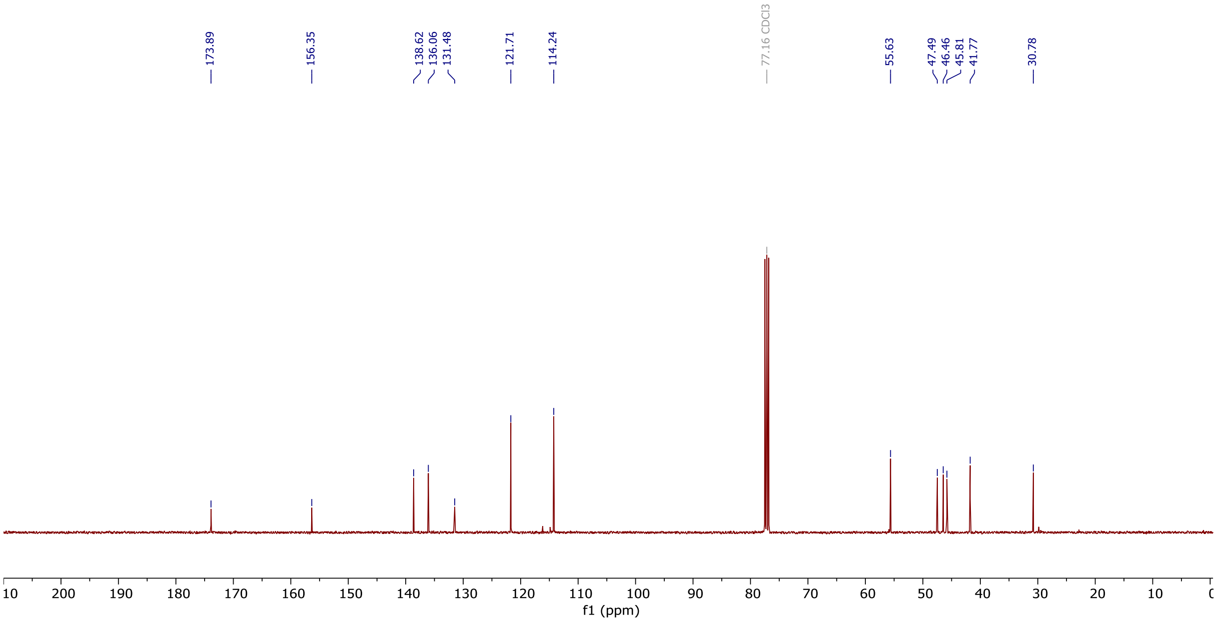

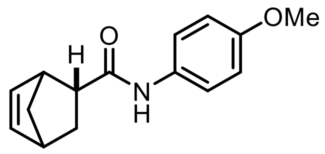

^{1}H NMR spectrum of *endo*-**48**.

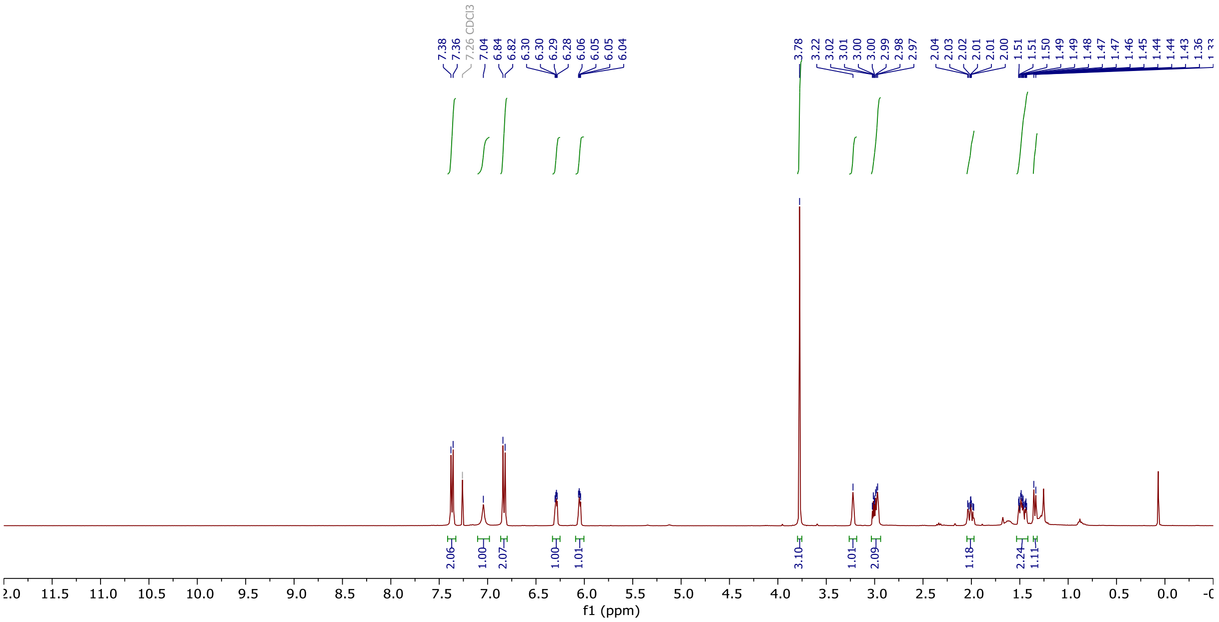

^{13}C NMR spectrum of *endo*-**48**.

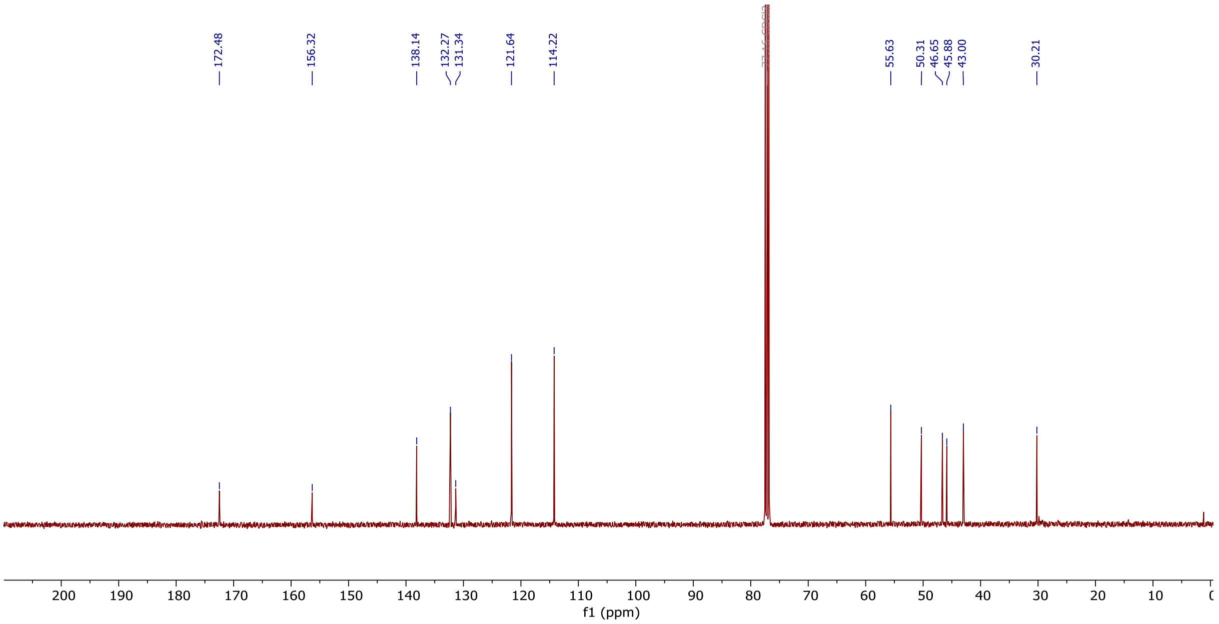

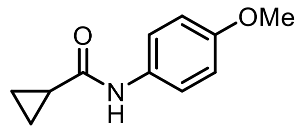

^{1}H NMR spectrum of **49**.

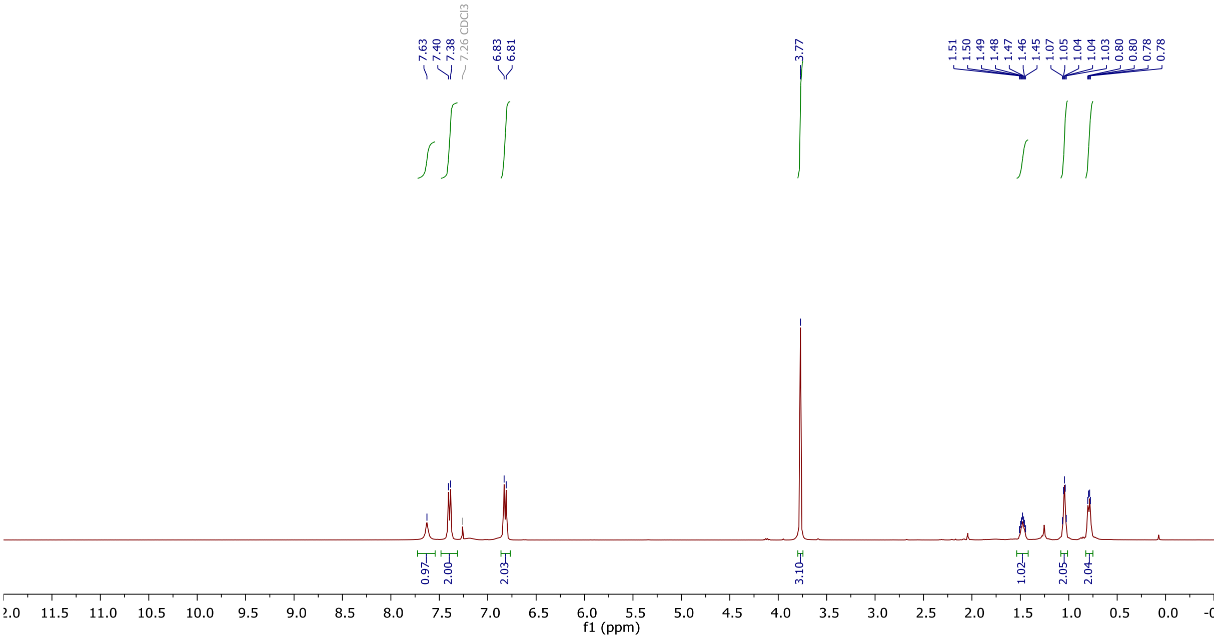

^{13}C NMR spectrum of **49**.

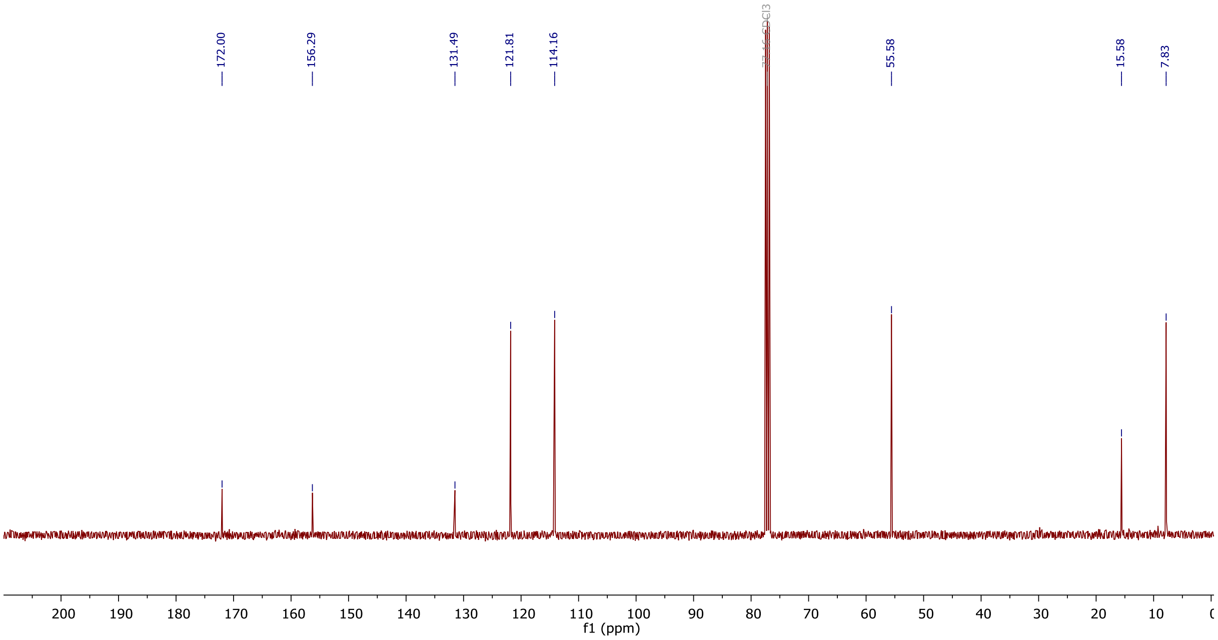

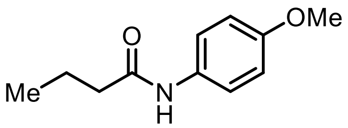

^{1}H NMR spectrum of **50**.

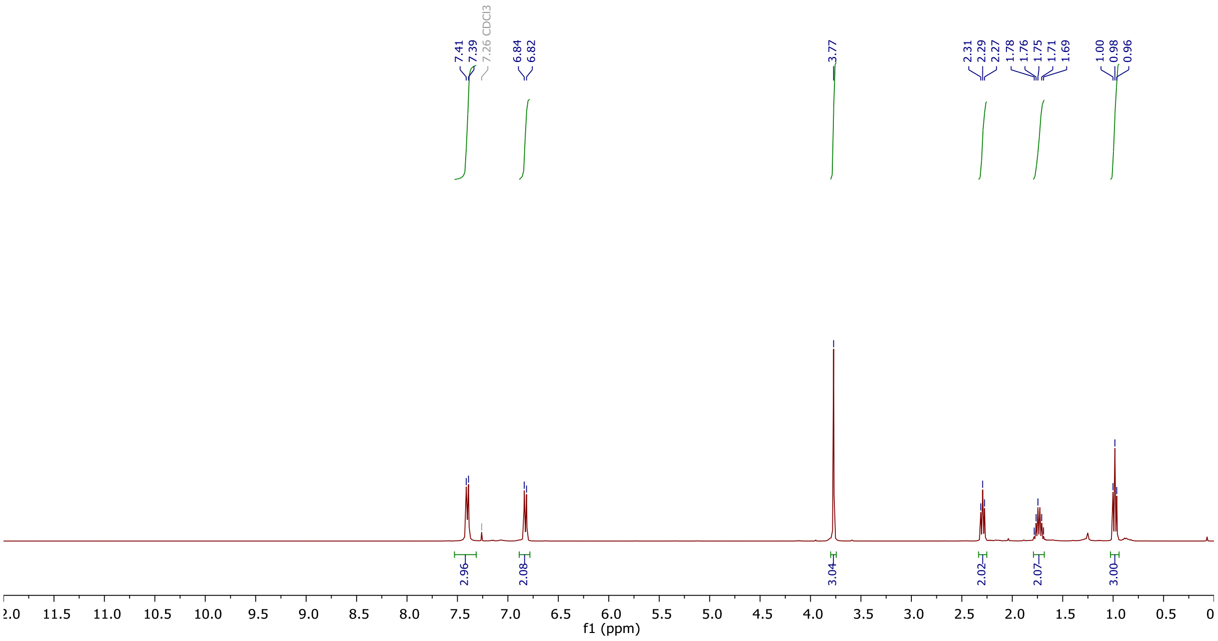

^{13}C NMR spectrum of **50**.

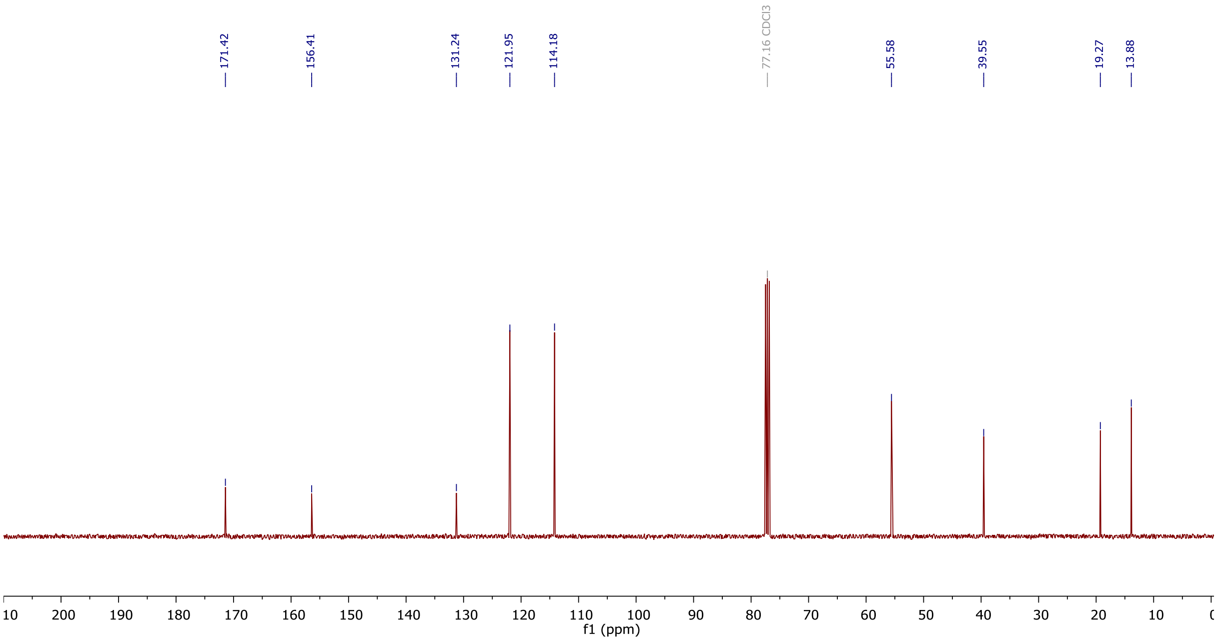

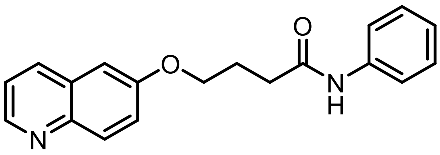

^{1}H NMR spectrum of **51**.

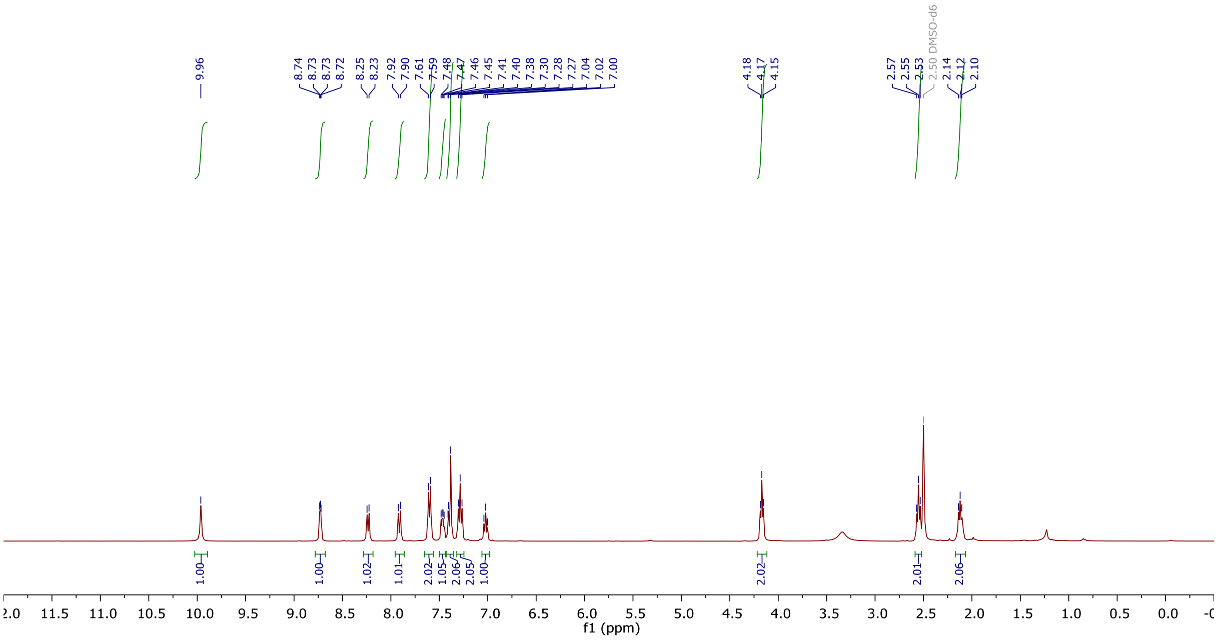

^{13}C NMR spectrum of **51**.

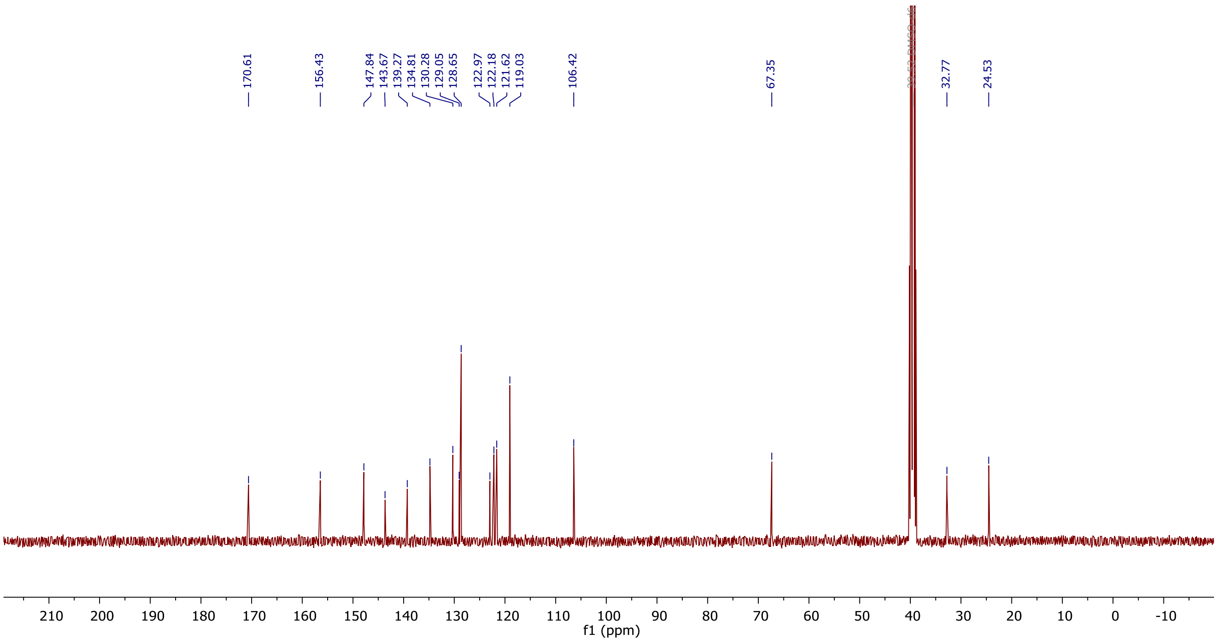

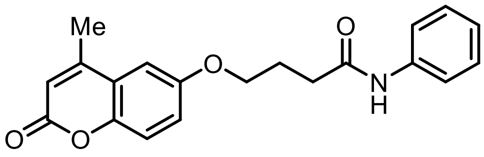

^{1}H NMR spectrum of **52**.

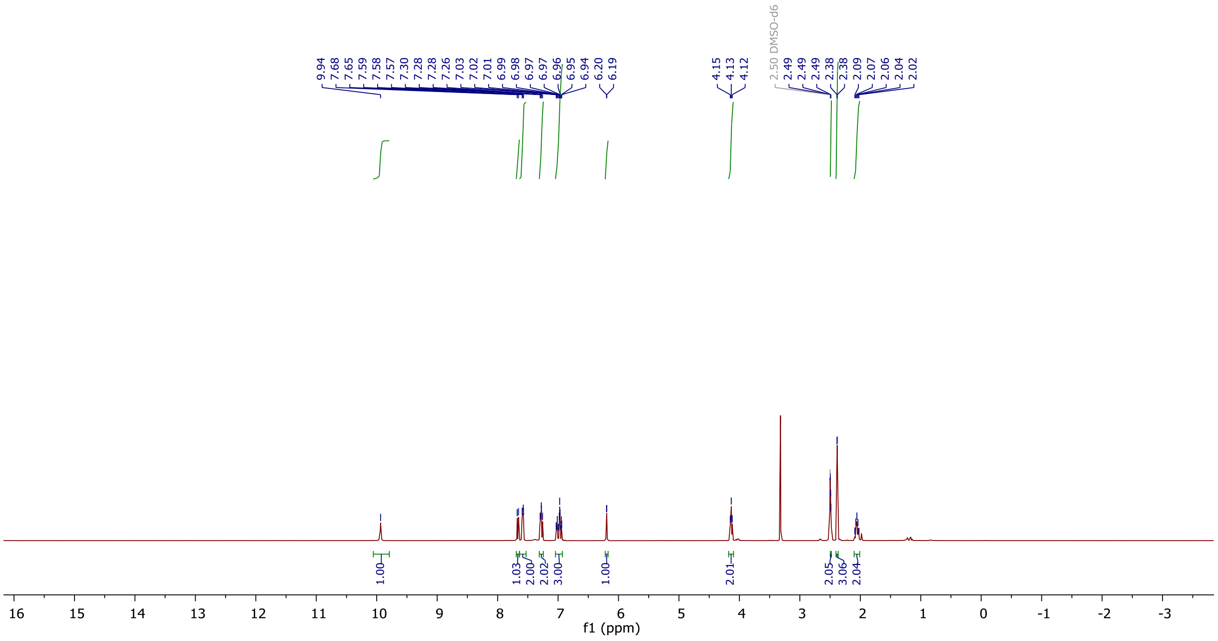

^{13}C NMR spectrum of **52**.

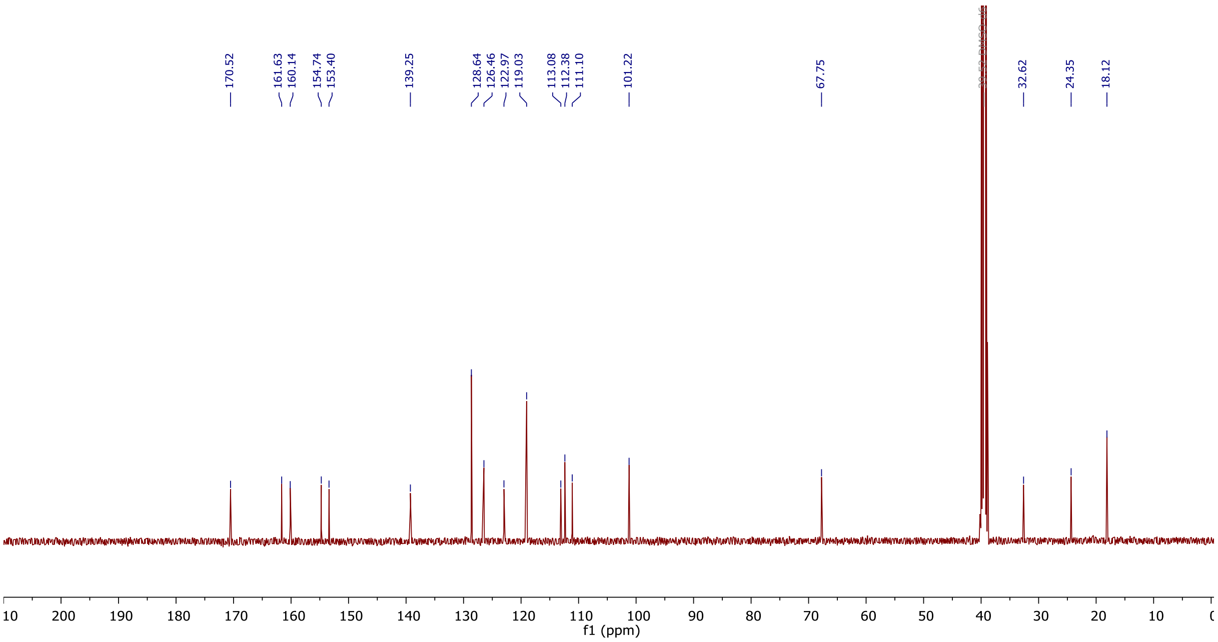

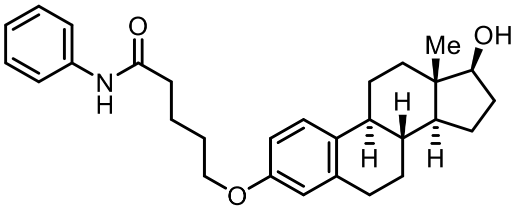

^{1}H NMR spectrum of **53**.

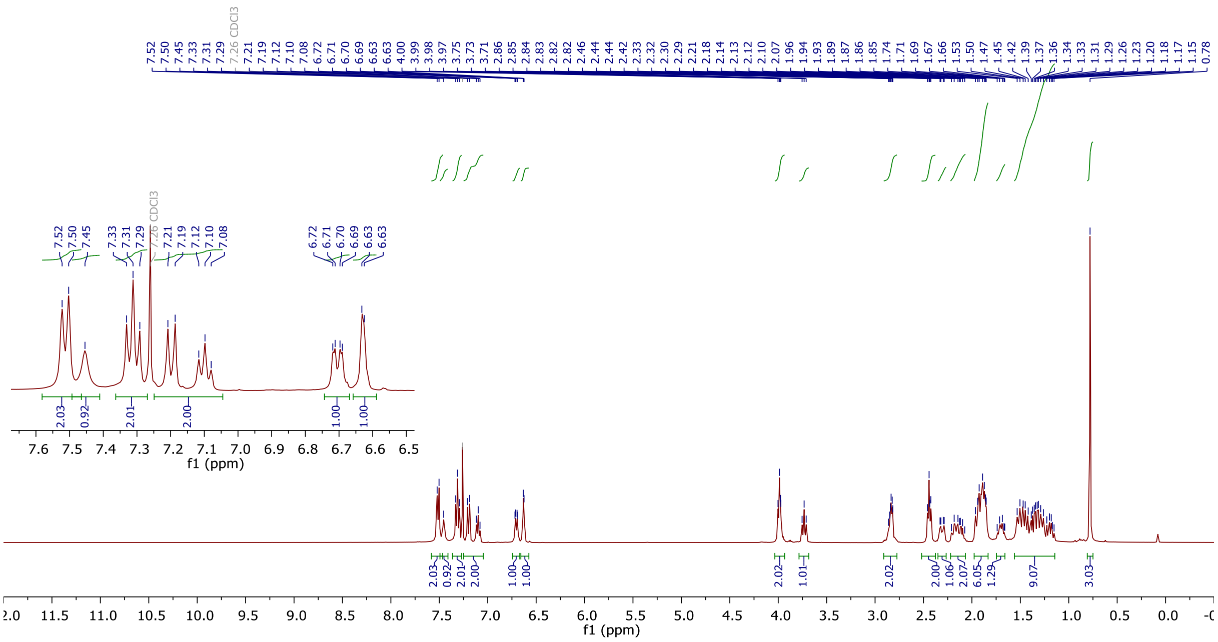

^{13}C NMR spectrum of **53**.

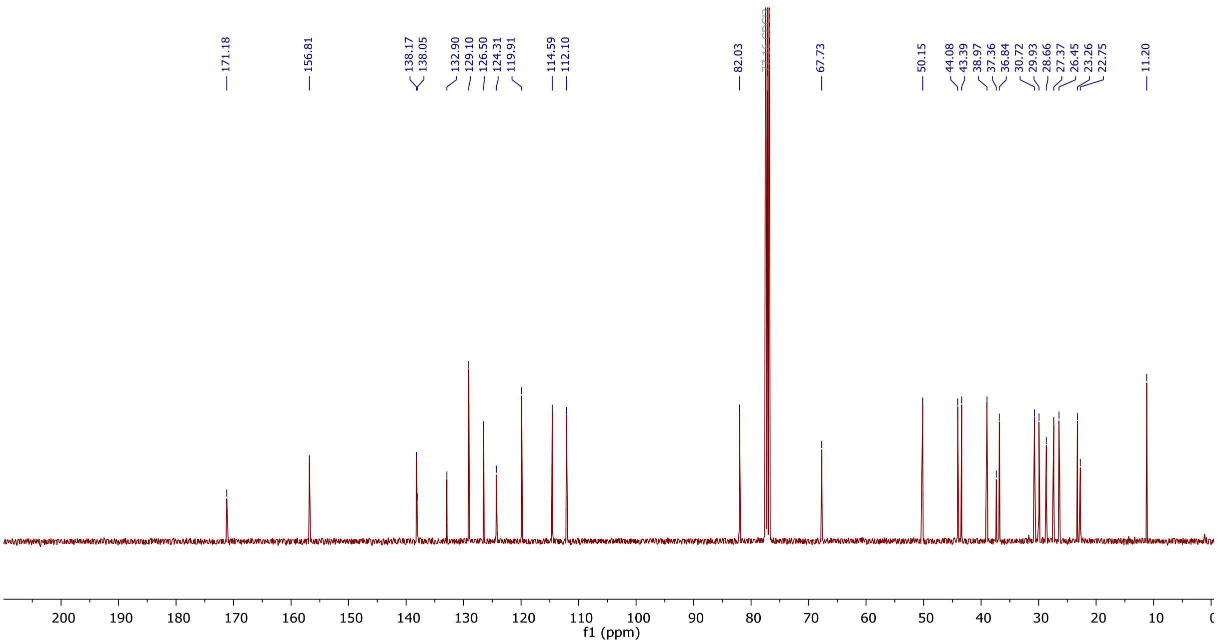

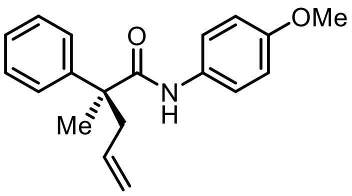

^{1}H NMR spectrum of **56** from the integrated synthesis.

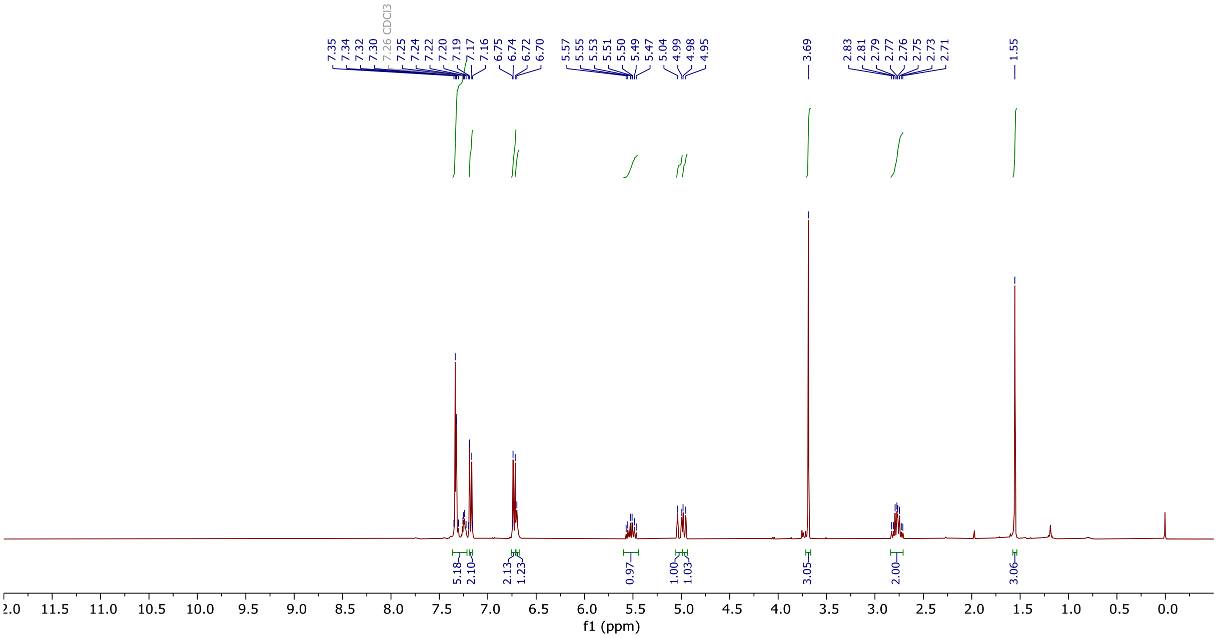

^{13}C NMR spectrum of **56** from the integrated synthesis.

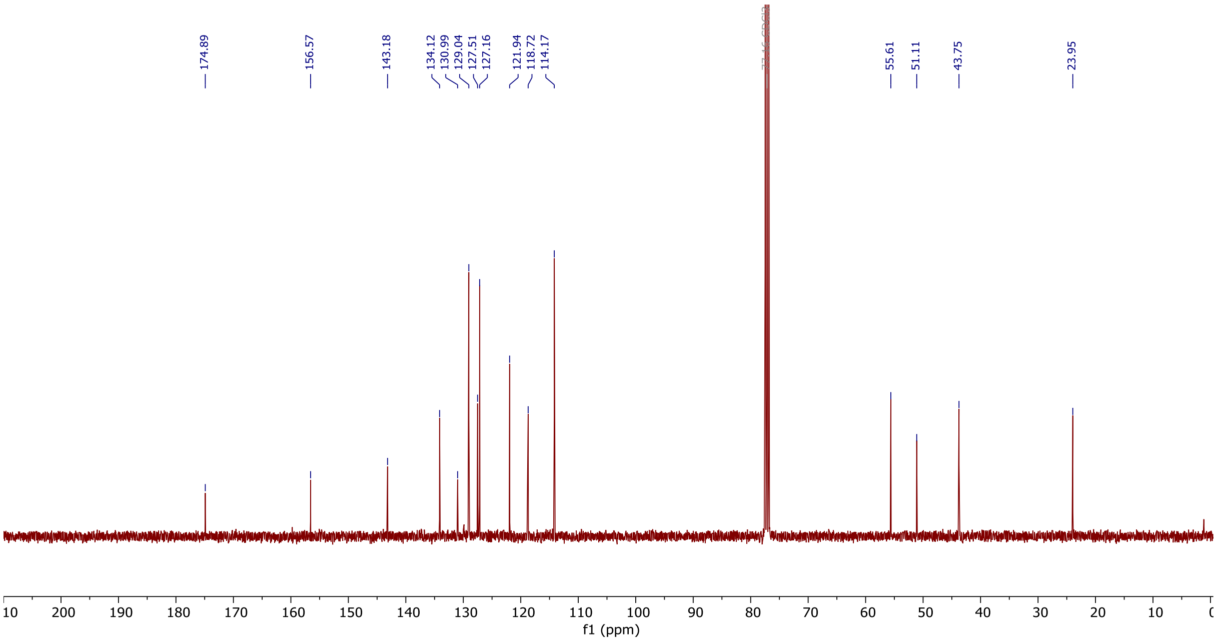

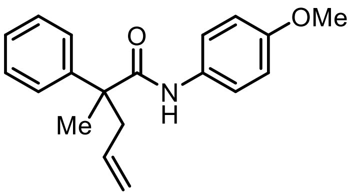

^{1}H NMR spectrum of rac-**56** from standard synthesis.

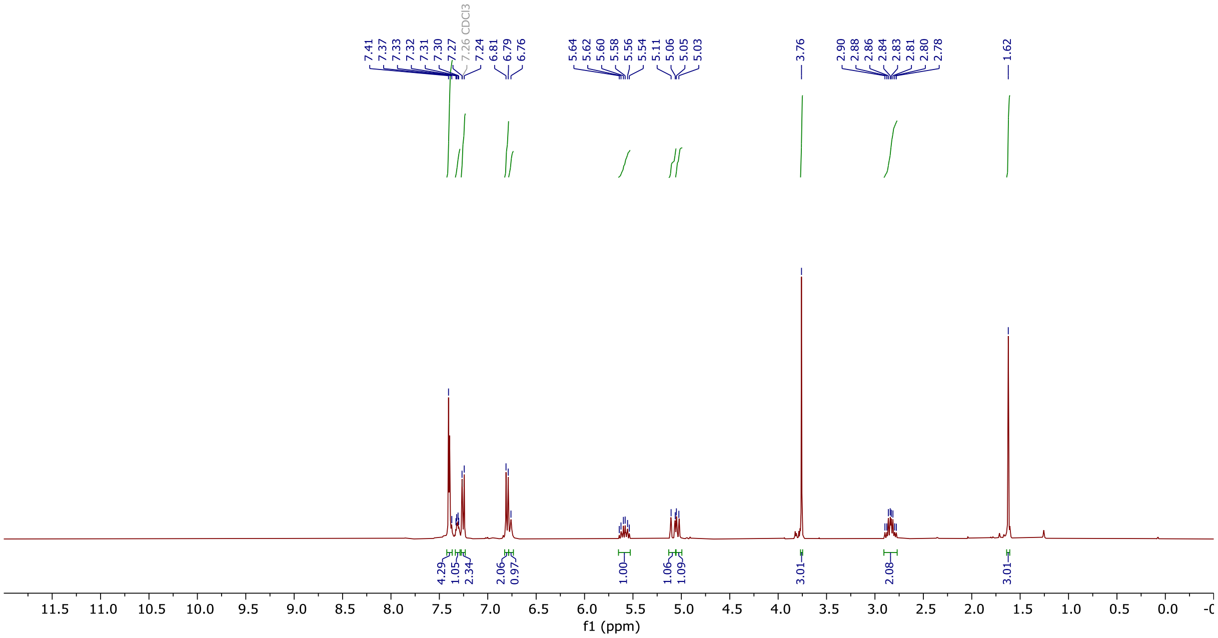

^{13}C NMR spectrum of rac-**56** from standard synthesis.

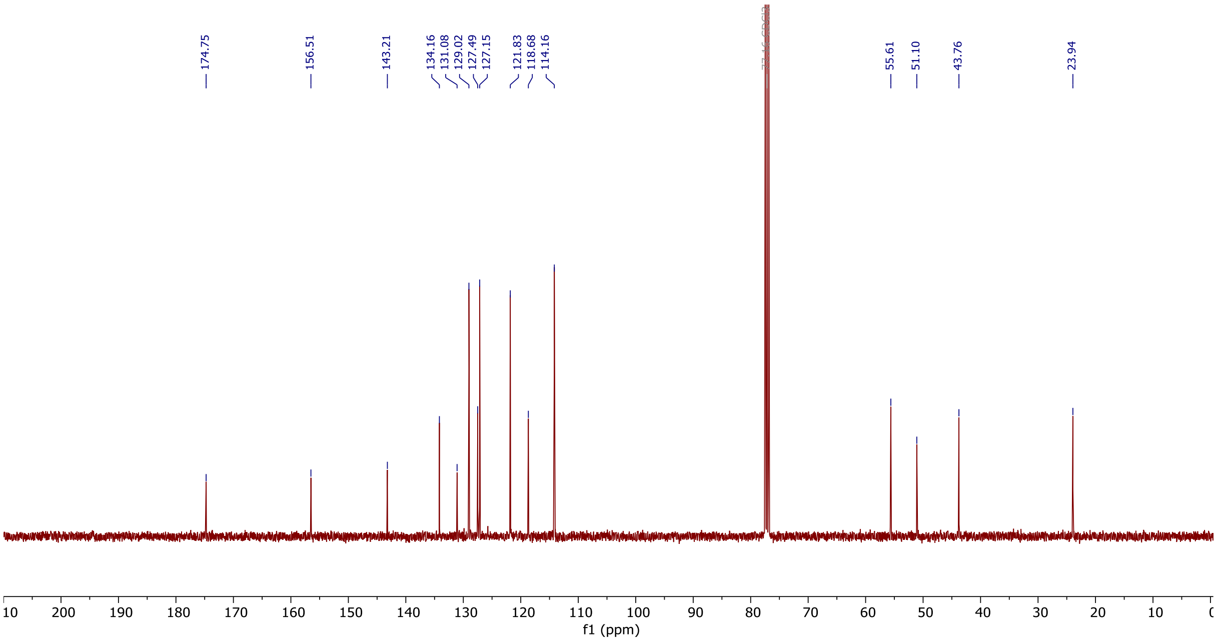

Chiral HPLC trace for rac-56:

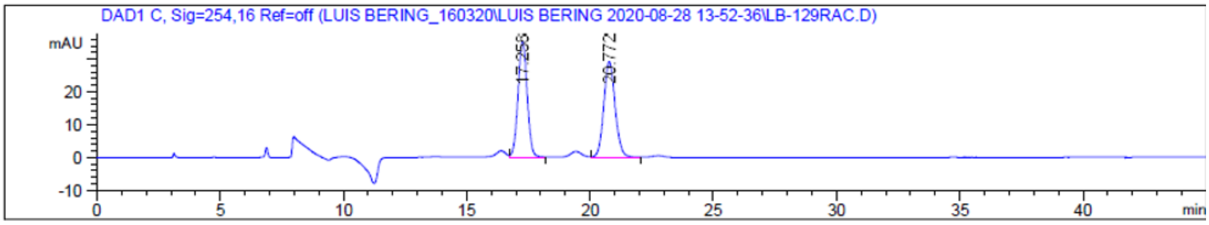


```
=====================================================================
                         Area Percent Report
=====================================================================

Sorted By             :      Signal
Multiplier            :      1.0000
Dilution              :      1.0000
Use Multiplier & Dilution Factor with ISTDs


Signal 1: DAD1 C, Sig=254,16 Ref=off

Peak RetTime Type  Width     Area      Height     Area
  #   [min]        [min]   [mAU*s]     [mAU]       %
----|-------|----|-------|----------|----------|--------|
   1  17.258 VB    0.3975  904.58020   35.23432  49.4514
   2  20.772 VB    0.4932  924.65070   29.06288  50.5486
```

Chiral HPLC trace for 56.

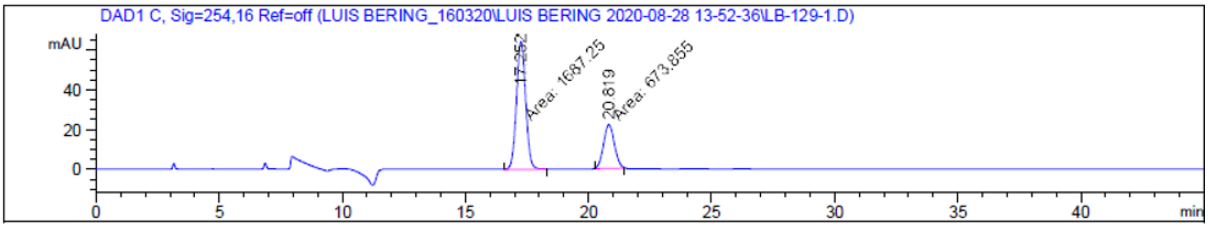


```
=====================================================================
                         Area Percent Report
=====================================================================

Sorted By             :      Signal
Multiplier            :      1.0000
Dilution              :      1.0000
Use Multiplier & Dilution Factor with ISTDs


Signal 1: DAD1 C, Sig=254,16 Ref=off

Peak RetTime Type  Width     Area      Height     Area
  #   [min]        [min]   [mAU*s]     [mAU]       %
----|-------|----|-------|----------|----------|--------|
   1  17.252 MM    0.4360 1687.24573   64.49347  71.4601
   2  20.819 MM    0.5110  673.85455   21.97999  28.5399
```

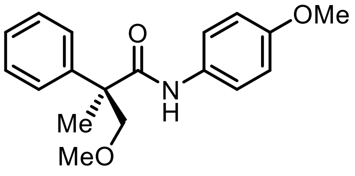

^{1}H NMR spectrum of **57** from the integrated synthesis.

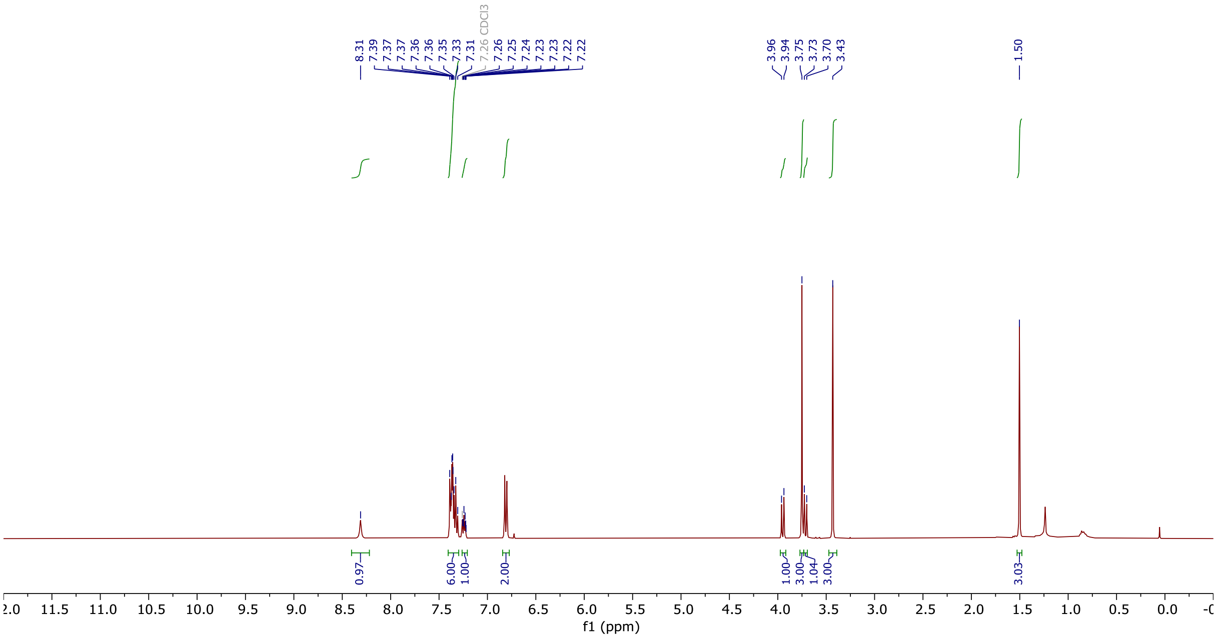

^{13}C NMR spectrum of **57** from the integrated synthesis.

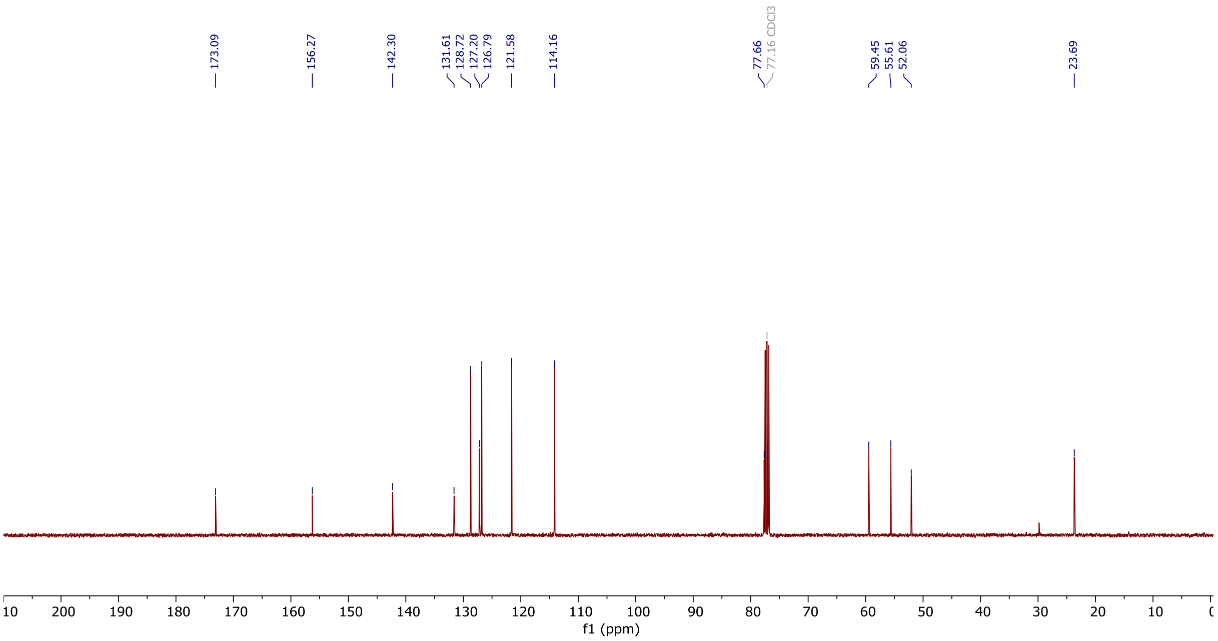

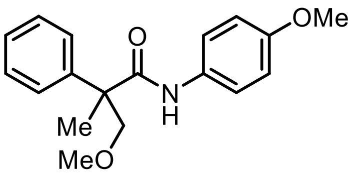

^{1}H NMR spectrum of rac-**57** from standard synthesis.

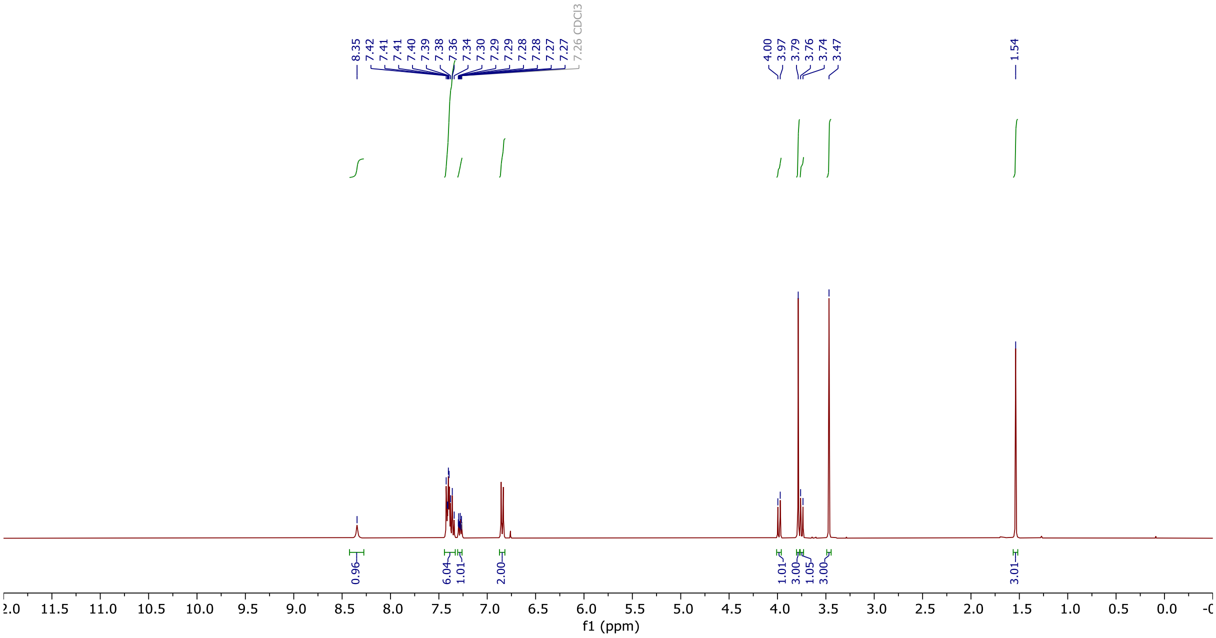

^{13}C NMR spectrum of rac-**57** from the integrated synthesis.

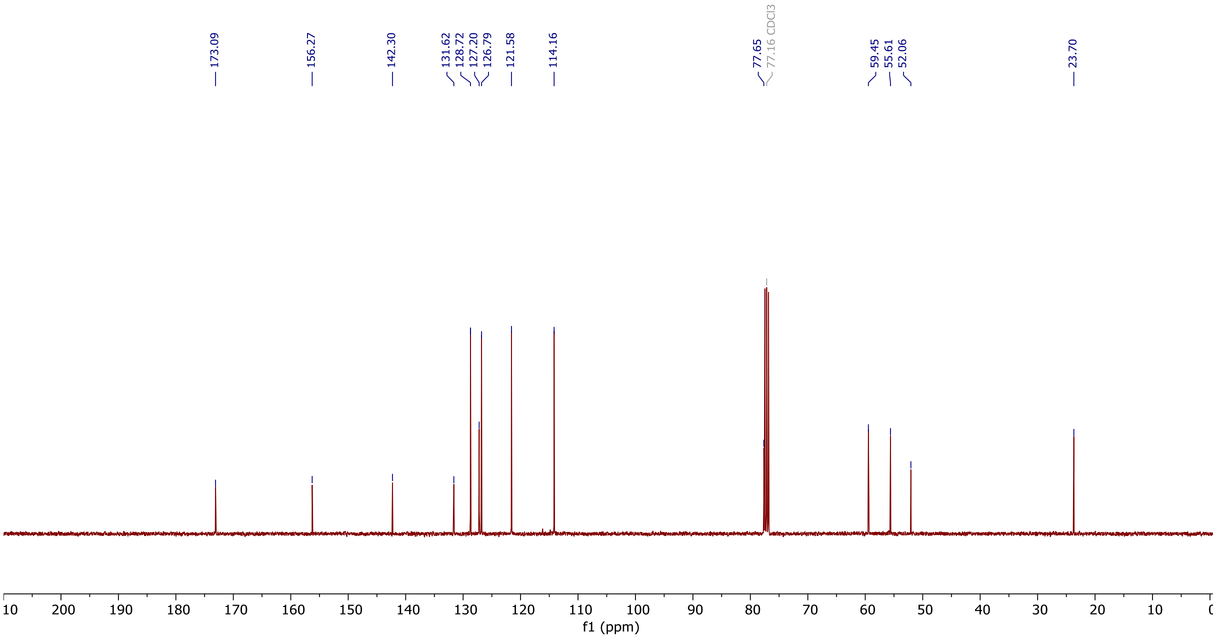

Chiral HPLC trace for rac-57:

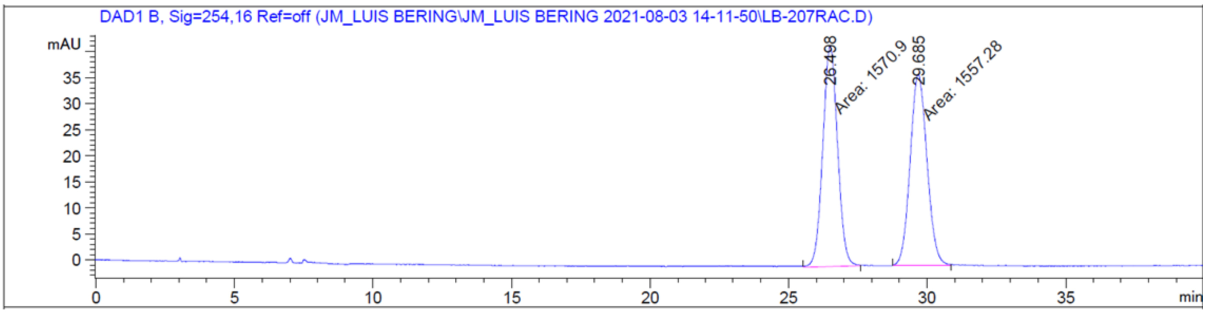

Signal 2: DAD1 B, Sig=254,16 Ref=off

Peak #	RetTime [min]	Type	Width [min]	Area [mAU*s]	Height [mAU]	Area %
1	26.498	MM	0.6194	1570.90417	42.27025	50.2177
2	29.685	MM	0.7068	1557.28271	36.72050	49.7823
Totals :				3128.18689	78.99075	

Chiral HPLC trace for 57.

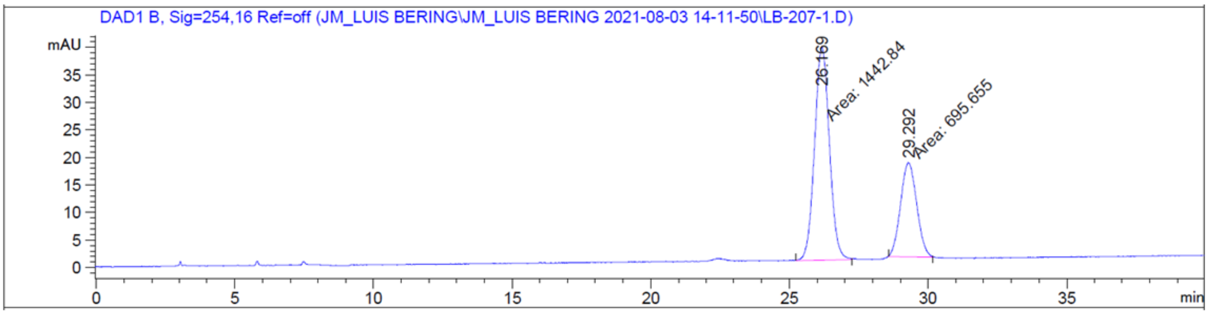

Signal 2: DAD1 B, Sig=254,16 Ref=off

Peak #	RetTime [min]	Type	Width [min]	Area [mAU*s]	Height [mAU]	Area %
1	26.169	MM	0.6197	1442.84216	38.80214	67.4699
2	29.292	MM	0.6766	695.65460	17.13480	32.5301
Totals :				2138.49677	55.93694	

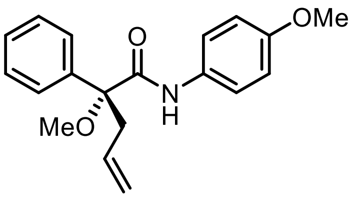

^{1}H NMR spectrum of **58** from the integrated synthesis.

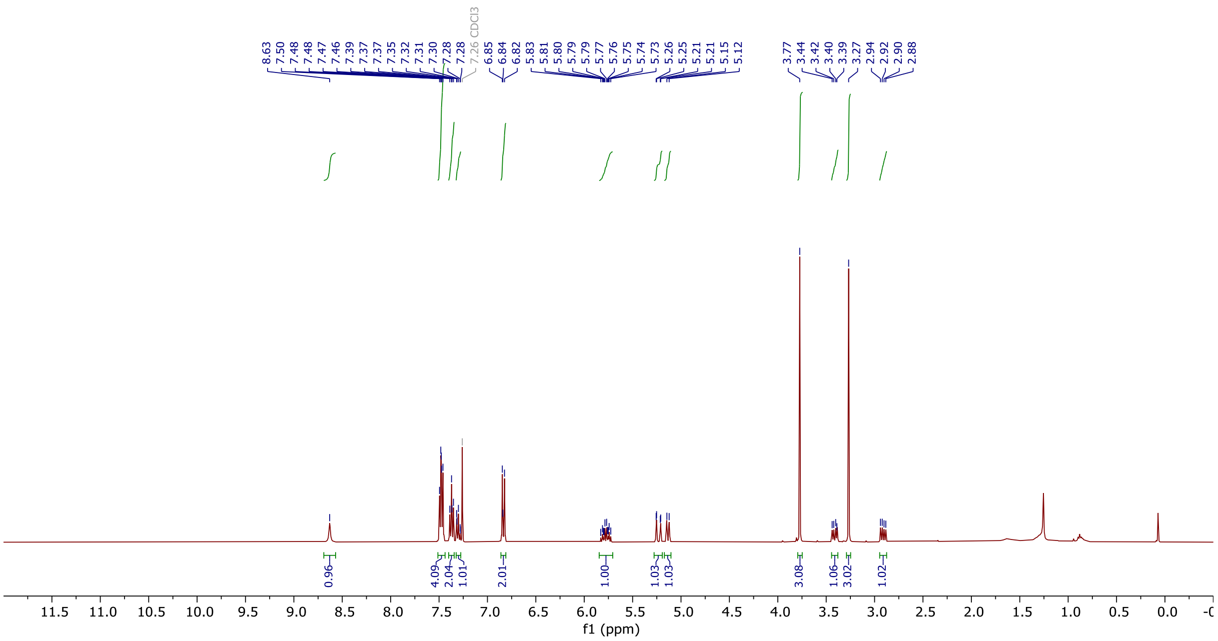

^{13}C NMR spectrum of **58** from the integrated synthesis.

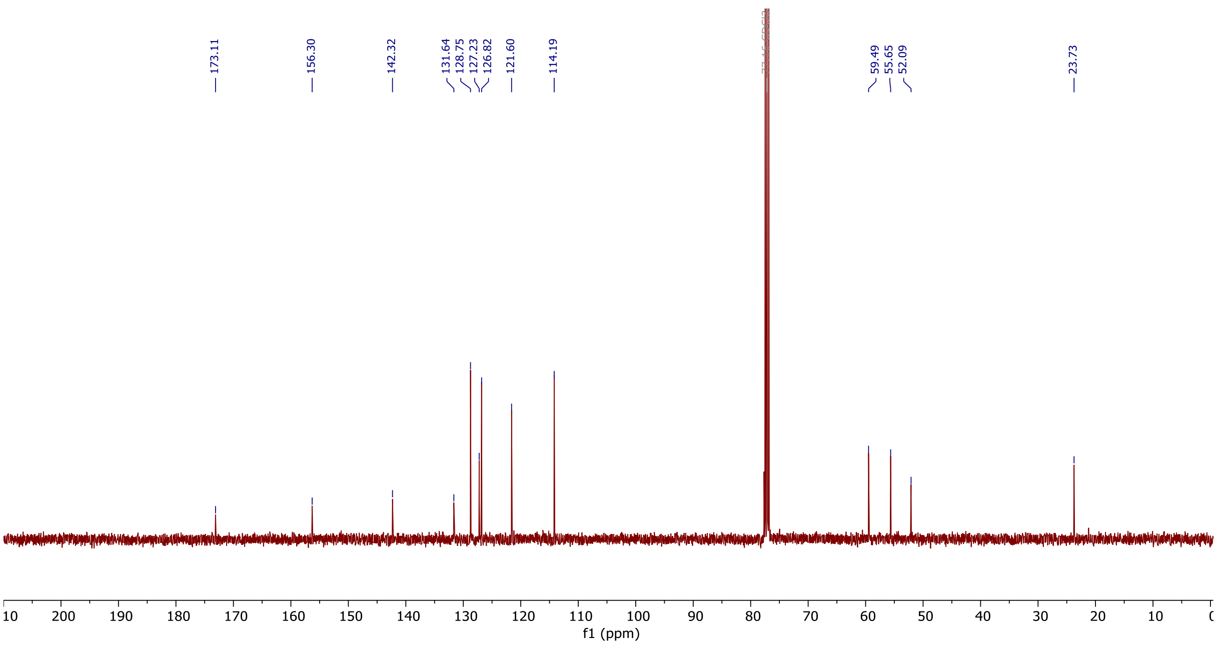

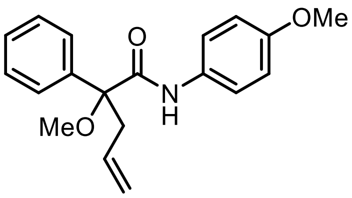

^{1}H NMR spectrum of rac-**58** from standard synthesis.

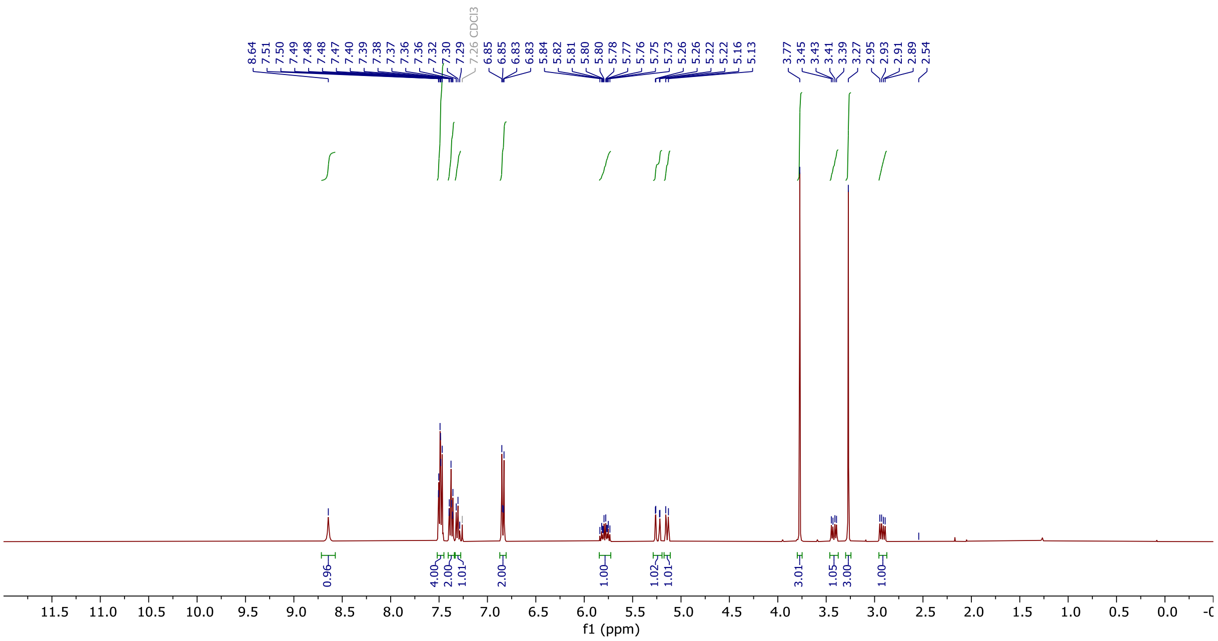

^{13}C NMR spectrum of rac-**58** from the integrated synthesis.

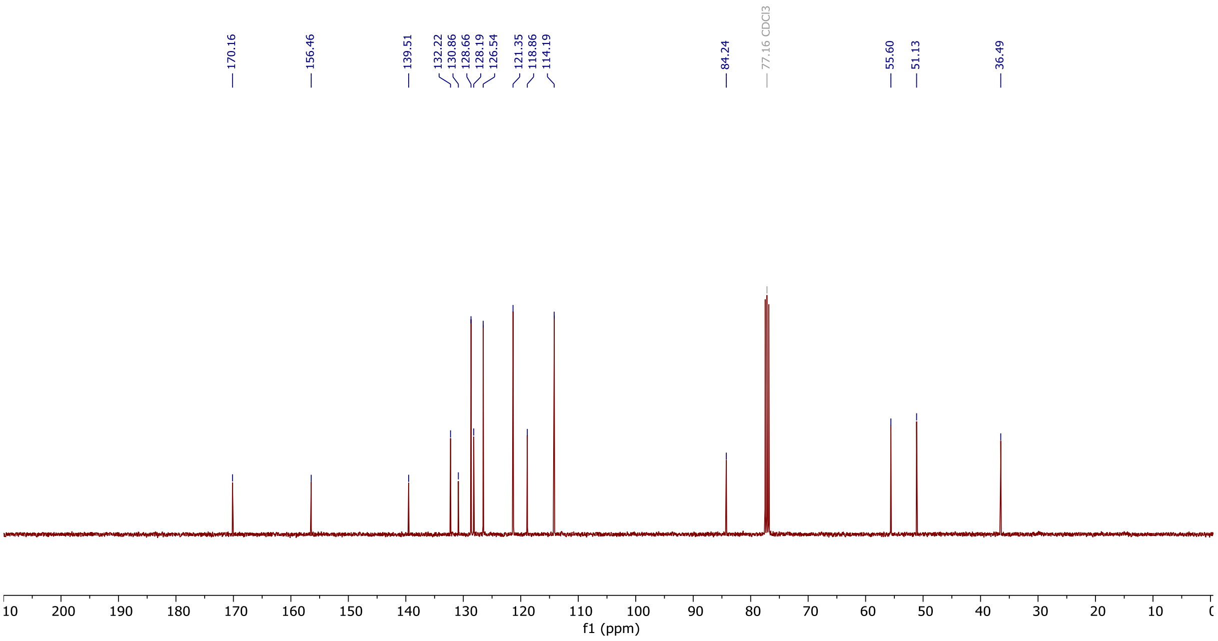

Chiral HPLC trace for rac-58:

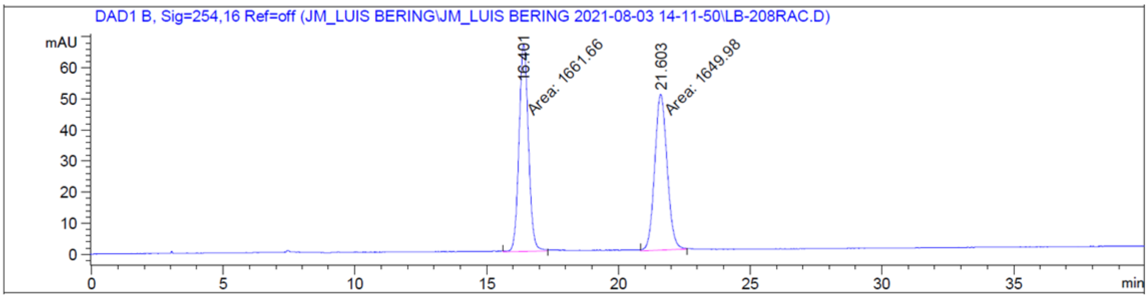

Signal 2: DAD1 B, Sig=254,16 Ref=off

Peak #	RetTime [min]	Type	Width [min]	Area [mAU*s]	Height [mAU]	Area %
1	16.401	MM	0.4156	1661.65601	66.63227	50.1763
2	21.603	MM	0.5487	1649.98193	50.11978	49.8237
Totals :				3311.63794	116.75205	

Chiral HPLC trace for 58.

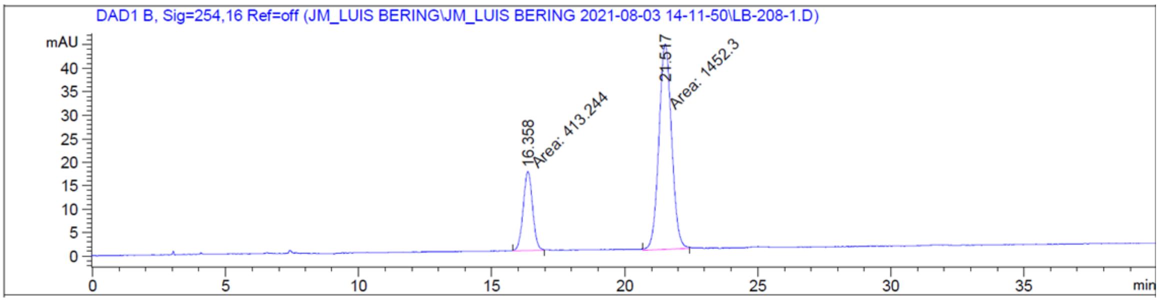

Signal 2: DAD1 B, Sig=254,16 Ref=off

Peak #	RetTime [min]	Type	Width [min]	Area [mAU*s]	Height [mAU]	Area %
1	16.358	MM	0.4103	413.24371	16.78608	22.1514
2	21.517	MM	0.5543	1452.29626	43.66717	77.8486
Totals :				1865.53998	60.45325	

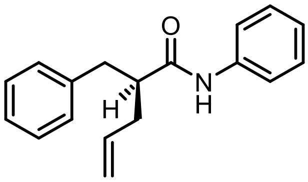

^{1}H NMR spectrum of **59** from the integrated synthesis.

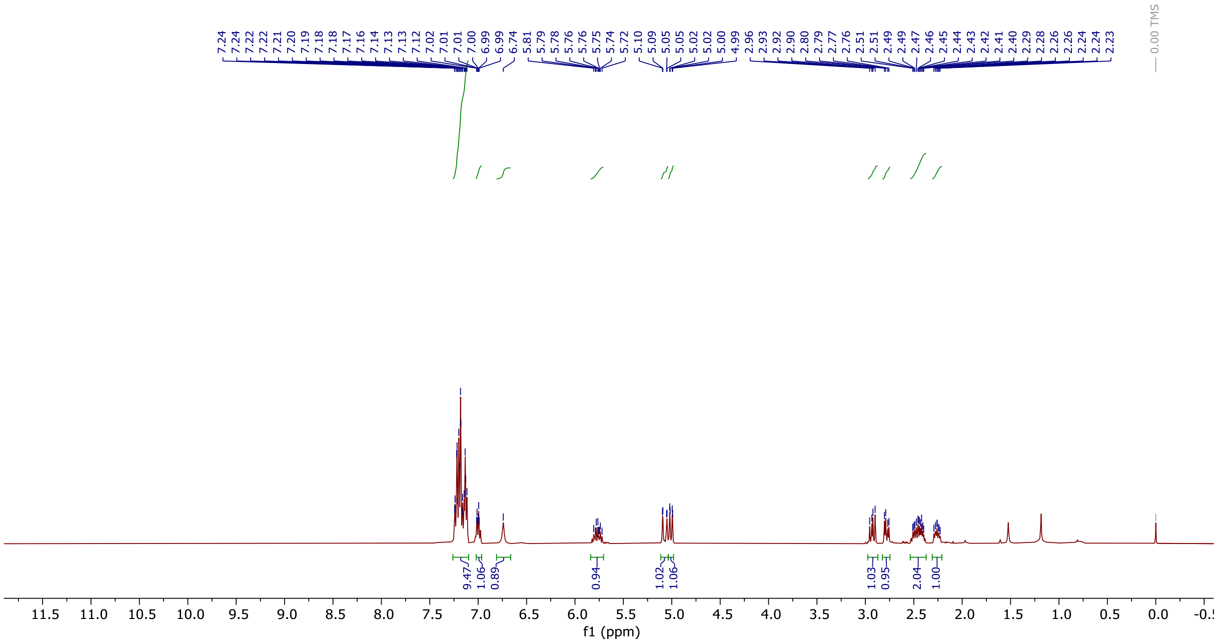

^{13}C NMR spectrum of **59** from the integrated synthesis.

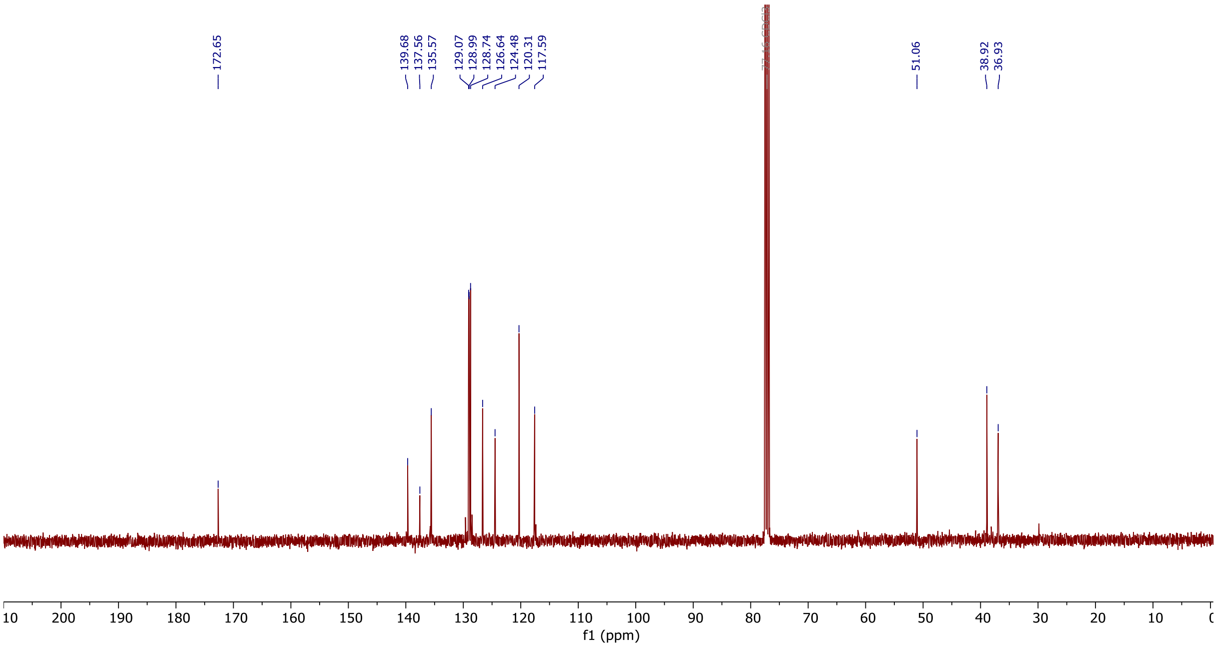

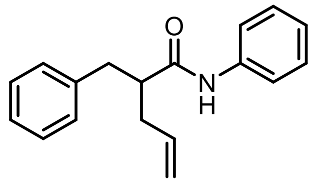

^{1}H NMR spectrum of rac-**59** from standard synthesis.

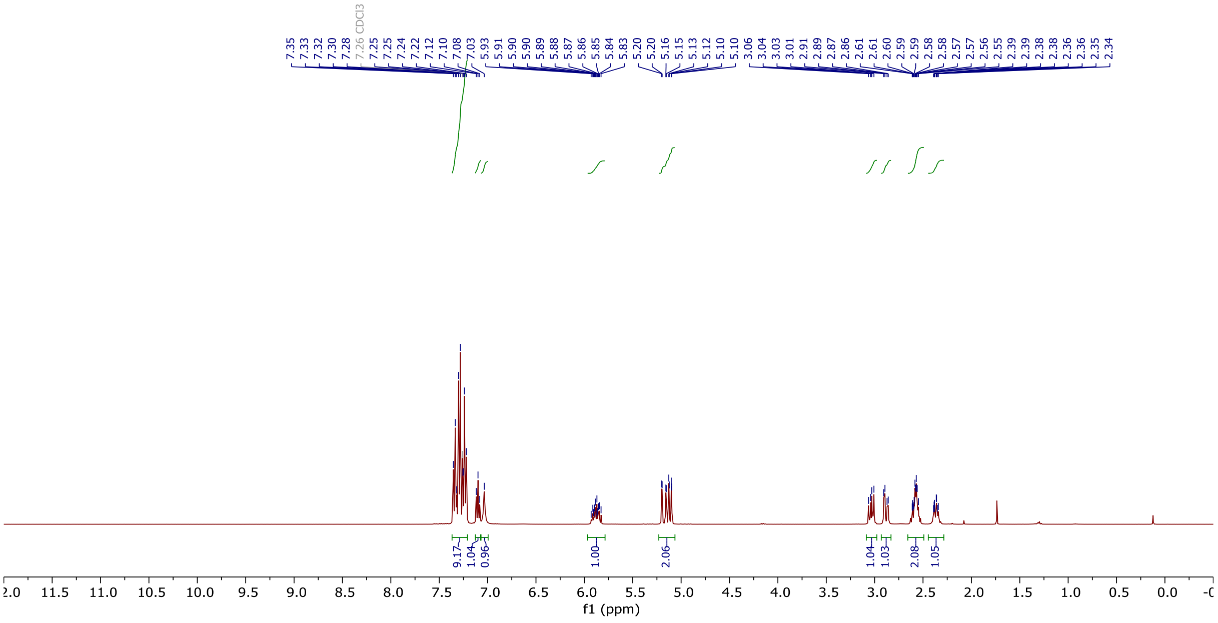

^{13}C NMR spectrum of rac-**59** from standard synthesis.

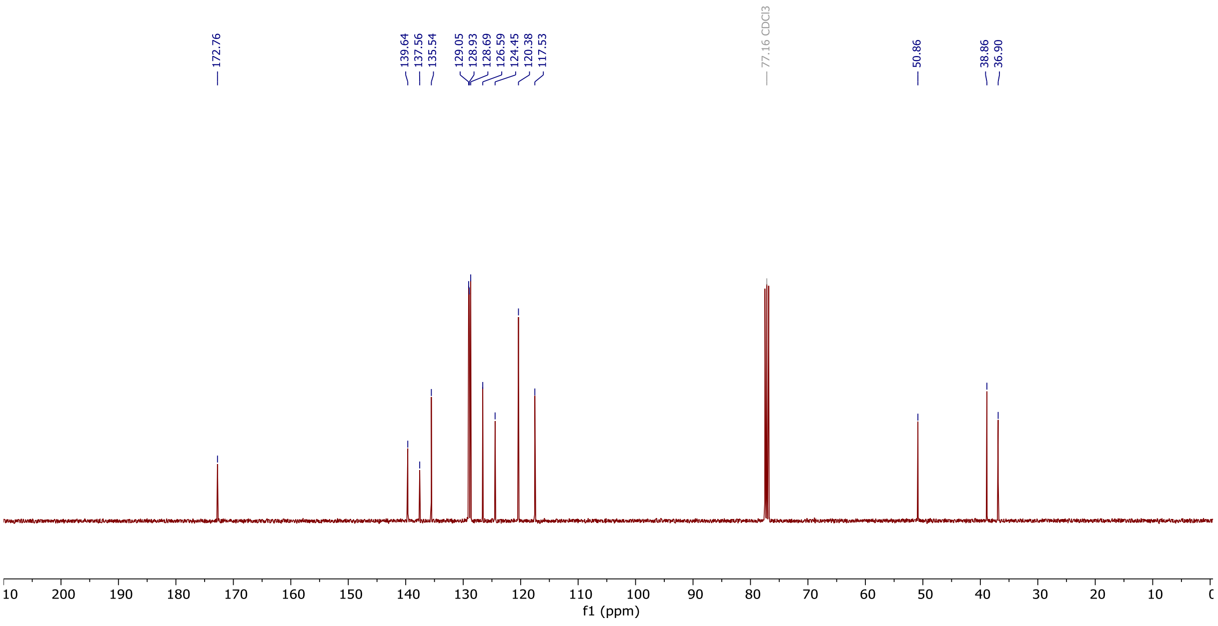

Chiral HPLC trace for rac-59:

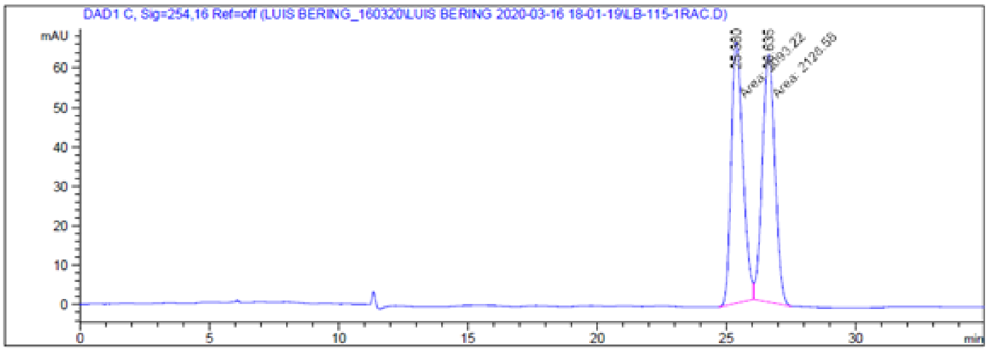

Area Percent Report

Sorted By : Signal
Multiplier : 1.0000
Dilution : 1.0000
Do not use Multiplier & Dilution Factor with ISTDs

Signal 1: DAD1 C, Sig=254,16 Ref=off

Peak #	RetTime [min]	Type	Width [min]	Area [mAU*s]	Height [mAU]	Area %
1	25.380	MM T	0.6263	2093.22095	66.17777	49.5812
2	26.635	MM T	0.5656	2128.58472	62.72195	50.4188

Chiral HPLC trace for 59.

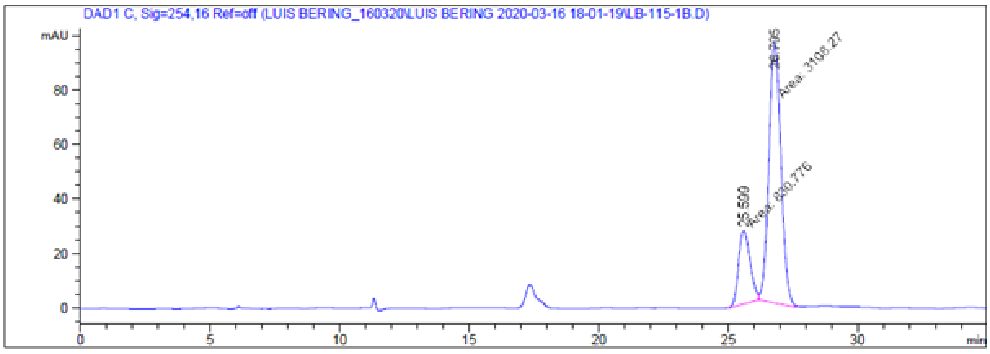

Area Percent Report

Sorted By : Signal
Multiplier : 1.0000
Dilution : 1.0000
Do not use Multiplier & Dilution Factor with ISTDs

Signal 1: DAD1 C, Sig=254,16 Ref=off

Peak #	RetTime [min]	Type	Width [min]	Area [mAU*s]	Height [mAU]	Area %
1	25.599	MM T	0.5158	830.77594	26.84508	21.0908
2	26.795	MM T	0.5419	3108.26904	95.59007	78.9092

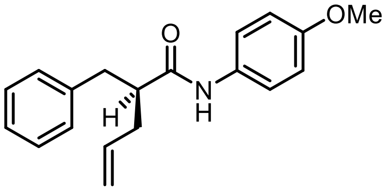

^{1}H NMR spectrum of **60** from the integrated synthesis.

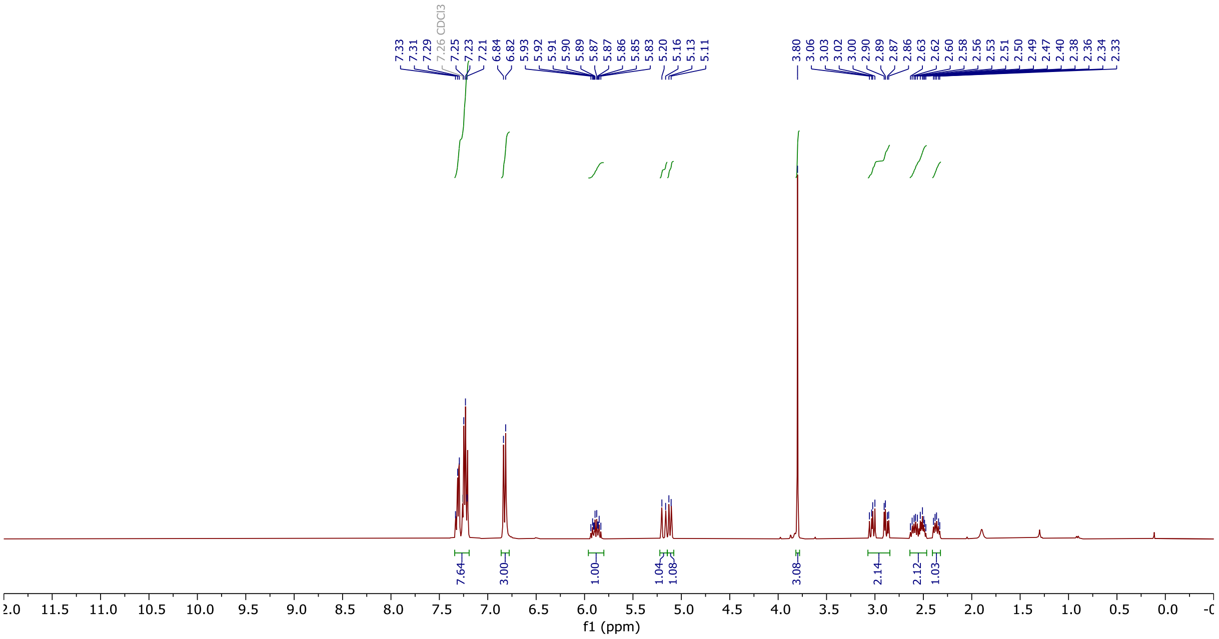

^{13}C NMR spectrum of **60** from the integrated synthesis.

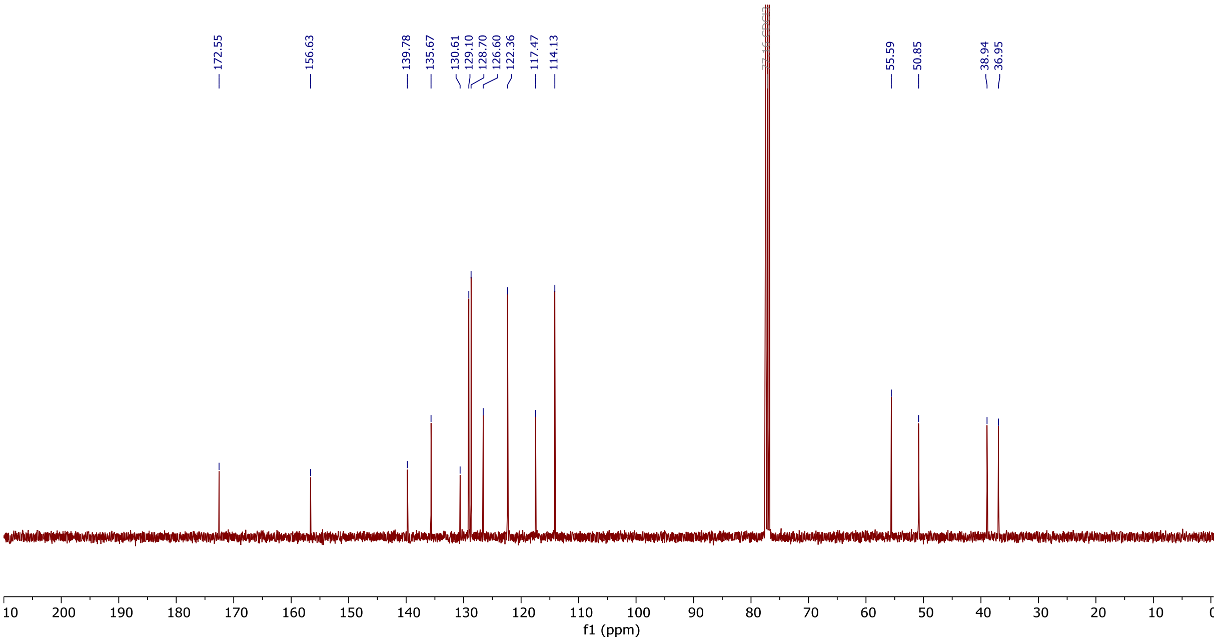

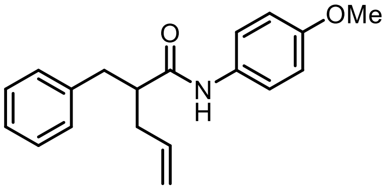

^{1}H NMR spectrum of rac-**60** from standard synthesis.

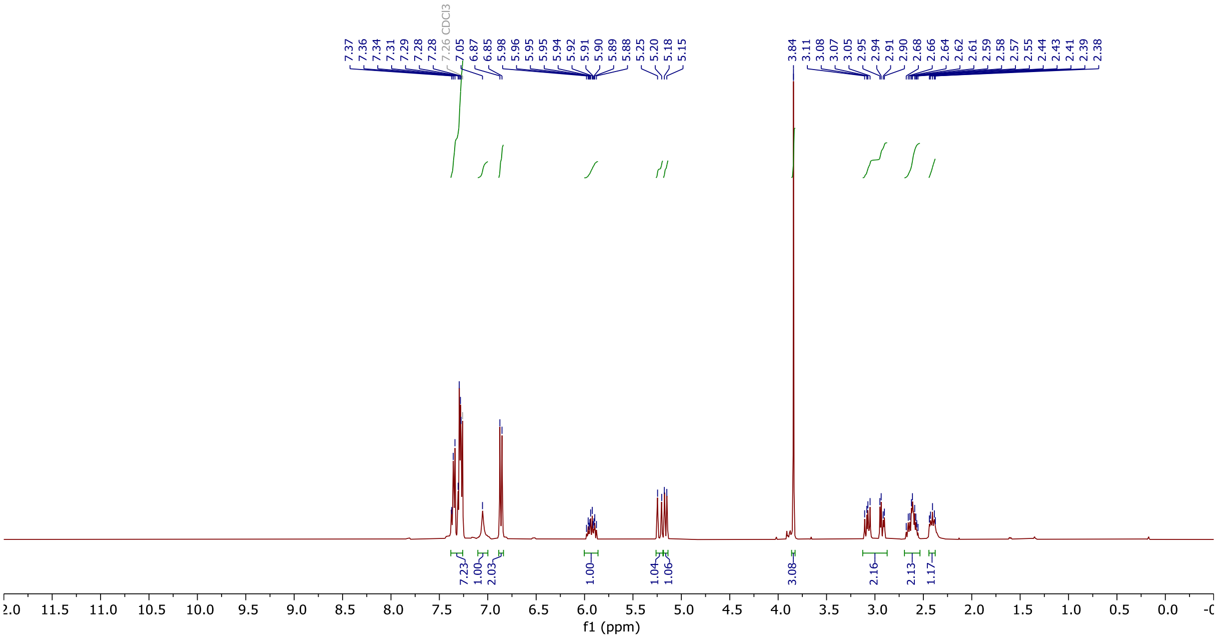

^{13}C NMR spectrum of rac-**60** from standard synthesis.

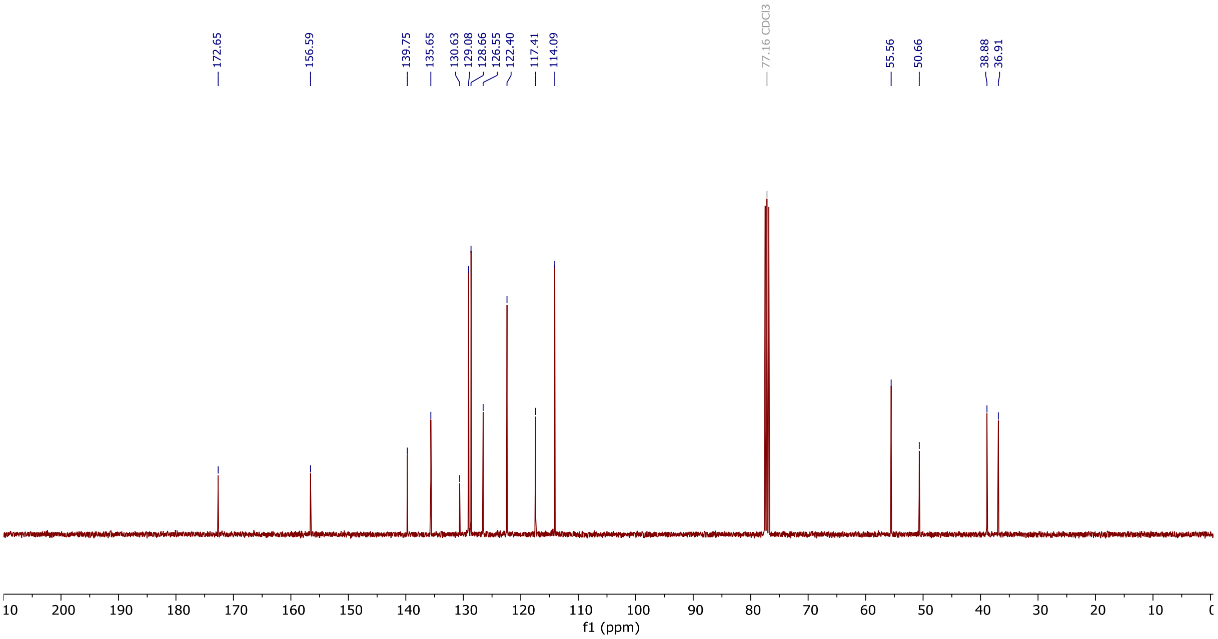

Chiral HPLC trace for rac-60:

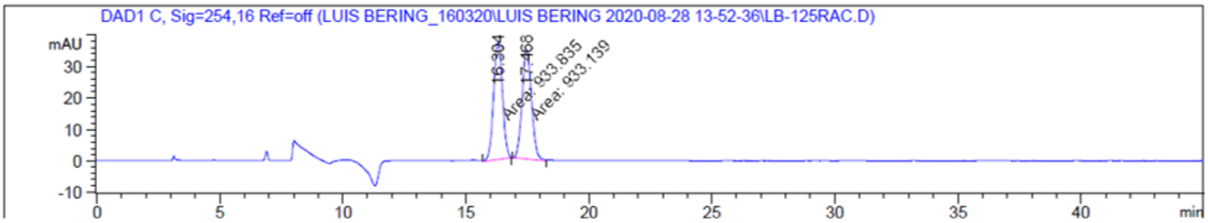

==

Area Percent Report

==

Sorted By : Signal
Multiplier : 1.0000
Dilution : 1.0000
Use Multiplier & Dilution Factor with ISTDs

Signal 1: DAD1 C, Sig=254,16 Ref=off

Peak #	RetTime [min]	Type	Width [min]	Area [mAU*s]	Height [mAU]	Area %
1	16.304	MM	0.4157	933.83545	37.43636	50.0187
2	17.468	MM	0.4479	933.13885	34.72518	49.9813

Chiral HPLC trace for 60.

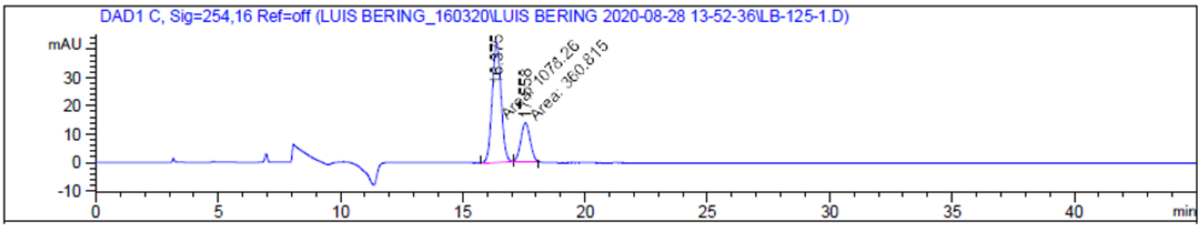

==

Area Percent Report

==

Sorted By : Signal
Multiplier : 1.0000
Dilution : 1.0000
Use Multiplier & Dilution Factor with ISTDs

Signal 1: DAD1 C, Sig=254,16 Ref=off

Peak #	RetTime [min]	Type	Width [min]	Area [mAU*s]	Height [mAU]	Area %
1	16.375	MM	0.4215	1078.25854	42.63397	74.9273
2	17.558	MM	0.4382	360.81464	13.72388	25.0727

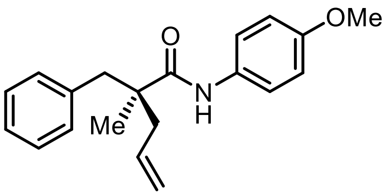

^{1}H NMR spectrum of **61** from the integrated synthesis.

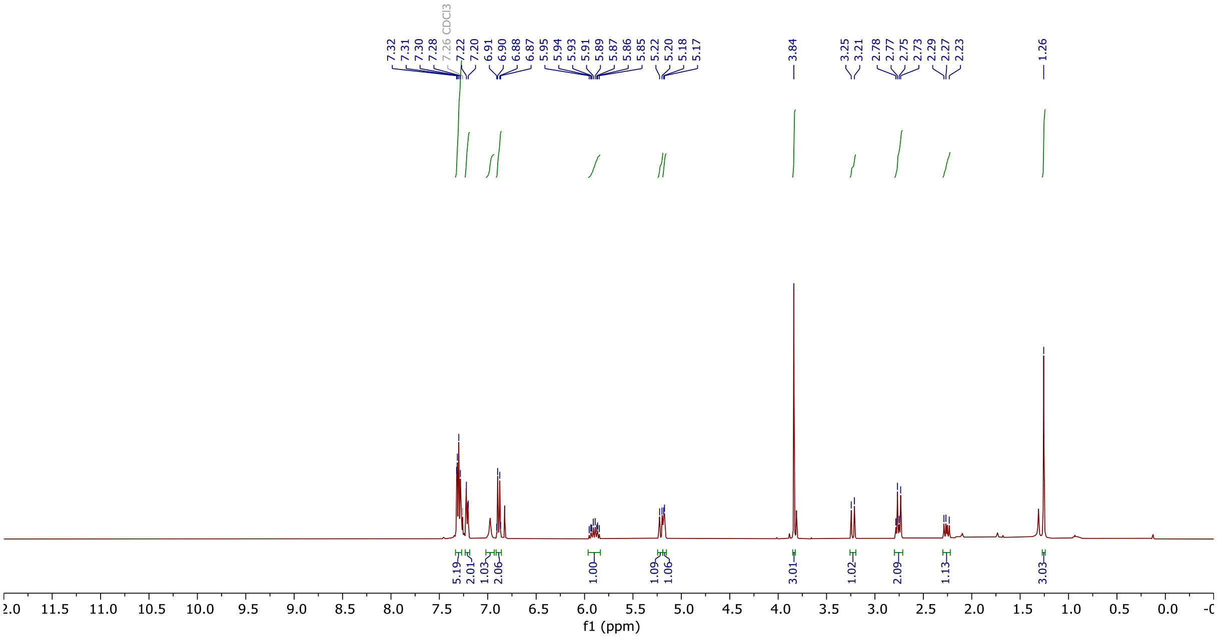

^{13}C NMR spectrum of **61** from the integrated synthesis.

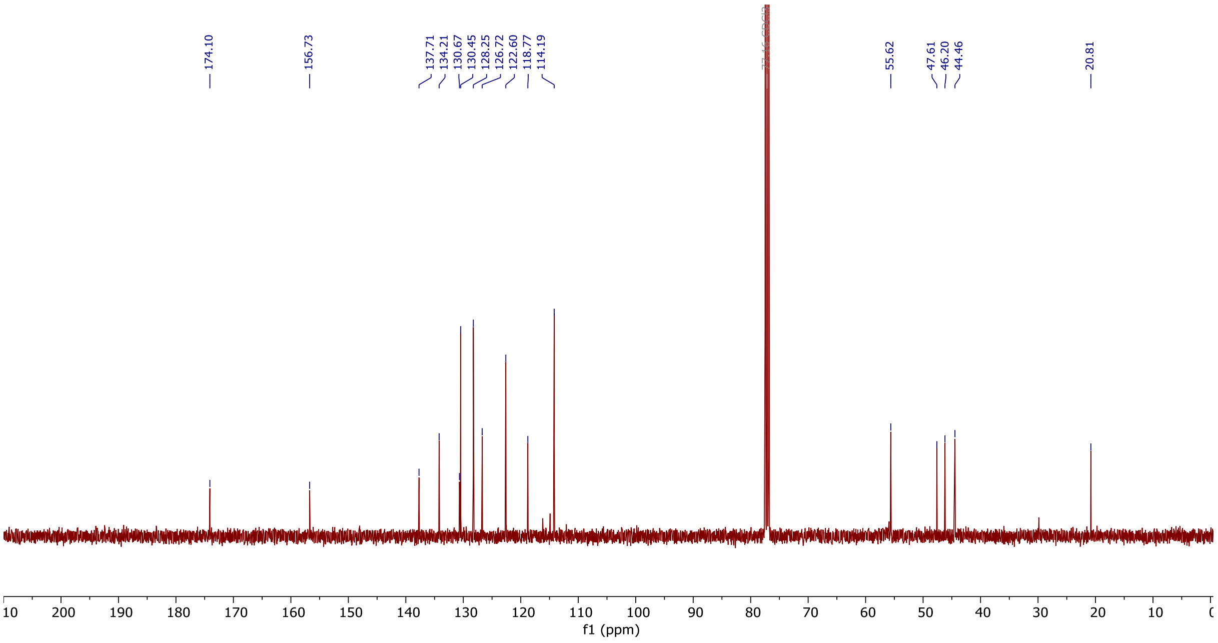

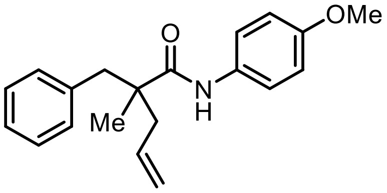

^{1}H NMR spectrum of rac-**61** from standard synthesis.

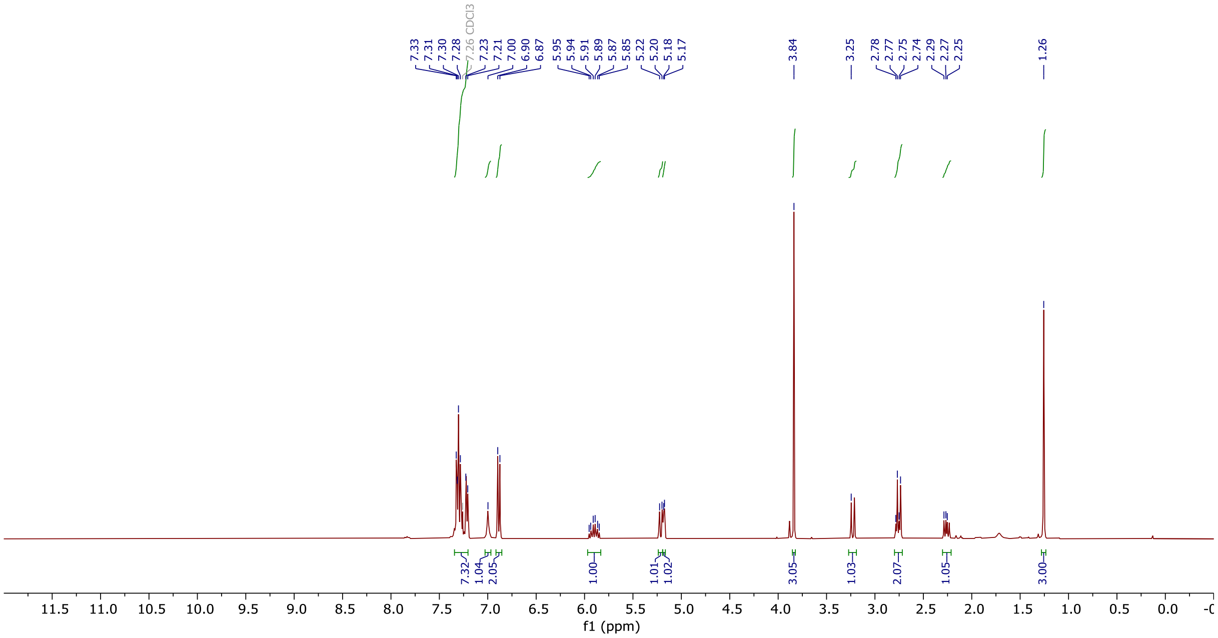

^{13}C NMR spectrum of rac-**61** from standard synthesis.

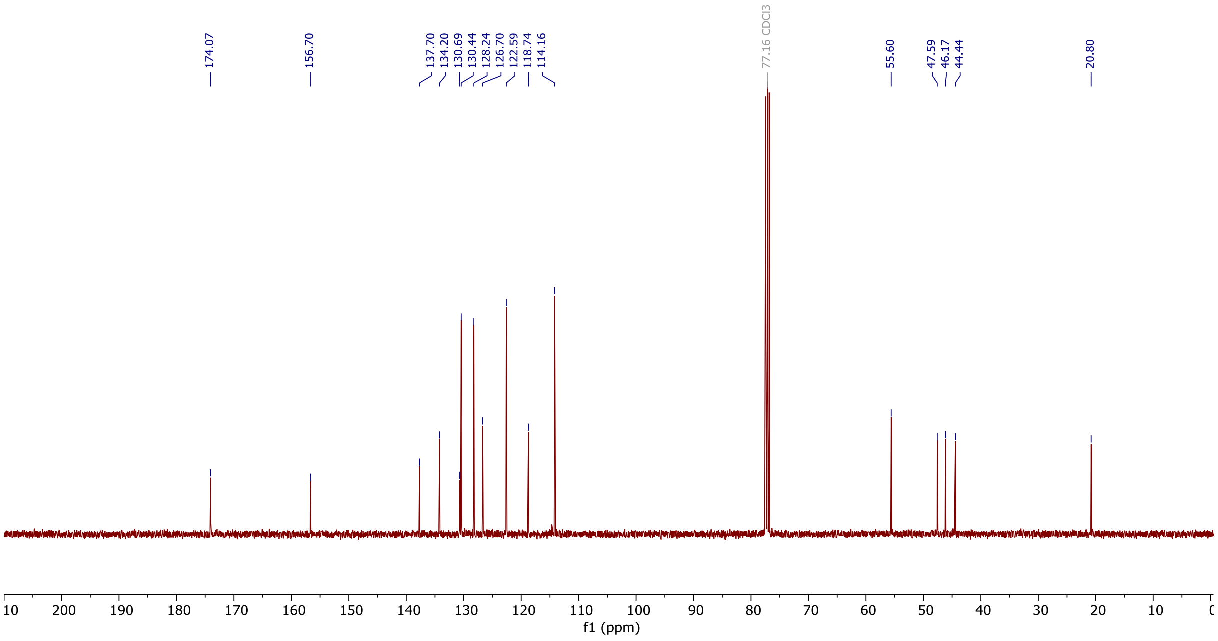

Chiral HPLC trace for rac-61:

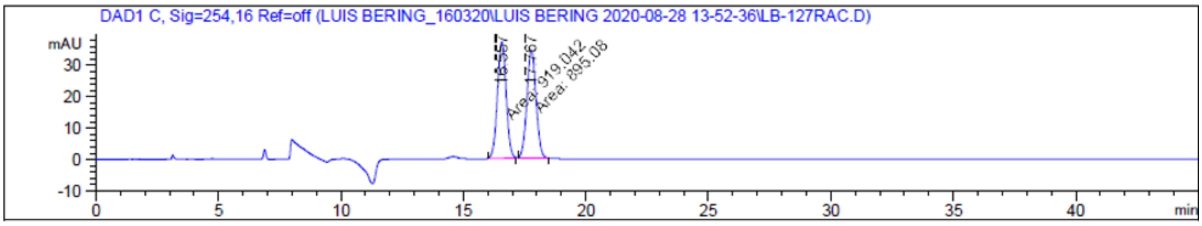


```
====================================================================
                        Area Percent Report
====================================================================
```

Sorted By : Signal
Multiplier : 1.0000
Dilution : 1.0000
Use Multiplier & Dilution Factor with ISTDs

Signal 1: DAD1 C, Sig=254,16 Ref=off

Peak #	RetTime [min]	Type	Width [min]	Area [mAU*s]	Height [mAU]	Area %
1	16.557	MM	0.4112	919.04205	37.24782	50.6604
2	17.767	MM	0.4329	895.08020	34.46050	49.3396

Chiral HPLC trace for 61.

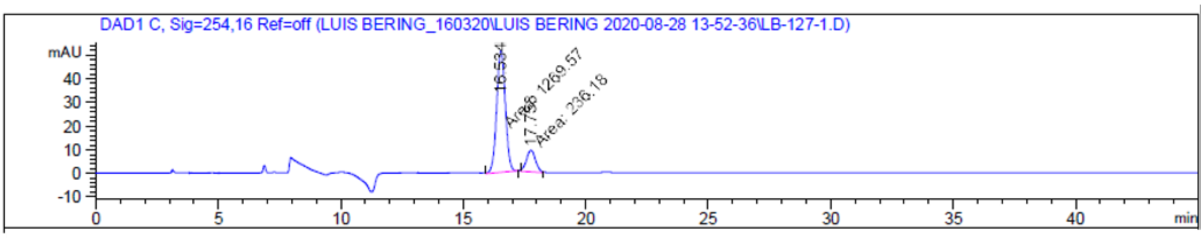


```
====================================================================
                        Area Percent Report
====================================================================
```

Sorted By : Signal
Multiplier : 1.0000
Dilution : 1.0000
Use Multiplier & Dilution Factor with ISTDs

Signal 1: DAD1 C, Sig=254,16 Ref=off

Peak #	RetTime [min]	Type	Width [min]	Area [mAU*s]	Height [mAU]	Area %
1	16.534	MM	0.4087	1269.56714	51.76629	84.3148
2	17.758	MM	0.4317	236.17955	9.11869	15.6852

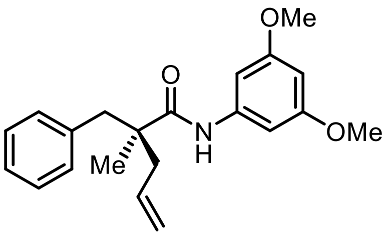

^{1}H NMR spectrum of **62** from the integrated synthesis.

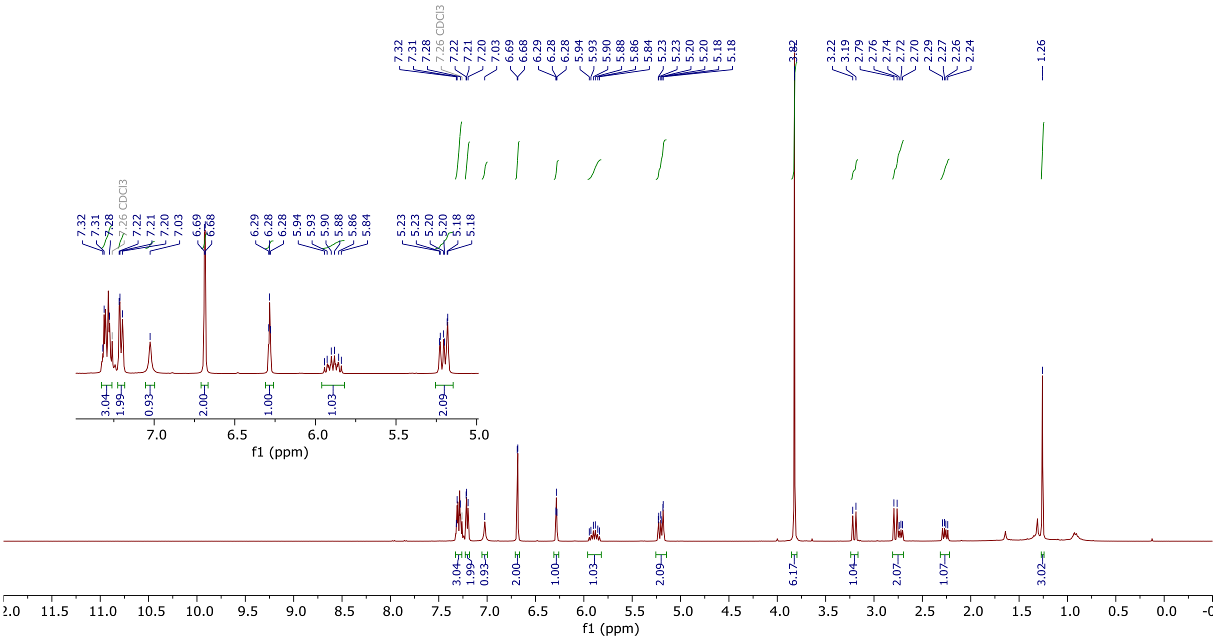

^{13}C NMR spectrum of **62** from the integrated synthesis.

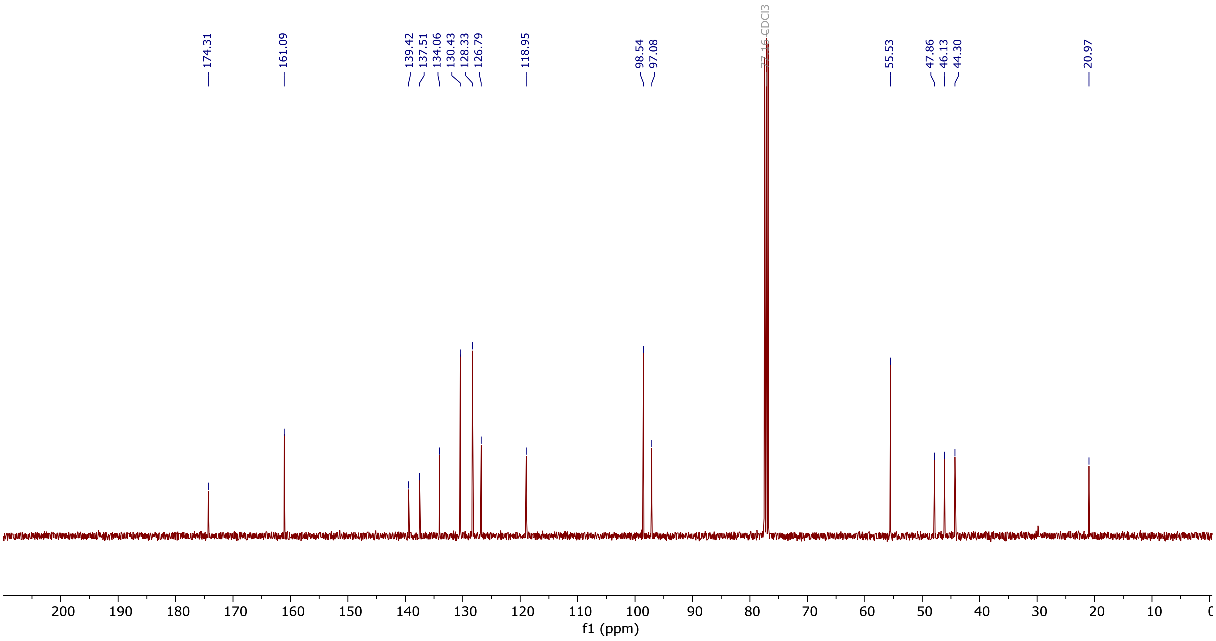

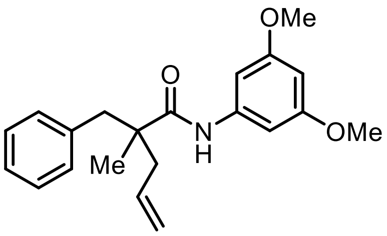

^{1}H NMR spectrum of rac-**62** from standard synthesis.

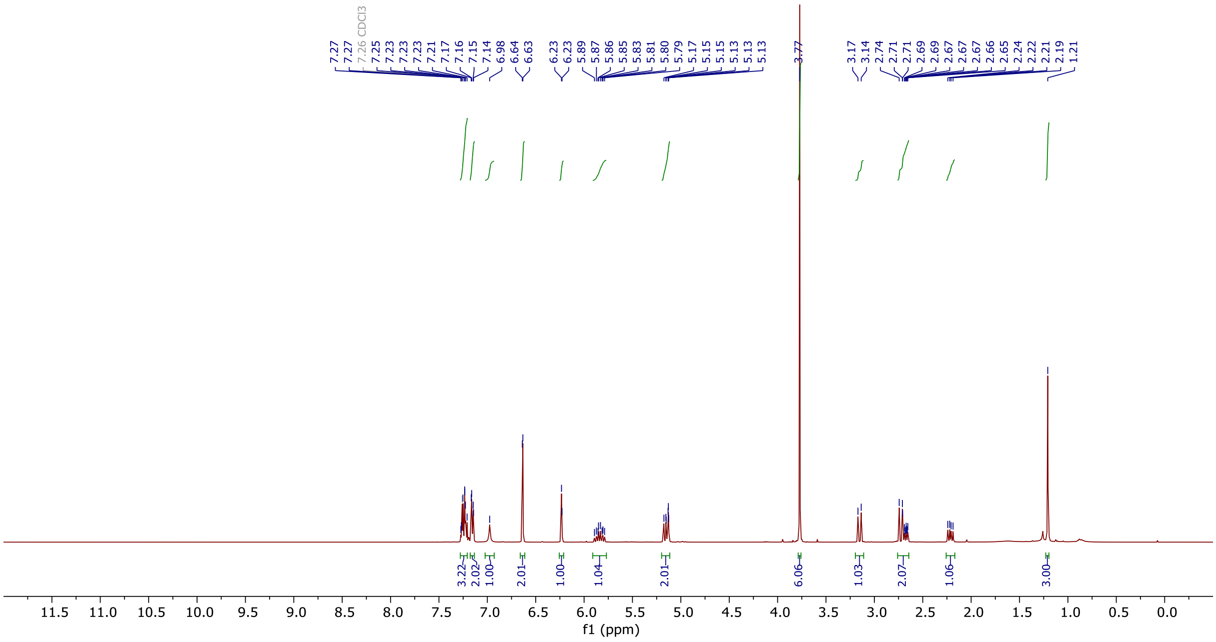

^{13}C NMR spectrum of rac-**62** from standard synthesis.

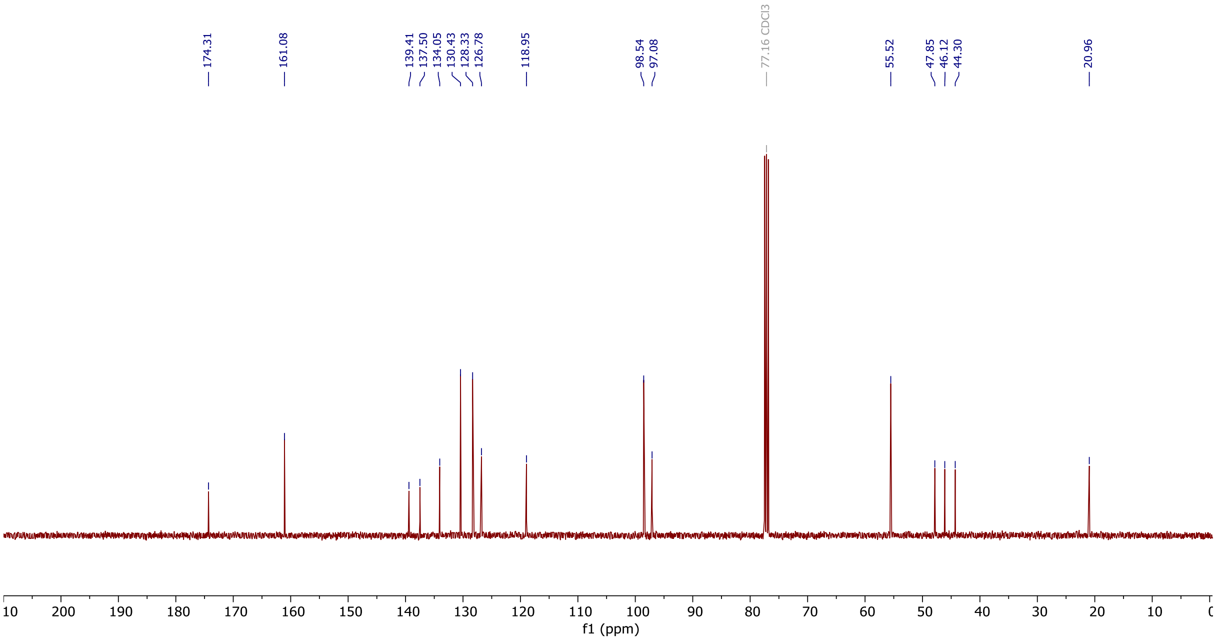

Chiral HPLC trace for rac-62:

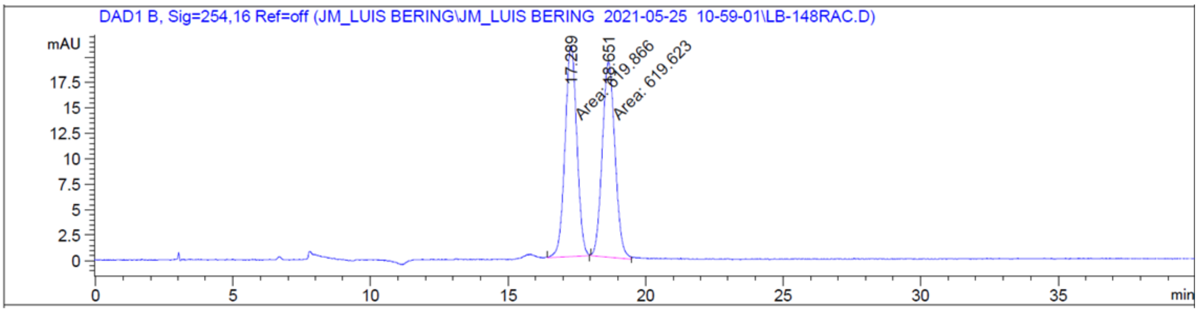

Signal 2: DAD1 B, Sig=254,16 Ref=off

Peak #	RetTime [min]	Type	Width [min]	Area [mAU*s]	Height [mAU]	Area %
1	17.289	MM	0.4987	619.86597	20.71784	50.0098
2	18.651	MM	0.5372	619.62323	19.22468	49.9902
Totals :				1239.48920	39.94252	

Chiral HPLC trace for 62.

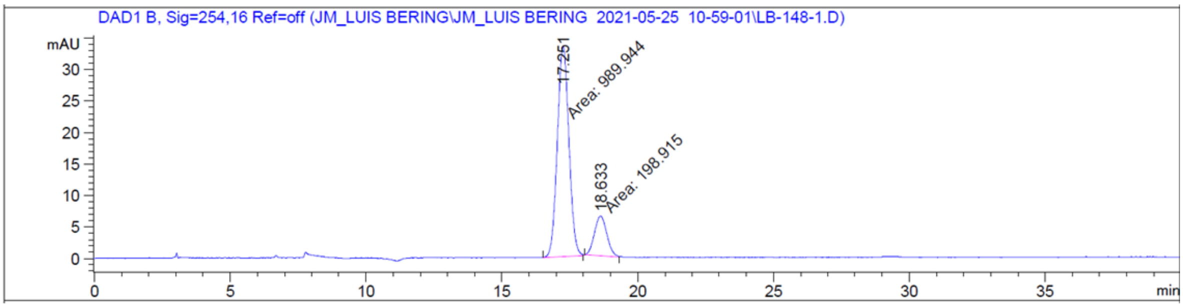

Signal 2: DAD1 B, Sig=254,16 Ref=off

Peak #	RetTime [min]	Type	Width [min]	Area [mAU*s]	Height [mAU]	Area %
1	17.251	MM	0.4936	989.94360	33.42439	83.2684
2	18.633	MM	0.5260	198.91516	6.30273	16.7316
Totals :				1188.85876	39.72712	

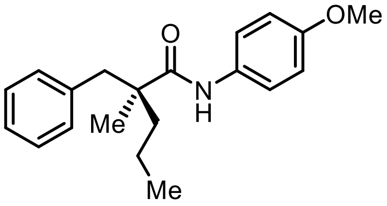

^{1}H NMR spectrum of **63** from the integrated synthesis.

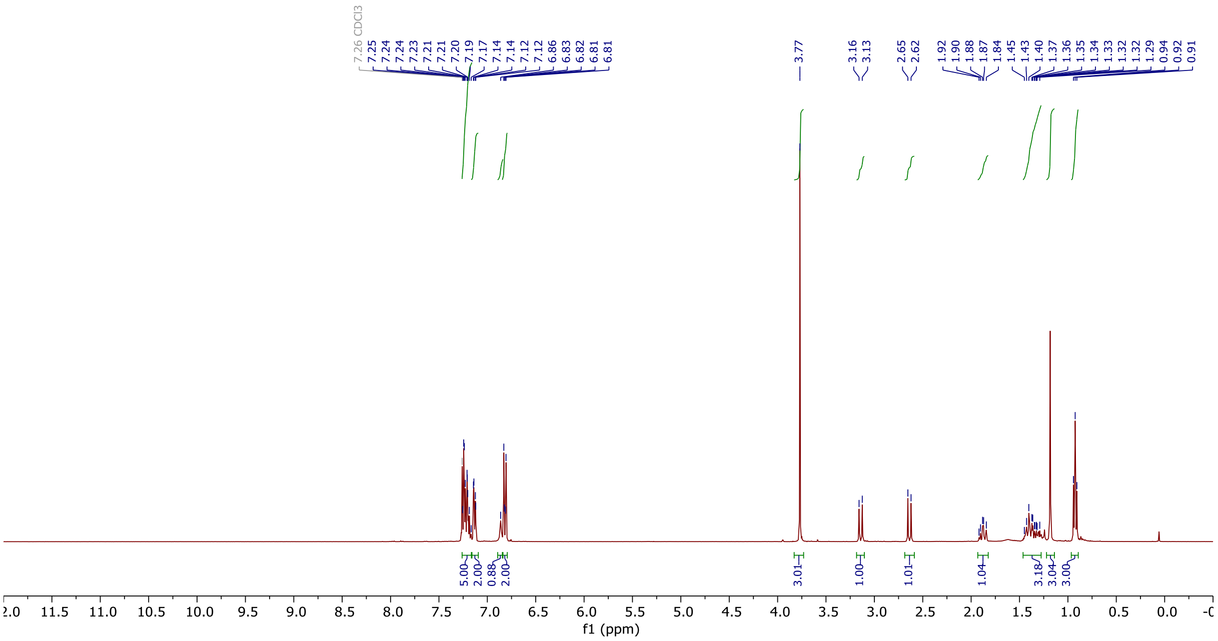

^{13}C NMR spectrum of **63** from the integrated synthesis.

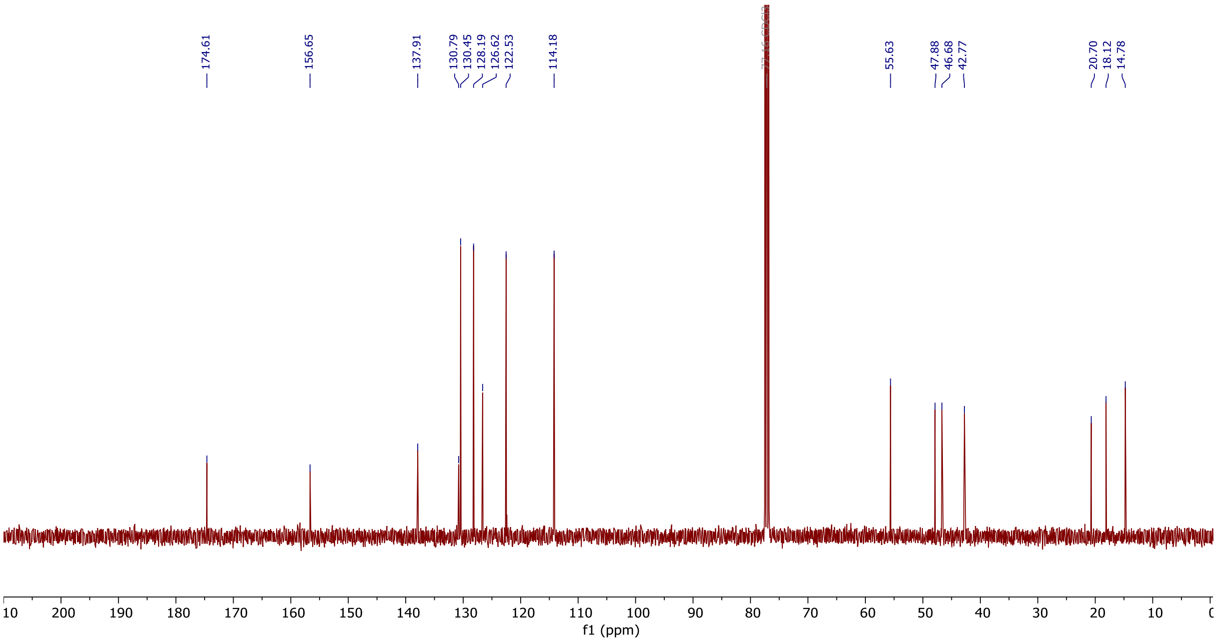

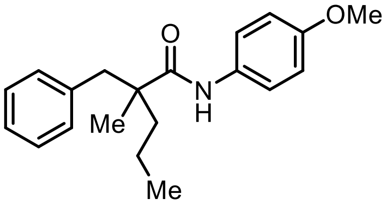

^{1}H NMR spectrum of rac-**63** from standard synthesis.

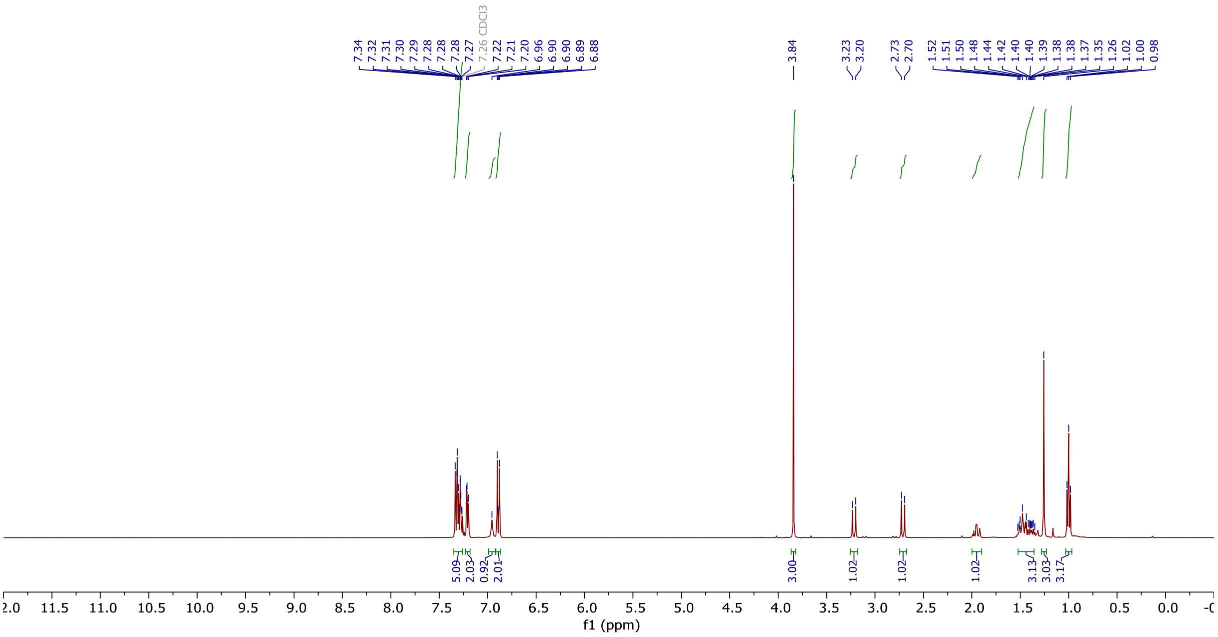

^{13}C NMR spectrum of rac-**63** from standard synthesis.

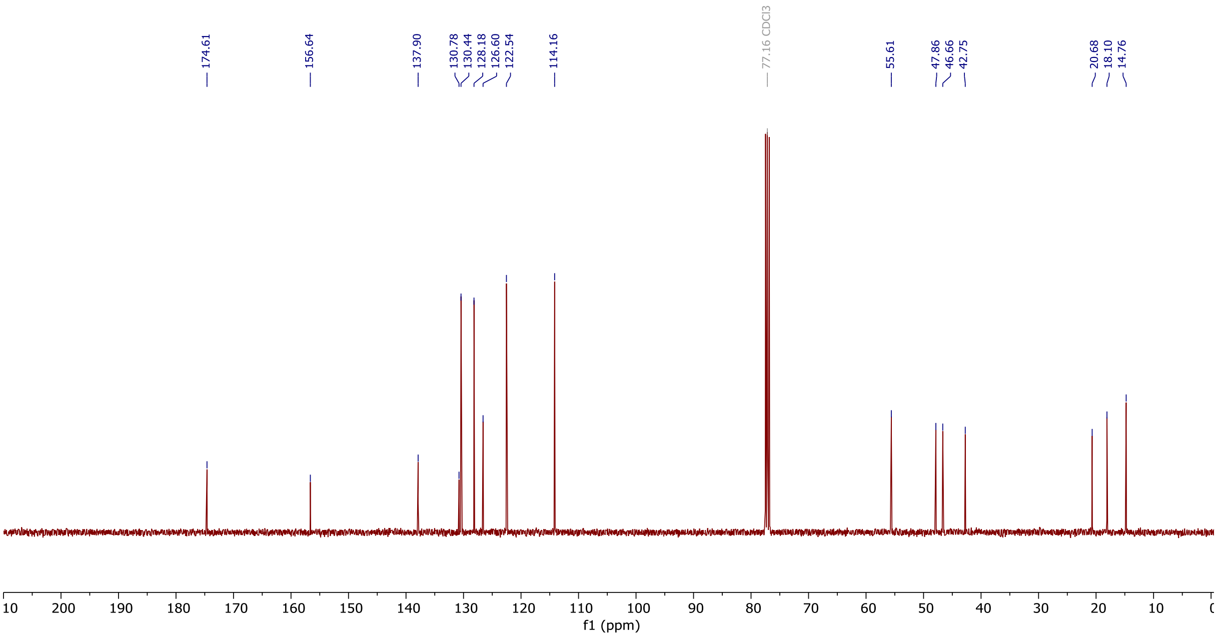

Chiral HPLC trace for rac-63:

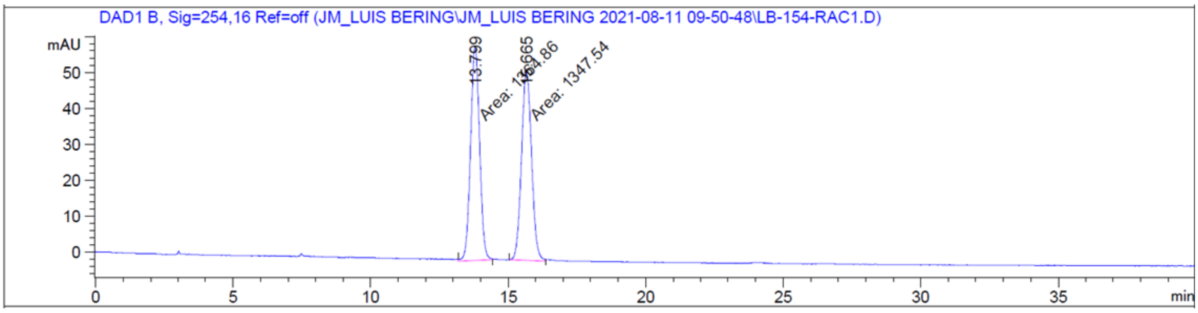

Signal 2: DAD1 B, Sig=254,16 Ref=off

Peak #	RetTime [min]	Type	Width [min]	Area [mAU*s]	Height [mAU]	Area %
1	13.799	MM	0.3821	1364.85779	59.53579	50.3192
2	15.665	MM	0.4266	1347.54443	52.64365	49.6808
Totals :				2712.40222	112.17944	

Chiral HPLC trace for 63.

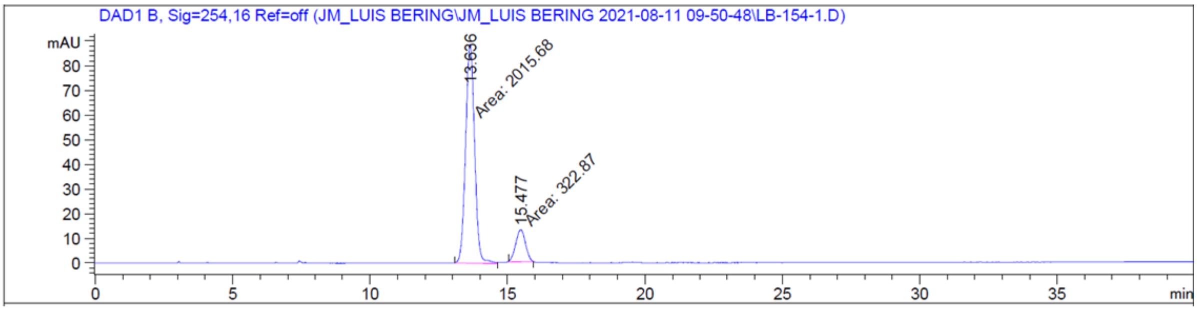

Signal 2: DAD1 B, Sig=254,16 Ref=off

Peak #	RetTime [min]	Type	Width [min]	Area [mAU*s]	Height [mAU]	Area %
1	13.636	MM	0.3790	2015.68152	88.64081	86.1936
2	15.477	MM	0.4117	322.87000	13.07012	13.8064
Totals :				2338.55151	101.71093	

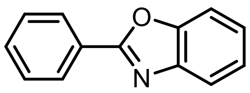

^{1}H NMR spectrum of **65**.

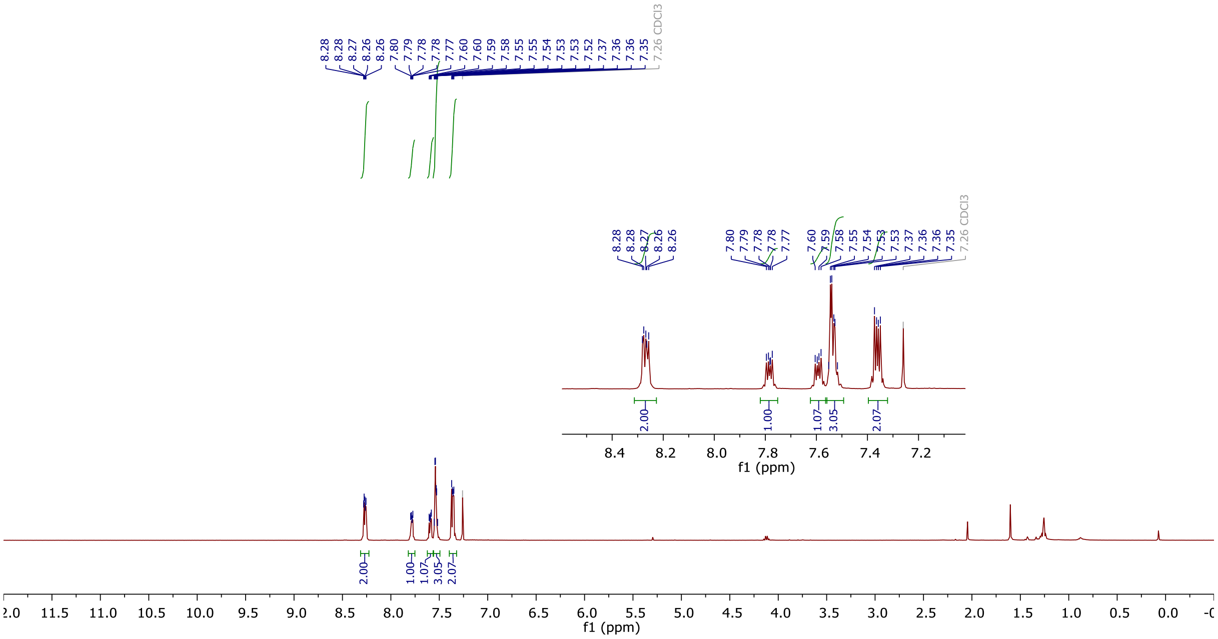

^{13}C NMR spectrum of **65**.

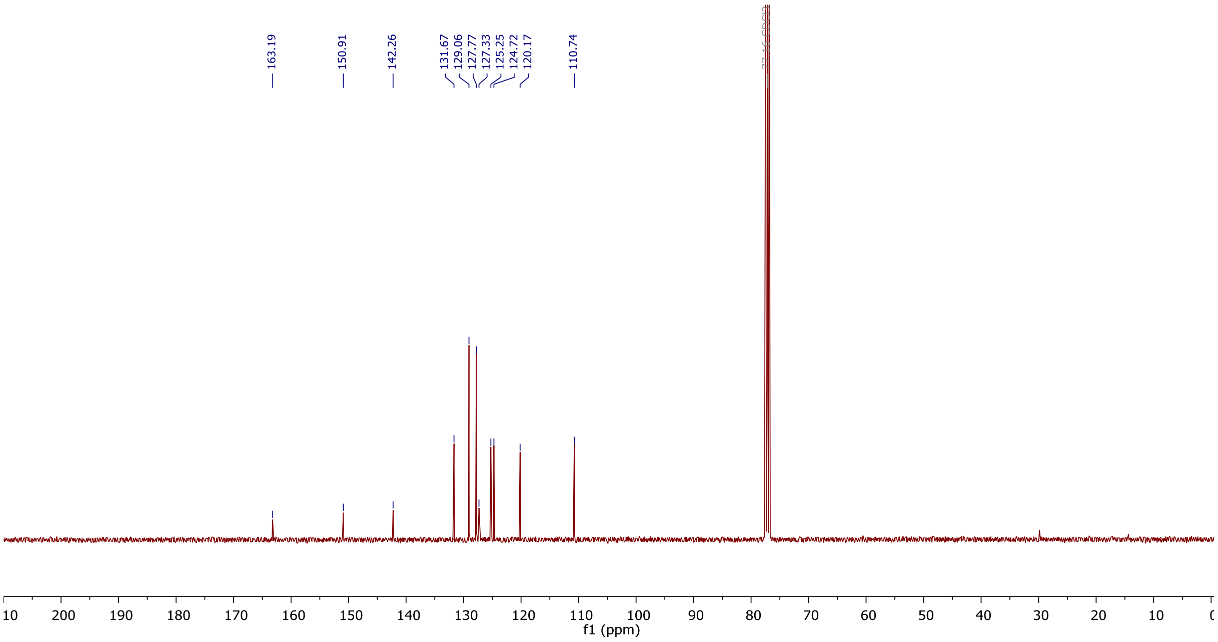

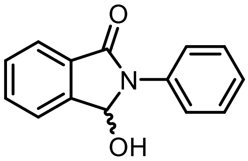

^{1}H NMR spectrum of **67**.

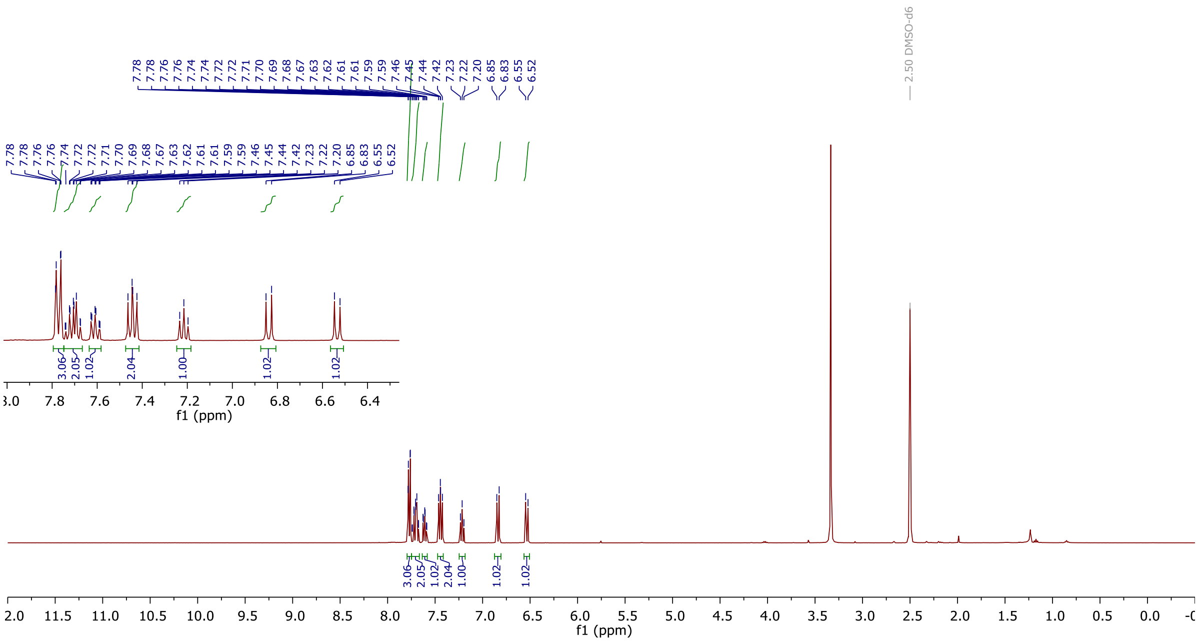

^{13}C NMR spectrum of **67**.

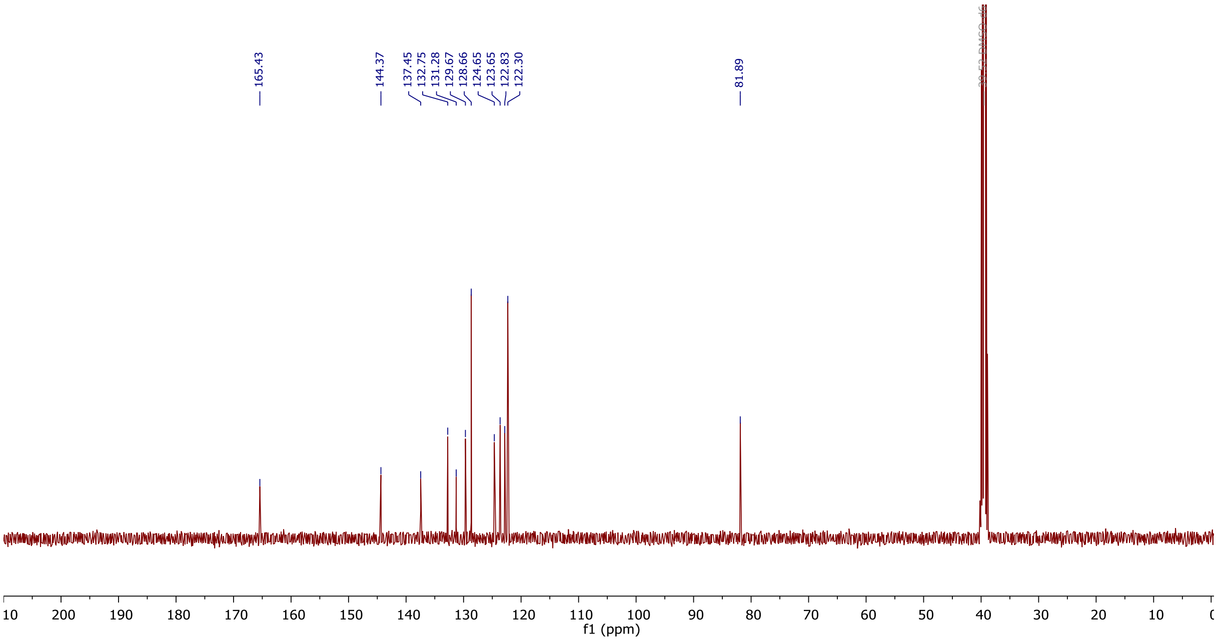

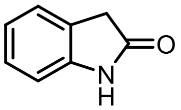

^{1}H NMR spectrum of **69**.

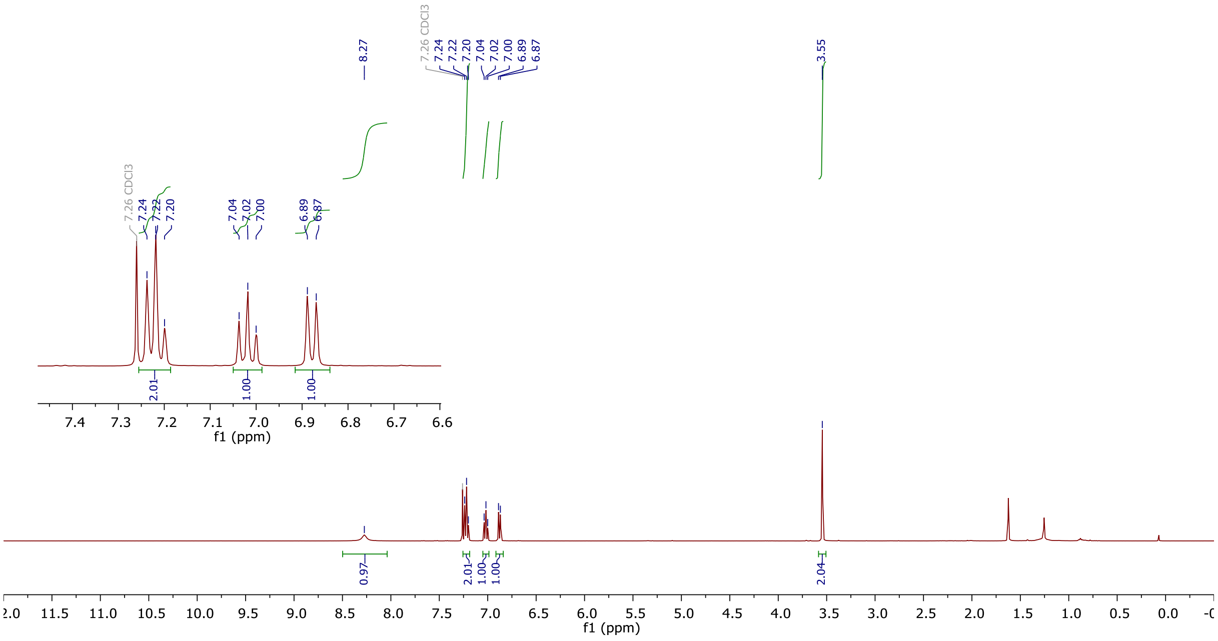

^{13}C NMR spectrum of **69**.

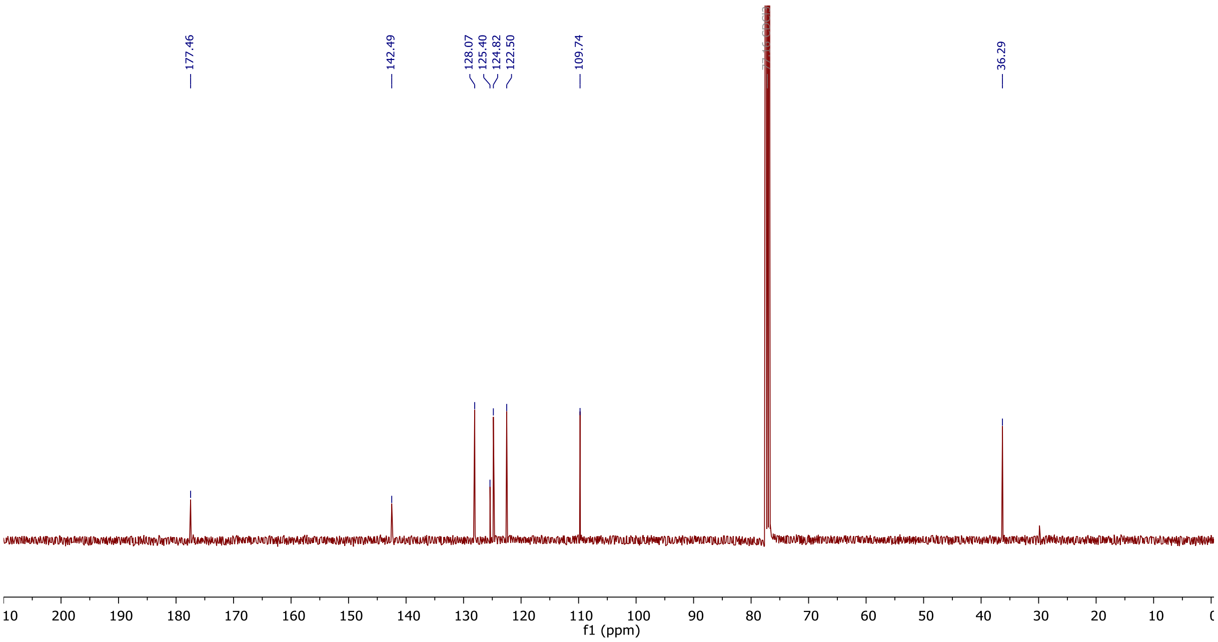

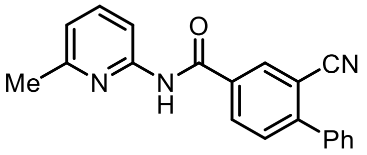

^{1}H NMR spectrum of **72**.

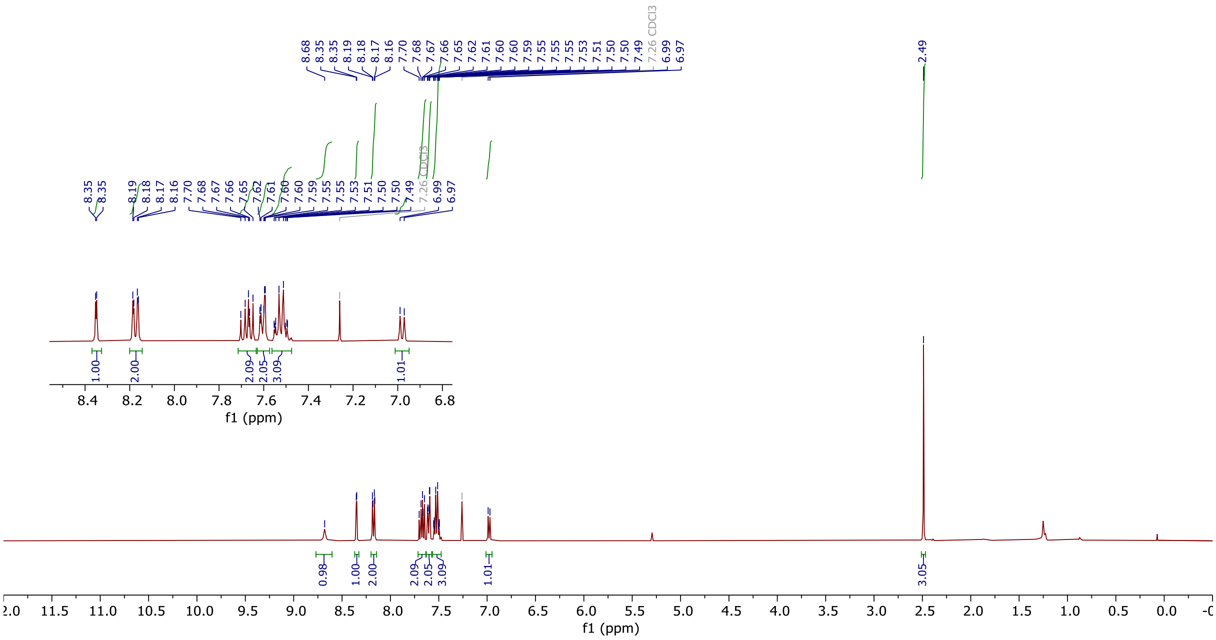

^{13}C NMR spectrum of **72**.

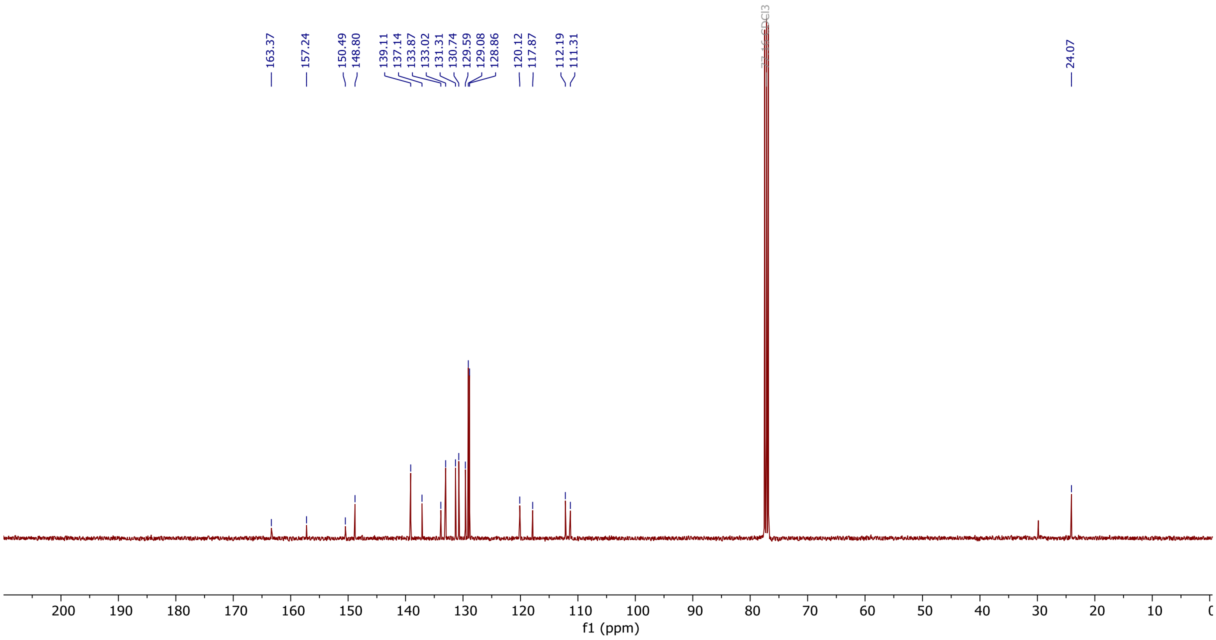

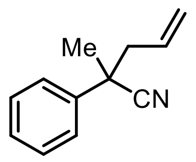

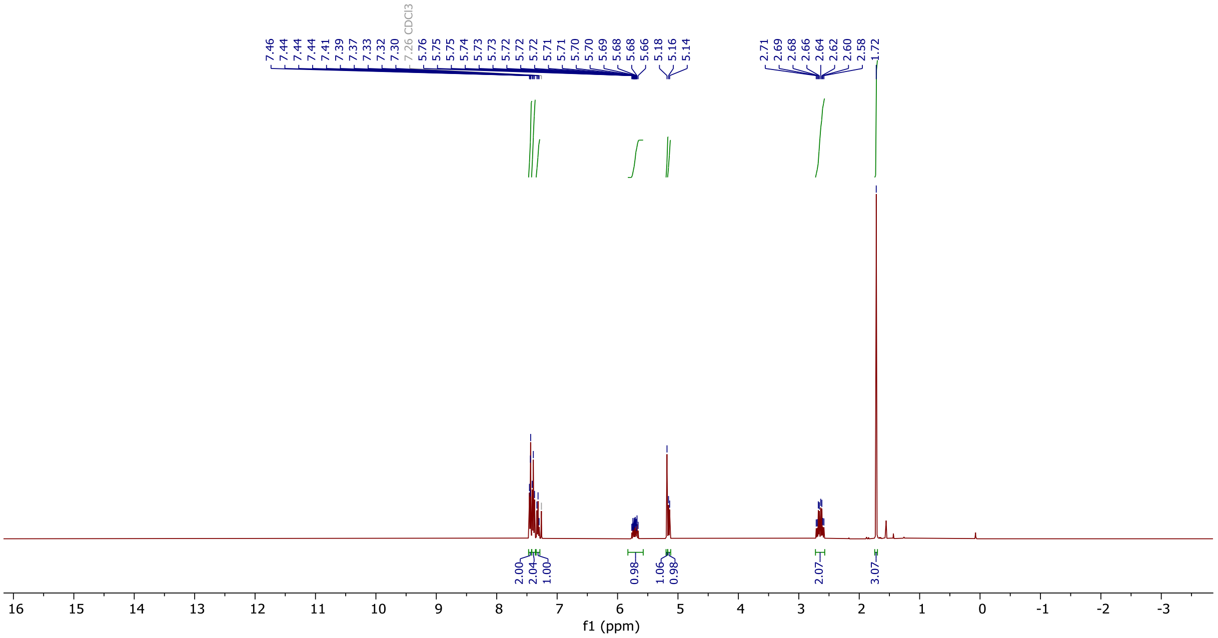

¹H NMR spectrum of **54**.

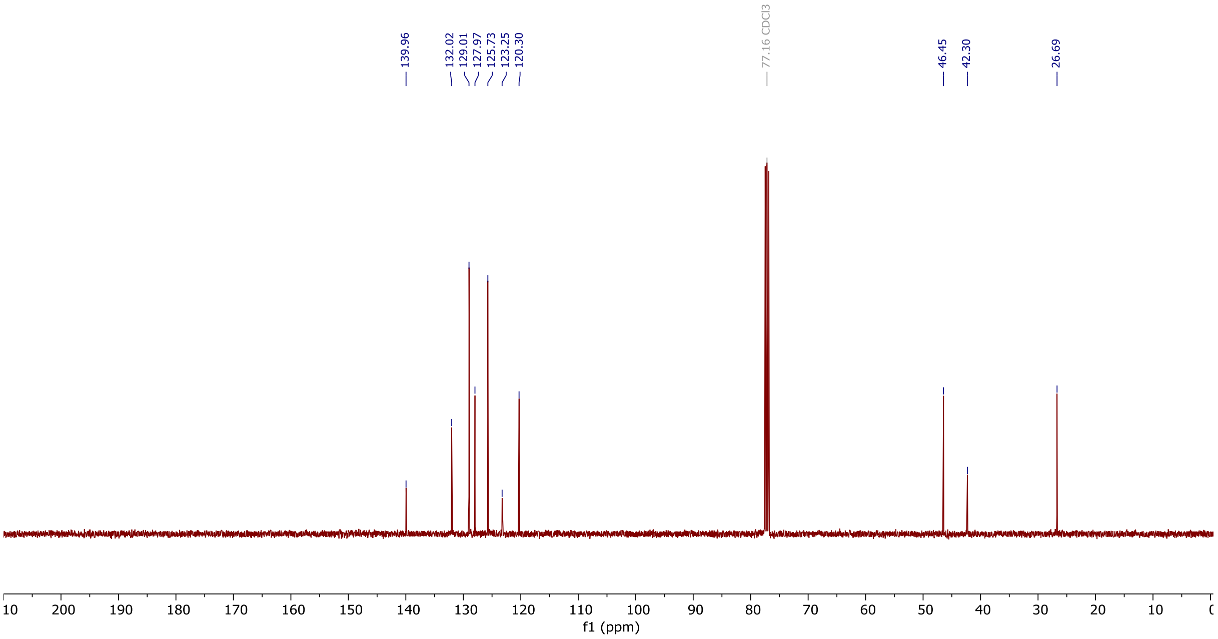

¹³C NMR spectrum of **54**.

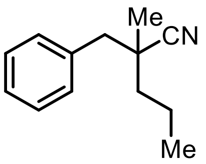

^{1}H NMR spectrum of **64**.

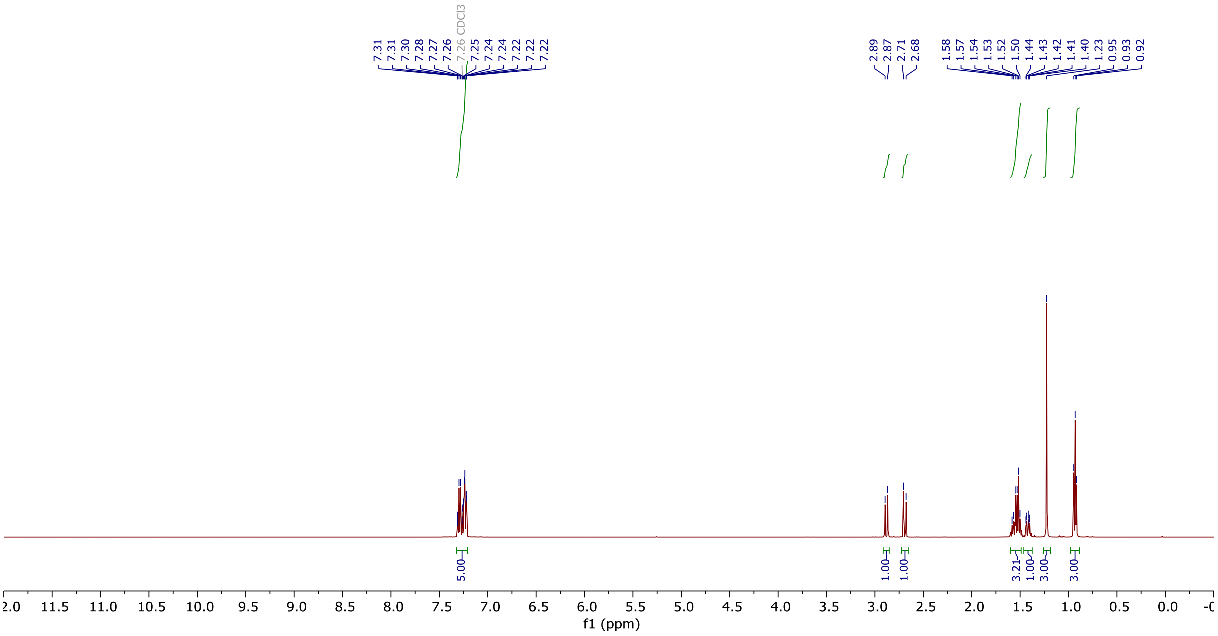

^{13}C NMR spectrum of **64**.

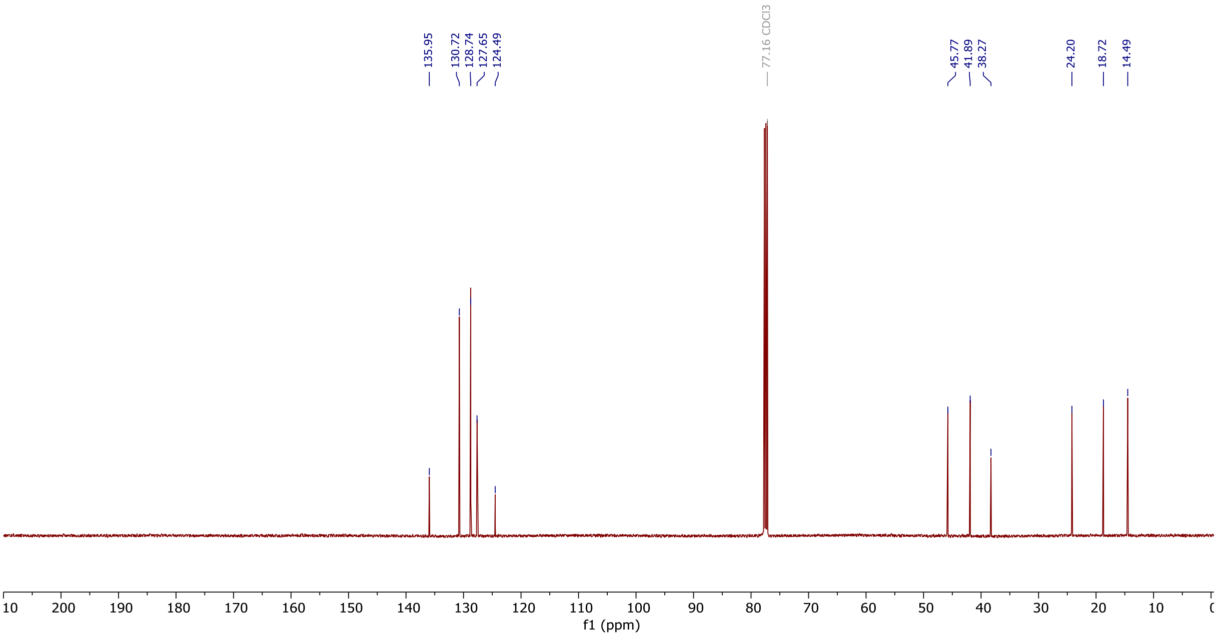

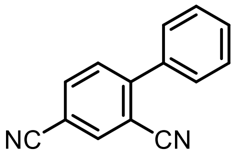

^{1}H NMR spectrum of **70**.

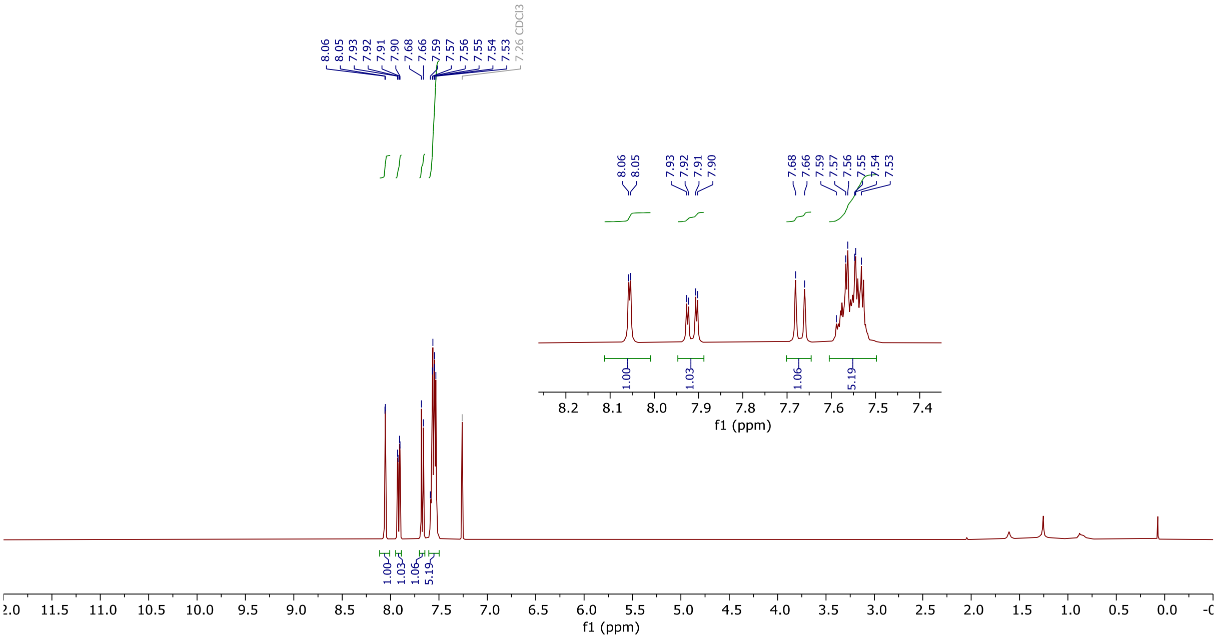

^{13}C NMR spectrum of **70**.

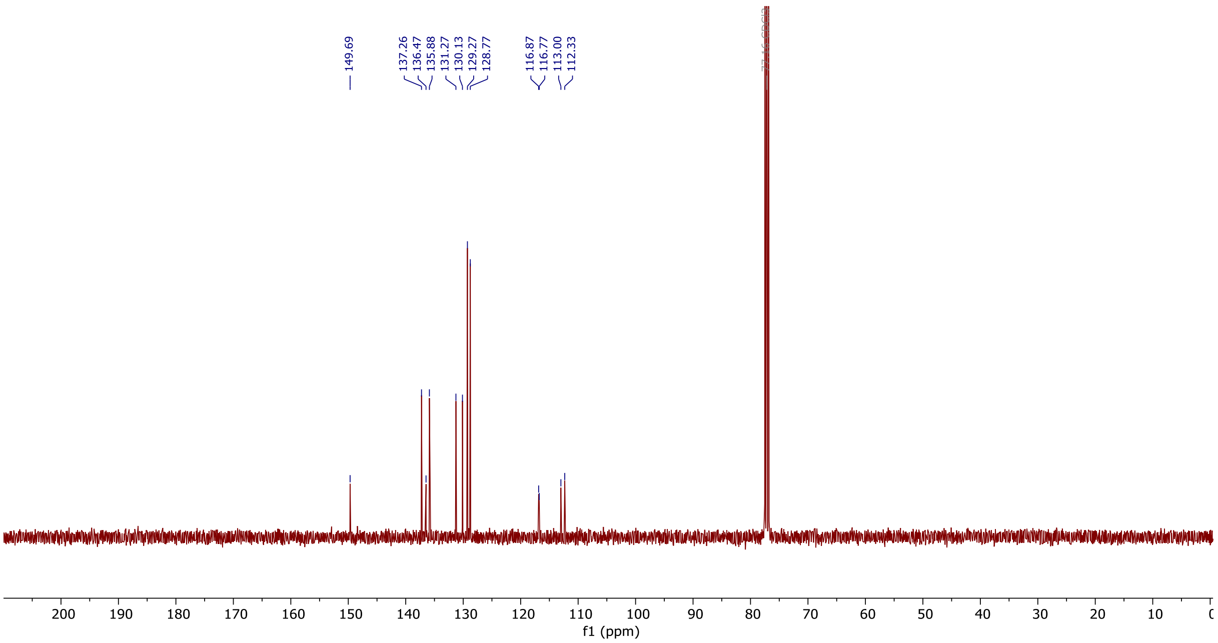

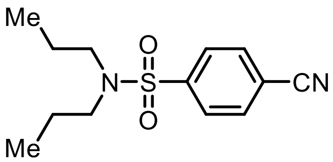

^{1}H NMR spectrum of **73**.

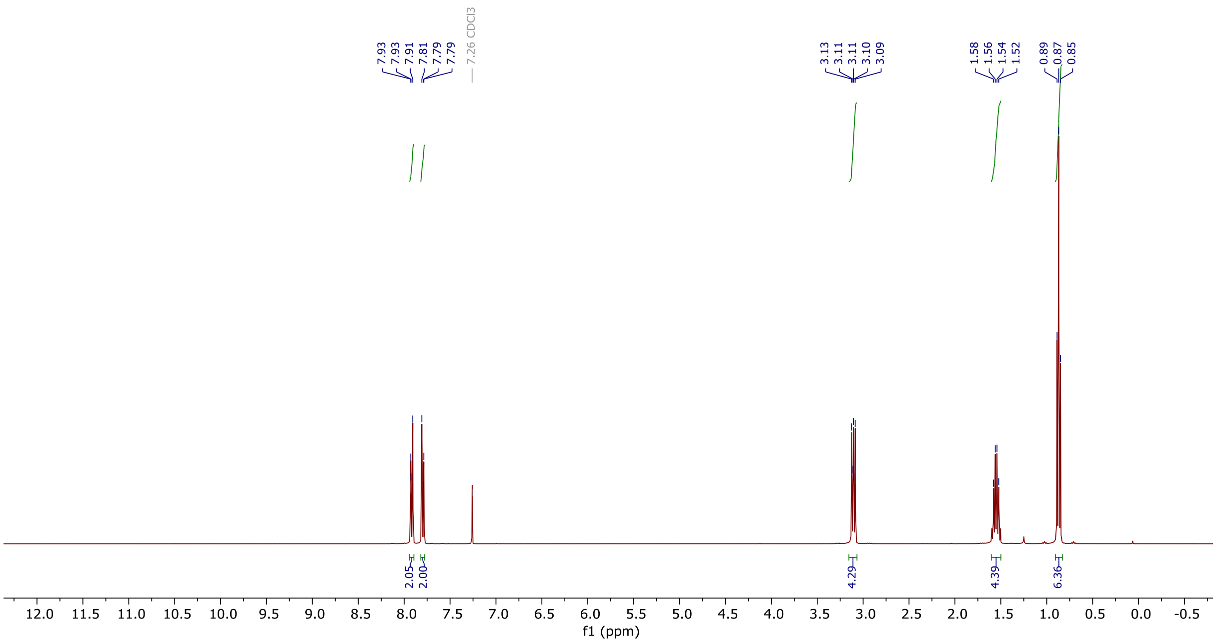

^{13}C NMR spectrum of **73**.

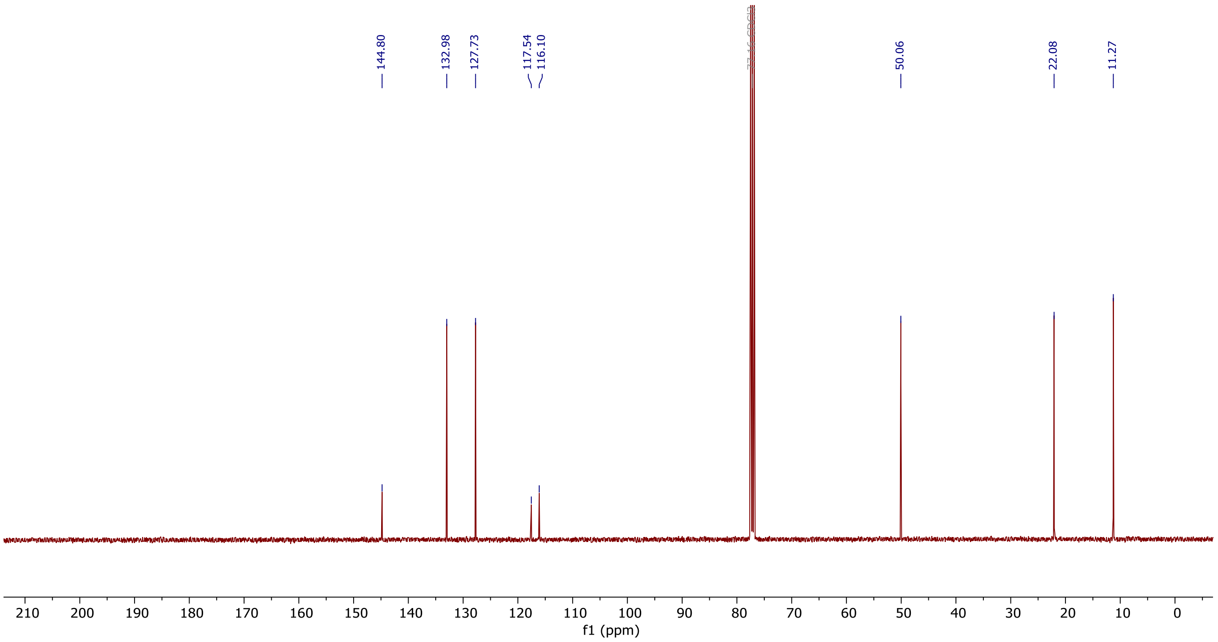

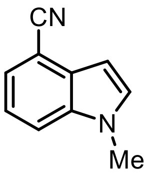

^{1}H NMR spectrum of **74**.

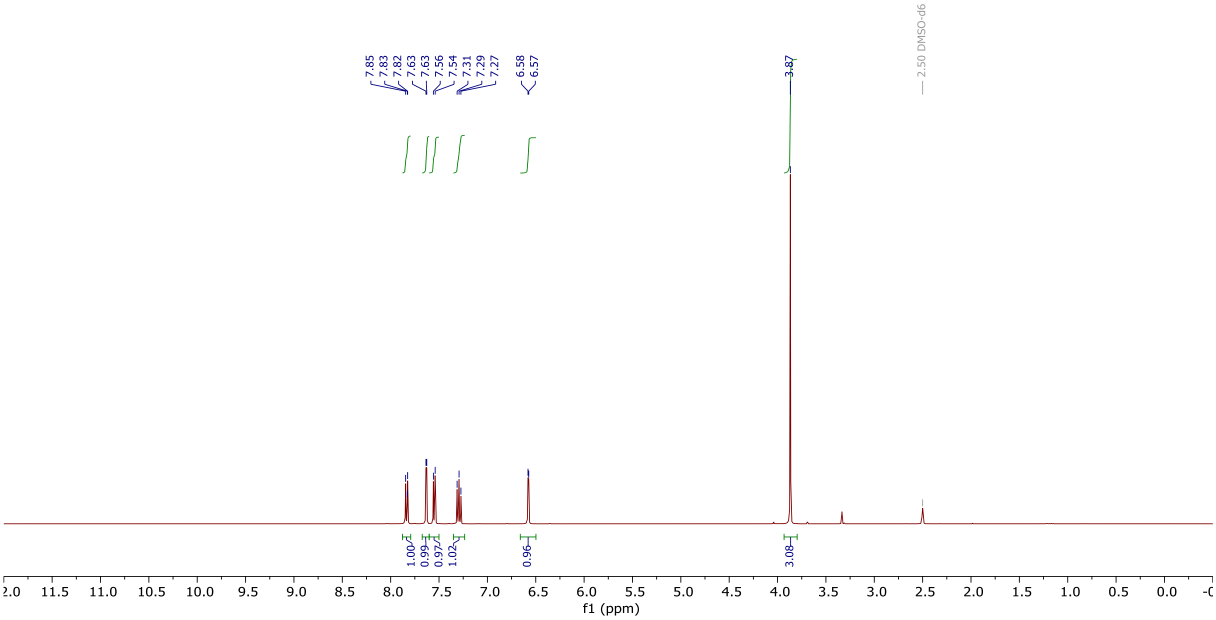

^{13}C NMR spectrum of **74**.

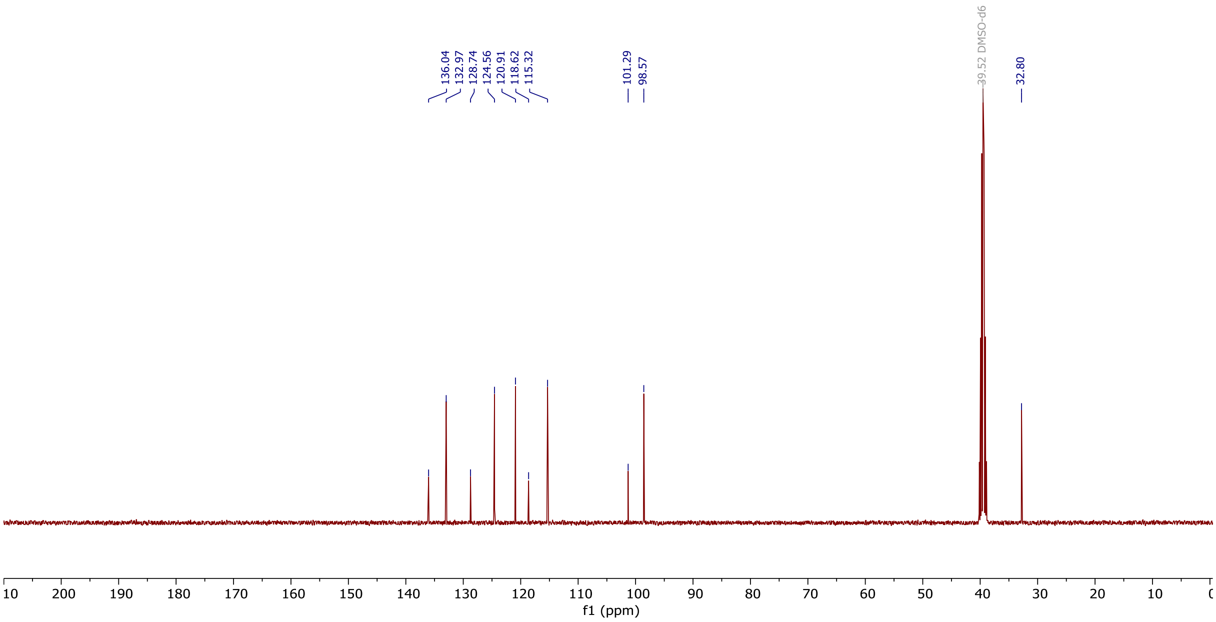

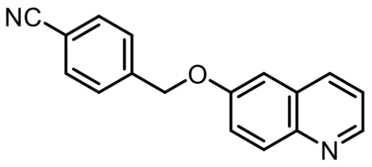

^{1}H NMR spectrum of **75**.

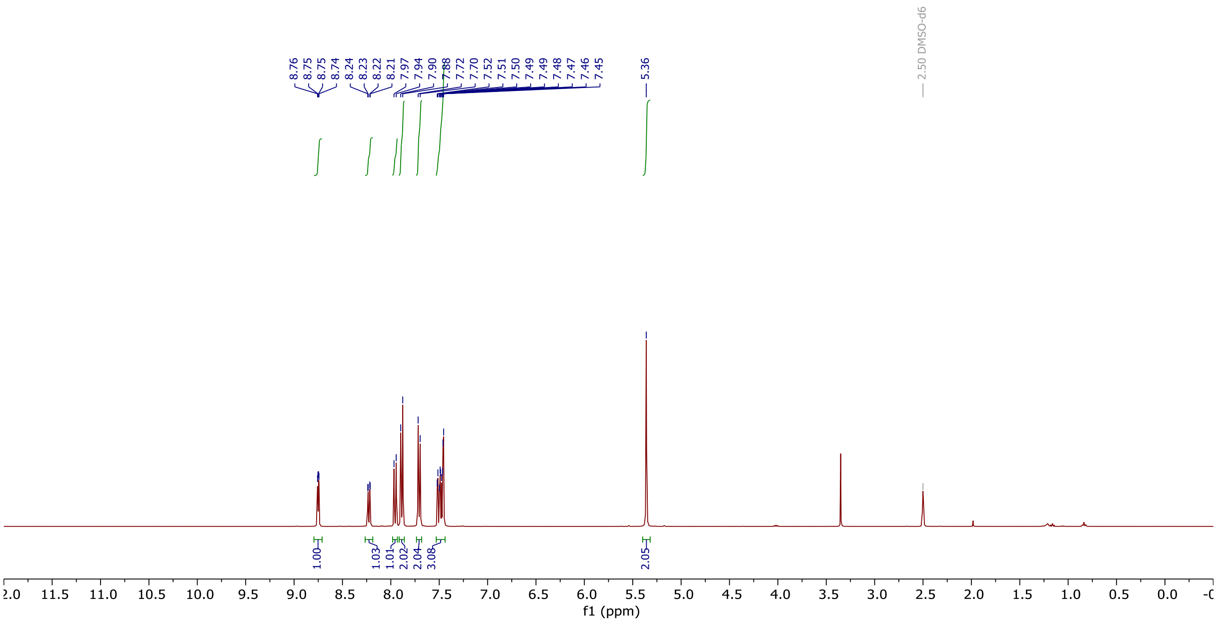

^{13}C NMR spectrum of **75**.

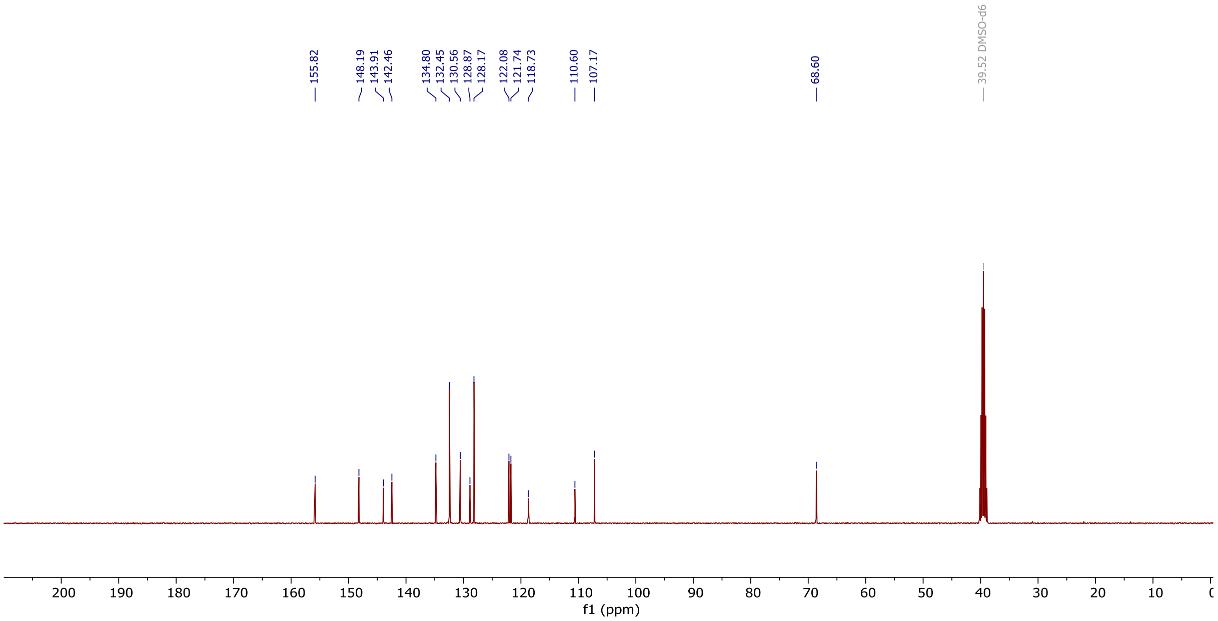

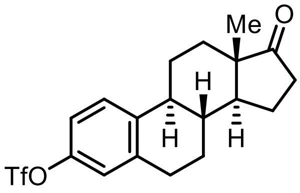

^{1}H NMR spectrum of **76**.

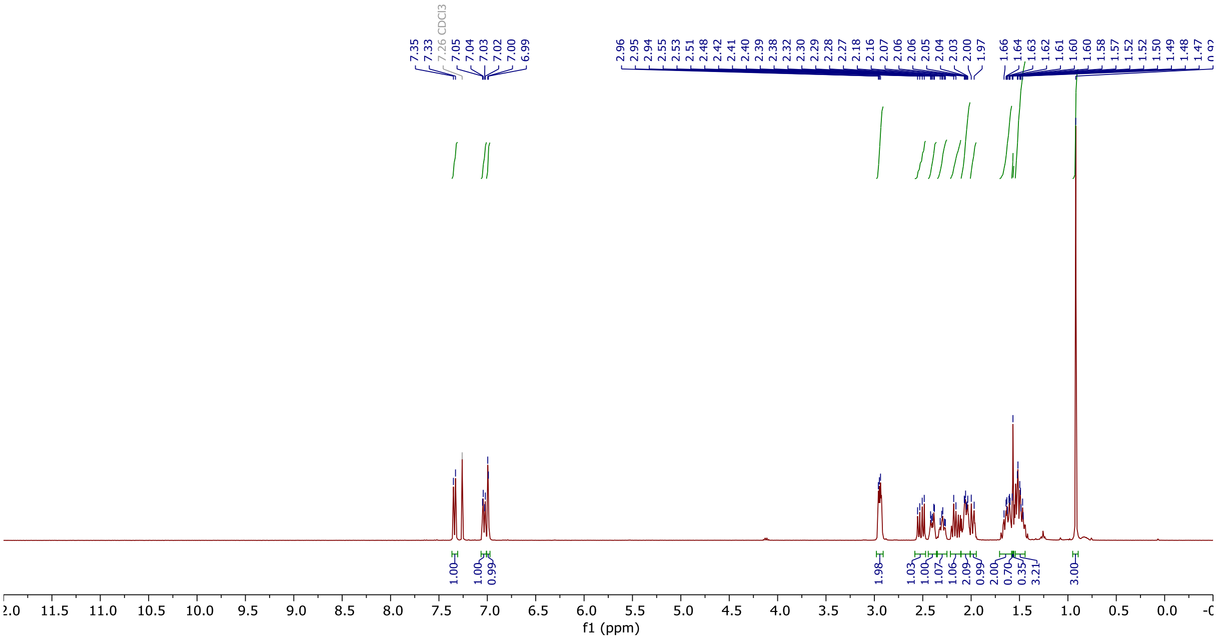

^{13}C NMR spectrum of **76**.

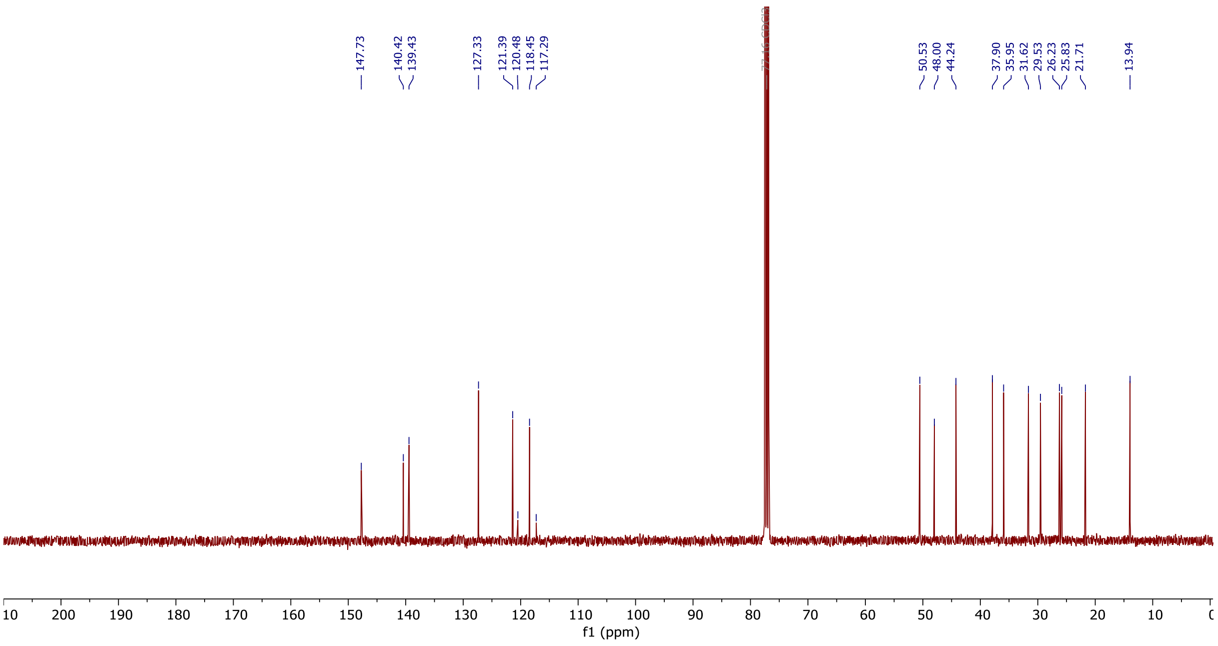

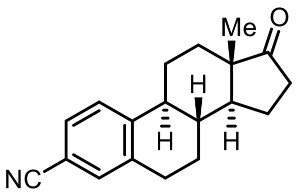

^{1}H NMR spectrum of **77**.

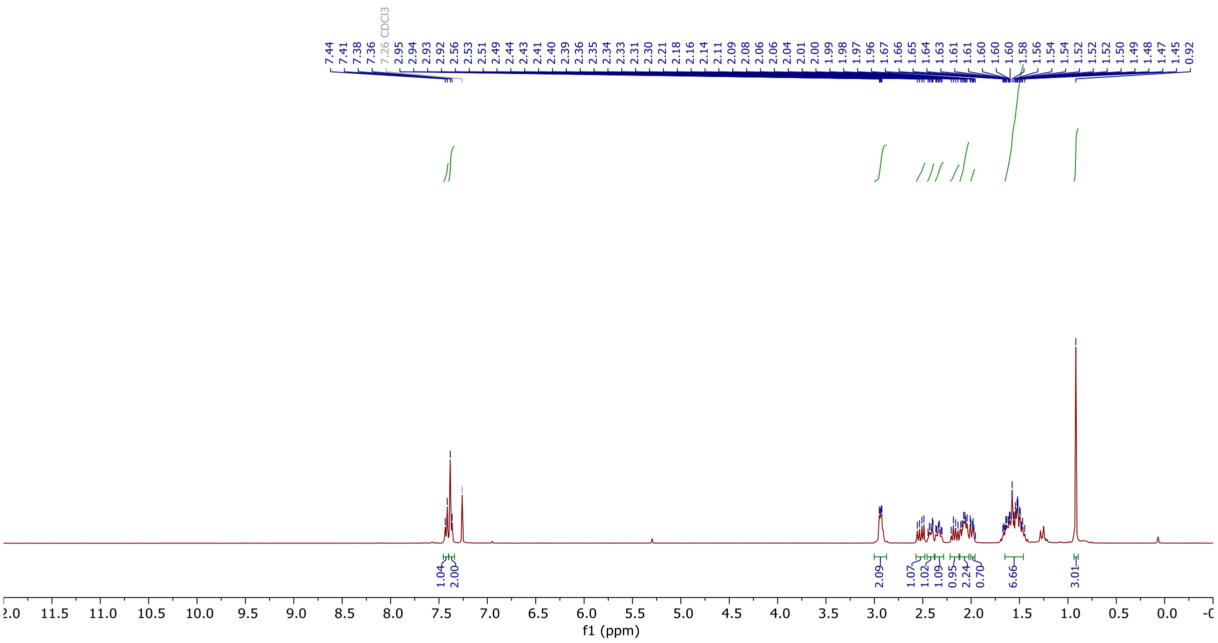

^{13}C NMR spectrum of **77**.

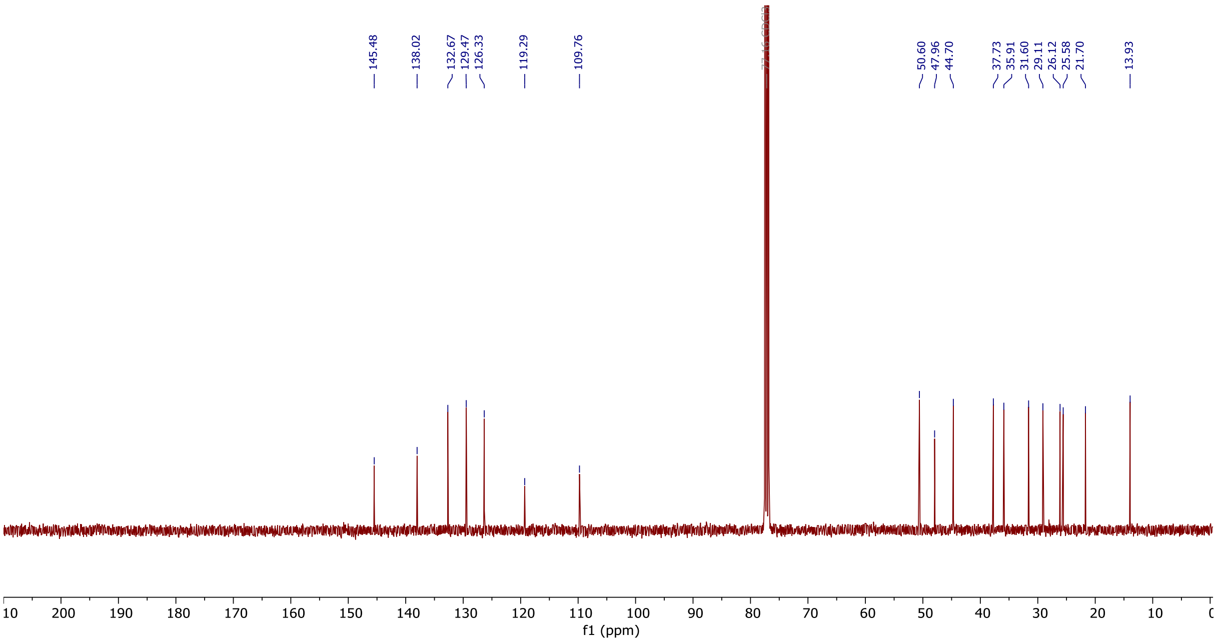

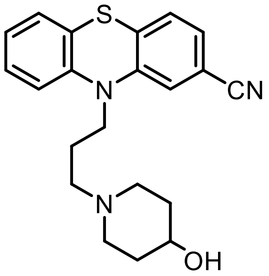

^{1}H NMR spectrum of **78**.

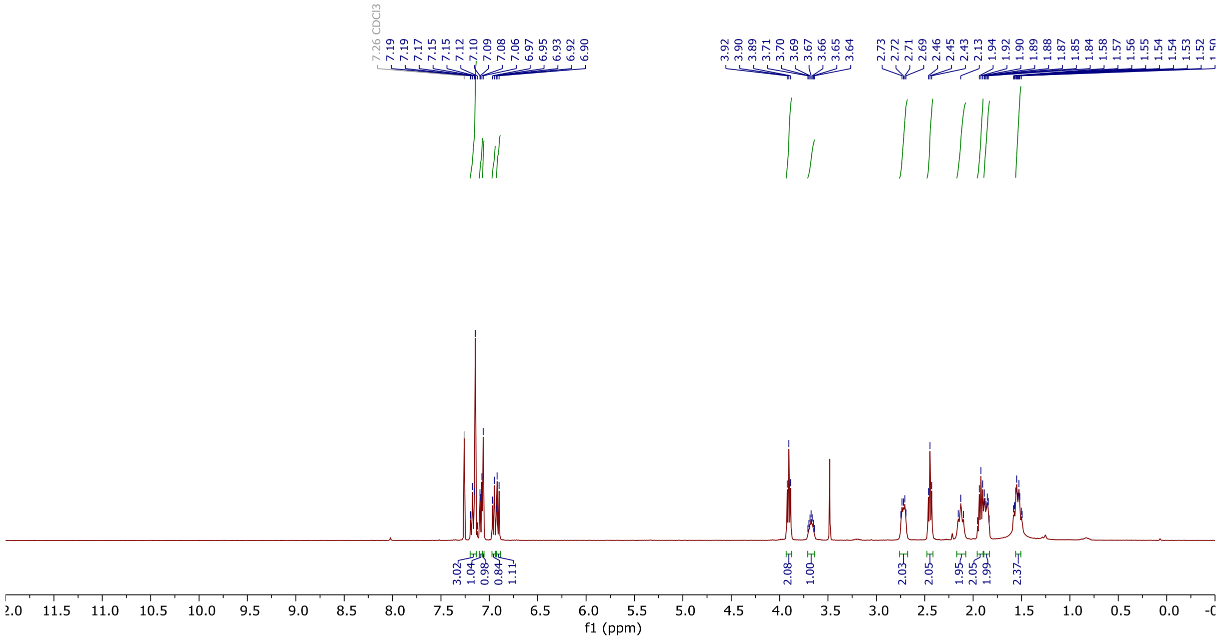

^{13}C NMR spectrum of **78**.

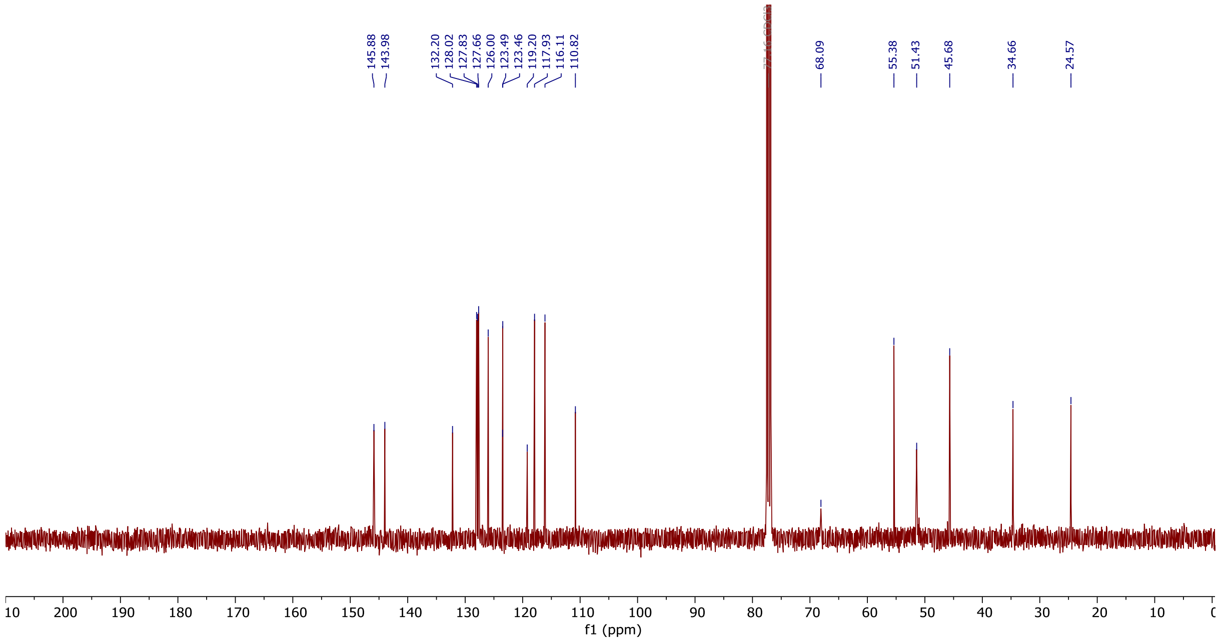

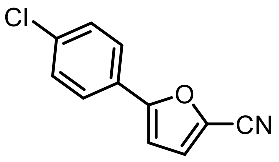

^{1}H NMR spectrum of **79**.

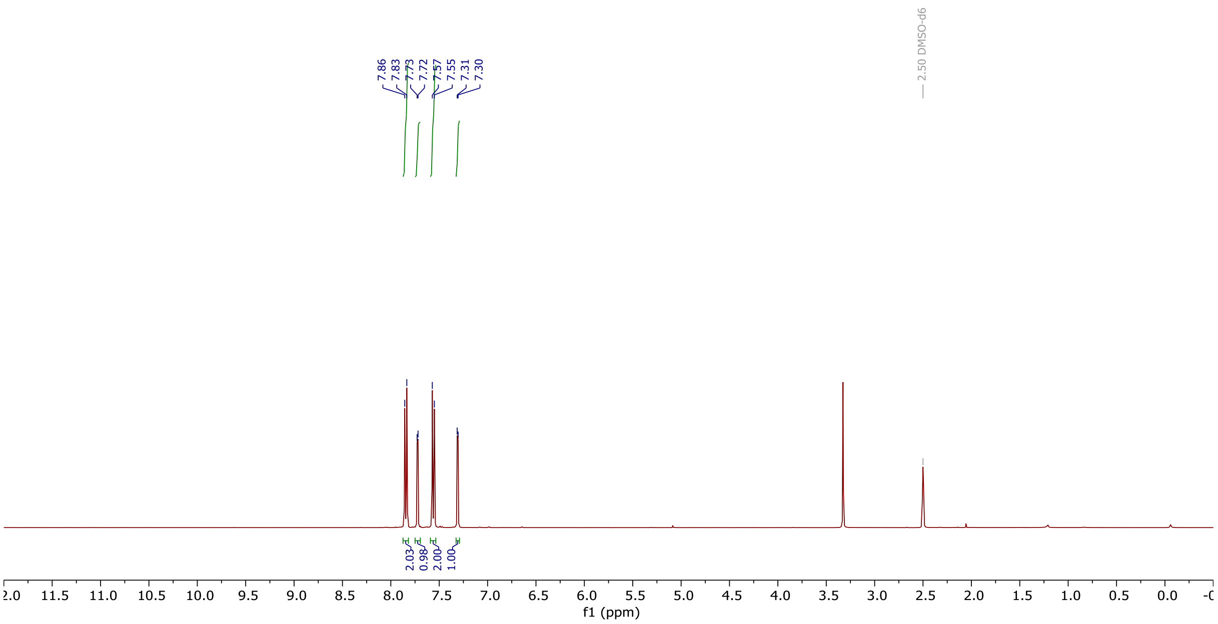

^{13}C NMR spectrum of **79**.

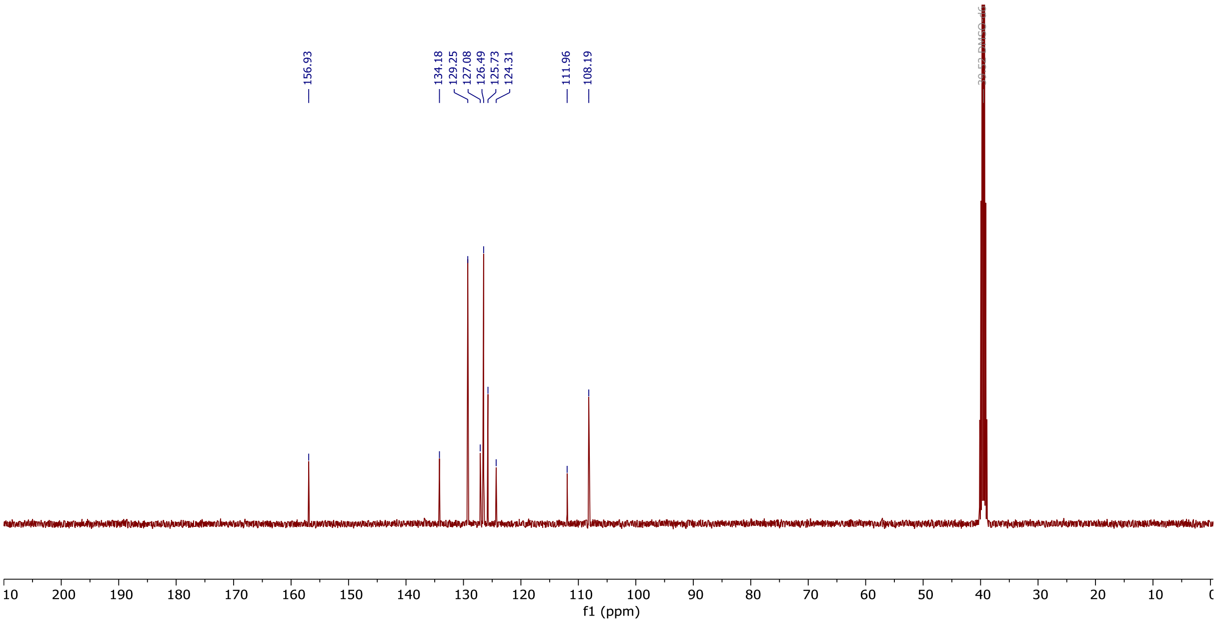

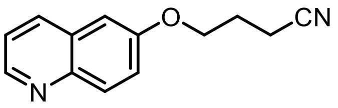

^{1}H NMR spectrum of **80**.

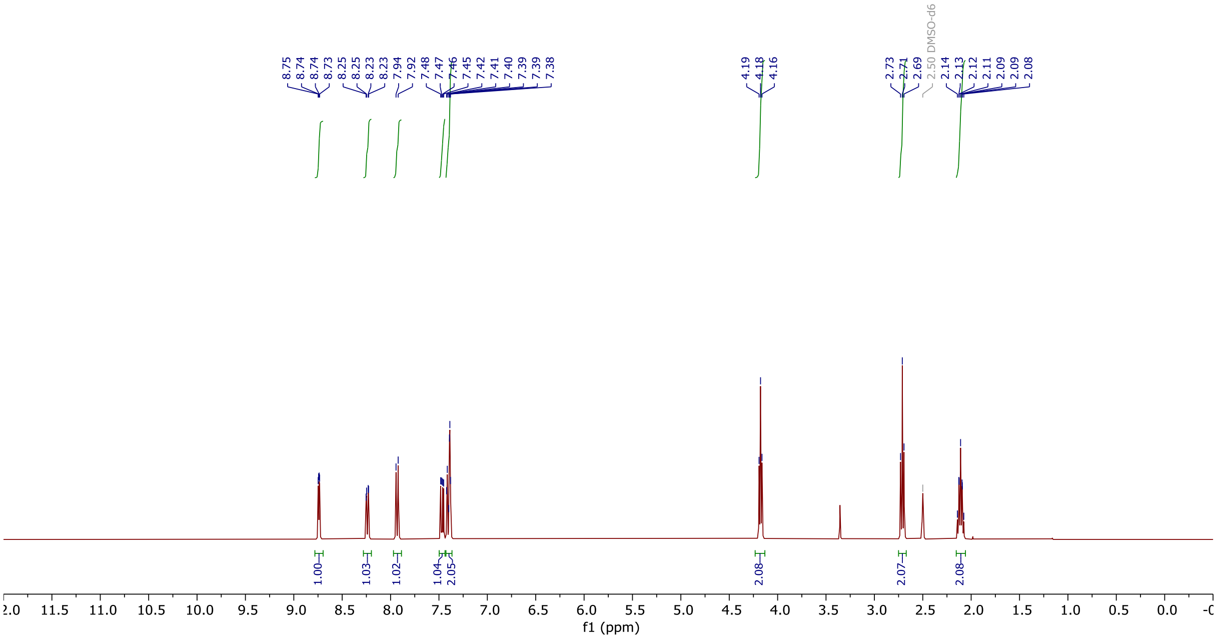

^{13}C NMR spectrum of **80**.

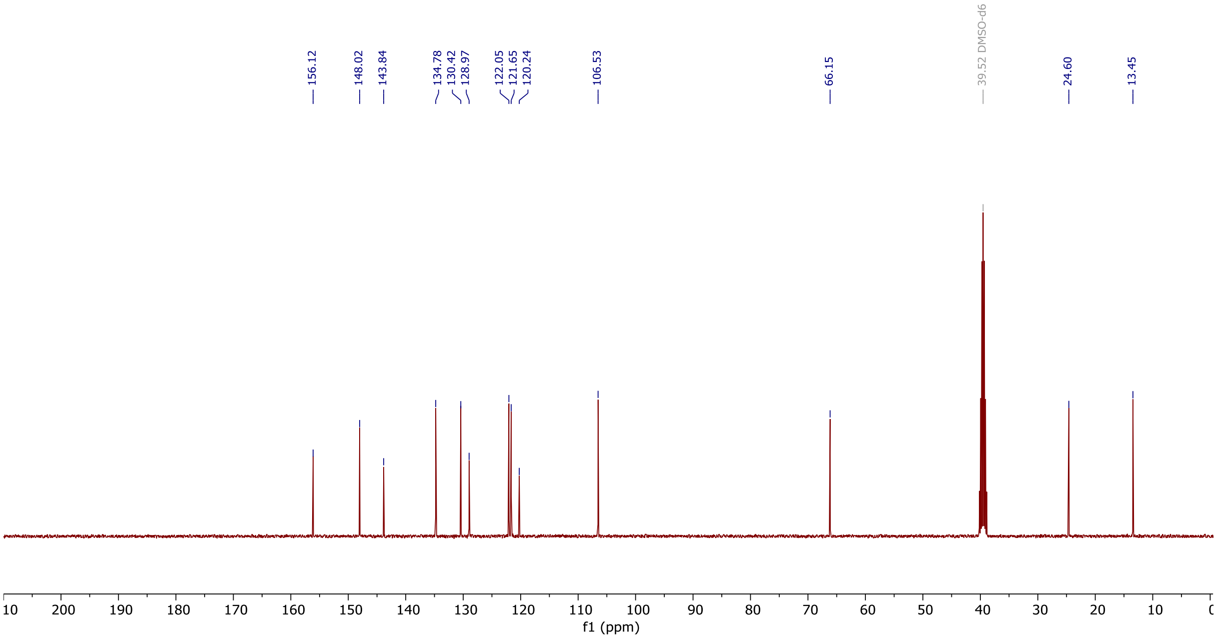

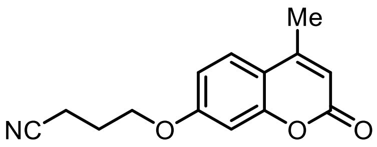

^{1}H NMR spectrum of **81**.

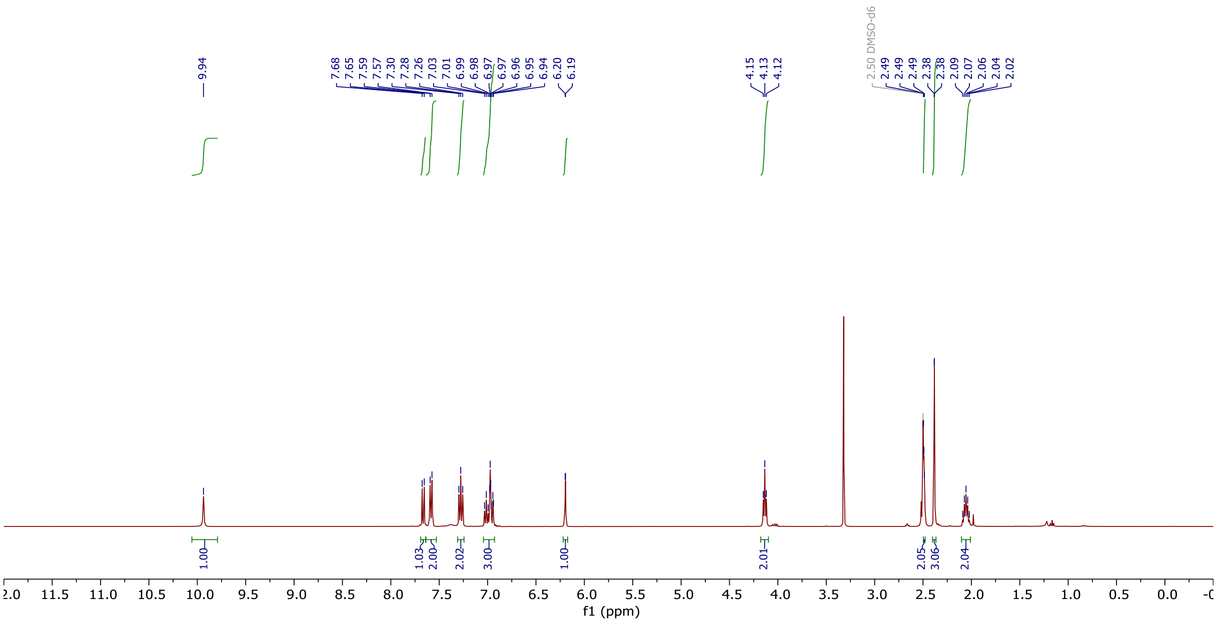

^{13}C NMR spectrum of **81**.

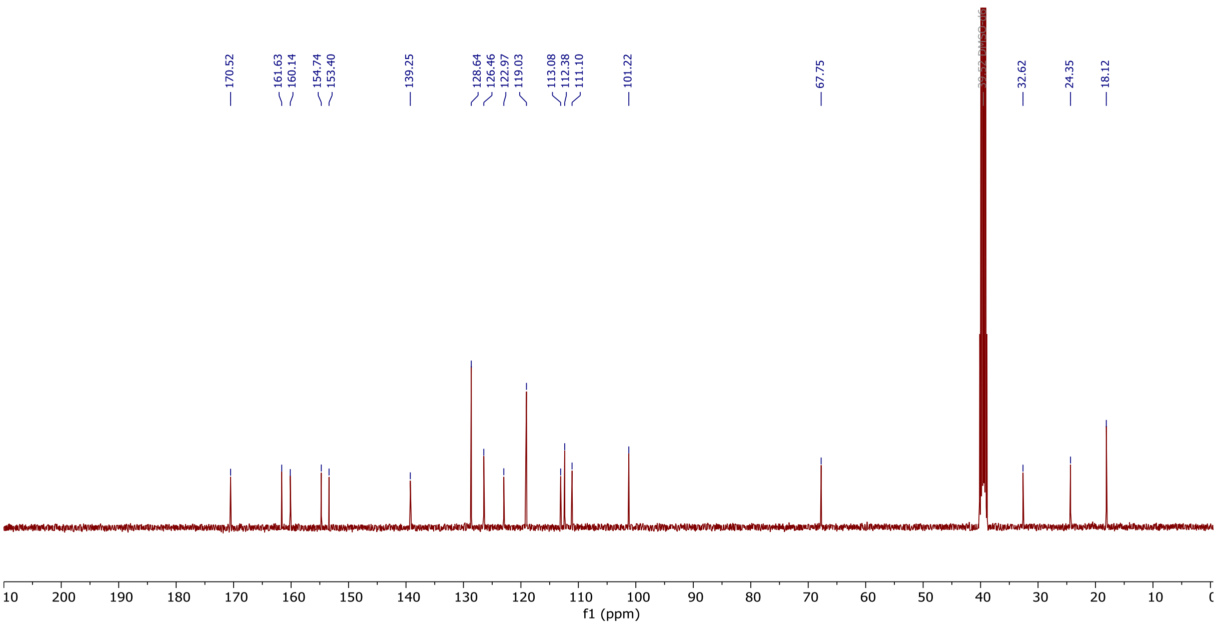

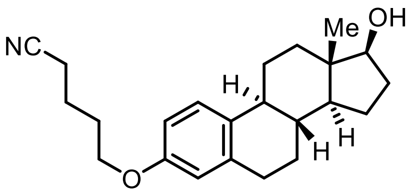

^{1}H NMR spectrum of **82**.

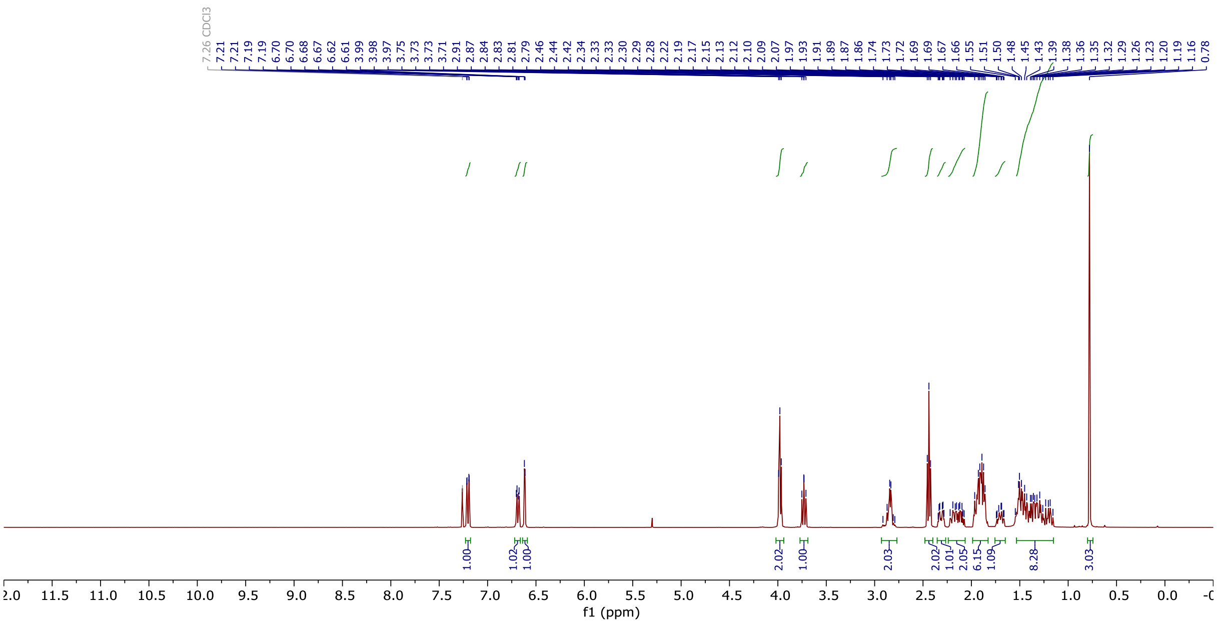

^{13}C NMR spectrum of **82**.

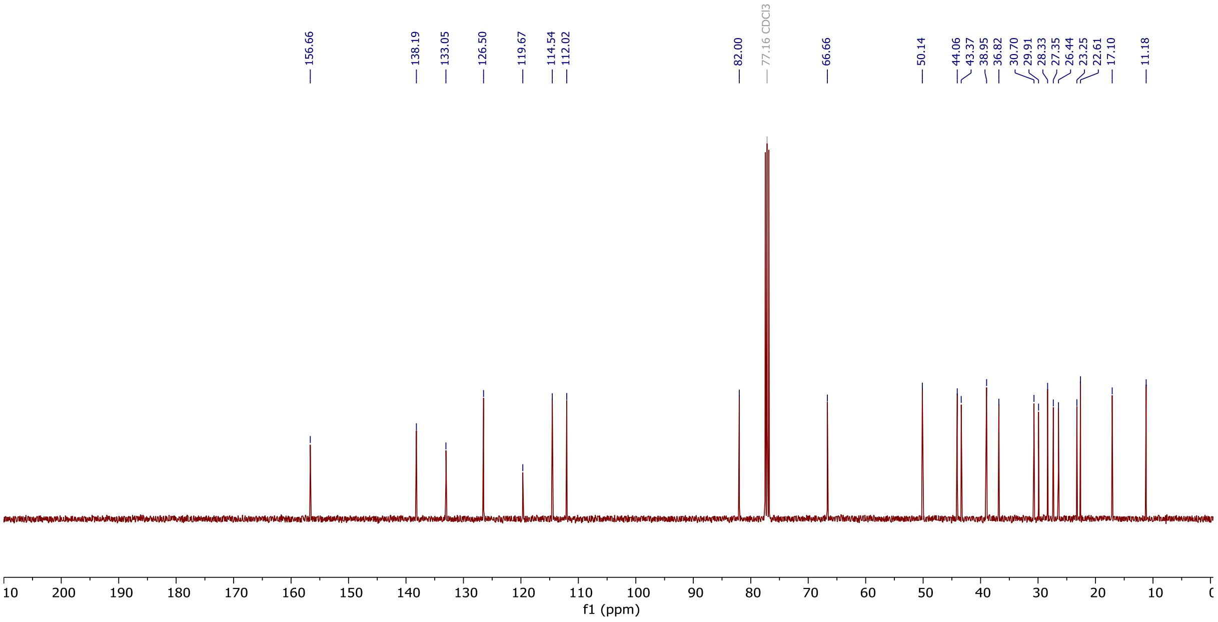

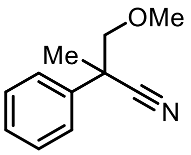

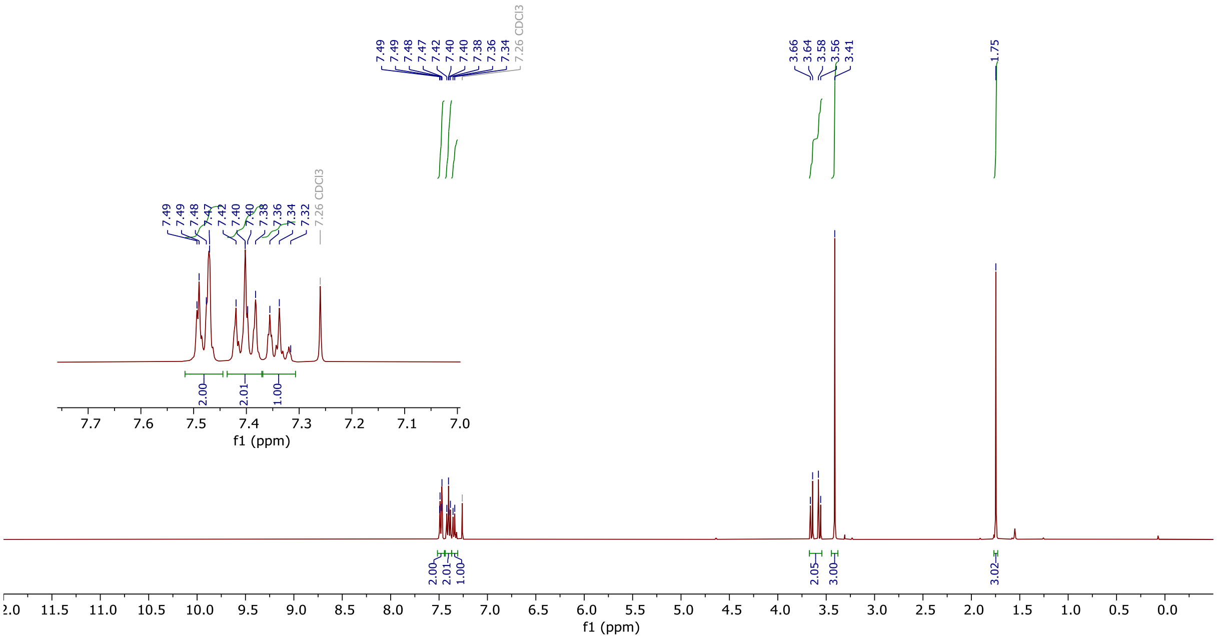

^{1}H NMR spectrum of **83**.

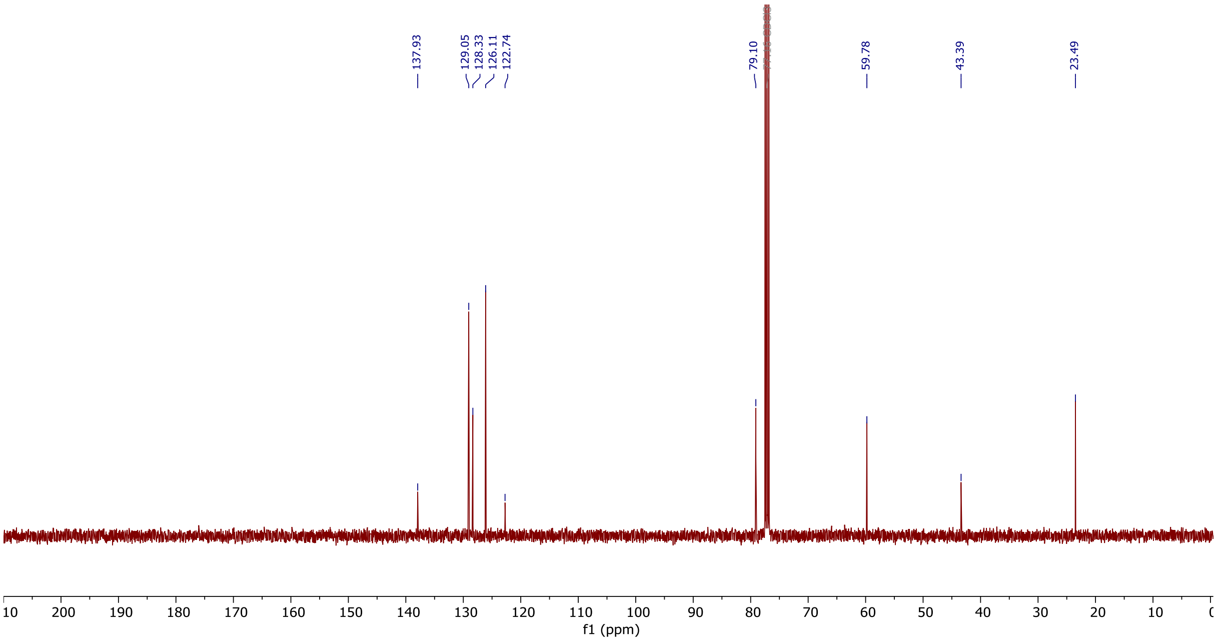

^{13}C NMR spectrum of **83**.

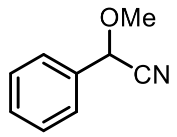

^{1}H NMR spectrum of **84**.

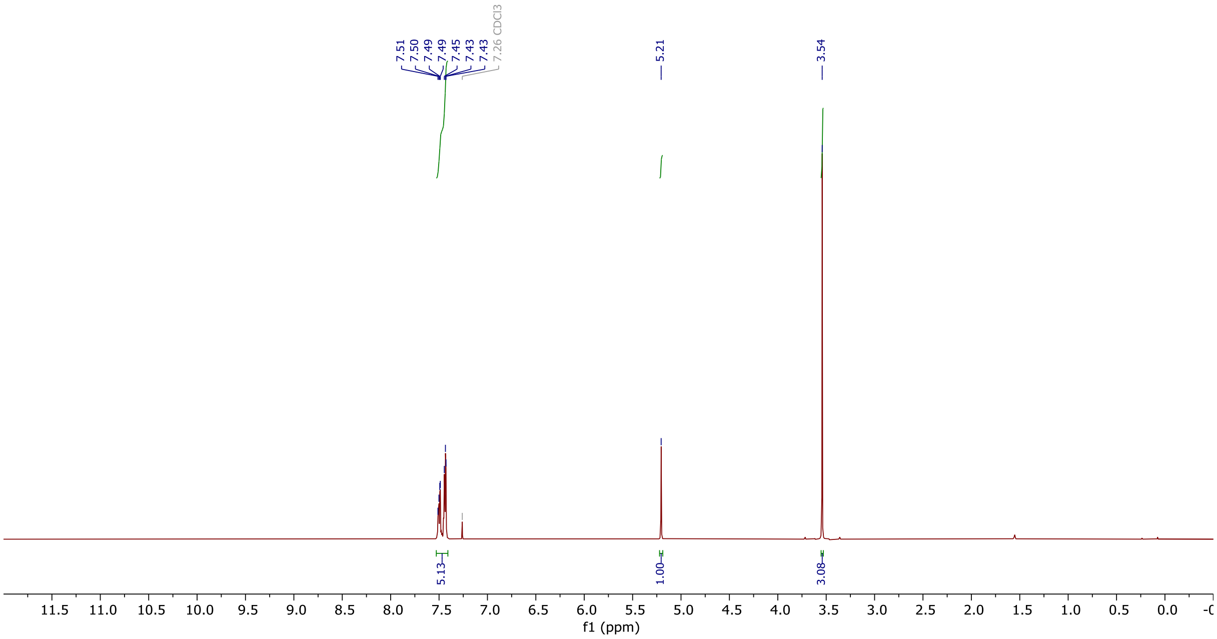

^{13}C NMR spectrum of **84**.

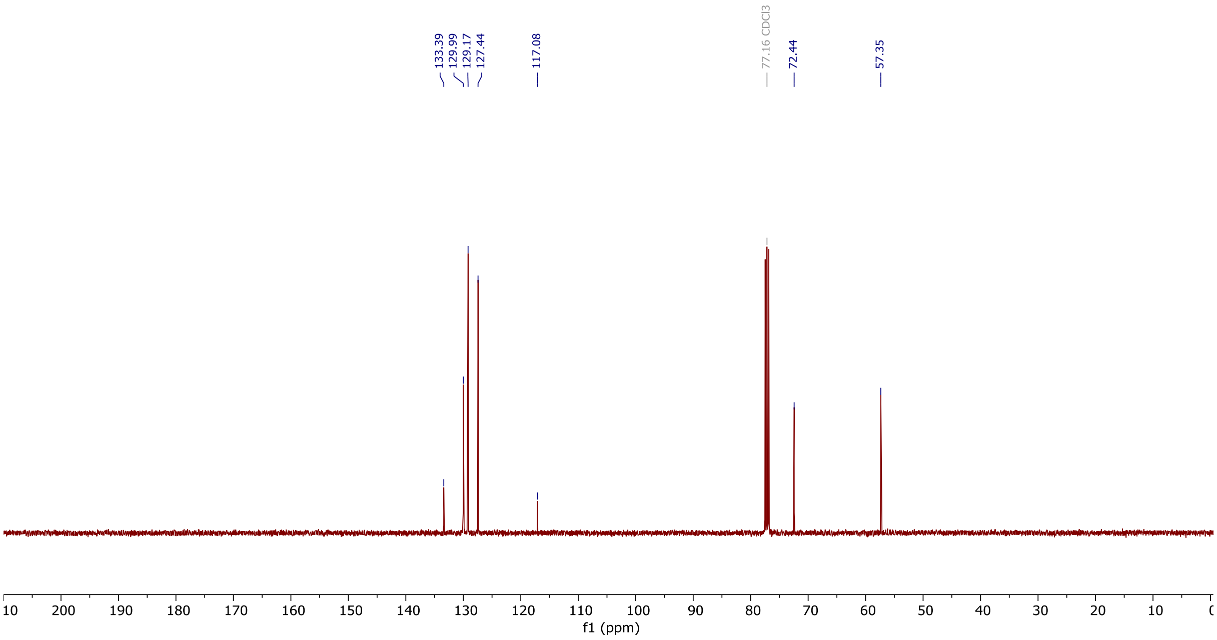

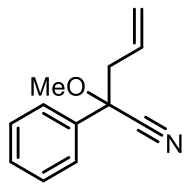

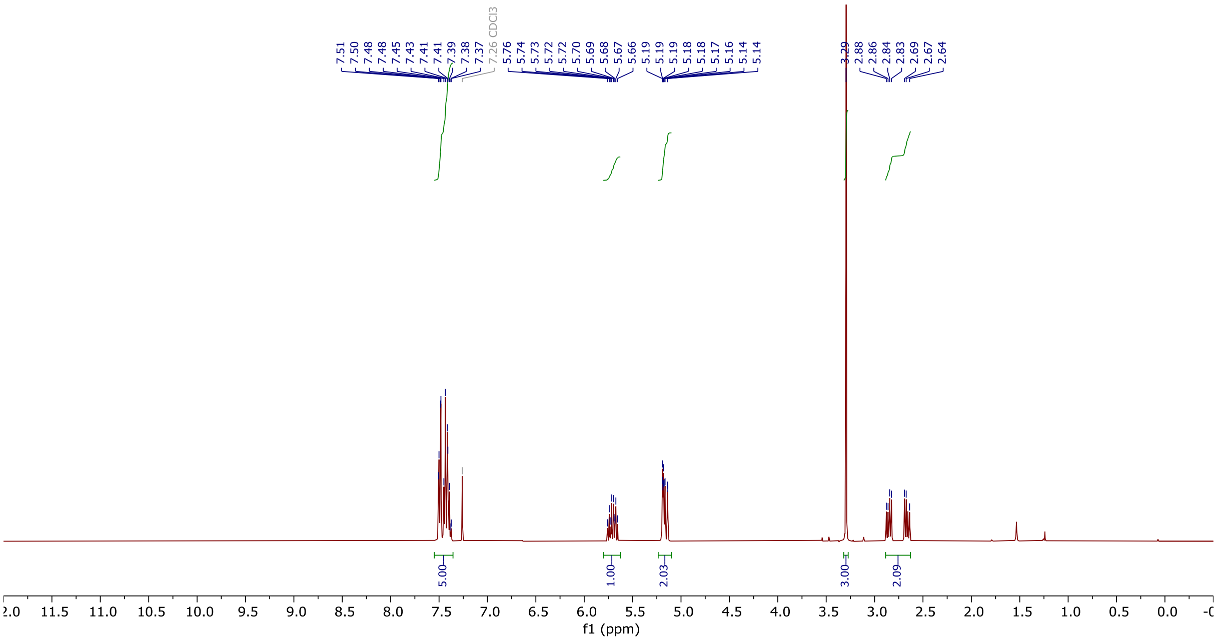

^{1}H NMR spectrum of **85**.

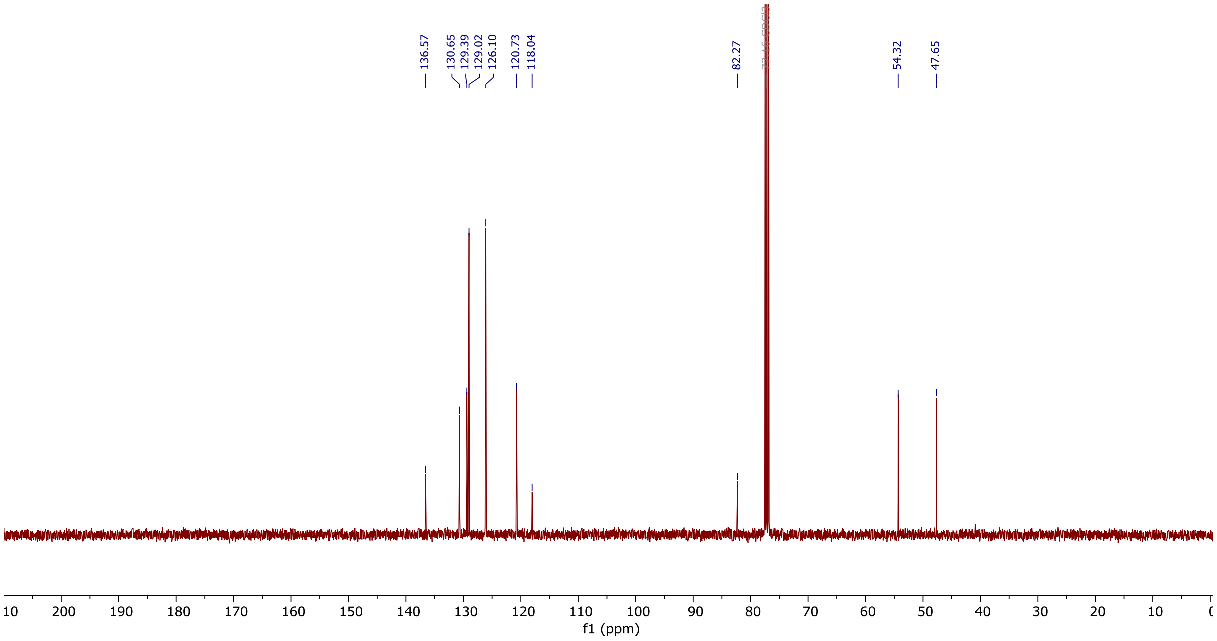

^{13}C NMR spectrum of **85**.

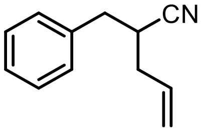

^{1}H NMR spectrum of **86**.

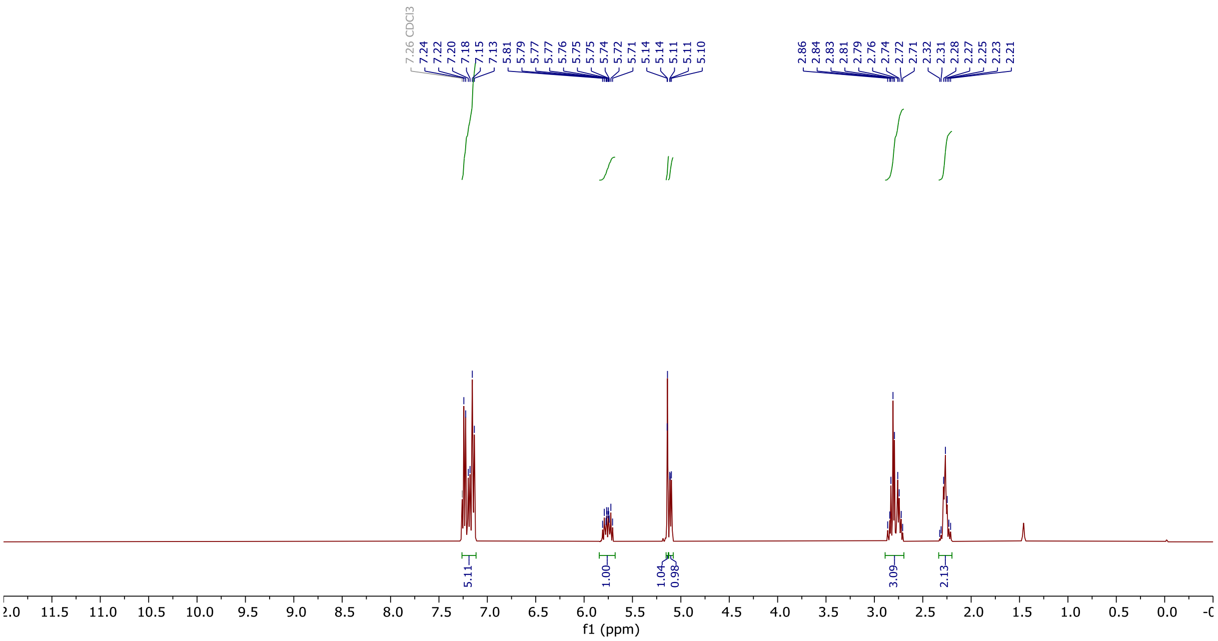

^{13}C NMR spectrum of **86**.

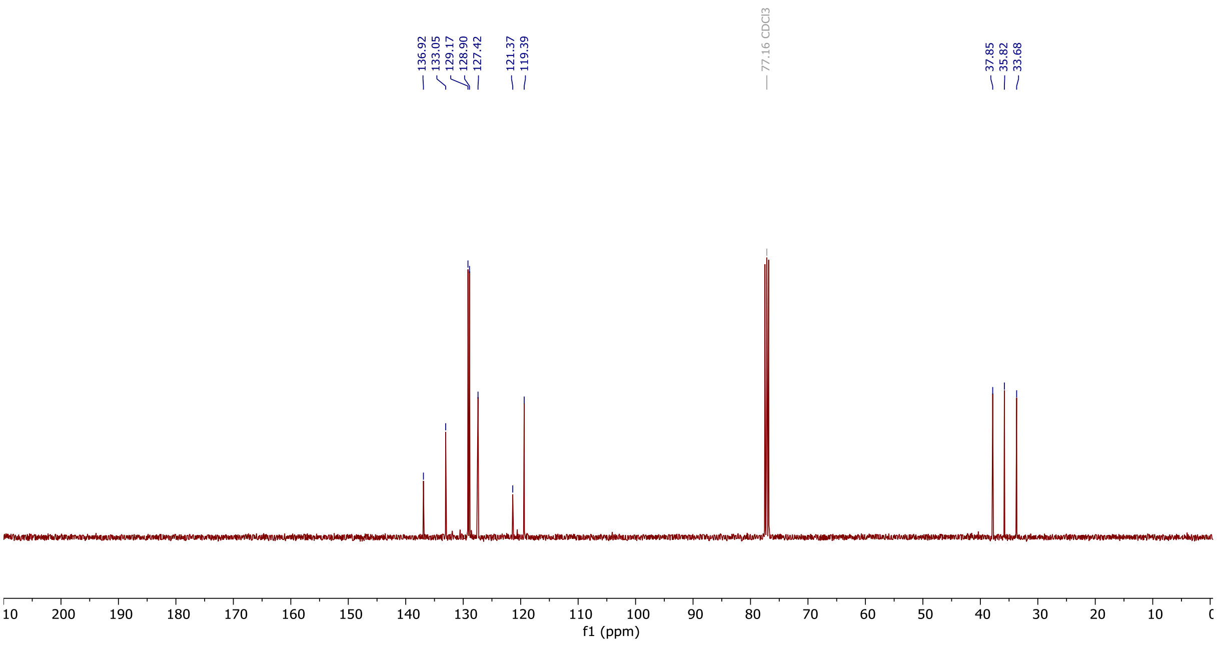

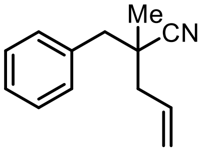

^{1}H NMR spectrum of **87**.

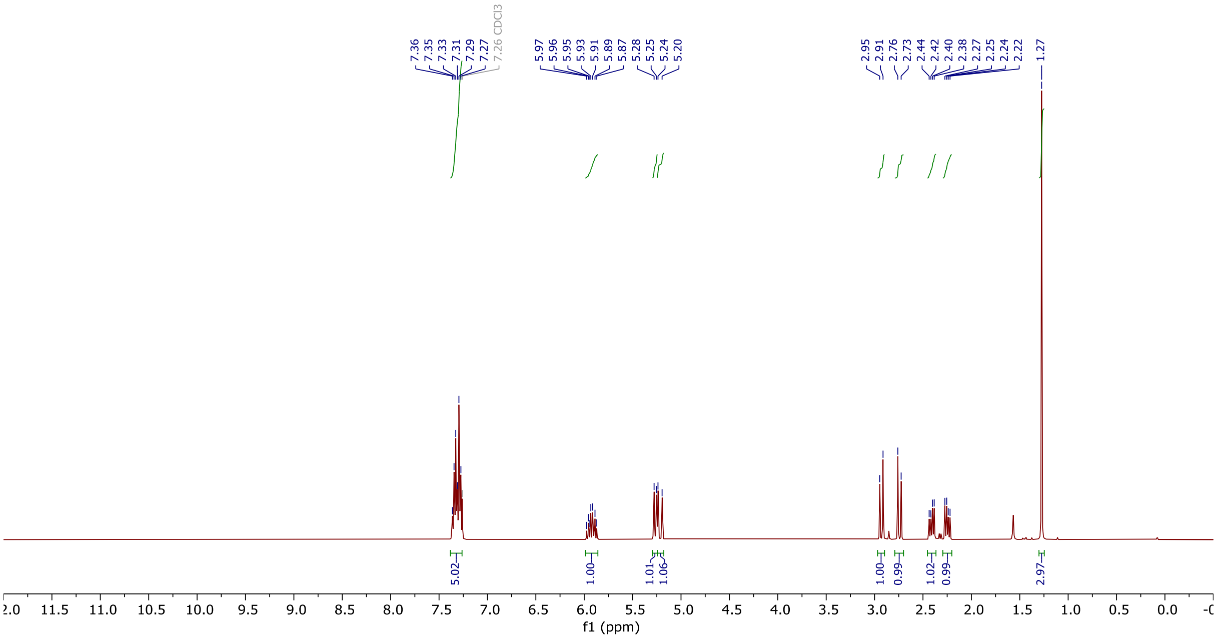

^{13}C NMR spectrum of **87**.

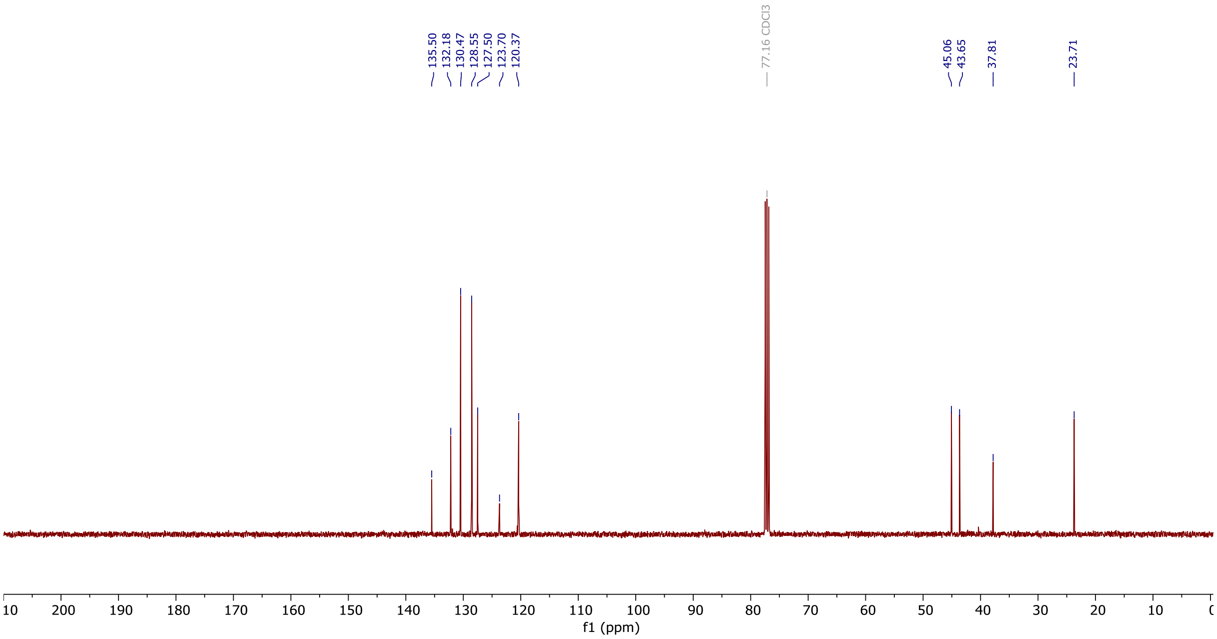

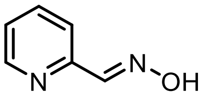

^{1}H NMR spectrum of **L10**.

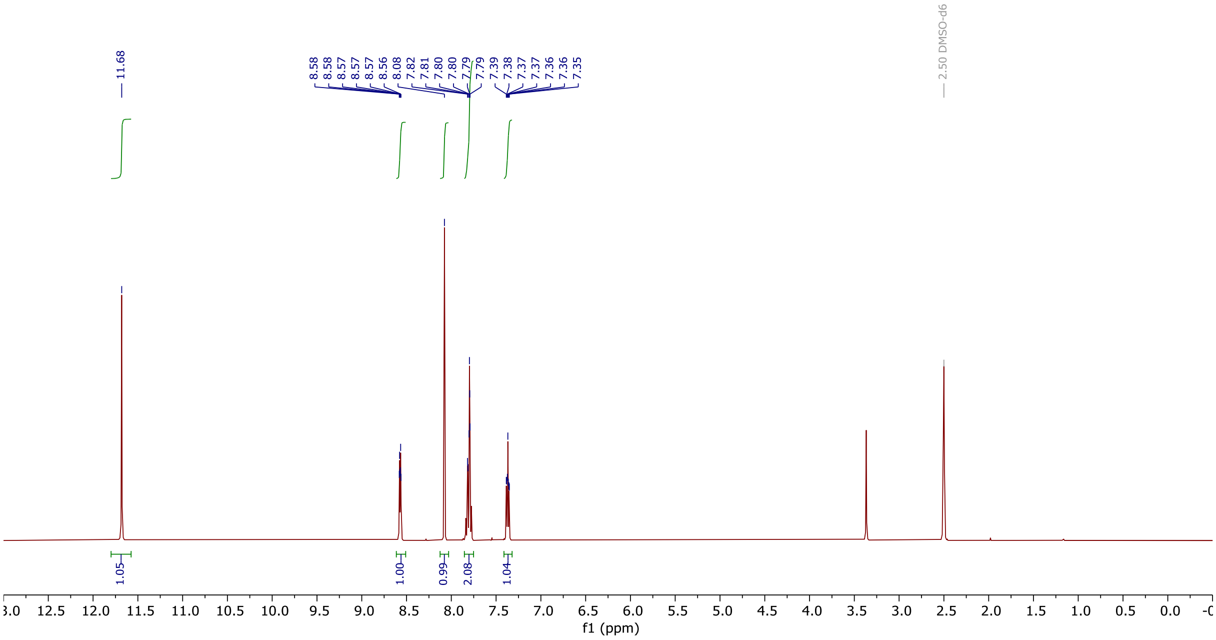

^{13}C NMR spectrum of **L10**.

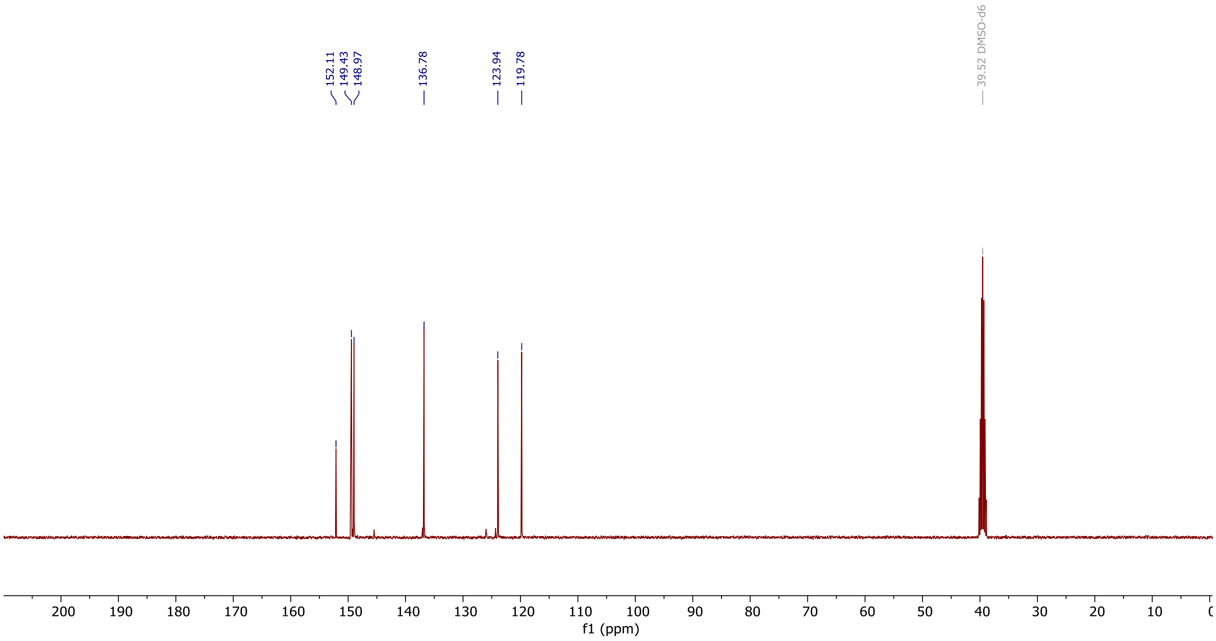

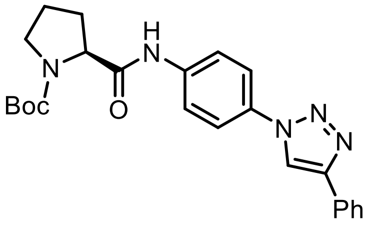

^{1}H NMR spectrum of ***tert*-Butyl (*S*)-2-((4-(4-phenyl-1H-1,2,3-triazol-1-yl)phenyl)carbamoyl)pyrrolidine-1-carboxylate**.

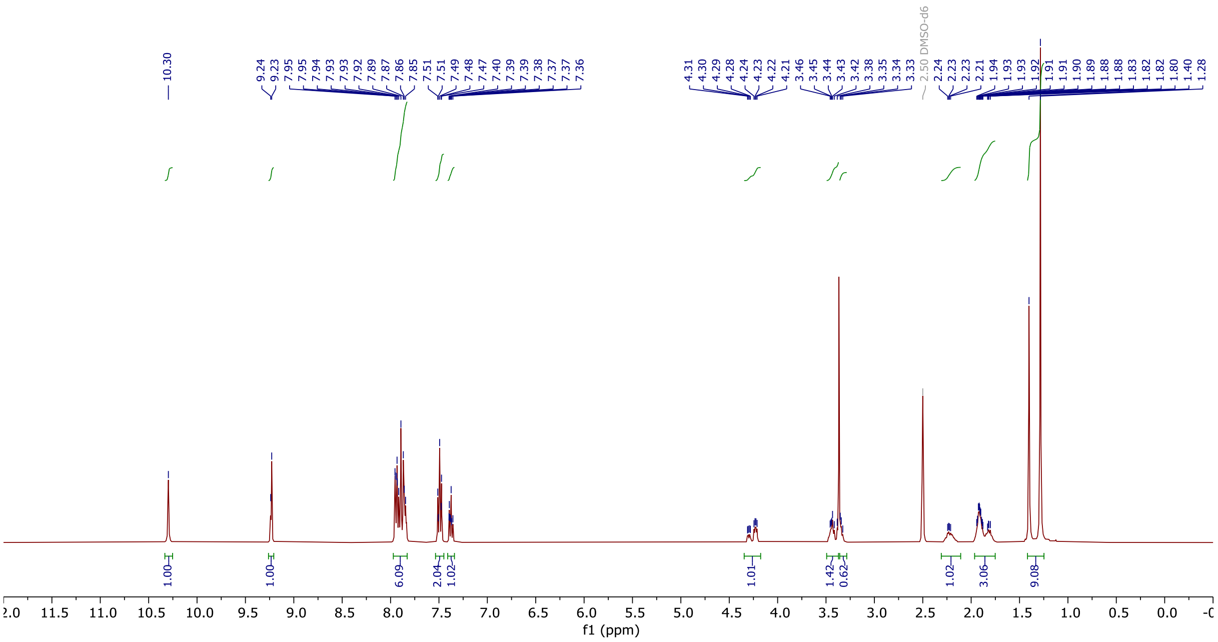

^{13}C NMR spectrum of ***tert*-Butyl (*S*)-2-((4-(4-phenyl-1H-1,2,3-triazol-1-yl)phenyl)carbamoyl)pyrrolidine-1-carboxylate**.

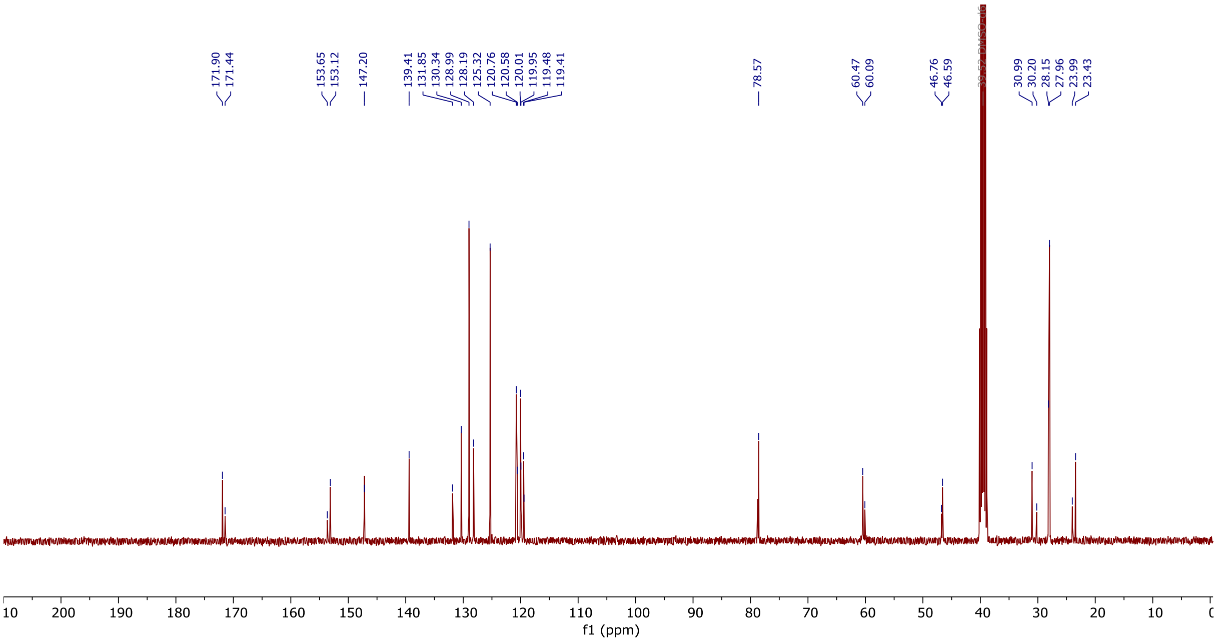

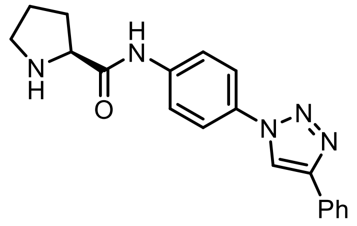

^{1}H NMR spectrum of **L11**.

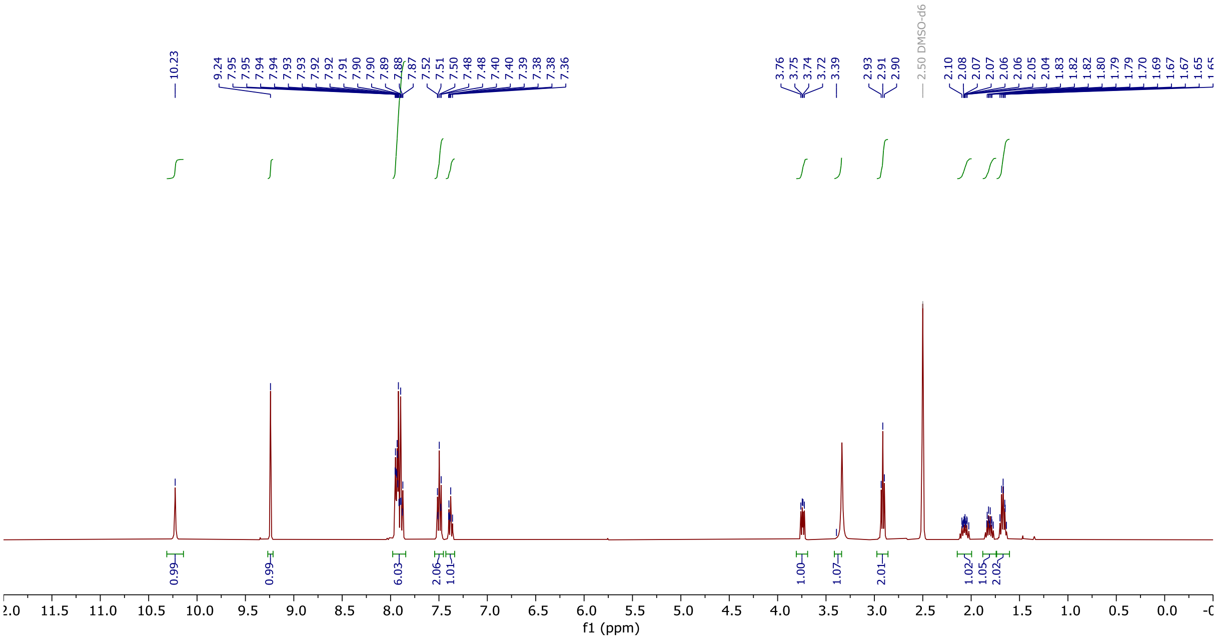

^{13}C NMR spectrum of **L11**.

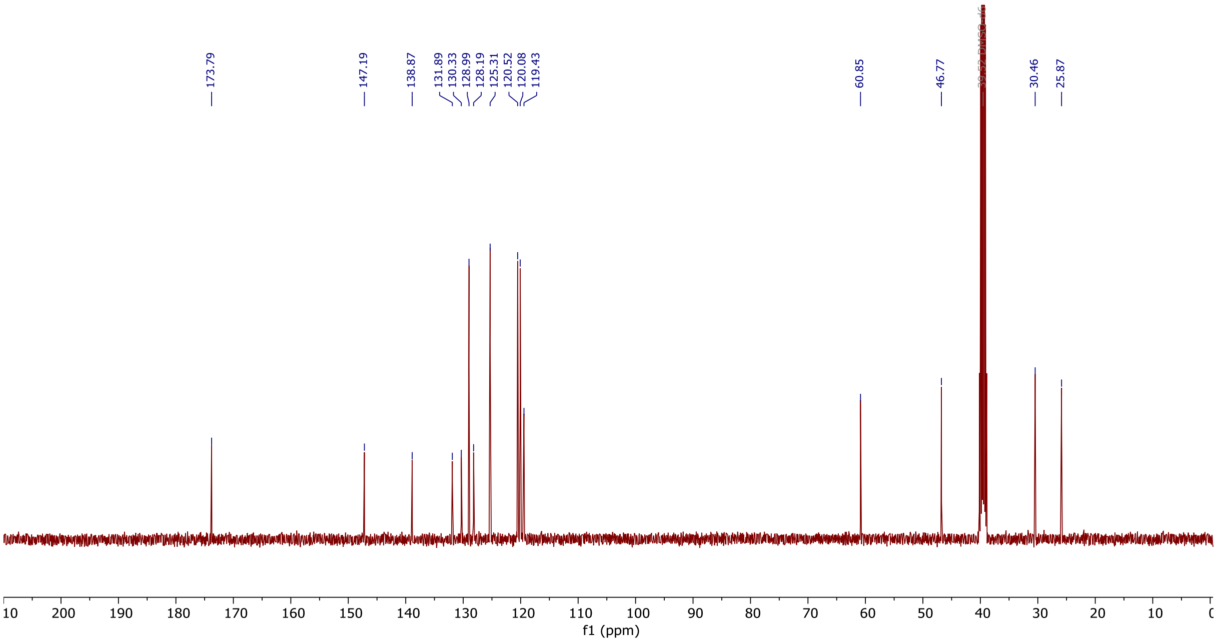

Supplementary References

1. Hou, Y., Lu, S. & Liu, G. Iodine(III)-Mediated [3 + 2] Cyclization for One-Pot Synthesis of Benzo[d]isoxazole-4,7-diols in Aqueous Medium. *J. Org. Chem.* **78**, 8386-8395 (2013).
2. Chakraborti, G., Paladhi, S., Mandal, T. & Dash, J. "On Water'' Promoted Ullmann-Type C–N Bond-Forming Reactions: Application to Carbazole Alkaloids by Selective N-Arylation of Aminophenols. *J. Org. Chem.* **83**, 7347-7359 (2018).
3. Rzeznicka, K., Schätzle, S., Böttcher, D., Klein, J. & Bornscheuer, U.T. Cloning and functional expression of a nitrile hydratase (NHase) from Rhodococcus equi TG328-2 in Escherichia coli, its purification and biochemical characterisation. *Appl. Microbiol. Biotechnol.* **85**, 1417-1425 (2010).
4. Craven, E.J., Latham, J., Shepherd, S.A., Khan, I., Diaz-Rodriguez, A., Greaney, M.F. & Micklefield, J. Programmable late-stage C−H bond functionalization enabled by integration of enzymes with chemocatalysis. *Nat Catal.* **4**, 385-394 (2021).
5. Jha, A., Chou, T.-Y., Aljaroudi, Z., Ellis, B.D. & Cameron, T.S. Aza-Diels–Alder reaction between N-aryl-1-oxo-1H-isoindolium ions and tert-enamides: Steric effects on reaction outcome. *Beilstein J. Org. Chem.* **10**, 848-857 (2014).
6. Liu, J., Tian, Y., Shi, J., Zhang, S. & Cai, Q. An Enantioselective Synthesis of Spirobilactams through Copper-Catalyzed Intramolecular Double N-Arylation and Phase Separation. *Angew. Chem. Int. Ed.* **54**, 10917-10920 (2015).
7. Turnbull, B.W.H. & Evans, P.A. Enantioselective Rhodium-Catalyzed Allylic Substitution with a Nitrile Anion: Construction of Acyclic Quaternary Carbon Stereogenic Centers. *J. Am. Chem. Soc.* **137**, 6156-6159 (2015).
8. Fleming, F.F., Liu, W., Ghosh, S. & Steward, O.W. Metalated Nitriles: Internal 1,2-Asymmetric Induction. *J. Org. Chem.* **73**, 2803-2810 (2008).
9. Tnay, Y.L., Chen, C., Chua, Y.Y., Zhang, L. & Chiba, S. Copper-Catalyzed Aerobic Spirocyclization of Biaryl-N-H-imines via 1,4-Aminooxygenation of Benzene Rings. *Org. Lett.* **14**, 3550-3553 (2012).
10. Kalinin, S., Kopylov, S., Tuccinardi, T., Sapegin, A., Dar'in, D., Angeli, A., Supuran, C.T. & Krasavin, M. Lucky Switcheroo: Dramatic Potency and Selectivity Improvement of Imidazoline Inhibitors of Human Carbonic Anhydrase VII. *ACS Med. Chem. Lett.* **8**, 1105-1109 (2017).

11. Ciszewski, Ł.W., Durka, J. & Gryko, D. Photocatalytic Alkylation of Pyrroles and Indoles with α-Diazo Esters. *Org. Lett.* **21**, 7028-7032 (2019).
12. Wintgens, V., Lorthioir, C., Miskolczy, Z., Amiel, C. & Biczók, L. Substituent Effects on the Inclusion of 1-Alkyl-6-alkoxy-quinolinium in 4-Sulfonatocalix[n]arenes. *ACS Omega* **3**, 8631-8637 (2018).
13. Taeufer, T. & Pospech, J. Palladium-Catalyzed Synthesis of N,N-Dimethylanilines via Buchwald–Hartwig Amination of (Hetero)aryl Triflates. *J. Org. Chem.* **85**, 7097-7111 (2020).
14. Cohen, D.T. & Buchwald, S.L. Mild Palladium-Catalyzed Cyanation of (Hetero)aryl Halides and Triflates in Aqueous Media. *Org. Lett.* **17**, 202-205 (2015).
15. Sasano, Y., Kogure, N., Nagasawa, S., Kasabata, K. & Iwabuchi, Y. 2-Azaadamantane N-oxyl (AZADO)/Cu Catalysis Enables Chemoselective Aerobic Oxidation of Alcohols Containing Electron-Rich Divalent Sulfur Functionalities. *Org. Lett.* **20**, 6104-6107 (2018).
16. Ismail, M.A., Arafa, R.K., Brun, R., Wenzler, T., Miao, Y., Wilson, W.D., Generaux, C., Bridges, A., Hall, J.E. & Boykin, D.W. Synthesis, DNA Affinity, and Antiprotozoal Activity of Linear Dications: Terphenyl Diamidines and Analogues. *J. Med. Chem.* **49**, 5324-5332 (2006).
17. Pardeshi, K.A., Ravikumar, G. & Chakrapani, H. Esterase Sensitive Self-Immolative Sulfur Dioxide Donors. *Org. Lett.* **20**, 4-7 (2018).
18. Zhang, G., Cui, L., Wang, Y. & Zhang, L. Homogeneous Gold-Catalyzed Oxidative Carboheterofunctionalization of Alkenes. *J. Am. Chem. Soc.* **132**, 1474-1475 (2010).
19. Peng, J.-B., Wu, F.-P., Li, D., Geng, H.-Q., Qi, X., Ying, J. & Wu, X.-F. Palladium-Catalyzed Regioselective Carbonylative Coupling/Amination of Aryl Iodides with Unactivated Alkenes: Efficient Synthesis of β-Aminoketones. *ACS Catal.* **9**, 2977-2983 (2019).
